# Proteome-wide mendelian randomization identifies causal plasma proteins in venous thromboembolism development

**DOI:** 10.1038/s10038-023-01186-6

**Published:** 2023-08-03

**Authors:** Haobo Li, Zhu Zhang, Yuting Qiu, Haoyi Weng, Shuai Yuan, Yunxia Zhang, Yu Zhang, Linfeng Xi, Feiya Xu, Xiaofan Ji, Risheng Hao, Peiran Yang, Gang Chen, Xianbo Zuo, Zhenguo Zhai, Chen Wang

**Affiliations:** 1 National Center for Respiratory Medicine; State Key Laboratory of Respiratory Health and Multimorbidity; National Clinical Research Center for Respiratory Diseases; Institute of Respiratory Medicine, Chinese Academy of Medical Sciences; Department of Pulmonary and Critical Care Medicine, Center of Respiratory Medicine, China-Japan Friendship Hospital, Beijing, China; 2 China-Japan Friendship Hospital, Chinese Academy of Medical Sciences & Peking Union Medical College, Beijing, China; 3 Capital Medical University, Beijing, China; 4 WeGene, Shenzhen, China; Hunan Provincial Key Lab on Bioinformatics, School of Computer Science and Engineering, Central South University, Changsha, China; 5 Unit of Cardiovascular and Nutritional Epidemiology, Institute of Environmental Medicine, Karolinska Institutet, Stockholm, Sweden; 6 State Key Laboratory of Respiratory Health and Multimorbidity, Department of Physiology, Institute of Basic Medical Sciences, Chinese Academy of Medical Sciences and School of Basic Medicine, Peking Union Medical College; National Center for Respiratory Medicine; Institute of Respiratory Medicine, Chinese Academy of Medical Sciences; National Clinical Research Center for Respiratory Diseases, Beijing, China; 7 Department of Pharmacy, China-Japan Friendship Hospital, Beijing, China

**Keywords:** Thromboembolism, Genome-wide association studies

## Abstract

Genome-wide association studies (GWAS) have identified numerous risk loci for venous thromboembolism (VTE), but it is challenging to decipher the underlying mechanisms. We employed an integrative analytical pipeline to transform genetic associations to identify novel plasma proteins for VTE. Proteome-wide association studies (PWAS) were determined by functional summary-based imputation leveraging data from a genome-wide association analysis (14,429 VTE patients, 267,037 controls), blood proteomes (1348 cases), followed by Mendelian randomization, Bayesian colocalization, protein-protein interaction, and pathway enrichment analysis. Twenty genetically regulated circulating protein abundances (F2, F11, ABO, PLCG2, LRP4, PLEK, KLKB1, PROC, KNG1, THBS2, SERPINA1, RARRES2, CEL, GP6, SERPINE2, SERPINA10, OBP2B, EFEMP1, F5, and MSR1) were associated with VTE. Of these 13 proteins demonstrated Mendelian randomized correlations. Six proteins (F2, F11, PLEK, SERPINA1, RARRES2, and SERPINE2) had strong support in colocalization analysis. Utilizing multidimensional data, this study suggests PLEK, SERPINA1, and SERPINE2 as compelling proteins that may provide key hints for future research and possible diagnostic and therapeutic targets for VTE.

## Introduction

Venous thromboembolism (VTE), including deep vein thrombosis (DVT) and pulmonary thromboembolism (PTE), is the third most common life-threatening cardiovascular disease after myocardial infarction and stroke. The global incidence rate of VTE is estimated to range between 115 and 269 per 100,000 and mortality rates related to VTE is estimated to range between 9.4 and 32.3 per 100,000 [1–4]. VTE is a complex disease caused by a combination of genetic predisposing factors and acquired risk factors. Additionally, more than 60% of the variation in susceptibility to common thrombosis is attributable to genetic factors [5, 6].

Genome-wide association study (GWAS) is a research method based on linkage disequilibrium in a population and uses single-nucleotide polymorphisms (SNPs) as markers to search for genetic factors associated with complex diseases, which can reveal the genetic mechanisms related to the occurrence, development, and treatment of diseases [7] in a comprehensive manner. In recent years, GWAS has been applied to uncover the genetic etiology of VTE [8–11], and several SNPs and genes have been identified to be related to its pathogenesis. For instance, coagulation factors including coagulation factor II (*F2*), coagulation factor V (*F5*), and coagulation factor XI (*F11*) are typical factors participating in the coagulation process, while protein C (*PROC*) plays a role in anticoagulation. Glycoprotein 6 (*GP6*) and phospholipase C gamma 2 (*PLCG2*) are related to platelet generation and regulation [9, 12].

Since the results of GWAS are in the form of SNPs, the isolated outcomes of GWAS are difficult to reflect the impact on genes or proteins. Recently, a novel analytical method called proteome-wide association studies (PWAS) was developed to clarify how proteins involve in the occurrence and development of diseases [13]. Previous PWAS were mostly conducted on nervous system diseases such as depression, lacunar stroke, Alzheimer’s disease, and post-traumatic stress disorder [13–16] because these studies used tissue-specific proteins instead of blood proteins [17, 18]. Recent release of data on human plasma proteomes [19] enables the explorations of the associations of proteins and the risk of blood diseases, such as VTE.

In this study, we performed PWAS combining the data from GWAS and protein quantitative trait locus (pQTL) to investigate the proteins deserved further investigation as diagnostic and therapeutic targets for VTE. Mendelian randomization (MR) analysis and Bayesian colocalization analysis were also conducted to clarify the causal relationship between the discovered proteins and VTE pathogenesis. Protein-protein interaction (PPI), and pathway enrichment were applied to explore the potential mechanisms of candidate proteins.

## Materials and methods

### Summary statistics of genome-wide association studies

We generated genome-wide association study summary statistics from the UK Biobank (UKB, access code 56,719) [20]. We identified 14,429 unrelated British Caucasian cases based on a self-report questionnaire (1068, 1093, 1094 from data field 20002) and hospital records (ICD-10: I260, I269, I801, I802, I803, I808, I809, I828, I829, O223, O229, O871; ICD-9: 41511, 41512, 41513, 41519, 45111, 45119, 4512, 4519, 4531, 4532, 45340, 45341, 45342, 4539; OPER4: L791, L902). Patients diagnosed with portal vein thrombosis (ICD-10: I81), Budd-Chiari syndromes (ICD-10: I820), and other coagulation defects (ICD-10: D68.X) were excluded from the cases. We then selected 267,037 unrelated controls of the same ancestry to form a cohort of 281,466 individuals.

For UKB data, we performed variant level quality control by criteria of MAF > 0.01, genotype missingness <0.02, and pHWE >1 × 10^–10^ on 96 million imputed variants. We then obtained 8,473,913 variants for downstream analysis. All genotyped variants passing quality control on autosomal chromosomes were tested for association with VTE through logistic regression adjusting for age, sex, and top ten principal components using PLINK [21].

### Human blood proteome reference weight for PWAS

We next obtained whole-blood pQTL data from the Atherosclerosis Risk in Communities (ARIC) study [19] including 1348 cis-heritable plasma proteins from 7213 European Americans (EA) to match the GWAS datasets. Proteomic profiling was performed using the SomaScan technology using the v.4.1 platform. Genotyping was conducted using the Infinium Multi-Ethnic Global BeadChip array (Illumina, GenomeStudio) and imputed to the TOPMed reference panel (Freeze 5 on GRCh38). These pQTL data were used as reference weights for subsequent PWAS analysis.

### PWAS

Using the FUSION pipeline (http://gusevlab.org/projects/fusion/), we integrated the GWAS summary statistics with the reference human plasma proteomes (ARIC study [19]) to perform the PWAS analysis [22]. We calculated the VTE genetic effect (PWAS z-score) and combined it with the pre-calculated plasma proteome reference weight (z-score × proteome weight) to evaluate the effects of significant SNPs in the GWAS on the protein abundance. Finally, FUSION identified candidate genes associated with VTE regulating the abundance of proteins in the plasma. To control the potential effect of multiple testing on the study results, the false discovery rate (FDR) of *P* value < 0.05 was used as the significance threshold in our PWAS analysis.

### MR analysis

MR was used to verify whether PWAS-significant genes were associated with VTE via their cis-regulated plasma protein abundance [23, 24]. In the MR analysis, protein-relevant SNPs were used as instrumental variables (IVs) to test the causal effect of the exposure (protein expression) on the outcome (VTE). For MR analysis, the inverse variance weighted (IVW) method [25] was used as the main MR analyses. The MR-Egger [26] method was used to detect directional pleiotropy according to the intercept of weighted linear regression of the SNP‐outcome coefficients on SNP‐exposure coefficients. We used the default parameters and *P* value < 0.0025 was set as the significance level (0.05/20 = 0.0025).

### Bayesian colocalization analysis

We applied COLOC to assess the probability of the same variant being responsible for both changing VTE risk and protein expression [27]. We used the FUSION parameter to perform colocalization based on the GWAS and pQTL data. We used the default COLOC priors of *p*1 = 10^−4^, *p*2 = 10^−4^, and *p*12 = 10^−5^, where *p*1 is the probability that a given variant is associated with VTE, *p*2 is the probability that a given variant is a significant pQTL, and *p*12 is the probability that a given variant is significant in both GWAS and pQTL. A posterior colocalization probability (PP4) of 80% was used to denote a shared causal signal. The regional association plots were generated by the R package “LocusCompareR” [28].

### Protein-protein interaction and pathway enrichment analysis

We used STRING, a web platform to investigate networks among the 20 significant proteins based on PPI [29]. Additionally, Gene Ontology (GO) and Kyoto Encyclopedia of Genes and Genomes (KEGG) enrichment assays were performed to conduct gene set enrichment analysis on genes within a PPI network using the R package “clusterProfiler” [30].

### Expression analysis of candidate proteins

GSE19151 [31] (https://www.ncbi.nlm.nih.gov/geo/, accessed on 5 February 2023) provided proteins expression in healthy and VTE blood tissues. We verified whether candidate proteins were differentially expressed in VTE patients compared with healthy controls. GSE19151 contained 95 blood tissue samples from Caucasians (47 VTE patients and 48 healthy controls). Differentially expression between VTE patients and healthy controls were screened with *P* < 0.0083 (0.05/6 = 0.0083).

## Results

### PWAS identifies 20 candidate proteins associated with VTE using plasma pQTL

**Fig. 1:**
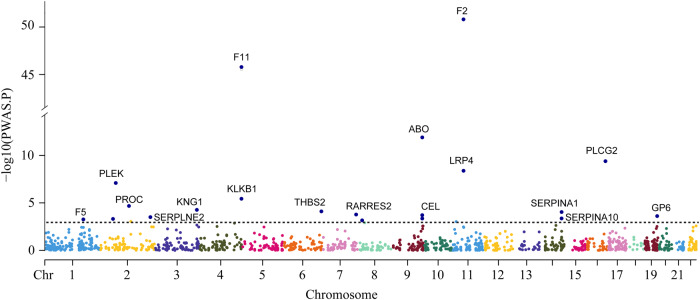
Manhattan plot for the VTE PWAS integrating the VTE GWAS (*N* = 281,466) with the ARIC proteomes (*n* = 1348). Each point represents a single test of association between a gene and VTE ordered by genomic position on the x-axis and the association strength on the y-axis as the -log_10_ *P* value of a z-score test. The PWAS identified 20 genes whose cis-regulated plasma protein abundances were associated with VTE at FDR *p* < 0.05. The red horizontal line reflects the significant threshold of FDR *p* < 0.05

There are 1529 genome-wide significant loci for GWAS of VTE (Supplementary Figs. 1 and 2). The PWAS conducted in the GWAS identified 20 genes whose cis-regulated plasma protein levels were associated with VTE at a FDR of *P* < 0.05 (Table 1, Fig. 1, Supplementary Table 1, and Supplementary Fig. 2) [F2, F11, ABO Blood Group (ABO), PLCG2, LDL receptor-related protein 4 (LRP4), pleckstrin (PLEK), kallikrein B1 (KLKB1), PROC, kininogen 1 (KNG1), thrombospondin 2 (THBS2), serpin family A member 1 (SERPINA1), retinoic acid receptor responder 2 (RARRES2), carboxyl ester lipase (CEL), GP6, serpin family E member 2 (SERPINE2), serpin family A member 10 (SERPINA10), odorant binding protein 2B (OBP2B), EGF containing fibulin extracellular matrix protein 1 (EFEMP1), F5, and macrophage scavenger receptor 1 (MSR1)].

**Table 1:** The results of the PWAS of VTE, followed by Mendelian randomization and COLOC

Protein	CHR	PWAS			MR		COLOC	Causal MR or COLOC
		PWAS z-score	PWAS *P*	PWAS FDR *P*	MR *P*	Causal	PP4	
F2	11	15.0	8.2 × 10^–51^	1.1 × 10^–47^	2.5 × 10^–31^	yes	1.00	yes
F11	4	14.2	8.5 × 10^–46^	5.7 × 10^–43^	5.4 × 10^–32^	yes	1.00	yes
ABO	9	7.1	1.3 × 10^–12^	5.7 × 10^–10^	6.8 × 10^–12^	yes	0.00	yes
PLCG2	16	6.3	4.1 × 10^–10^	1.4 × 10^–07^	5.3 × 10^–09^	yes	0.03	yes
LRP4	11	5.9	4.2 × 10^–09^	1.1 × 10^–06^	4.6 × 10^–08^	yes	0.00	yes
PLEK	2	–5.4	8.1 × 10^–08^	1.8 × 10^–05^	2.6 × 10^–12^	yes	1.00	yes
KLKB1	4	4.6	3.7 × 10^–06^	7.1 × 10^–04^	0.050	no	0.00	no
PROC	2	–4.3	2.1 × 10^–05^	3.5 × 10^–03^	2.0 × 10^–04^	yes	0.61	yes
KNG1	3	4.0	5.4 × 10^–05^	8.1 × 10^–03^	3.4 × 10^–07^	yes	0.68	yes
THBS2	6	–3.9	7.9 × 10^–05^	0.011	6.6 × 10^–13^	yes	0.29	yes
SERPINA1	14	–3.9	9.2 × 10^–05^	0.011	6.6 × 10^–08^	yes	0.96	yes
RARRES2	7	–3.8	1.7 × 10^–04^	0.019	0.017	no	0.80	yes
CEL	9	–3.7	2.0 × 10^–04^	0.020	0.085	no	0.00	no
GP6	19	3.7	2.5 × 10^–04^	0.024	0.039	no	0.69	no
SERPINE2	2	–3.6	3.2 × 10^–04^	0.028	5.2 × 10^–06^	yes	0.91	yes
SERPINA10	14	–3.5	4.3 × 10^–04^	0.036	9.9 × 10^–04^	yes	0.26	yes
OBP2B	9	–3.5	4.4 × 10^–04^	0.035	0.124	no	0.00	no
EFEMP1	2	–3.5	4.9 × 10^–04^	0.037	4.3 × 10^–08^	yes	0.11	yes
F5	1	3.5	5.5 × 10^–04^	0.039	0.536	no	0.00	no
MSR1	8	–3.4	6.9 × 10^–04^	0.047	4.3 × 10^–03^	no	0.35	no

### MR verifies the causal relationship of 13 proteins with VTE using plasma pQTL

**Fig. 2:**
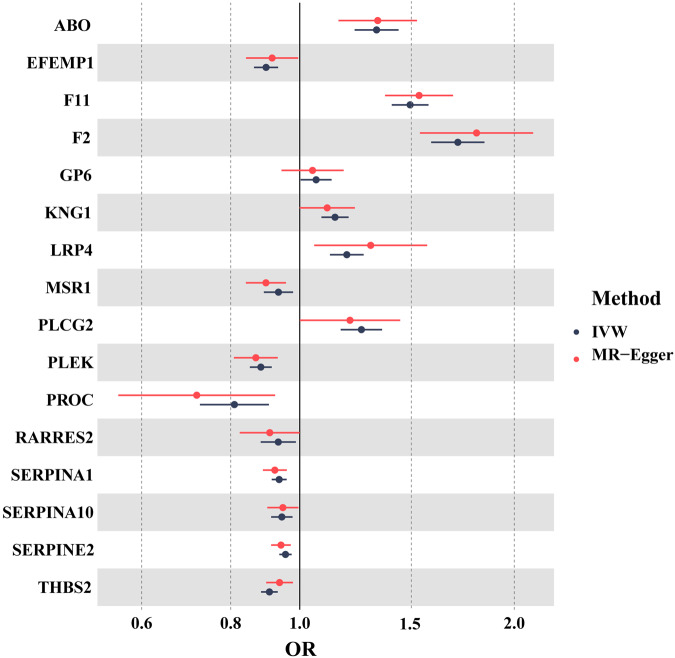
Result of Mendelian randomization (MR) between PWAS-significant proteins and the risk of VTE. Among these 20 proteins, 13 were consistent with being causal based on MR. IVW inverse variance weighted

Most of the analyzed proteins could be instrumented using several SNPs. MR estimates were mainly based on the IVW method. We further confirmed that thirteen proteins, including F11, ABO, PLCG2, LRP4, PLEK, PROC, KNG1, THBS2, SERPINA1, SERPINE2, SERPINA10, EFEMP1, and F2, have a causal relationship with VTE. Associations between lower EFEMP1, PLEK, PROC, SERPINA1, SERPINA10, SERPINE2, and THBS2 levels and higher VTE risk were identified, as well as associations between higher ABO, F2, F11, KNG1, LRP4, and PLCG2 levels and higher VTE risk (Table 1, Fig. 2, Supplementary Tables 2 and 3).

### Colocalization between VTE GWAS and pQTL in the plasma

**Fig. 3:**
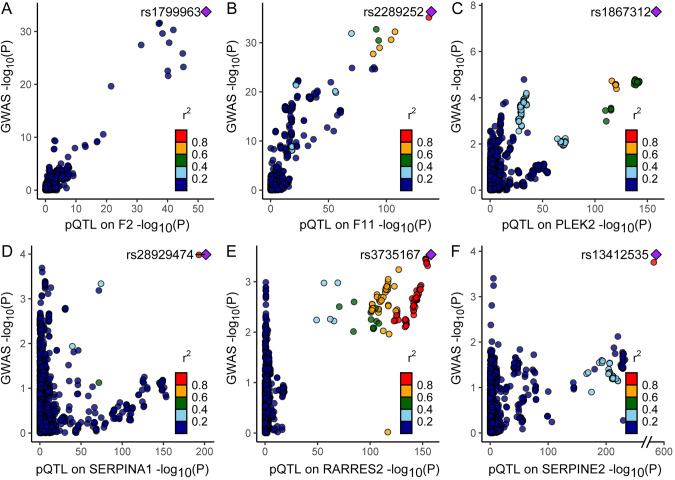
The locus-compare scatter plot for the association signals at F2, F11, PLEK, SERPINA1, RARRES2, and SERPINE2. Colocalization analyses results are shown for (**A**) F2, (**B**) F11, (**C**) PLEK, (**D**) SERPINA1, (**E**) RARRES2, and (**F**) SERPINE2. The locus-compare scatter plot compares the protein quantitative trait loci (pQTL) results and the genome-wide association study (GWAS) results, which indicates whether the GWAS top locus is also the leading SNP in the pQTL result

The colocalization analysis reported for each protein, the probability that the GWAS and pQTL share the same variant, referred to as hypothesis 4 (PP4). This analysis found that 6 of the 20 proteins provided evidence of genetic colocalization based on a PP4 > 80%. The results indicated that F2, F11, PLEK, SERPINA1, RARRES2, and SERPINE2 play important roles in VTE risk (PP4 = 100.0, 99.9, 100.0, 95.5, 80.3, and 91.2%, respectively; Table 1, Fig. 3, Supplementary Table 4, Supplementary Table 5, and Supplementary Fig. 4).

### Protein-protein interaction and pathway enrichment analysis

**Fig. 4:**
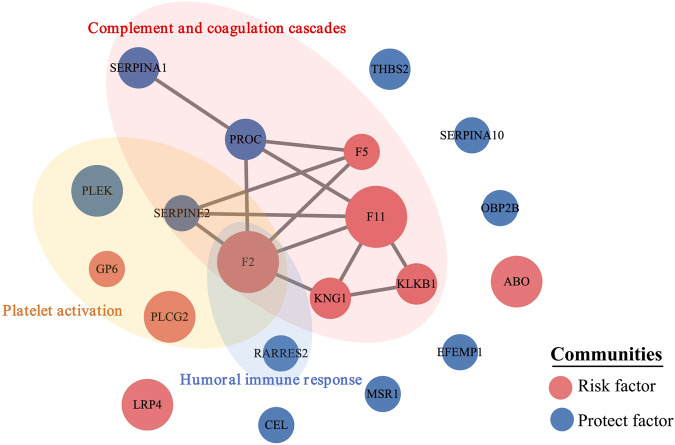
Protein-protein interaction (PPI) network and pathways among the 20 significant proteins in VTE from the PWAS. The lines represent physical PPI. The thickness of the lines is proportional to the evidence for the PPI. Enrichment of pathways was determined using a hypergenometric test with Bonforroni adjustment for multiple testing correction

We used the STRING database to investigate the connectivity among the 20 VTE-related proteins from the PWAS and found a protein community based on PPIs. A module is a set of proteins that are more connected to one another than they are to other groups of proteins. The module included F2, F5, F11, PROC, SERPINA1, SERPINE2, KLKB1, and KNG1 (Fig. 4, and Supplementary Table 6).

We also carried out pathway enrichment analysis on genes within a PPI network. We found that these 20 significant genes were involved in complement and coagulation cascades (F2, F5, F11, KLKB1, PROC, KNG1, SERPINA1, and SERPINE2), platelet activation (F2, PLCG2, PLEK, GP6, and SERPINE2), and the immune response (F2 and RARRES2; Fig. 4 and Supplementary Table 7).

### Examination of expression in VTE patients

**Fig. 5:**
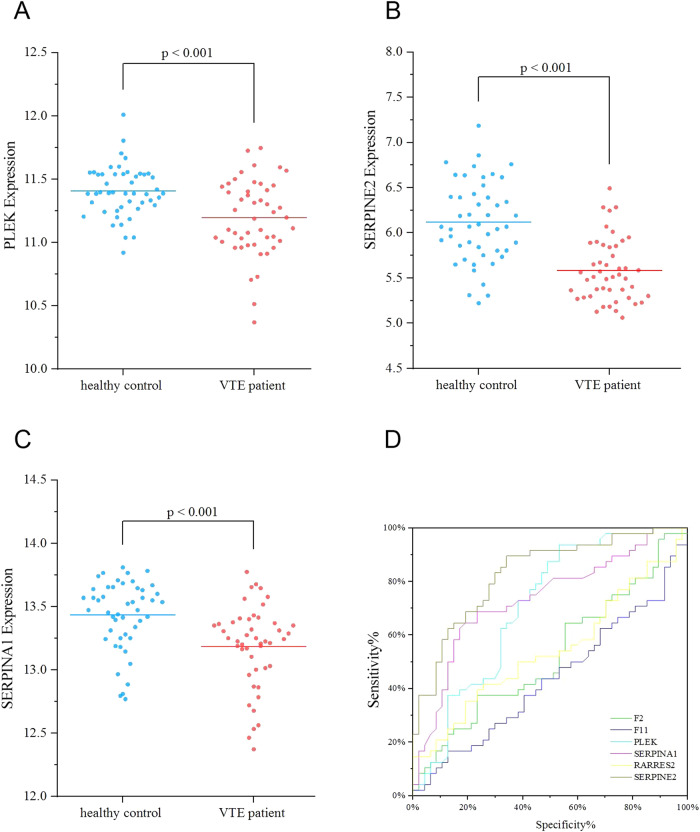
Differentially expressed of (**A**) PLEK, (**B**) SERPINE2, and (**C**) SERPINA1 in VTE patients and healthy controls. (**D**) Receiver operating characteristic (ROC) results of different proteins between the VTE and healthy controls

To further confirm the dysregulation of these proteins in VTE, we analyzed blood tissues from VTE patients and healthy control in GSE19151. Gene expression analysis showed that three out of six VTE-related proteins, PLEK, SERPINA1, and SERPINE2, had significantly lower expression levels in the VTE group compared with healthy control group (*p* < 0.001) (Fig. 5A–C), while the expression level of RARRES2, F2, and F11 was no significant between the VTE group and healthy control group (*p* > 0.05).

### Diagnosis performance of candidate plasma biomarkers

We inspected the individual diagnostic performance of PLEK, SERPINA1, SERPINE2, RARRES2, F2 and F11 between the VTE patients and healthy controls. In Fig. 5D, the area under the ROC curve (AUC) of SERPINE2, SERPINA1, and PLEK were 0.83, 0.74, and 0.71, respectively those of other proteins were in the range of 0.52–0.56 in the VTE diagnosis. SERPINE2, SERPINA1, and PLEK showed good performance for the VTE diagnosis.

### Significance of the protein findings

Our research identified the lowest *P* values for the SNPs within 1 Mb of each of these 20 genes using the summary statistics from the UKB. We determined that ten genes (*PROC*, *KNG1*, *THBS2*, *SERPINA1*, *RARRES2*, *GP6*, *SERPINE2*, *SERPINA10*, *EFEMP1*, and *MSR1*) had SNPs with *P* values ranging from 4.05 × 10^−4^ to 5.83 × 10^−7^ (Supplementary Table 8), implying that these genes could not be significant in GWAS of VTE. These findings are consistent with observations from other PWAS studies that found the novel genes from regions below genome-wide significant *P* values [32–34].

## Discussion

In this research, we performed a PWAS analysis of VTE and found 20 proteins that may be involved in VTE pathogenesis, including several known VTE-associated genes, such as *F2*, *F5*, *F11*, *PROC*, and *ABO* gene-encoded proteins. The MR analysis proved that 13 of the 20 PWAS-selected genes were linked with VTE via cis-regulated protein abundance. COLOC analysis found 6 representative SNPs (*F2* rs1799963, *F11* rs2289252, *PLEK* rs1867312, *SERPINA1* rs28929474, *SERPINE2* rs3735167, and *RARRES2* rs13412535) that are responsible for both VTE risk and protein level modulation. In the following PPI and functional analysis, relevant proteins were enriched in pathways complement and coagulation cascades (F2, F5, F11, KLKB1, PROC, KNG1, SERPINA1, and SERPINE2), platelet activation (F2, PLCG2, PLEK, GP6, and SERPINE2), and immune response (F2 and RARRES2).

A cross-ancestry investigation of VTE genomic predictors conducted by Thibord et al revealed some protein encoding genes related to VTE risk, some of which are supported by our study, including findings on F2, F11, GP6, PLEK, PROS1, and MSR1 [10]. Furthermore, we performed COLOC analysis to assess the probability of some SNPs might be responsible for VTE by changing particular protein expression, which makes outcomes more robust to some extent.

In our analysis, we identified some classical VTE-proteins, such as F2 and F11 [35]. F2 is also known as prothrombin, which is an indispensable key factor in both endogenous and exogenous coagulation pathways and possesses procoagulant, anticoagulant, and antifibrinolytic activities. The G20210A variant (rs1799963) of the *F2* gene is common in the Caucasian population. It can promote an increase in prothrombin expression and activity and the synthesis of prothrombin, and thus increase the risk of thrombosis [36]. F11 affects VTE as a part of the extrinsic coagulation pathway. Interestingly, as another well-known gene, the F5 was only significant in our PWAS study. Maybe the result of the MR study is caused by weak IVs for F5.

Furthermore, we also discovered four novel proteins in VTE including SERPINA1, SERPINE2, and PLEK, which are the most meaningful findings in our research. Some existing evidence has implied their potential relationship with VTE. Genetic variations of the *SERPINA1* gene rs28929474 are associated with the risk of VTE [37, 38]. The genetic variant of the *SERPINA1* rs2749527 has also been reported to influence plasma cortisol levels [39, 40] and an MR analysis discovered that higher plasma cortisol levels were associated with a reduced risk of VTE [41]. SERPINE2 has been discovered to function in many vascular disorders, such as atherosclerosis and restenosis [42]. It differs from conventional thrombosis-related factors as it exists on the surface of most cells but is barely expressed in plasma. SERPINE2 can play an inhibitory role in the coagulation system as well as in the fibrinolytic system [43, 44]. As a result, it is a significant regulator of hemostasis, thrombosis, and vascular disorders, although its function in VTE has not been clarified [45]. The *PLEK* gene is an active factor in VTE, and it was confirmed in our research as well. A large GWAS meta-analysis found that *PLEK* rs1867312 was an independent genetic risk signal for VTE [8]. Additionally, the transcribed protein of *PLEK*, pleckstrin, is found in platelets and is involved with platelet biology [46–48].

Our study has several advantages. First, PWAS of VTE was conducted using the human proteome and summary statistics from the UKB, a large population-based prospective study with deep genetic and phenotypic data. Second, we performed the PWAS and verified the risk proteins with independent MR validation analysis. Third, based on Bayesian colocalization used to estimate the probability that two associated signals were observed at a particular site with a common causal variant, we confirmed the pathogenetic proteins (F2, F11, PLEK, SERPINA1, RARRES2, and SERPINE2) of VTE. Finally, PWAS could detect proteins which could be ignored by GWAS.

Our research also has some limitations. First, the impact of ethnic differences on the genetic architecture of VTE cannot be ignored. The current proteomic information was from the European population; thus, the applicability of this study to other populations needs to be discussed. Second, this study only used GWAS and pQTL for PWAS analysis and barely obtained results at the level of protein, which might have minimized the robustness of our conclusions. We could further screen and explore through TWAS to achieve a more complete understanding of the molecular mechanism involved in VTE. Finally, based on our PWAS conclusion, we only performed MR, COLOC, PPI, and functional analysis to verify their rationality, and more diverse and multi-level exploration should be carried out for verification.

## Conclusion

This research conducted a PWAS analysis to explore the proteomic pathogenesis of VTE. Several proteins including SERPINA1, SERPINE2, and PLEK, were considered to play a role in the development of VTE and are of great value for further research to find new diagnostic and therapeutic targets for VTE.

### Supplementary information

Supplementary information

## Data Availability

For access to GWAS summary statistics from the UK Biobank (UKB, access code 56719) in this manuscript see: www.ukbiobank.ac.uk/. For access to the results of the pQTL analysis and protein weights described in this manuscript see: 10.1038/s41588-022-01051-w. All codes analysed in this study can be obtained by a reasonable request to corresponding authors.

## References

[1] Di Nisio M, van Es N, Büller HR (2016). Deep vein thrombosis and pulmonary embolism. Lancet..

[2] Wendelboe AM, Raskob GE (2016). Global burden of thrombosis: epidemiologic aspects. Circ Res.

[3] Schulman S, Ageno W, Konstantinides SV (2017). Venous thromboembolism: past, present and future. Thromb Haemost.

[4] Zhang Z, Lei J, Shao X, Dong F, Wang J, Wang D (2019). Trends in hospitalization and in-hospital mortality From VTE, 2007 to 2016, in China. Chest..

[5] Souto JC, Almasy L, Borrell M, Blanco-Vaca F, Mateo J, Soria JM (2000). Genetic susceptibility to thrombosis and its relationship to physiological risk factors: the GAIT study. Genetic analysis of idiopathic thrombophilia. Am J Hum Genet.

[6] Morange PE, Suchon P, Trégouët DA (2015). Genetics of venous thrombosis: update in 2015. Thromb Haemost.

[7] Hirschhorn JN, Daly MJ (2005). Genome-wide association studies for common diseases and complex traits. Nat Rev Genet.

[8] Lindstrom S, Wang L, Smith EN, Gordon W, van Hylckama Vlieg A, de Andrade M (2019). Genomic and transcriptomic association studies identify 16 novel susceptibility loci for venous thromboembolism. Blood..

[9] Klarin D, Busenkell E, Judy R, Lynch J, Levin M, Haessler J (2019). Genome-wide association analysis of venous thromboembolism identifies new risk loci and genetic overlap with arterial vascular disease. Nat Genet.

[10] Thibord F, Klarin D, Brody JA, Chen MH, Levin MG, Chasman DI (2022). Cross-ancestry investigation of venous thromboembolism genomic predictors. Circulation.

[11] Zhang Z, Li H, Weng H, Zhou G, Chen H, Yang G (2023). Genome-wide association analyses identified novel susceptibility loci for pulmonary embolism among Han Chinese population. BMC Med.

[12] Croles FN, Nasserinejad K, Duvekot JJ, Kruip MJ, Meijer K, Leebeek FW (2017). Pregnancy, thrombophilia, and the risk of a first venous thrombosis: systematic review and bayesian meta-analysis. BMJ..

[13] Wingo TS, Liu Y, Gerasimov ES, Gockley J, Logsdon BA, Duong DM (2021). Brain proteome-wide association study implicates novel proteins in depression pathogenesis. Nat Neurosci.

[14] Zhang C, Qin F, Li X, Du X, Li T (2022). Identification of novel proteins for lacunar stroke by integrating genome-wide association data and human brain proteomes. BMC Med.

[15] Wingo AP, Liu Y, Gerasimov ES, Gockley J, Logsdon BA, Duong DM (2021). Integrating human brain proteomes with genome-wide association data implicates new proteins in Alzheimer’s disease pathogenesis. Nat Genet.

[16] Wingo TS, Gerasimov ES, Liu Y, Duong DM, Vattathil SM, Lori A (2022). Integrating human brain proteomes with genome-wide association data implicates novel proteins in post-traumatic stress disorder. Mol Psychiatry.

[17] Ou YN, Yang YX, Deng YT, Zhang C, Hu H, Wu BS (2021). Identification of novel drug targets for Alzheimer’s disease by integrating genetics and proteomes from brain and blood. Mol Psychiatry.

[18] Liu J, Li X, Luo XJ (2021). Proteome-wide association study provides insights into the genetic component of protein abundance in psychiatric disorders. Biol Psychiatry.

[19] Zhang J, Dutta D, Köttgen A, Tin A, Schlosser P, Grams ME (2022). Plasma proteome analyses in individuals of European and African ancestry identify cis-pQTLs and models for proteome-wide association studies. Nat Genet.

[20] Bycroft C, Freeman C, Petkova D, Band G, Elliott LT, Sharp K (2018). The UK Biobank resource with deep phenotyping and genomic data. Nature..

[21] Purcell S, Neale B, Todd-Brown K, Thomas L, Ferreira MA, Bender D (2007). PLINK: a tool set for whole-genome association and population-based linkage analyses. Am J Hum Genet.

[22] Gusev A, Ko A, Shi H, Bhatia G, Chung W, Penninx BW (2016). Integrative approaches for large-scale transcriptome-wide association studies. Nat Genet.

[23] Zhu Z, Zhang F, Hu H, Bakshi A, Robinson MR, Powell JE (2016). Integration of summary data from GWAS and eQTL studies predicts complex trait gene targets. Nat Genet.

[24] Porcu E, Rüeger S, Lepik K, Santoni FA, Reymond A, Kutalik Z (2019). Mendelian randomization integrating GWAS and eQTL data reveals genetic determinants of complex and clinical traits. Nat Commun.

[25] Burgess S, Bowden J, Fall T, Ingelsson E, Thompson SG (2017). Sensitivity analyses for robust causal inference from mendelian randomization analyses with multiple genetic variants. Epidemiology.

[26] Bowden J, Davey Smith G, Burgess S (2015). Mendelian randomization with invalid instruments: effect estimation and bias detection through Egger regression. Int J Epidemiol.

[27] Giambartolomei C, Vukcevic D, Schadt EE, Franke L, Hingorani AD, Wallace C (2014). Bayesian test for colocalisation between pairs of genetic association studies using summary statistics. PLoS Genet.

[28] Liu B, Gloudemans MJ, Rao AS, Ingelsson E, Montgomery SB (2019). Abundant associations with gene expression complicate GWAS follow-up. Nat Genet.

[29] Szklarczyk D, Gable AL, Lyon D, Junge A, Wyder S, Huerta-Cepas J (2019). STRING v11: protein-protein association networks with increased coverage, supporting functional discovery in genome-wide experimental datasets. Nucleic Acids Res.

[30] Wu T, Hu E, Xu S, Chen M, Guo P, Dai Z (2021). clusterProfiler 4.0: a universal enrichment tool for interpreting omics data. Innov (Camb).

[31] Lewis DA, Stashenko GJ, Akay OM, Price LI, Owzar K, Ginsburg GS (2011). Whole blood gene expression analyses in patients with single versus recurrent venous thromboembolism. Thromb Res.

[32] Wu BS, Chen SF, Huang SY, Ou YN, Deng YT, Chen SD (2022). Identifying causal genes for stroke via integrating the proteome and transcriptome from brain and blood. J Transl Med.

[33] Toikumo S, Xu H, Gelernter J, Kember RL, Kranzler HR (2022). Integrating human brain proteomic data with genome-wide association study findings identifies novel brain proteins in substance use traits. Neuropsychopharmacology..

[34] Zhang Z, Meng P, Zhang H, Jia Y, Wen Y, Zhang J (2022). Brain proteome-wide association study identifies candidate genes that regulate protein abundance associated with post-traumatic stress disorder. Genes (Basel).

[35] Yuan S, Burgess S, Laffan M, Mason AM, Dichgans M, Gill D (2021). Genetically proxied inhibition of coagulation factors and risk of cardiovascular disease: a Mendelian randomization study. J Am Heart Assoc.

[36] Zhang Y, Zhang Z, Shu S, Niu W, Xie W, Wan J (2021). The genetics of venous thromboembolism: a systematic review of thrombophilia families. J Thromb Thrombolysis.

[37] Riis J, Nordestgaard BG, Afzal S (2022). α(1) -Antitrypsin Z allele and risk of venous thromboembolism in the general population. J Thromb Haemost.

[38] Manderstedt E, Halldén C, Lind-Halldén C, Elf J, Svensson PJ, Engström G (2022). Thrombotic risk determined by rare and common SERPINA1 variants in a population-based cohort study. J Thromb Haemost.

[39] Bolton JL, Hayward C, Direk N, Lewis JG, Hammond GL, Hill LA (2014). Genome wide association identifies common variants at the SERPINA6/SERPINA1 locus influencing plasma cortisol and corticosteroid binding globulin. PLoS Genet.

[40] Yuan S, Titova OE, Zhang K, Gou W, Schillemans T, Natarajan P (2023). Plasma protein and venous thromboembolism: prospective cohort and mendelian randomisation analyses. Br J Haematol.

[41] Allara E, Lee WH, Burgess S, Larsson SC (2022). Genetically predicted cortisol levels and risk of venous thromboembolism. PLoS One.

[42] Kanse SM, Chavakis T, Al-Fakhri N, Hersemeyer K, Monard D, Preissner KT (2004). Reciprocal regulation of urokinase receptor (CD87)-mediated cell adhesion by plasminogen activator inhibitor-1 and protease nexin-1. J Cell Sci.

[43] Boulaftali Y, Adam F, Venisse L, Ollivier V, Richard B, Taieb S (2010). Anticoagulant and antithrombotic properties of platelet protease nexin-1. Blood..

[44] Boulaftali Y, Ho-Tin-Noe B, Pena A, Loyau S, Venisse L, François D (2011). Platelet protease nexin-1, a serpin that strongly influences fibrinolysis and thrombolysis. Circulation..

[45] Bouton MC, Boulaftali Y, Richard B, Arocas V, Michel JB, Jandrot-Perrus M (2012). Emerging role of serpinE2/protease nexin-1 in hemostasis and vascular biology. Blood..

[46] Coppinger JA, Cagney G, Toomey S, Kislinger T, Belton O, McRedmond JP (2004). Characterization of the proteins released from activated platelets leads to localization of novel platelet proteins in human atherosclerotic lesions. Blood..

[47] Fröbel J, Cadeddu RP, Hartwig S, Bruns I, Wilk CM, Kündgen A (2013). Platelet proteome analysis reveals integrin-dependent aggregation defects in patients with myelodysplastic syndromes. Mol Cell Proteom.

[48] Schmidt GJ, Reumiller CM, Ercan H, Resch U, Butt E, Heber S (2019). Comparative proteomics reveals unexpected quantitative phosphorylation differences linked to platelet activation state. Sci Rep..

