# Supplementary material for: Proteome-wide mendelian randomization identifies causal plasma proteins in venous thromboembolism development
Source: J Hum Genet. 2023 Aug 3;68(12):805–12. doi: 10.1038/s10038-023-01186-6 (PMC10678328; doi:10.1038/s10038-023-01186-6)
Supplement: Supplementary file 1 — Supplementary information [file 10038_2023_1186_MOESM1_ESM.pdf]

Supplementary information

Supplementary Figure 1 Manhattan plots for the GWAS of VTE 1

Supplementary Figure 2 Quantile-quantile plots for the GWAS of VTE 2

Supplementary Figure 3 Quantile-quantile plots for the PWAS of VTE 3

Supplementary Figure 4 The locus-compare scatter plot for the association signals Supplementary 4-23

Supplementary Table 1 Plasma PWAS results in VTE. 24-49

Supplementary Table 2 MR results of plasma pQTL and VTE GWAS. 50

Supplementary Table 3 Instrumental variables for 20 VTE significant proteins and annotations. 51-62

Supplementary Table 4 Colocalization results of plasma pQTL and VTE GWAS. 63

Supplementary Table 5 Colocalization results of snp in plasma pQTL and VTE GWAS. 64-83

Supplementary Table 6 Protein-protein interactions identified by the STRING Database. 84

Supplementary Table 7 Enrichment pathways among the 20 potential proteins from the PWAS of VTE. 85

Supplementary Table 8 Lowest p-values for association between SNPs within 1 Mb of each of the 20 causal proteins using summary statistics from the GWAS. 86

1

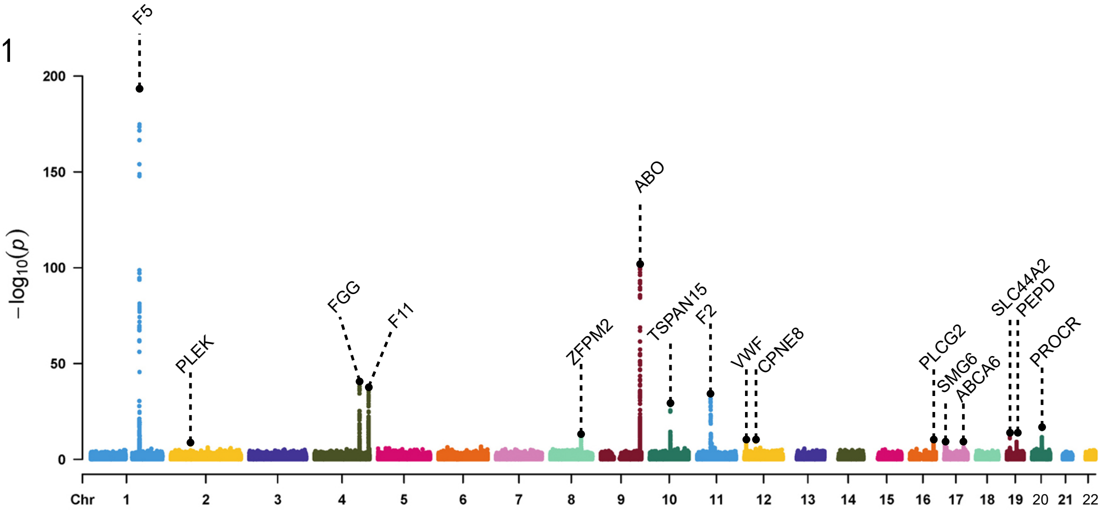

F5
PLEK
FGG
F11
ZFPM2
ABO
TSPAN15
F2
VWF
CPNE8
PLCG2
SMG6
ABCA6
SLC44A2
PEPD
PROCR
200
150
100
50
0
$-\log_{10}(p)$
Chr
1
2
3
4
5
6
7
8
9
10
11
12
13
14
15
16
17
18
19
20
21
22

2

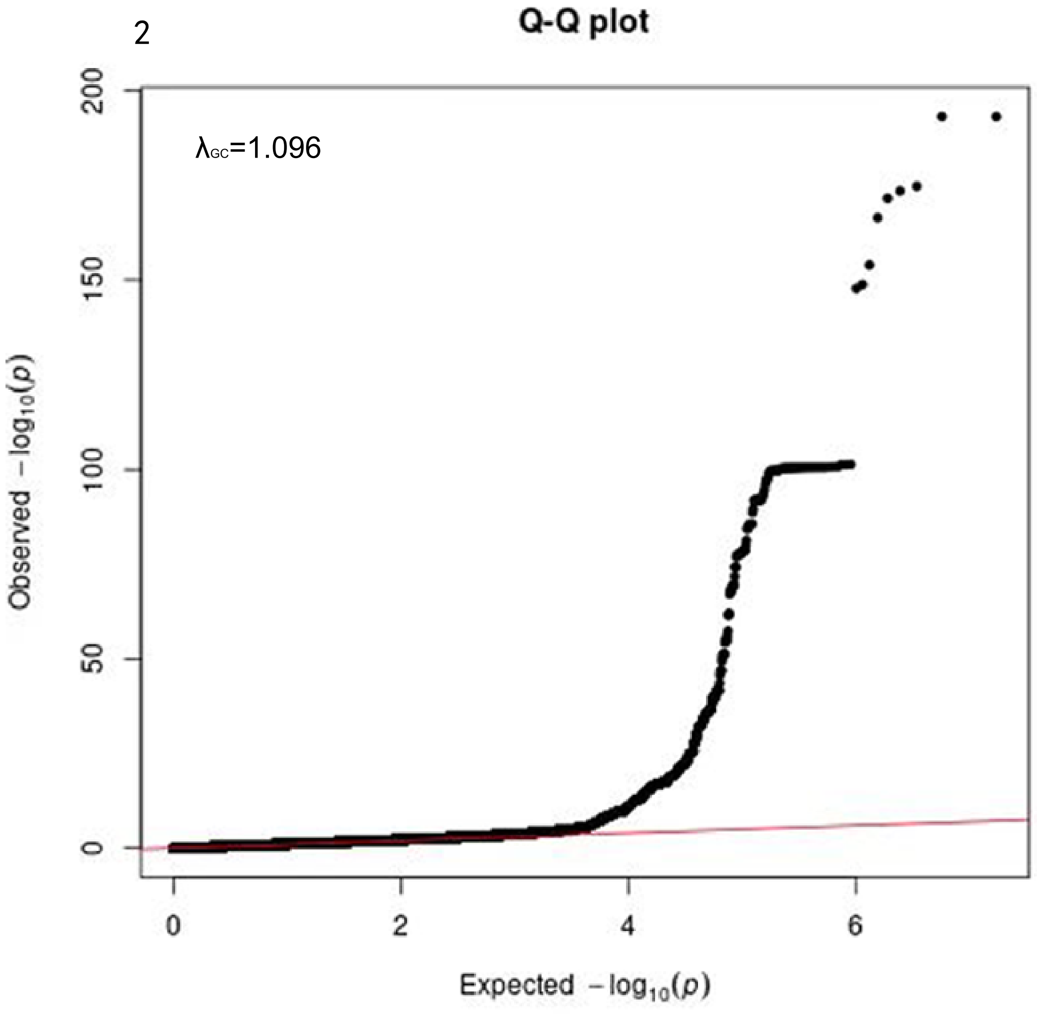
Q-Q plot
λGC=1.096
200
150
100
50
0
0
2
4
6
Observed −log10(p)
Expected −log10(p)

3

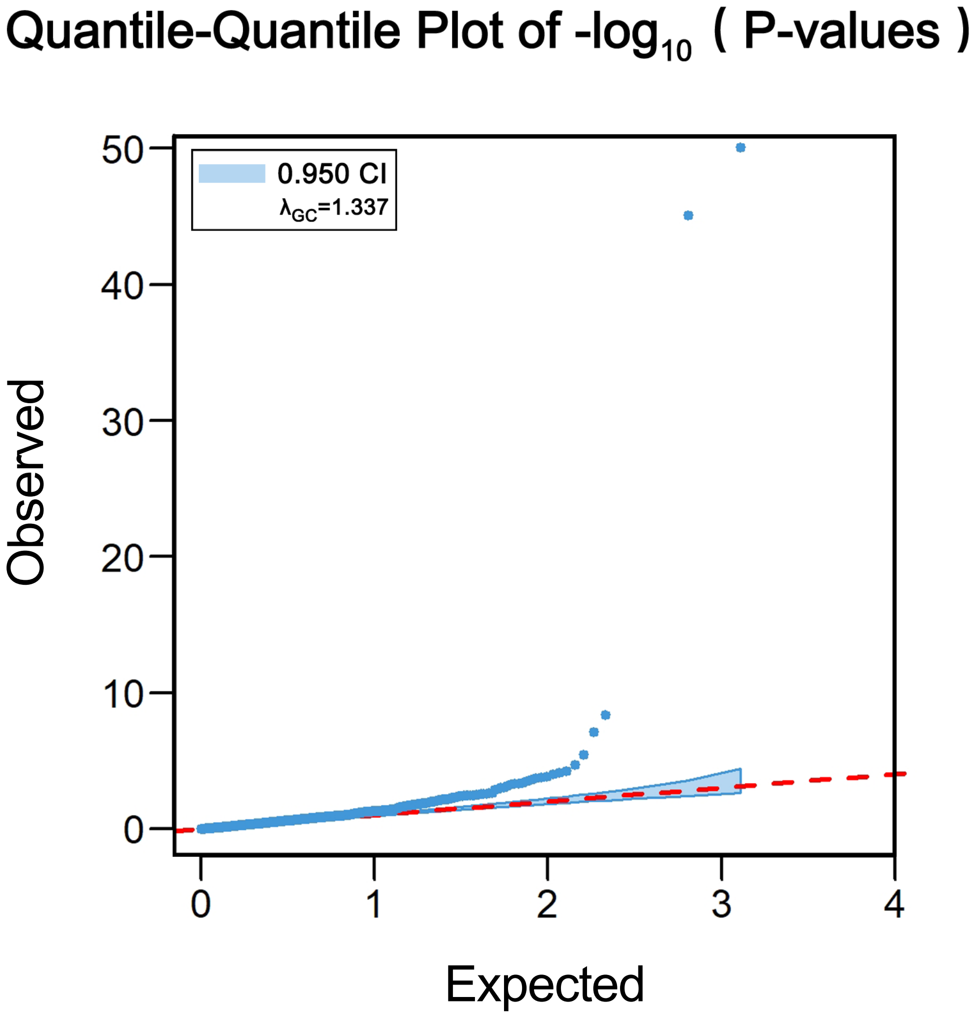
Quantile-Quantile Plot of -log10 (P-values)
0.950 CI
λGC=1.337
Observed
Expected
0
10
20
30
40
50
0
1
2
3
4

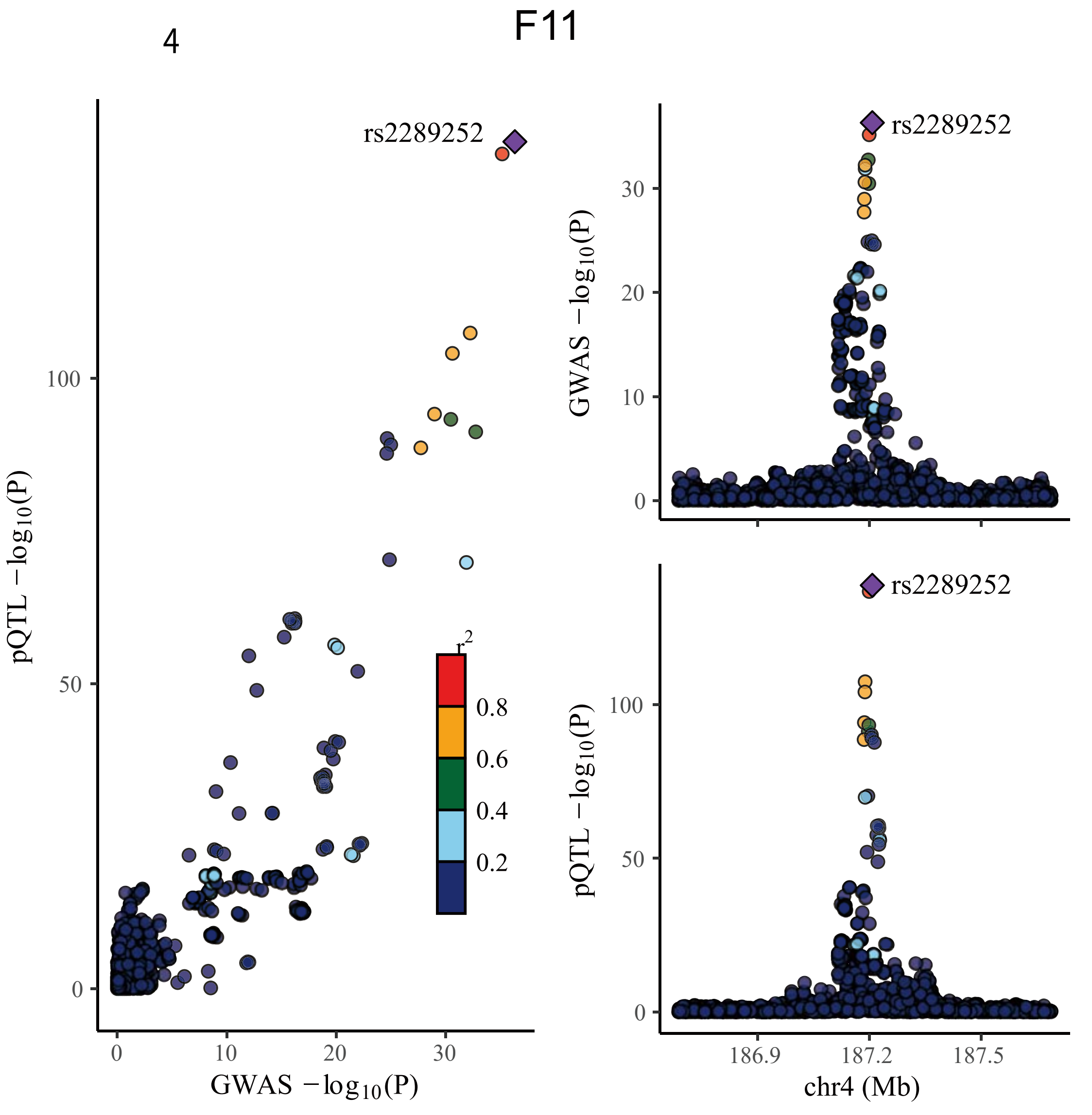
F11
4
rs2289252
pQTL −log10(P)
GWAS −log10(P)
r2
0.8
0.6
0.4
0.2
chr4 (Mb)
186.9
187.2
187.5

5

F2

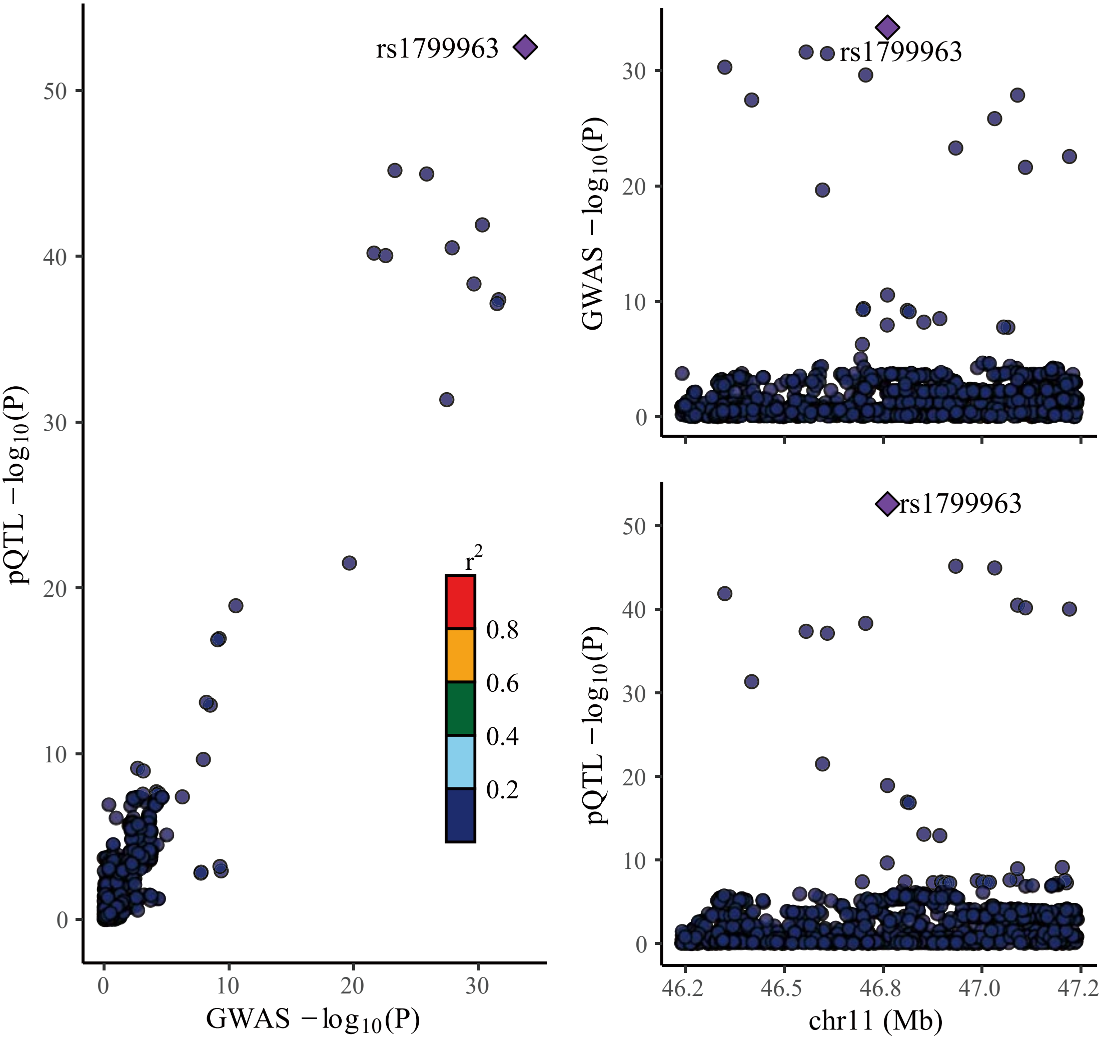

rs1799963
pQTL −log10(P)
GWAS −log10(P)
r2
0.8
0.6
0.4
0.2
GWAS −log10(P)
pQTL −log10(P)
chr11 (Mb)
46.2
46.5
46.8
47.0
47.2

6

THBS2

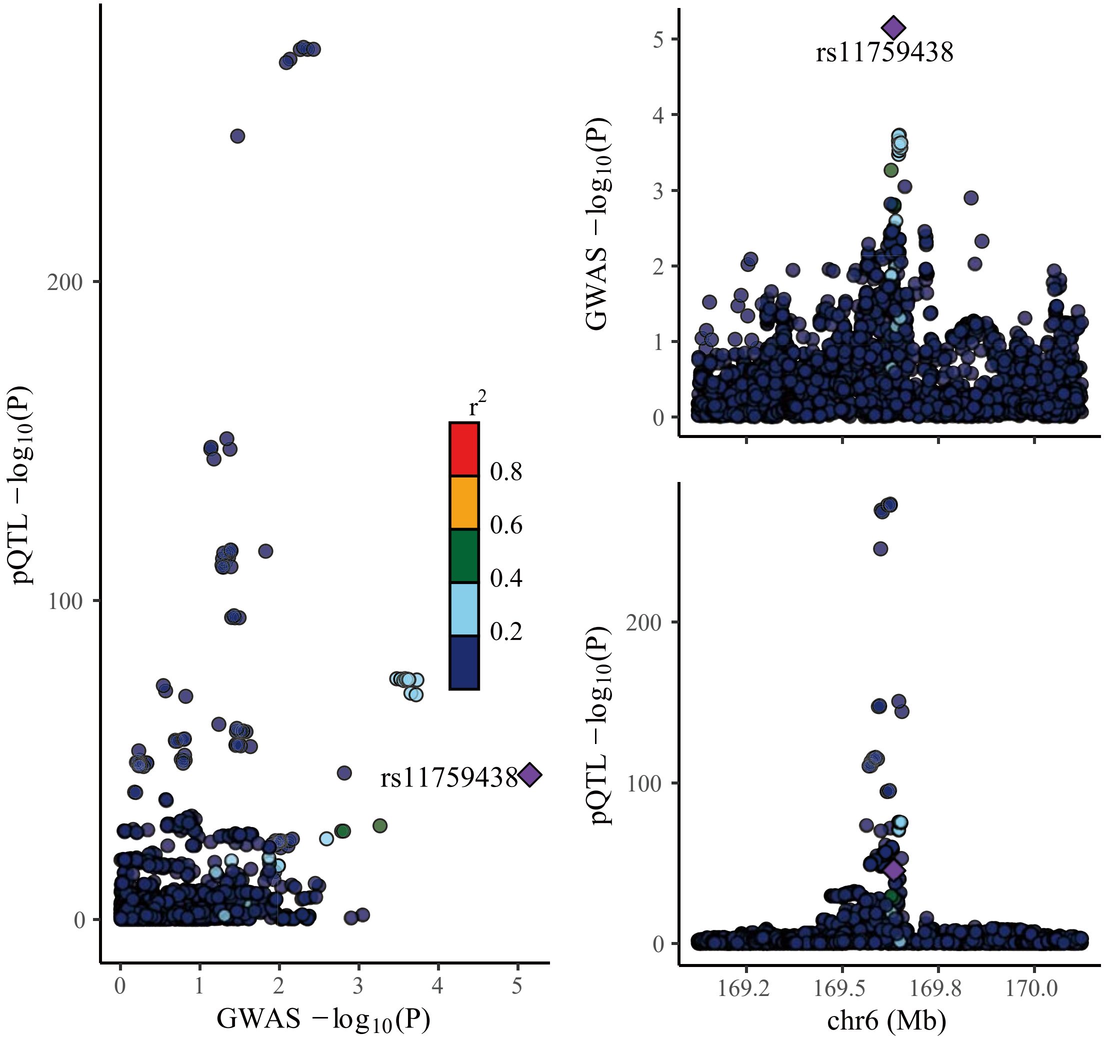
pQTL −log10(P)
GWAS −log10(P)
r2
0.8
0.6
0.4
0.2
rs11759438
GWAS −log10(P)
rs11759438
pQTL −log10(P)
chr6 (Mb)
169.2
169.5
169.8
170.0

7

PLCG2

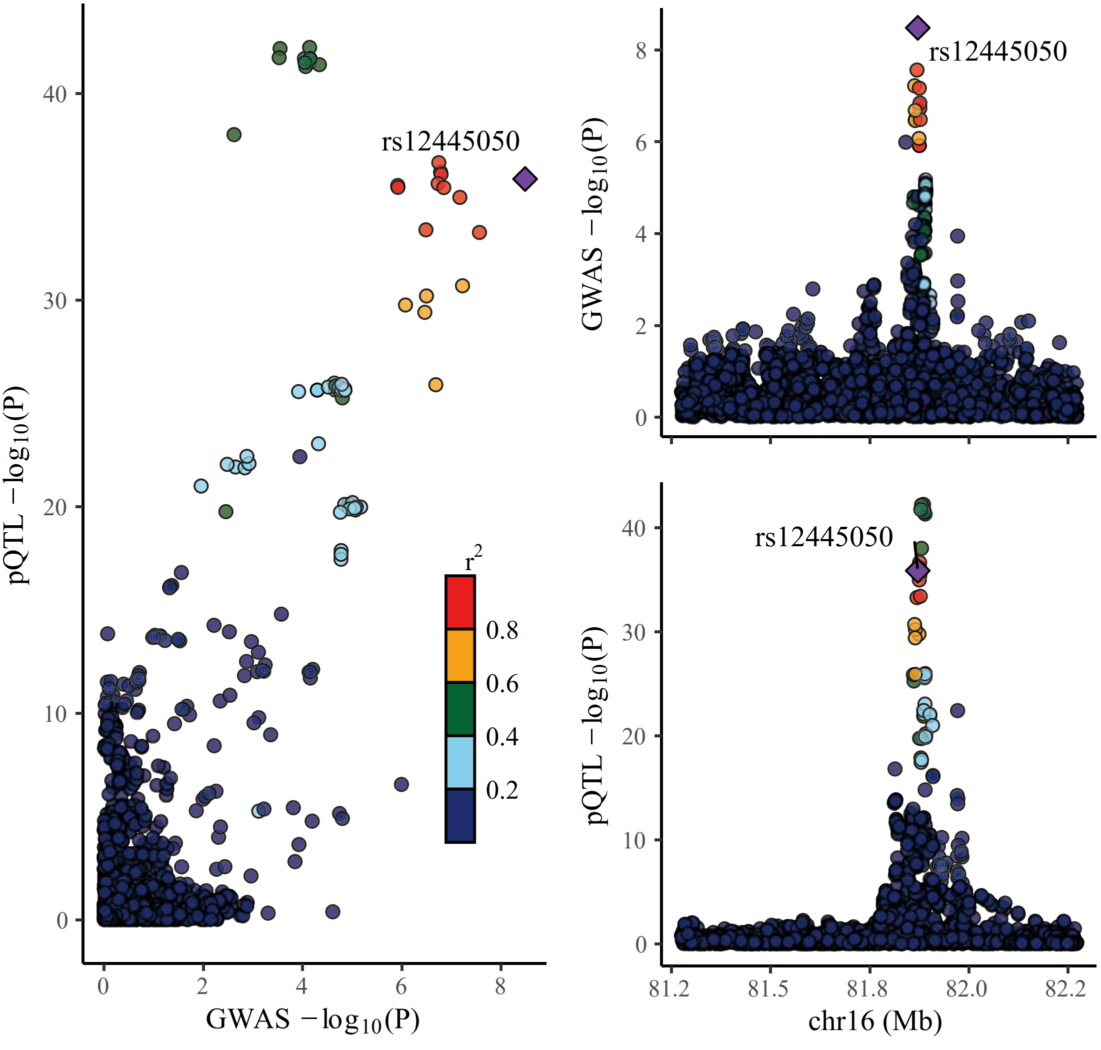

rs12445050
pQTL −log10(P)
GWAS −log10(P)
r²
0.8
0.6
0.4
0.2
40
30
20
10
0
0
2
4
6
8
GWAS −log10(P)
pQTL −log10(P)
chr16 (Mb)
81.2
81.5
81.8
82.0
82.2

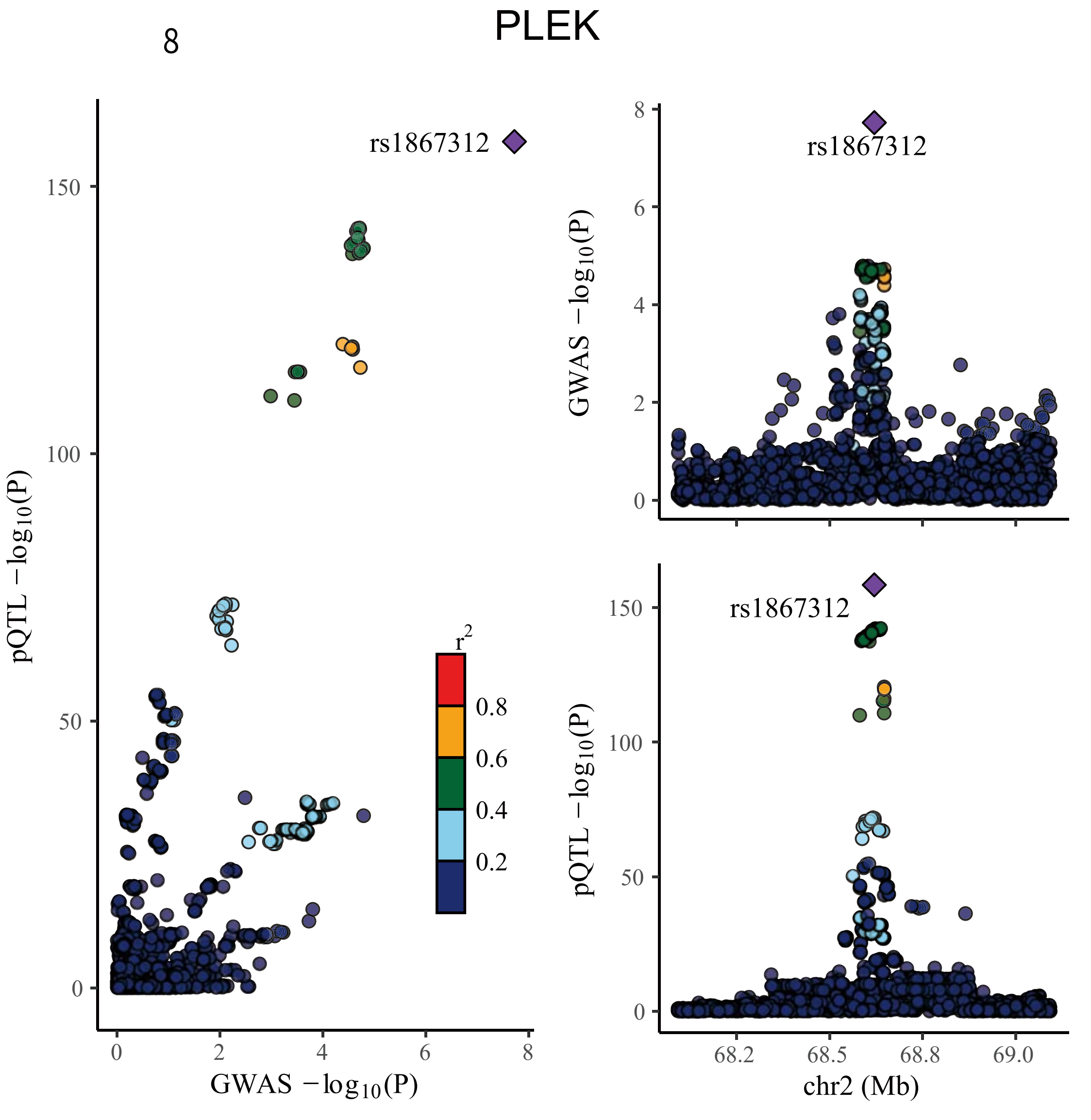
PLEK
rs1867312
pQTL −log10(P)
GWAS −log10(P)
r2
0.8
0.6
0.4
0.2
chr2 (Mb)
68.2
68.5
68.8
69.0

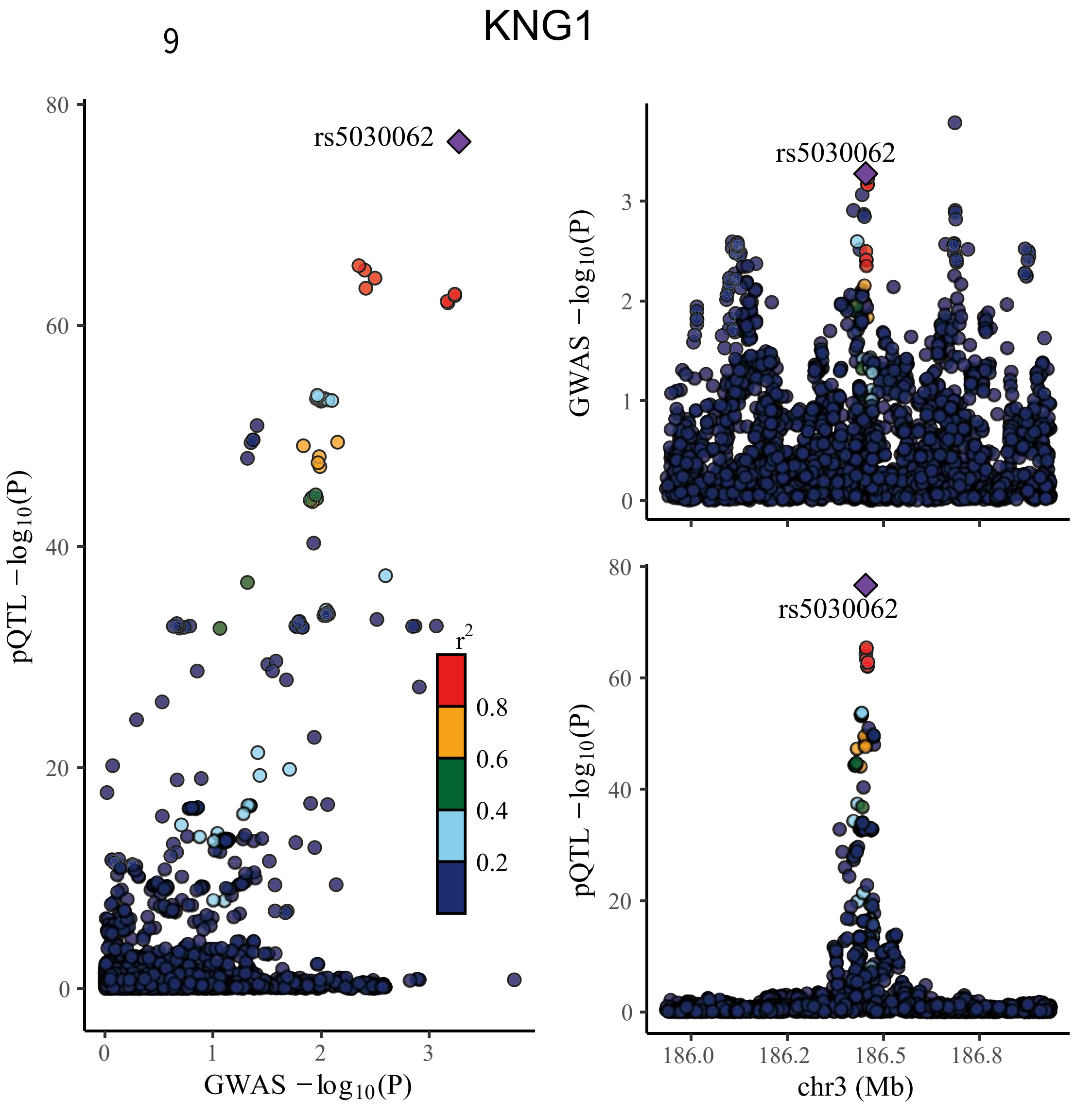

KNG1
9
rs5030062
pQTL −log10(P)
GWAS −log10(P)
r2
0.8
0.6
0.4
0.2
GWAS −log10(P)
pQTL −log10(P)
chr3 (Mb)
186.0
186.2
186.5
186.8

10

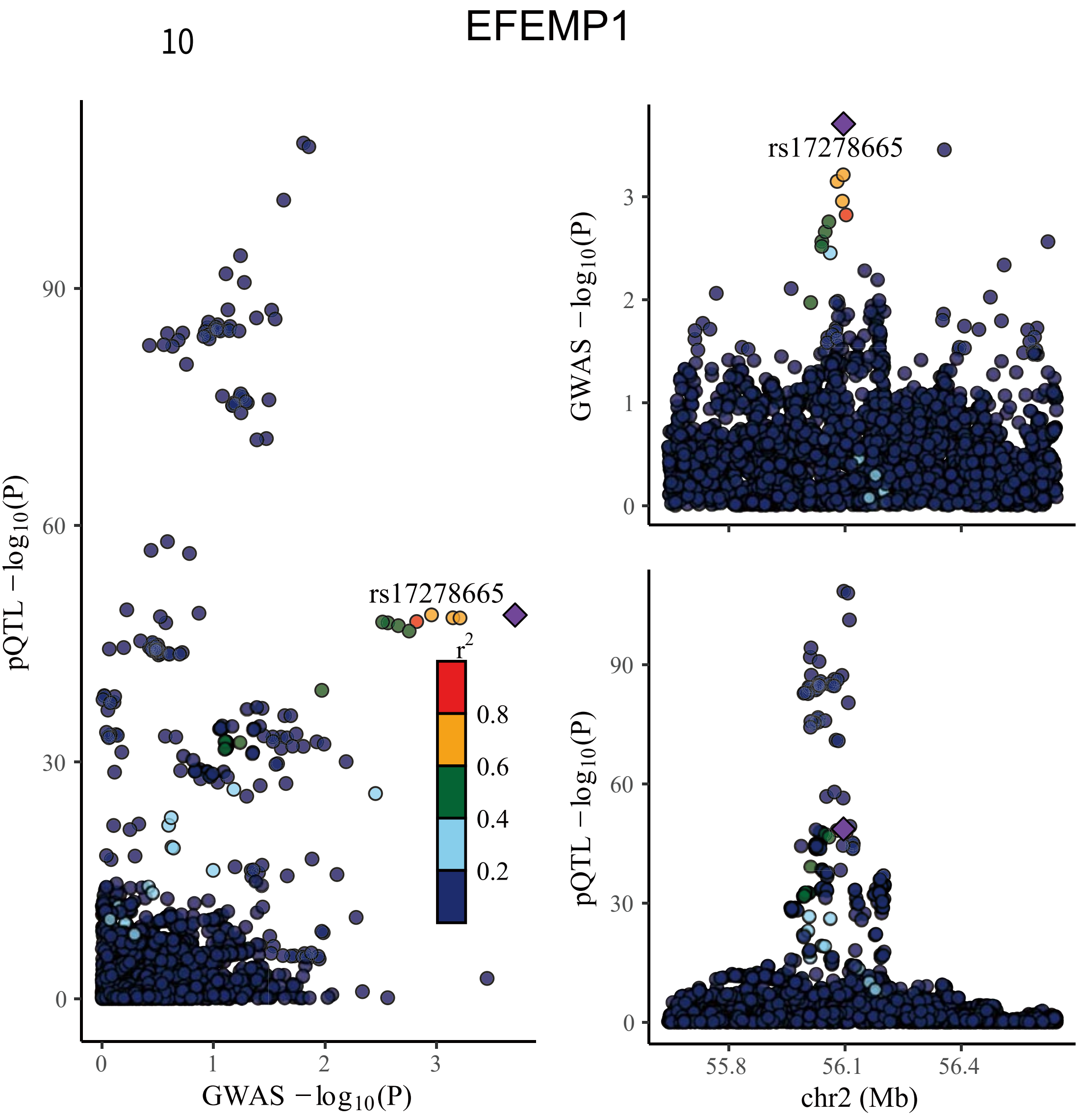

EFEMP1
rs17278665
pQTL −log10(P)
GWAS −log10(P)
r2
0.8
0.6
0.4
0.2
90
60
30
0
1
2
3
GWAS −log10(P)
pQTL −log10(P)
chr2 (Mb)
55.8
56.1
56.4

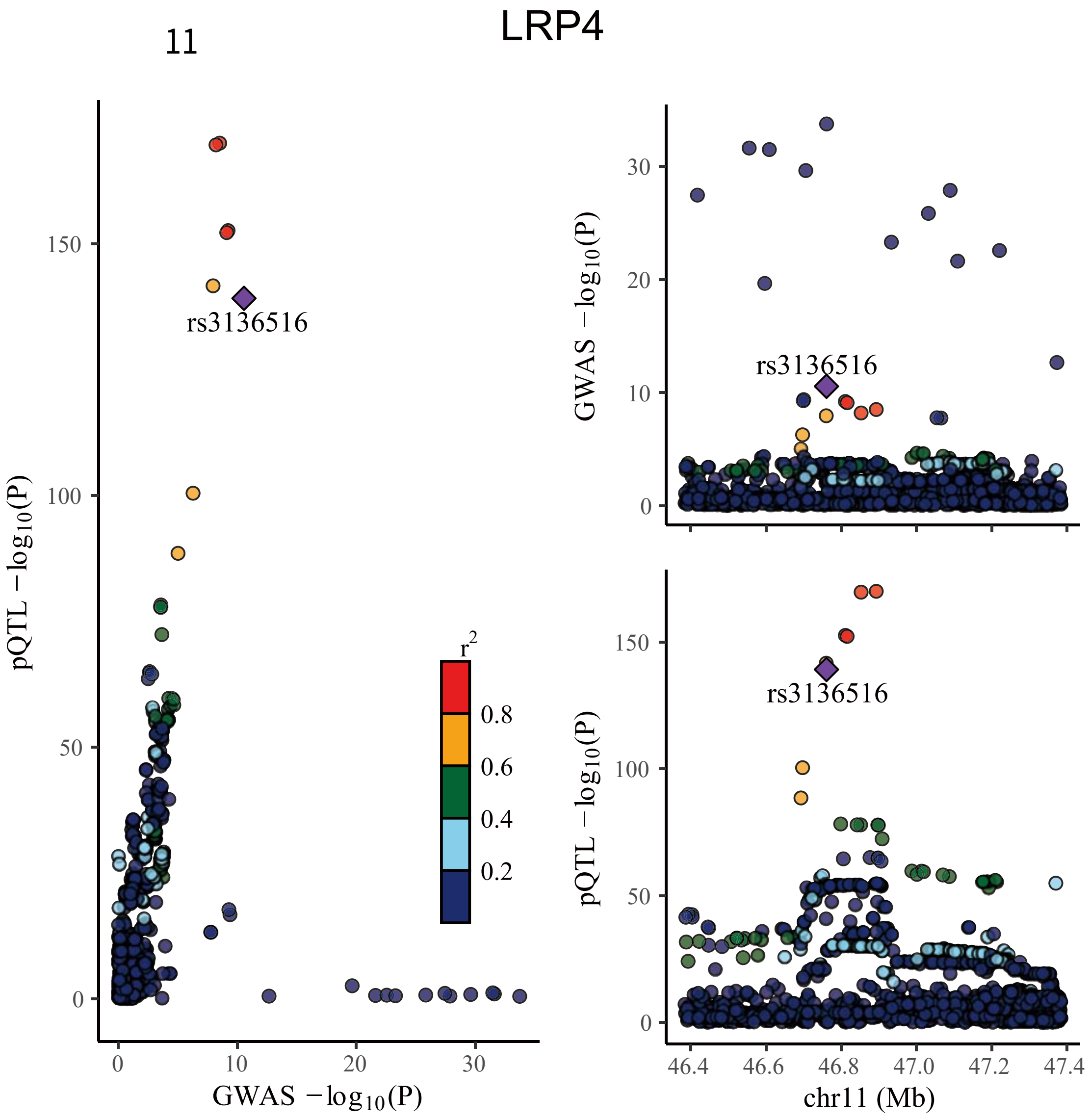

11
LRP4
rs3136516
pQTL −log10(P)
GWAS −log10(P)
r2
0.8
0.6
0.4
0.2
rs3136516
rs3136516
chr11 (Mb)

SERPINE2

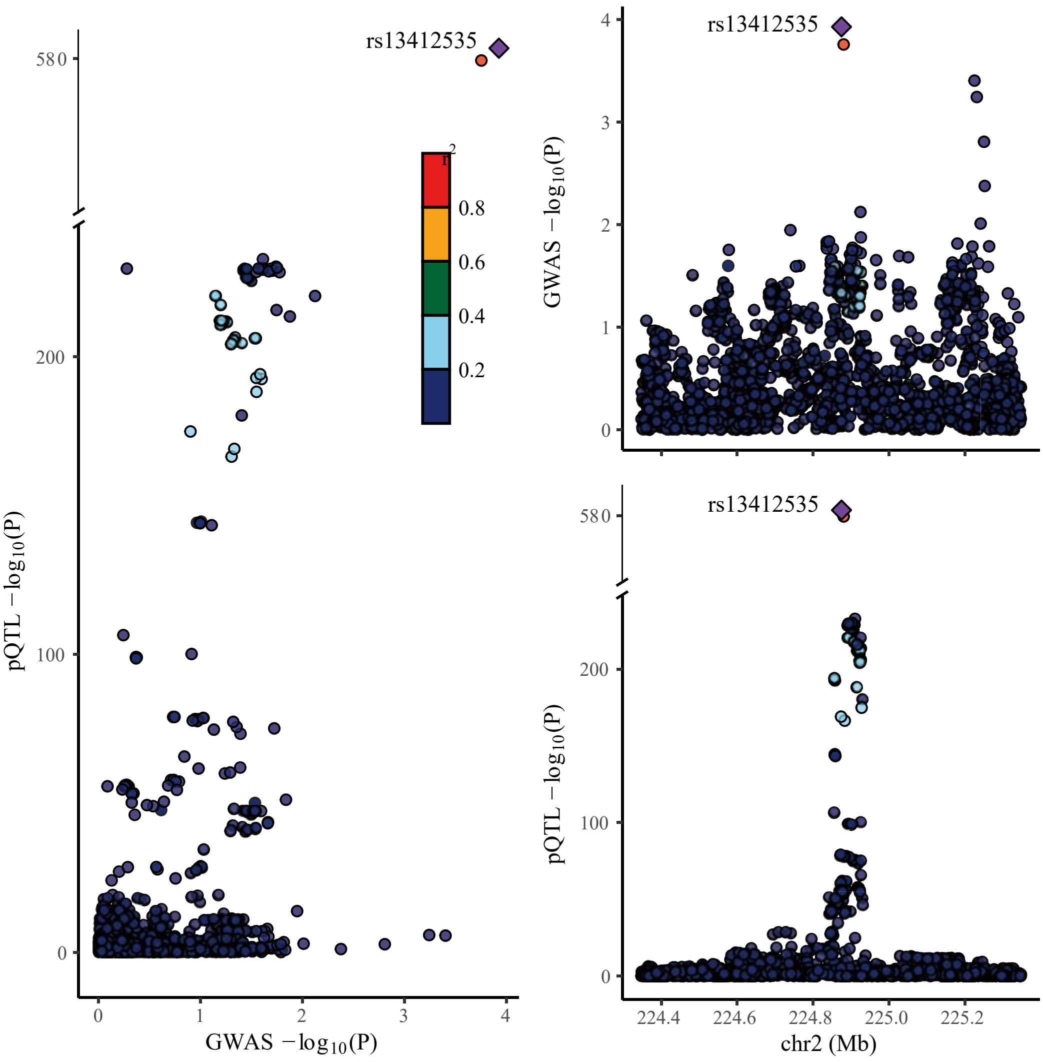

rs13412535
580
200
100
0
pQTL −log10(P)
0
1
2
3
4
GWAS −log10(P)
r2
0.8
0.6
0.4
0.2
rs13412535
4
3
2
1
0
GWAS −log10(P)
rs13412535
580
200
100
0
pQTL −log10(P)
224.4
224.6
224.8
225.0
225.2
chr2 (Mb)

ABO

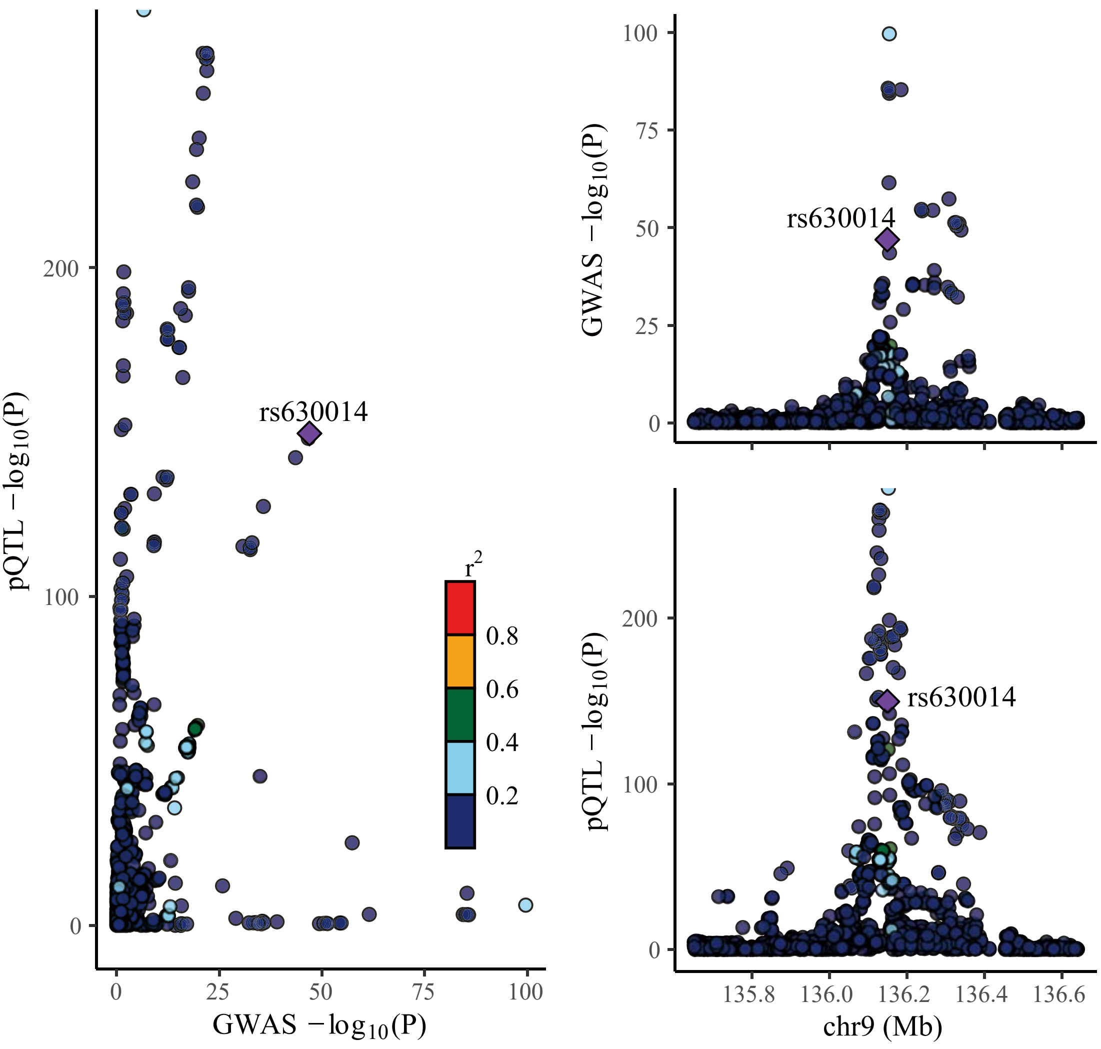

14

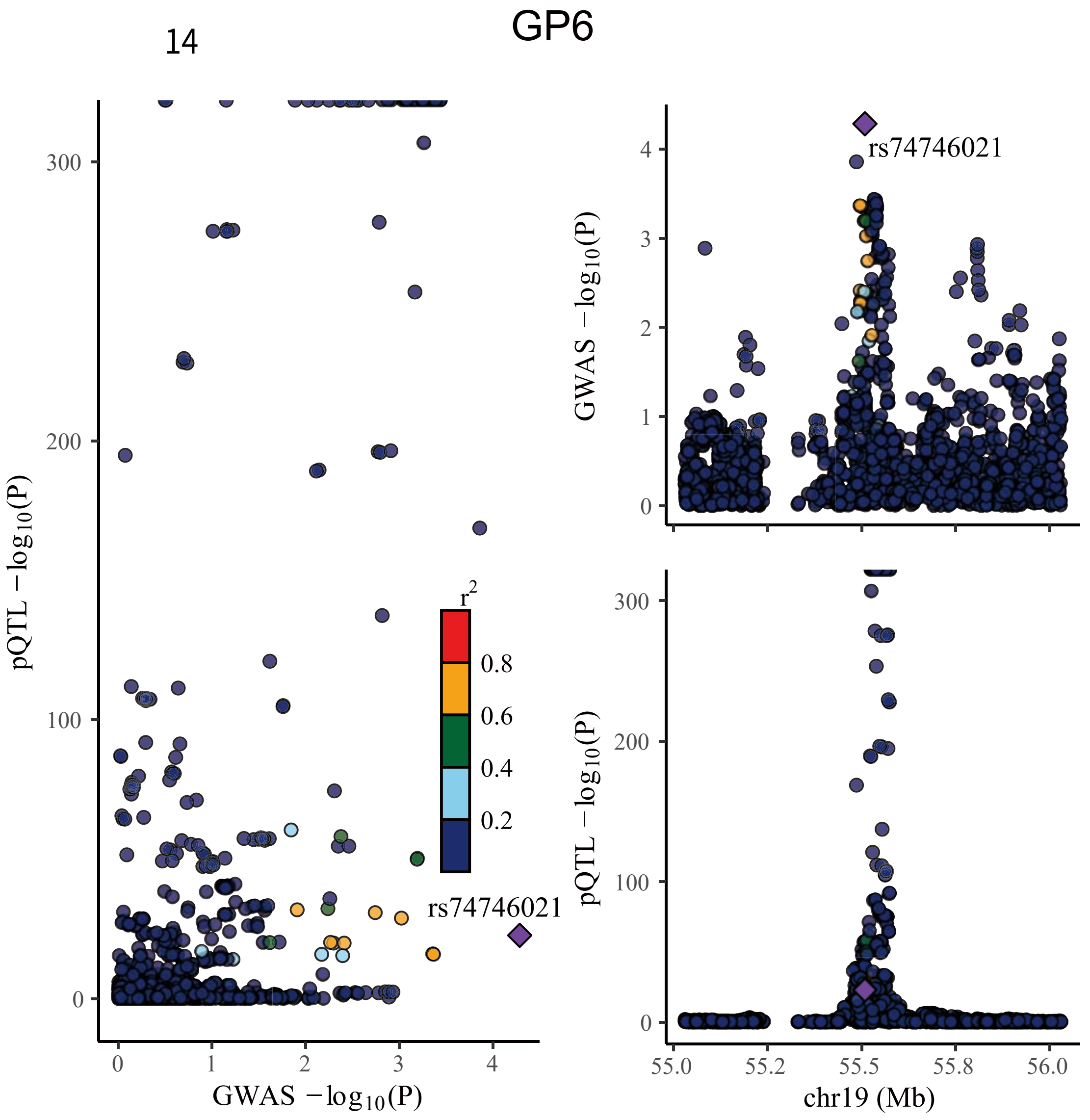
GP6
pQTL −log10(P)
GWAS −log10(P)
r2
0.8
0.6
0.4
0.2
rs74746021
chr19 (Mb)
55.0
55.2
55.5
55.8
56.0

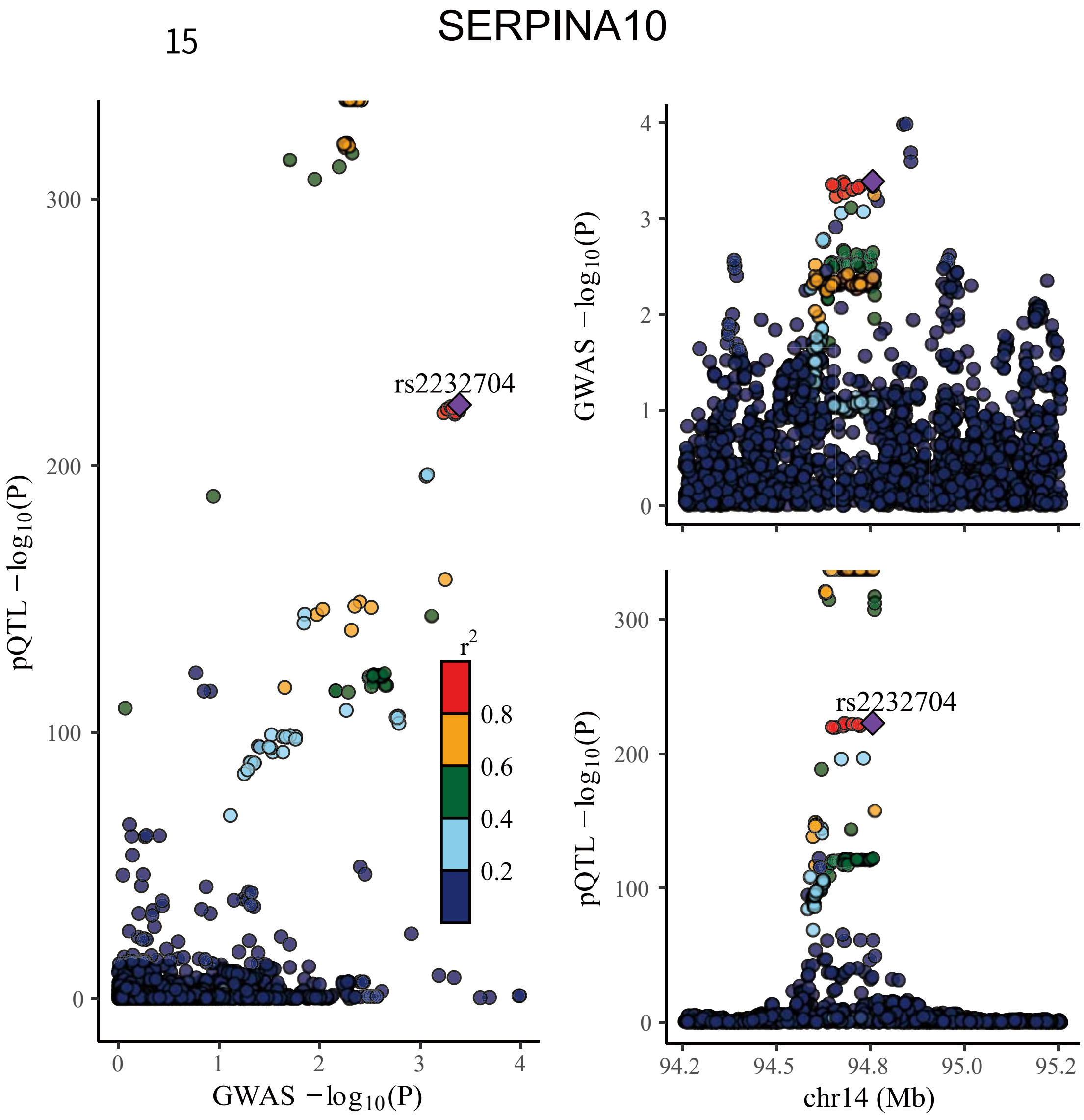
SERPINA10
rs2232704
pQTL $-\log_{10}(P)$
GWAS $-\log_{10}(P)$
r^2
0.8
0.6
0.4
0.2
rs2232704
pQTL $-\log_{10}(P)$
chr14 (Mb)
94.2
94.5
94.8
95.0
95.2

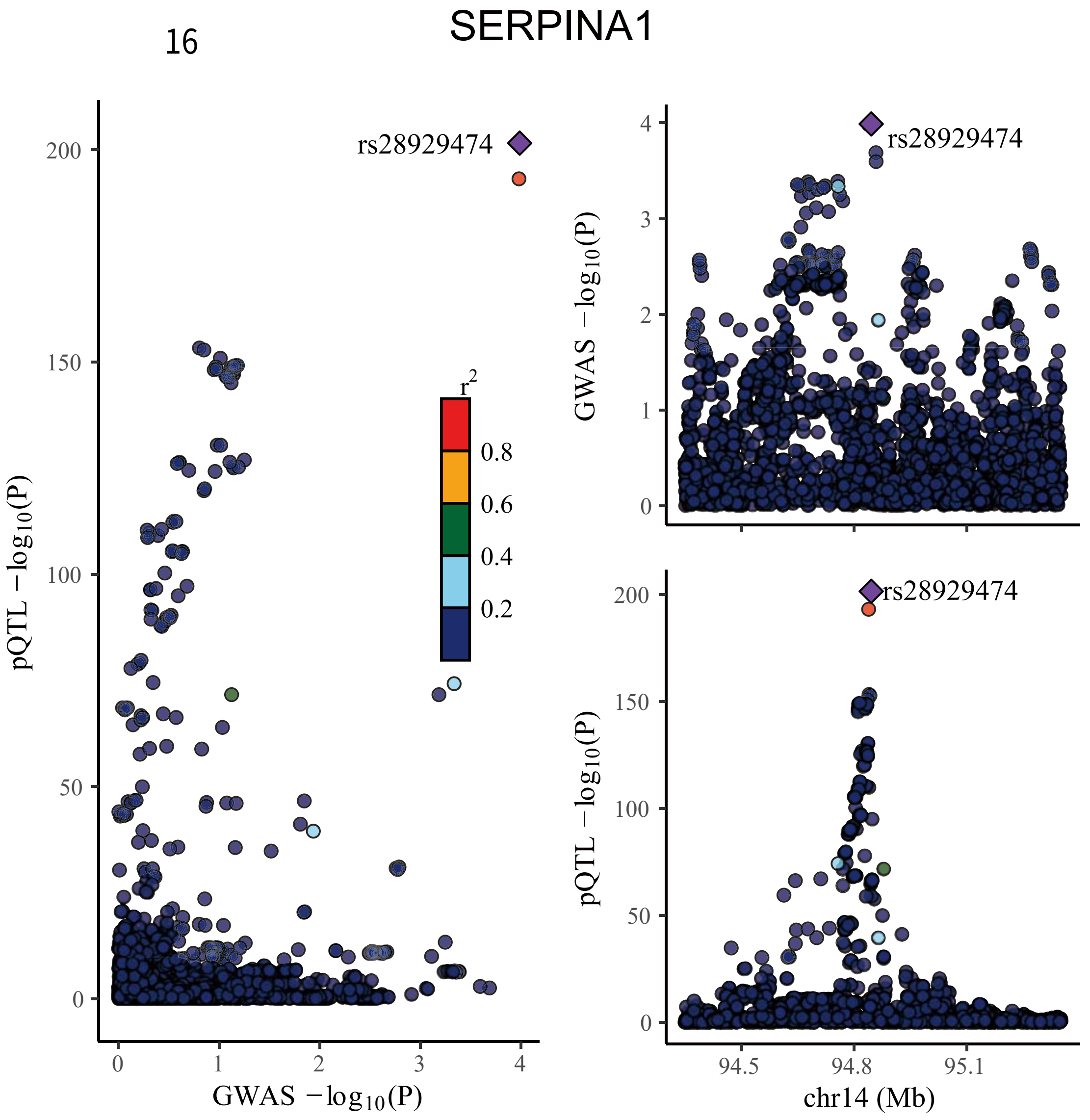

16
SERPINA1
rs28929474
r2
0.8
0.6
0.4
0.2
pQTL −log10(P)
GWAS −log10(P)
chr14 (Mb)
94.5
94.8
95.1

PROC

17

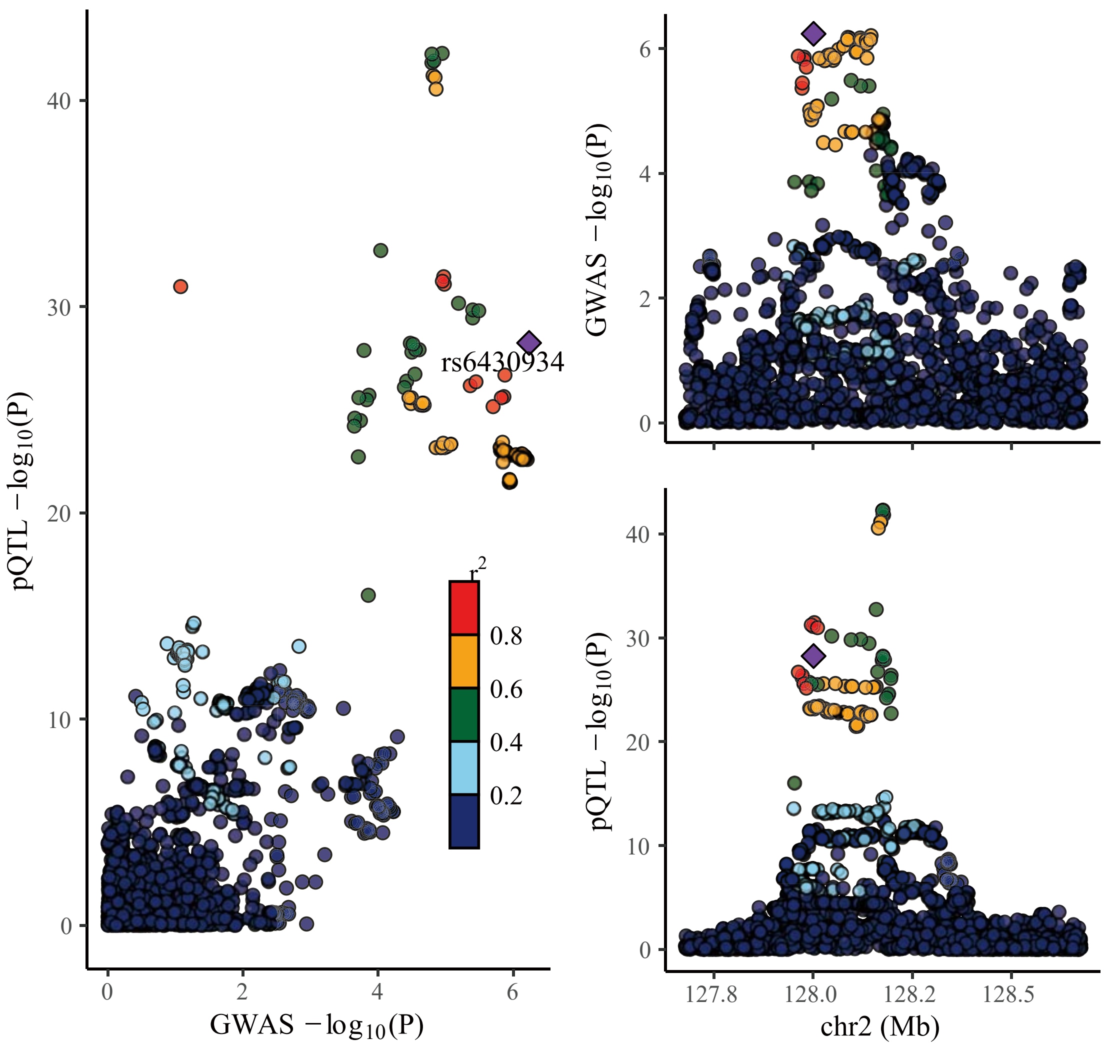

rs6430934
pQTL −log10(P)
GWAS −log10(P)
r2
0.8
0.6
0.4
0.2
chr2 (Mb)
127.8
128.0
128.2
128.5

18

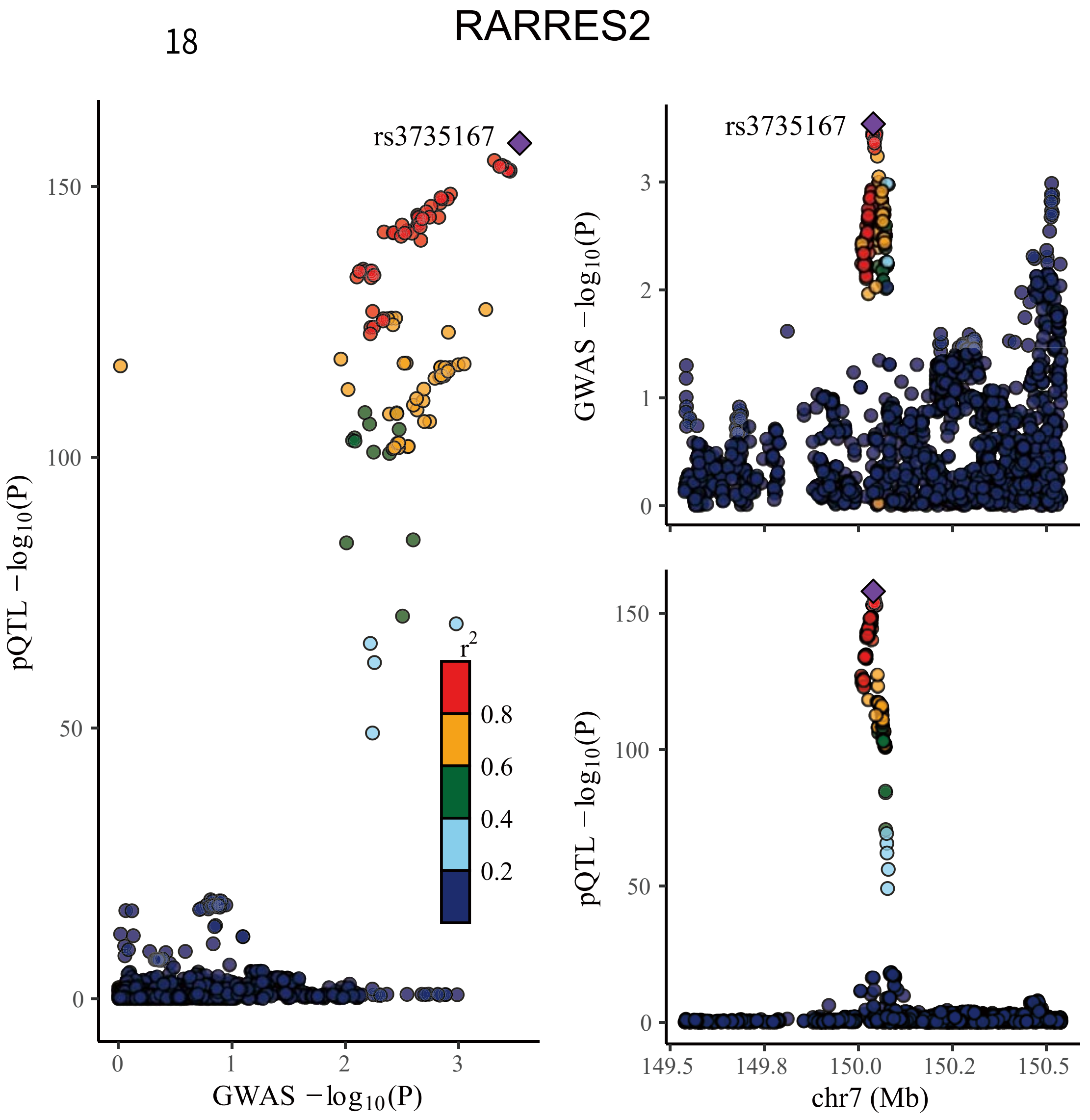
RARRES2
rs3735167
rs3735167
pQTL −log10(P)
GWAS −log10(P)
GWAS −log10(P)
pQTL −log10(P)
chr7 (Mb)
r2
0.8
0.6
0.4
0.2
149.5
149.8
150.0
150.2
150.5

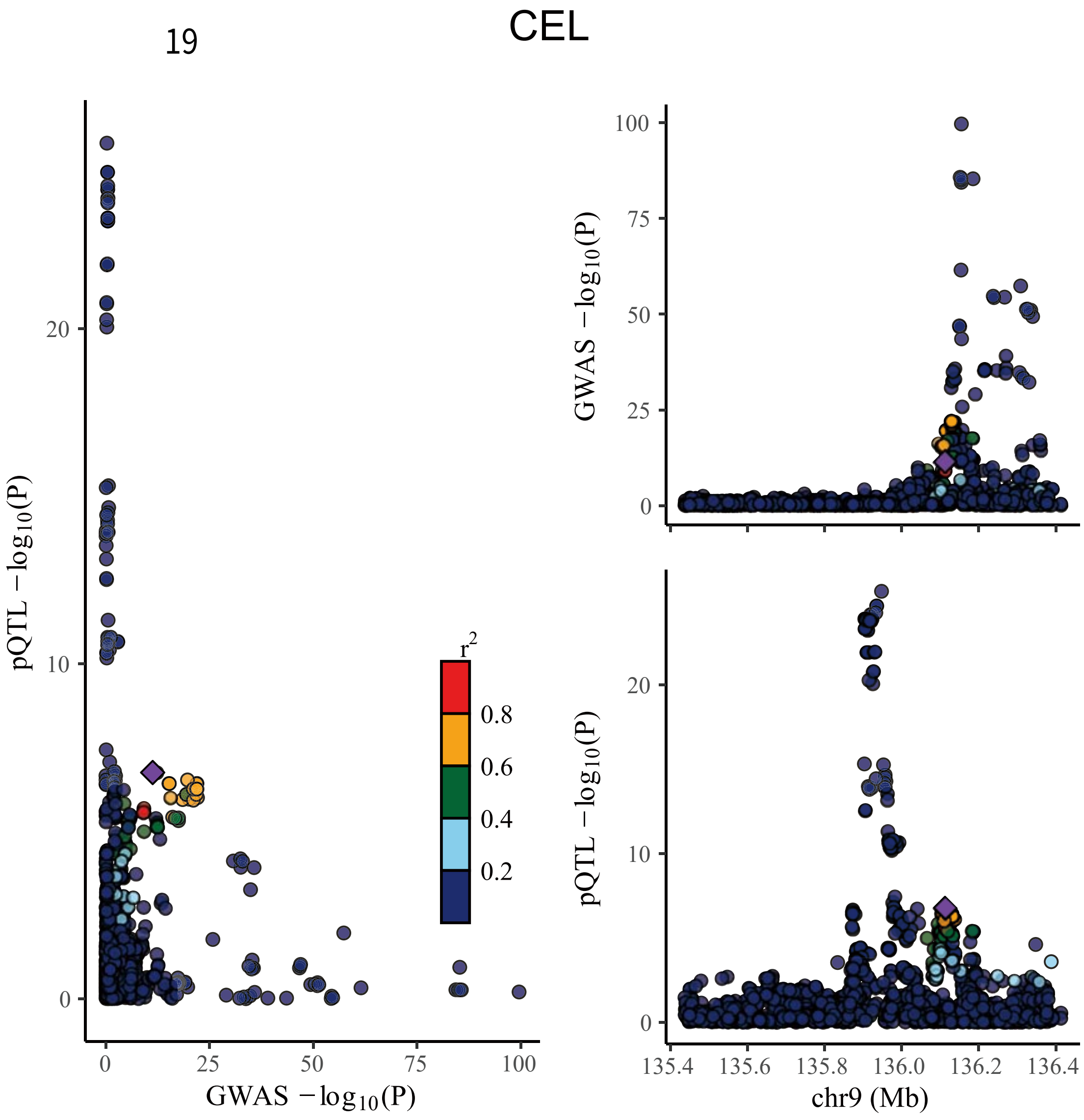
CEL
pQTL −log10(P)
GWAS −log10(P)
r2
0.8
0.6
0.4
0.2
chr9 (Mb)
135.4
135.6
135.8
136.0
136.2
136.4

KLKB1

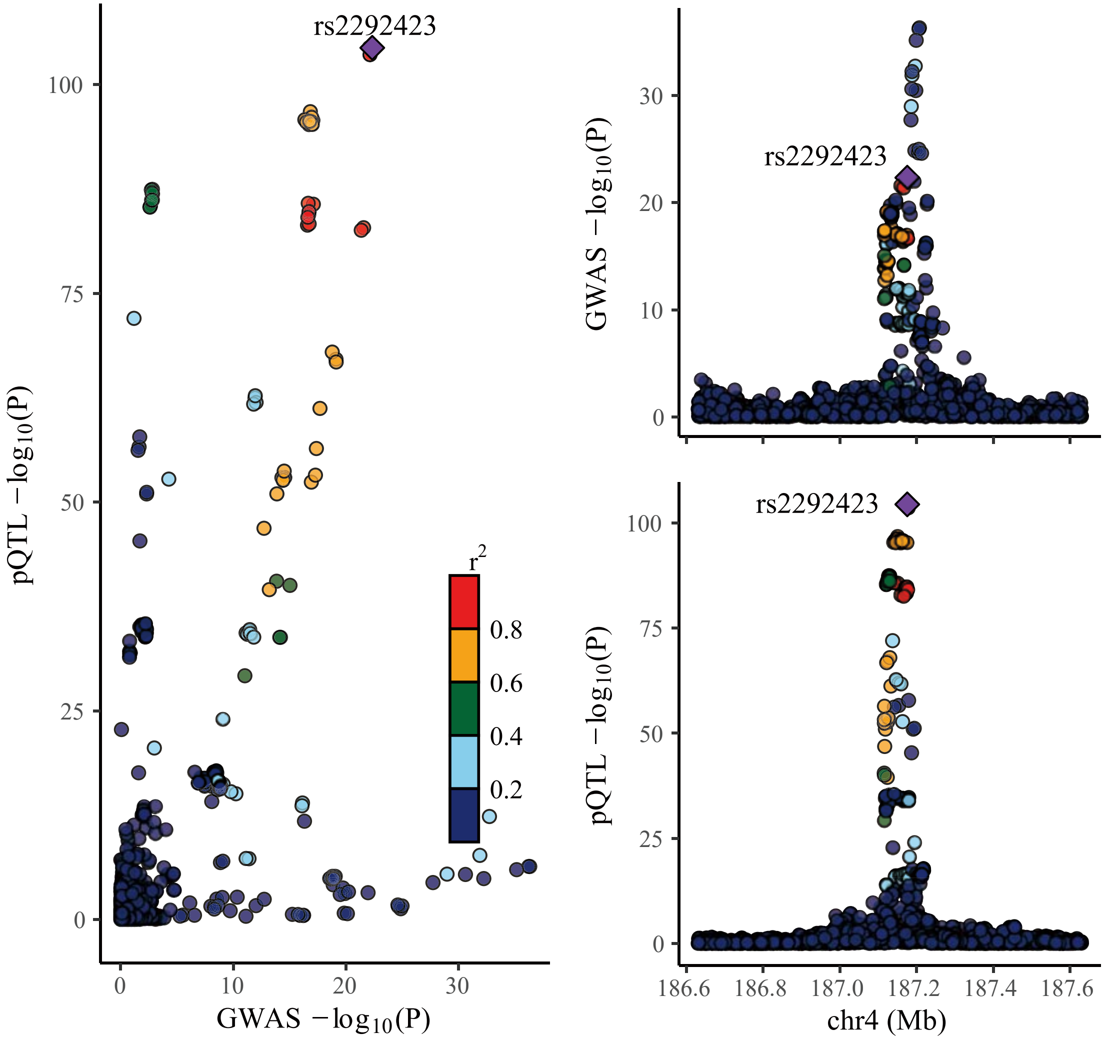
rs2292423
pQTL −log10(P)
GWAS −log10(P)
r2
0.8
0.6
0.4
0.2
100
75
50
25
0
0
10
20
30
rs2292423
GWAS −log10(P)
30
20
10
0
rs2292423
pQTL −log10(P)
100
75
50
25
0
186.6
186.8
187.0
187.2
187.4
187.6
chr4 (Mb)

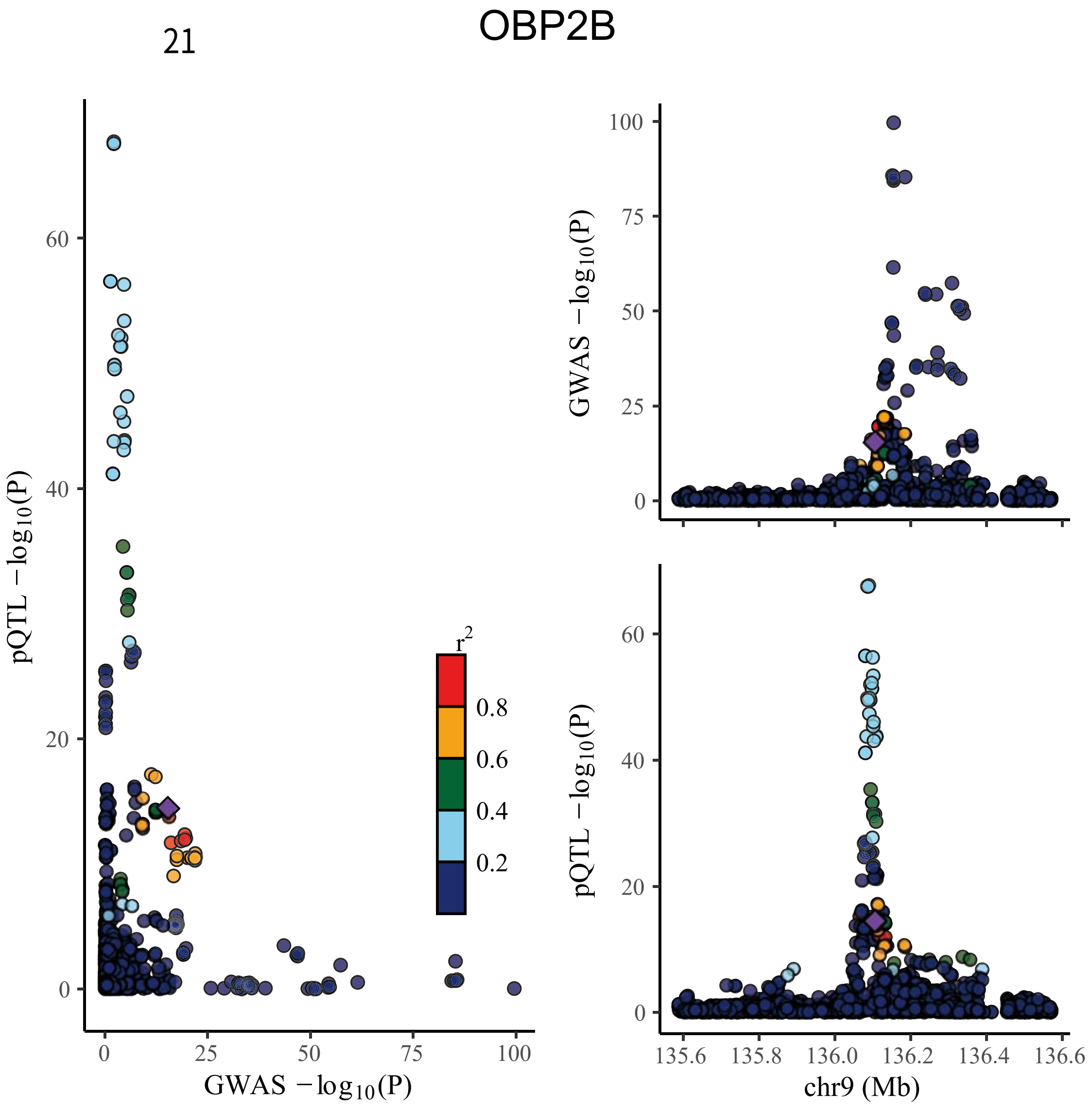
OBP2B
pQTL −log10(P)
GWAS −log10(P)
r2
0.8
0.6
0.4
0.2
chr9 (Mb)
135.6
135.8
136.0
136.2
136.4
136.6

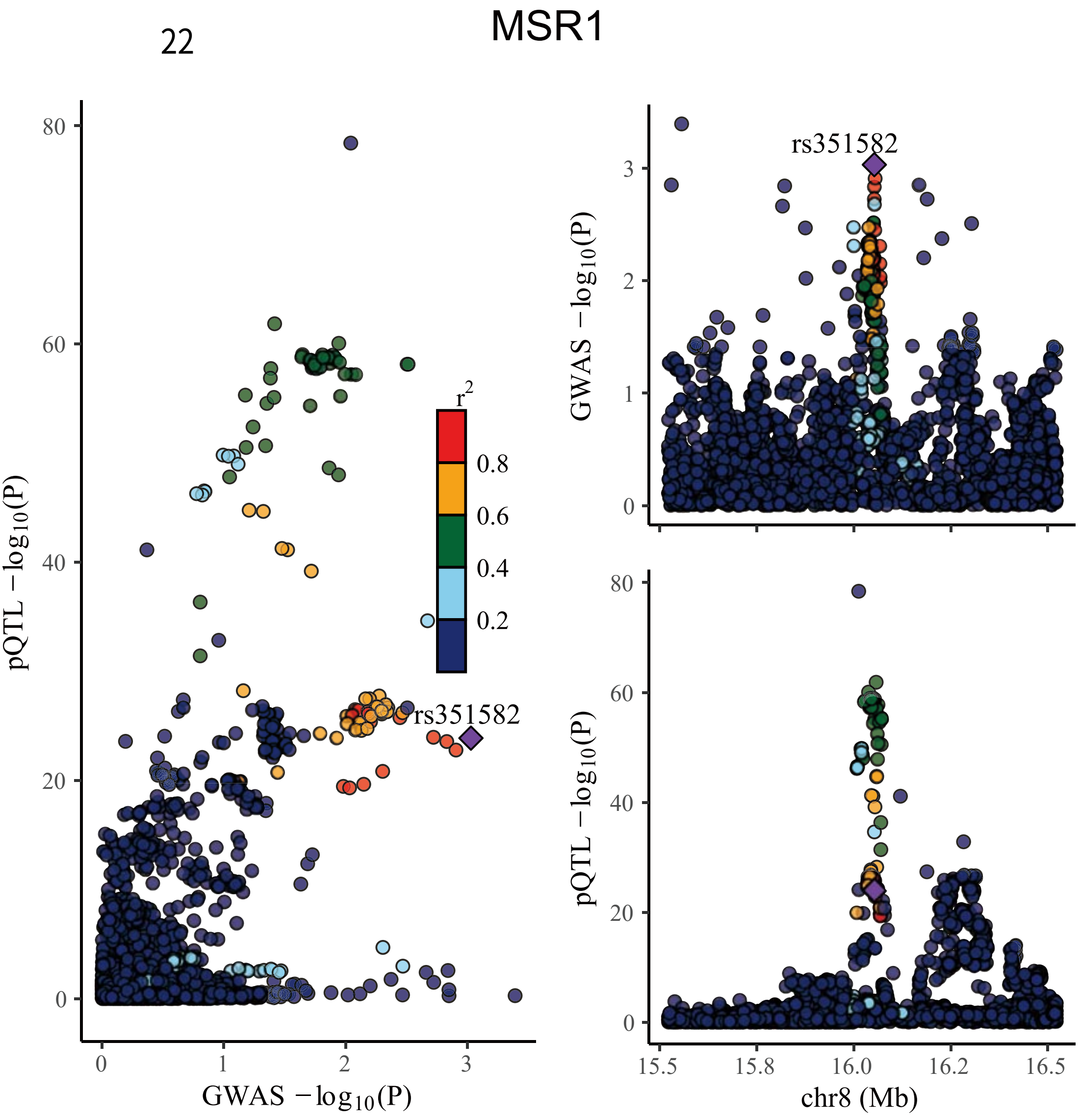
MSR1
pQTL −log10(P)
GWAS −log10(P)
r2
0.8
0.6
0.4
0.2
rs351582
chr8 (Mb)
15.5
15.8
16.0
16.2
16.5

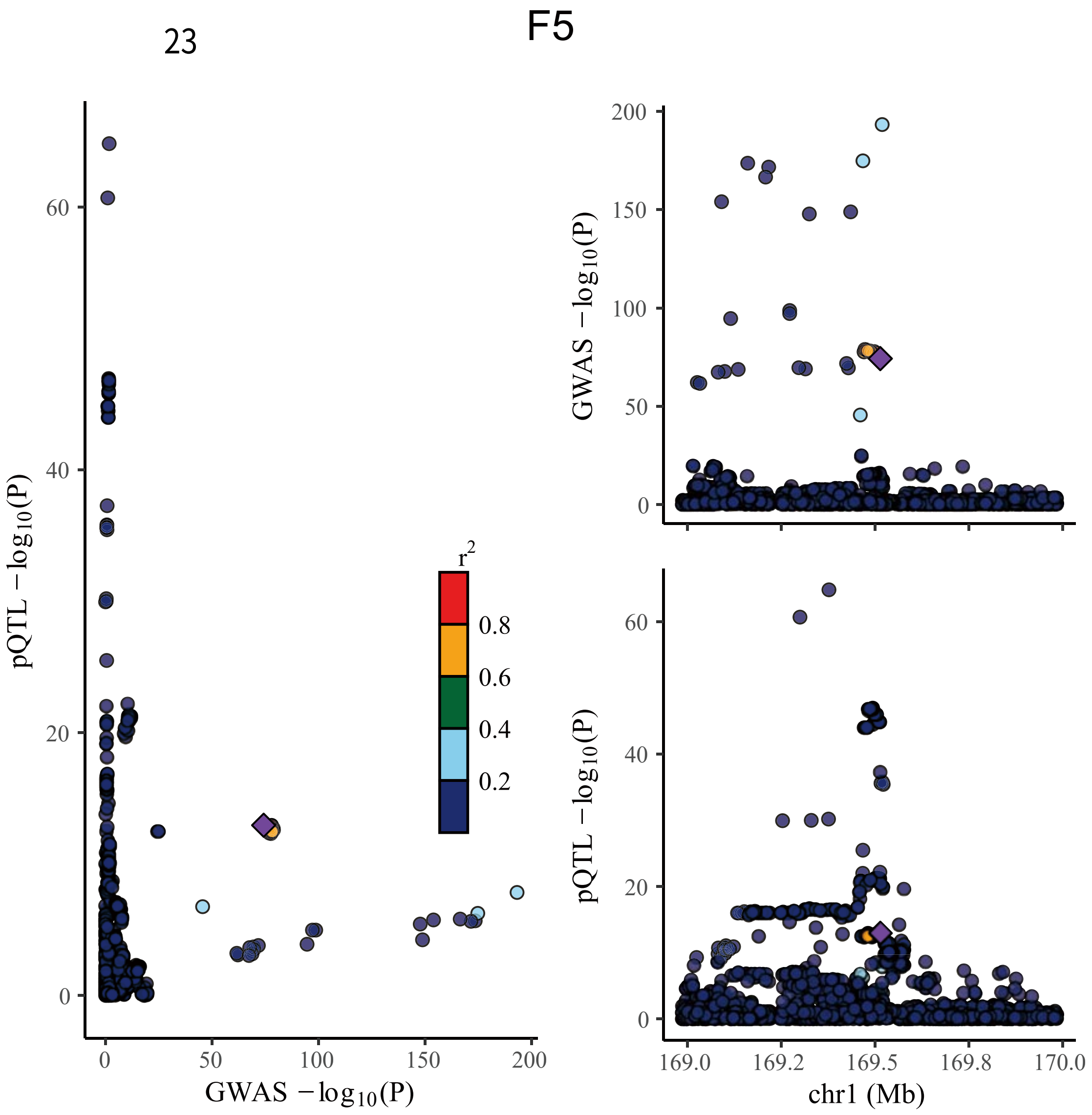

23
F5
pQTL −log10(P)
GWAS −log10(P)
r2
0.8
0.6
0.4
0.2
chr1 (Mb)
169.0
169.2
169.5
169.8
170.0

Supplementary Table 1 Plasma PWAS results in VTE.

SEQID	protein	CHR	P0	P1	HSQ	BEST.GWAS.ID	BEST.GWAS.Z	PQTL.ID	PQTL.R2	PQTL.Z	PQTL.GWAS.Z	NSNP	NWGT	MODEL	MODELCV.R2	MODELCV.PV	PWAS.Z	PWAS.P
SeqId_5316_54	F2	11	46740746	46761056	0.08857	rs1799963	12.243	rs1799963	0.031647	15.25	12.243	21	21	enet	0.049	4.3E-81	14.99308	8.15E-51
SeqId_2190_55	F11	4	187187343	187210835	0.13894	rs2289252	12.714	rs2289252	0.083288	24.6	12.714	78	78	enet	0.11	2E-185	14.2049	8.54E-46
SeqId_9253_52	ABO	9	136108665	136151440	0.18175	rs8176749	9.803	rs8176632	0.29217	45.9	5.15	34	34	enet	0.33	0	7.0967	1.28E-12
SeqId_10070_22	PLCG2	16	81772702	81996290	0.07411	rs12445050	5.92	rs62045706	0.02466	13.65	3.969	55	55	enet	0.052	3E-86	6.2509	4.08E-10
SeqId_19558_10	LRP4	11	46878268	46940193	0.13522	rs1799963	12.243	rs2306029	0.101858	-27.12	-5.926	44	44	enet	0.13	2.1E-222	5.87744	4.17E-09
SeqId_7875_86	PLEK	2	68592414	68624585	0.09408	rs1867312	-5.622	rs1867312	0.09516	26.22	-5.622	41	41	enet	0.12	5E-207	-5.3655	8.07E-08
SeqId_4152_58	KLKB1	4	187130133	187179625	0.07841	rs4241822	9.841	rs2304595	0.062513	21.43	9.828	36	36	enet	0.091	1.4E-151	4.6282	0.00000369
SeqId_2961_1	PROC	2	128176003	128186818	0.01981	rs7585314	-4.835	rs1799809	0.02481	13.66	-4.39	19	19	enet	0.026	1.1E-42	-4.2524	0.0000211
SeqId_15343_337	KNG1	3	186435137	186462199	0.07782	rs5030062	3.46	rs5030062	0.0462	18.4	3.465	54	54	enet	0.074	4.1E-123	4.0359	0.0000544
SeqId_3339_33	THBS2	6	169615875	169654145	0.23366	rs6605524	3.66	rs74507247	0.15851	33.89	-2.807	97	97	enet	0.2	0	-3.948	0.0000787
SeqId_3580_25	SERPINA1	14	94843084	94857030	0.3887	rs28929474	3.884	rs28929474	0.119226	-29.41	3.884	93	93	enet	0.27	0	-3.9103	0.0000922
SeqId_3079_62	RARRES2	7	150035406	150038751	0.07695	rs3735167	-3.625	rs3735167	0.094737	26.19	-3.625	46	46	enet	0.096	8.7E-161	-3.7617	0.000169
SeqId_9796_4	CEL	9	135937368	135947248	0.05484	rs656105	-7.441	rs2075733	0.01444	10.56	-0.553	82	82	enet	0.032	3.1E-53	-3.7235	0.000196
SeqId_3194_36	GP6	19	55525073	55549632	0.18358	rs892090	3.448	rs1613662	0.30436	46.87	3.425	82	82	enet	0.35	0	3.66659	0.000246
SeqId_19154_41	SERPINE2	2	224839762	224904035	0.48586	rs13412535	3.85	rs13412535	0.31113	-47.41	3.85	73	73	enet	0.35	0	-3.6019	0.000316
SeqId_13119_26	SERPINA10	14	94746797	94759605	0.13759	rs7141205	3.962	rs12434093	0.206856	38.64	-2.854	233	233	enet	0.22	0	-3.5187	0.000434
SeqId_5680_54	OBP2B	9	136080664	136084637	0.05287	rs7027827	6.131	rs4454354	0.0408	17.3	-2.698	20	20	enet	0.043	8.8E-71	-3.5126	0.000444
SeqId_8480_29	EFEMP1	2	56093102	56151274	0.07472	rs9309272	3.424	rs3791679	0.06499	21.82	-2.419	45	45	enet	0.082	1E-135	-3.4859	0.00049
SeqId_4906_35	F5	1	169481189	169555826	0.23606	rs2213868	-19.13	rs61808983	0.0647	-21.68	-0.644	54	54	enet	0.11	5.3E-184	3.4547	0.000551
SeqId_15533_97	MSR1	8	15965387	16424999	0.1926	rs79675835	-2.86	rs41341748	0.046749	-18.61	2.61	56	56	enet	0.095	1.3E-158	-3.3926	0.000692
SeqId_9017_58	LCT	2	136545420	136594754	0.34097	rs36084664	3.531	rs191079	0.27647	-44.86	-3.198	73	73	enet	0.31	0	3.3201	0.0009
SeqId_4297_62	SPON1	11	13984270	14289679	0.13759	rs10832182	3.64	rs10832164	0.123763	-29.94	2.353	105	105	enet	0.17	1E-302	-3.30725	0.000942
SeqId_10419_1	SCARA5	8	27727400	27850190	0.05125	rs2685422	3.559	rs2685422	0.024653	-13.79	3.559	74	74	enet	0.035	1.4E-58	-3.2224	0.001271
SeqId_4989_7	FGG	4	155525286	155534119	0.0257	rs1118824	6.655	rs1800790	0.015204	10.72	-5.113	41	41	enet	0.02	3.9E-33	-3.2001	0.00137
SeqId_7784_1	KNG1	3	186435137	186462199	0.23141	rs698078	3.44	rs76438938	0.12	-29.71	2.062	91	91	enet	0.19	0	-3.06558	0.00217
SeqId_9843_5	ACTN1	14	69340845	69446157	0.02481	rs114857623	2.678	rs80123288	0.004356	-8.03	2.671	36	36	enet	0.016	9.6E-27	-3.0388	0.00237
SeqId_9216_100	PLXNB2	22	50713408	50746075	0.28398	rs2273260	3.55	rs28573806	0.20199	38.26	3.056	75	75	enet	0.27	0	3.0062	0.00265
SeqId_16882_27	PHPT1	9	139743176	139745479	0.10386	rs4880149	3.065	rs872463	0.12056	-29.58	2.835	73	73	enet	0.13	4.5E-221	-2.9966	0.00273
SeqId_5703_26	OLFM1	9	137967268	138013026	0.02162	rs4842175	3.36	rs72778149	0.00777	7.64	-2.408	53	53	enet	0.015	1.4E-25	-2.994	0.00275
SeqId_4141_79	CXCL10	4	76942271	76944650	0.0075	rs7679463	-2.855	rs151000561	0.000494	5.94	-2.343	20	20	enet	0.0047	3.1E-09	-2.9624	0.00305
SeqId_7015_8	LILRB5	19	54753279	54761164	0.43643	rs118125339	3.002	rs12975366	0.405162	-54.06	-2.502	54	54	enet	0.46	0	2.96162	0.00306
SeqId_9769_48	DNER	2	230222345	230579271	0.06539	rs10498240	-2.235	rs34661502	0.02462	-13.87	-2.201	82	82	enet	0.061	1.5E-100	2.9366	0.00332
SeqId_9815_5	ARHGAP1	11	46698630	46722169	0.00305	rs11038977	3.629	rs11038977	0.000336	-4.24	3.629	12	12	enet	0.0018	0.0002	-2.93134	0.00338
SeqId_11196_31	COL6A3	2	238232646	238322971	0.06075	rs11694395	2.538	rs1050785	0.02303	12.99	-2.305	49	49	enet	0.034	1.7E-56	-2.9296	0.00339
SeqId_6375_75	XXYLT1	3	194789013	194991888	0.10192	rs55912217	-2.71	rs13072883	0.0431	17.83	-1.039	57	57	enet	0.093	5.1E-156	-2.92229	0.00347
SeqId_4964_67	ERAP1	5	96096514	96143803	0.34597	rs27689	3.713	rs30185	0.356	-50.71	3.543	91	91	enet	0.59	0	-2.92132	0.00349
SeqId_10512_13	CSF2RB	22	37309670	37336481	0.31357	rs2075936	-3	rs2239749	0.24546	-42.1	-2.843	8	8	enet	0.24	0	2.9054	0.00367
SeqId_15388_24	FCGR3A	1	161511549	161520527	0.32822	rs61495795	-3.047	rs10800573	0.412	54.36	2.662	85	85	enet	0.42	0	2.8984	0.003751
SeqId_8250_2	PTPRJ	11	48002110	48192393	0.02046	rs1566734	2.89	rs1566734	0.013797	10.28	2.89	29	1	top1	0.014	8.4E-24	2.89	0.00385
SeqId_4831_4	SELL	1	169659806	169680843	0.2733	rs970740	-18.817	rs4987353	0.145	-32.38	-1.304	60	60	enet	0.23	0	2.8892	0.003862
SeqId_7823_22	ST6GALNAC2	17	74561459	74583038	0.00656	rs62086624	-1.89	rs11077885	0.000141	4.28	-0.771	23	23	enet	0.0025	0.000012	-2.8325	0.00462
SeqId_5676_54	ASIP	20	32782375	32857150	0.11394	rs819167	-4.22	rs6059655	0.128414	-30.46	2.262	26	26	enet	0.13	5.3E-224	-2.8057	0.00502
SeqId_3007_7	SIGLEC9	19	51628163	51639908	0.54829	rs2673908	2.785	rs2075803	0.525008	-61.54	2.768	14	14	enet	0.52	0	-2.80421	0.005044
SeqId_15582_25	FCN1	9	137795078	137809758	0.18904	rs11103571	-2.482	rs7037264	0.11636	28.99	-2.183	56	56	enet	0.19	0	-2.7893	0.00528
SeqId_8885_6	CACNA2D3	3	54156574	55108584	0.10579	rs12639537	3.01	rs6802227	0.0362	16.36	1.946	114	114	enet	0.074	2.6E-123	2.7774	0.00548
SeqId_17691_1	TPP1	11	6633999	6640679	0.06889	rs11040938	3.317	rs11040938	0.025115	13.52	3.317	30	30	enet	0.039	6.7E-65	2.735	0.00624
SeqId_18917_53	AMY2A	1	104160049	104301348	0.4733	rs142370265	-2.633	rs12076610	0.0733	24.09	1.372	143	143	enet	0.23	0	2.7341	0.006255
SeqId_16055_3	CFHR5	1	196946686	196978808	0.39818	rs114245464	-3.89	rs35662416	0.0609	-22.33	2.608	85	85	enet	0.18	3.9e-309	-2.725	0.00643
SeqId_11241_8	ASL	7	65540787	65559684	0.02175	rs77548345	3.919	rs11767072	0.008948	-8.95	-0.4665	31	31	enet	0.027	1.5E-44	2.7242	0.006445
SeqId_8244_16	FUT8	14	65877310	66210839	0.20344	rs117906123	2.426	rs8020305	0.052178	-20.95	1.695	84	84	enet	0.15	6.1E-257	-2.7214	0.0065

SeqId_2700_56	PROS1	3	93591895	93698847	0.06033	rs6795524	3.37	rs6795524	0.0089	-8.11	3.371	116	116	enet	0.028	1.2E-46	-2.68928	0.00716
SeqId_15584_9	CFHR2	1	196912868	196928752	0.66887	rs72732292	-3.585	rs4085749	0.273	-44.41	1.99	81	81	enet	0.52	0	-2.6517	0.008008
SeqId_15468_14	CFHR1	1	196788861	196801319	0.221	rs12402465	-4.117	rs7519758	0.298	-46.41	2.168	67	67	enet	0.3	0	-2.6511	0.008024
SeqId_16823_75	APOL3	22	36536378	36562225	0.28161	rs5995300	-2.76	rs132631	0.31116	47.39	-2.421	41	41	enet	0.32	0	-2.611	0.00903
SeqId_11237_49	PCOLCE	7	100199986	100205798	0.03227	rs221795	3.593	rs7385804	0.022862	13.02	2.015	33	33	enet	0.026	1.1E-43	2.6031	0.009237
SeqId_2879_9	SERPINA3	14	95078721	95090392	0.03891	rs1243510	2.906	rs6575449	0.017954	-11.66	-2.0497	63	63	enet	0.022	1.6E-37	2.5845	0.00975
SeqId_5012_67	AK1	9	130628765	130639954	0.07793	rs112417645	2.982	rs78181560	0.03231	-15.72	2.117	51	51	enet	0.045	5.5E-74	-2.5846	0.00975
SeqId_12334_25	SHMT1	17	18231174	18266877	0.19098	rs4398149	-2.812	rs4398149	0.202244	38.2	-2.812	38	38	enet	0.23	0	-2.579	0.00991
SeqId_3327_27	NTN4	12	96051583	96184967	0.05893	rs78455659	-3.035	rs17288108	0.040368	-17.11	2.563	59	59	enet	0.059	3.2E-97	-2.57697	0.00997
SeqId_3872_2	TPT1	13	45907606	45915419	0.01242	rs116996191	2.54	rs116996191	0.00546	6.45	2.535	21	1	top1	0.0055	2E-10	2.535	0.0112
SeqId_3184_25	F7	13	113760102	113774999	0.27952	rs2480952	-3.16	rs6046	0.32036	-48.3	-2.316	77	77	enet	0.37	0	2.529	0.0114
SeqId_7655_11	NPPB	1	11917521	11919002	0.02252	rs198375	2.561	rs198379	0.0197	12.01	2.432	25	25	enet	0.022	3E-36	2.491	0.012737
SeqId_15556_49	AMY2B	1	104096437	104122156	0.44915	rs142370265	-2.626	rs12076610	0.0612	21.42	1.372	145	145	enet	0.18	9.8e-321	2.4909	0.012744
SeqId_12348_46	SARS2	19	39405906	39421403	0.00766	rs4801971	-2.551	rs1808661	0.005265	-6.63	-2.484	12	1	top1	0.0053	4.1E-10	2.484	0.012992
SeqId_17140_57	PDGFD	11	103777917	104035107	0.15402	rs72974880	-2.933	rs10895596	0.116291	-28.99	1.934	97	97	enet	0.15	8.1E-252	-2.47645	0.0133
SeqId_5112_73	CD200	3	112051194	112081659	0.04029	rs41270439	2.9	rs1879514	0.0167	11.18	2.488	70	70	enet	0.026	1.1E-42	2.47484	0.0133
SeqId_12432_23	CACYBP	1	174968300	174981163	0.05079	rs16847450	-2.445	rs16847450	0.101	-27.06	-2.445	40	1	top1	0.1	1.7E-169	2.445	0.014485
SeqId_4913_78	CCL16	17	34303529	34308533	0.35241	rs113688187	2.983	rs112689088	0.29059	-45.79	2.445	70	70	enet	0.3	0	-2.4432	0.01456
SeqId_15483_377	AGRN	1	955500	991498	0.0733	rs74048006	2.651	rs4970350	0.0412	17.29	-2.15	27	27	enet	0.045	5.2E-75	-2.4374	0.014793
SeqId_4132_27	FST	5	52776456	52782964	0.00519	rs188578758	-2.565	rs546067954	-0.0000742	4.23	-2.562	31	31	enet	0.00016	0.14	-2.42672	0.01524
SeqId_18922_27	CD68	17	7482809	7485429	0.06969	rs9901673	-2.802	rs9901673	0.093962	26.18	-2.802	22	22	enet	0.099	6E-166	-2.4203	0.01551
SeqId_8255_34	MRVI1	11	10594638	10715535	0.04729	rs4909947	3.078	rs1075768	0.021385	-12.57	-1.562	65	65	enet	0.035	1.9E-58	2.39912	0.0164
SeqId_15526_33	GSS	20	33516235	33543830	0.12226	rs6088764	6.84	rs6088642	0.091434	25.78	2.507	36	36	enet	0.11	4.8E-180	2.3936	0.01668
SeqId_16296_43	LGR5	12	71833578	71980090	0.07048	rs7309392	2.762	rs17109804	0.00331	7.13	-1.878	68	68	enet	0.024	5.4E-41	-2.3912	0.01679
SeqId_3310_62	FCGR2B	1	161632937	161648444	0.44673	rs74127517	-2.864	rs6665610	0.39	53.04	-2.355	114	114	enet	0.53	0	-2.3772	0.017445
SeqId_17514_48	RAB21	12	72148643	72194066	0.0196	rs80051868	-2.651	rs2864808	0.007437	-8.81	1.906	50	50	enet	0.014	2.1E-23	-2.37474	0.01756
SeqId_12558_3	UBASH3B	11	122526430	122685181	0.05615	rs7940176	3.104	rs3937026	0.049776	-19	1.52	60	60	enet	0.066	2.8E-109	-2.36089	0.0182
SeqId_9288_7	FKBP7	2	179328391	179343327	0.18172	rs62176114	-3.104	rs1863671	0.16469	34.55	-2.495	72	72	enet	0.19	0	-2.3569	0.0184
SeqId_12494_99	GABARAPL2	16	75600273	75611779	0.02189	rs10781977	-2.43	rs2454867	0.01852	-11.7	1.99	49	49	enet	0.022	2.3E-36	-2.3414	0.0192
SeqId_16915_153	SEMA4A	1	156117157	156147543	0.06382	rs7695	2.187	rs12401997	0.0338	15.77	1.981	40	40	enet	0.038	2.6E-62	2.3251	0.020067
SeqId_12675_14	DARS	2	136663451	136743670	0.03262	rs72617016	-3.44	rs2304371	0.04806	-19.25	1.902	46	46	enet	0.059	4.7E-97	-2.3187	0.0204
SeqId_5202_4	PPID	4	159630279	159644548	0.3464	rs17843966	-2.902	rs17843929	0.298602	46.42	-2.81	29	29	enet	0.34	0	-2.3111	0.0208
SeqId_10761_5	TMED2	12	124069080	124083129	0.0032	rs9919	-2.292	rs9919	0.000911	-4.78	-2.292	8	1	top1	0.00091	0.0059	2.292	0.02191
SeqId_6039_24	CRHBP	5	76248870	76276983	0.15082	rs147231422	2.648	rs10942798	0.192	-37.44	-1.892	65	65	enet	0.22	0	2.28548	0.02228
SeqId_5487_7	SLAMF7	1	160708847	160724611	0.53167	rs77618518	-2.989	rs11581248	0.349	-50.21	-1.967	67	67	enet	0.38	0	2.2821	0.022481
SeqId_15559_5	ANTXR2	4	80822300	81046608	0.06734	rs62297554	-2.701	rs10010217	0.047723	19.9	1.918	63	63	enet	0.094	2.5E-157	2.2753	0.0229
SeqId_16805_5	PDE5A	4	120415552	120549959	0.14279	rs10007283	-3.111	rs58583086	0.11464	-28.83	2.269	87	87	enet	0.16	4.2E-282	-2.2593	0.0239
SeqId_4151_6	PLG	6	161123270	161175129	0.42466	rs144254355	-3.23	rs4252129	0.0765	-23.58	1.714	119	119	enet	0.17	7.2E-293	-2.246	0.0247
SeqId_11368_32	AK2	1	33473541	33546597	0.02471	rs113325293	-2.867	rs188456604	0.000811	-4.94	0.434	30	30	enet	0.0063	9.1E-12	-2.2343	0.025464
SeqId_5658_64	F13B	1	197007871	197036390	0.12153	rs112005340	-3.493	rs1332668	0.123	-30.07	1.873	71	71	enet	0.19	0	-2.2269	0.025952
SeqId_7141_21	MGAT4B	5	179224597	179233952	0.10799	rs155787	-2.337	rs113756550	0.0968	-26.76	-2.134	46	46	enet	0.1	1.6E-174	2.20605	0.02738
SeqId_5256_86	PDE5A	4	120415552	120549959	0.08502	rs983417	2.773	rs58583086	0.094649	-26.21	2.269	40	40	enet	0.12	1.1E-203	-2.1894	0.0286
SeqId_3364_76	CTSV	9	99791959	99918838	0.03887	rs72749658	2.502	rs4743059	0.02075	12.4	-2.14	60	60	enet	0.033	4.3E-54	-2.1882	0.0287
SeqId_13123_3	FLRT3	20	14303631	14318260	0.38155	rs149195265	2.66	rs1932953	0.286093	45.53	2.46	103	103	enet	0.42	0	2.1623	0.03059
SeqId_3484_60	AGT	1	230826522	230881322	0.06843	rs145082147	3.093	rs2071404	0.0327	17	-1.745	84	84	enet	0.058	2.5E-95	-2.1235	0.033708
SeqId_5722_78	PRCP	11	82533978	82681626	0.05268	rs12808296	2.322	rs2229437	0.034518	15.83	1.56	63	63	enet	0.043	1.6E-70	2.11604	0.0343
SeqId_8960_3	ERAP2	5	96211690	96255407	0.21865	rs2927608	-2.718	rs2910686	0.478	58.7	-2.716	110	110	enet	0.56	0	-2.11163	0.03472
SeqId_5749_53	CLPS	6	35762759	35765121	0.2757	rs2478468	-2.39	rs9380534	0.08079	24.2	-1.4747	147	147	enet	0.15	1.9E-252	-2.097	0.036
SeqId_3421_54	TNFSF8	9	117655623	117692875	0.03383	rs7047097	-2.474	rs3181348	0.02795	-14.53	2.294	32	32	enet	0.035	4E-58	-2.0956	0.0361
SeqId_13095_51	REG1A	2	79347488	79350535	0.15112	rs72812640	-2.156	rs11126696	0.04029	17.1	0.939	73	73	enet	0.093	5E-155	2.0819	0.0373
SeqId_5646_20	RNASE6	14	21249427	21250626	0.30203	rs11620733	-2.864	rs1045922	0.411804	54.53	-1.621	54	54	enet	0.45	0	-2.0776	0.0377
SeqId_17722_5	FKBP4	12	2904136	2914589	0.13	rs80079929	-3.996	rs56196860	0.063577	-21.48	-1.948	75	75	enet	0.078	9.6E-129	2.07384	0.03809
SeqId_5018_68	PRDX6	1	173446469	173457954	0.04502	rs33951697	-1.976	rs33951697	0.0784	24.06	-1.976	36	36	enet	0.093	9.9E-156	-2.0676	0.038677

SeqId_7266_4	SERPINA9	14	94929054	94946026	0.04229	rs11621961	3.409	rs11160181	0.017196	-11.5	0.455	33	33	enet	0.034	3.7E-57	-2.05	0.0404
SeqId_13126_52	DSC2	18	28638806	28682485	0.14464	rs62087332	2.53	rs1790683	0.04622	-18.89	2.227	49	49	enet	0.1	6.4E-169	-2.043	0.0411
SeqId_7779_86	CHST11	12	104849073	105155792	0.16039	rs11112132	-3.306	rs1704878	0.135637	-31.38	-1.695	113	113	enet	0.17	2.2E-302	2.04199	0.04115
SeqId_14273_19	PREP	6	105721093	105901937	0.20479	rs77207716	2.19	rs1051484	0.16438	34.47	1.439	93	93	enet	0.26	0	2.038	0.0415
SeqId_11117_2	SPATA20	17	48620419	48633213	0.14696	rs198542	-3.116	rs9890200	0.212866	-39.21	2.374	40	40	enet	0.22	0	-2.0324	0.04211
SeqId_6485_59	IGLL1	22	23915312	23922489	0.14933	rs140174	-2.25	rs9624216	0.04274	-17.62	-1.563	64	64	enet	0.06	9.4E-100	2.0317	0.04218
SeqId_6925_26	SNX8	7	2291405	2393953	0.06097	rs798553	-2.996	rs28514741	0.017219	-11.61	2.002	62	62	enet	0.028	9.3E-47	-2.0247	0.042903
SeqId_14208_3	RBP7	1	10057264	10076079	0.16397	rs35232720	2.145	rs35232720	0.106	-27.81	2.145	37	37	enet	0.14	1.6E-231	-2.0174	0.043652
SeqId_14088_38	IGFBP6	12	53491220	53496129	0.01544	rs7958457	-2.582	rs822688	0.011543	-9.3	1.683	32	32	enet	0.012	1.4E-20	-2.01464	0.04394
SeqId_4332_6	CLEC1B	12	10138241	10166023	0.11627	rs521040	2.375	rs521040	0.064748	22.43	2.375	44	44	enet	0.13	4.4E-228	2.01423	0.04399
SeqId_6383_90	TLL1	4	166794389	167025609	0.02499	rs10022413	2.144	rs28569800	0.000895	-4.98	1.822	68	68	enet	0.0066	2.6E-12	-2.013	0.0441
SeqId_17697_2	OVCA2	17	1945316	1946719	0.16487	rs12949014	3.043	rs145234879	0.05454	20	1.395	49	49	enet	0.077	9.7E-128	2.0072	0.04473
SeqId_15326_64	GBP1	1	89517565	89531043	0.0342	rs12143830	2.733	rs61798920	0.0115	-9.66	-1.982	54	54	enet	0.024	2.7E-40	1.9942	0.046126
SeqId_19251_56	SRGN	10	70847859	70864561	0.17612	rs117372387	2.38	rs2229498	0.066052	21.93	1.614	38	38	enet	0.11	6.2E-189	1.99309	0.0463
SeqId_13954_9	GNPNAT1	14	53241911	53258386	0.05646	rs2147977	2.4	rs2273743	0.056035	20.25	-1.995	38	38	enet	0.059	3E-97	-1.9853	0.0471
SeqId_15565_102	MUC16	19	8959520	9092018	0.03464	rs1423049	-2.398	rs193266507	0.010115	-8.69	-2.1591	52	52	enet	0.02	2.3E-34	1.9836	0.0473
SeqId_16561_9	A1BG	19	58856544	58864858	0.15749	rs1268538	-1.992	rs1268538	0.113266	-28.62	-1.992	72	72	enet	0.13	1.6E-214	1.97771	0.047962
SeqId_4153_11	SERPINA3	14	95078721	95090392	0.13705	rs28929474	3.884	rs17753556	0.083401	-24.61	-2.4046	61	61	enet	0.12	2.4E-196	1.9743	0.0483
SeqId_11530_37	HMBS	11	118955576	118964259	0.05483	rs45510602	2.217	rs72995454	0.008039	8.73	-0.967	37	37	enet	0.02	1E-33	-1.96856	0.049
SeqId_8974_172	COL15A1	9	101705461	101833074	0.10037	rs144504148	-3.032	rs10819566	0.04721	20.14	2.258	105	105	enet	0.15	6.5E-252	1.9659	0.0493
SeqId_2615_60	EFNA5	5	106712595	107006638	0.09505	rs78246717	2.7	rs7734574	0.0368	-16.83	-1.204	127	127	enet	0.082	3.7E-137	1.96593	0.04931
SeqId_8664_36	PKDCC	2	42275161	42285657	0.09315	rs4953236	2.73	rs13416595	0.04481	18.12	-1.261	29	29	enet	0.065	2.5E-108	-1.9648	0.0494
SeqId_9294_45	MFAP2	1	17300997	17307127	0.07799	rs3754508	2.412	rs761422	0.0728	23.65	0.897	29	29	enet	0.099	1E-165	1.9631	0.049636
SeqId_15491_20	CD248	11	66081965	66084515	0.01364	rs565972	2.236	rs565972	0.002212	5.71	2.236	18	18	enet	0.0029	0.0000033	1.95887	0.0501
SeqId_11387_3	ATF6B	6	32083041	32096030	0.17412	rs143504217	-3.39	rs6449	0.03036	14.9	0.5618	86	86	enet	0.054	9.8E-90	1.951	0.0511
SeqId_18930_28	SLIT2	4	20253528	20622184	0.03448	rs6833662	-2.115	rs587668	0.009457	-8.57	-1.143	65	65	enet	0.025	2.4E-41	1.9485	0.0514
SeqId_5231_79	PCSK9	1	55505120	55530525	0.08479	rs80192040	-2.066	rs11591147	0.0296	-14.96	1.384	51	51	enet	0.045	9.8E-75	-1.9476	0.051468
SeqId_2617_56	ERBB3	12	56470583	56497289	0.04228	rs3759094	-2.604	rs2292238	0.018141	-11.85	-1.947	60	1	top1	0.018	9E-31	1.947	0.05153
SeqId_16770_3	REG1B	2	79312149	79315145	0.13798	rs10520215	2.164	rs11126696	0.04901	18.85	0.942	67	67	enet	0.09	2.7E-149	1.9464	0.0516
SeqId_7955_195	ITIH1	3	52811611	52826084	0.2007	rs4434138	-2.47	rs1042779	0.224	-40.33	-1.777	35	35	enet	0.23	0	1.94291	0.052
SeqId_5103_30	CD200R1	3	112640052	112693950	0.0434	rs114921967	2.03	rs62263730	0.0249	13.56	1.81	36	36	enet	0.036	2.5E-60	1.93863	0.0525
SeqId_6574_11	FAIM3	1	207076662	207096592	0.00896	rs141229030	-2.614	rs72758947	0.00735	-7.43	1.939	33	1	top1	0.0073	1.8E-13	-1.939	0.052501
SeqId_2644_11	PRKCA	17	64298731	64806861	0.09878	rs78483340	-2.102	rs61762372	0.043912	17.84	-1.2115	80	80	enet	0.062	6.9E-102	-1.9387	0.05253
SeqId_13388_57	PCSK1	5	95726037	95769847	0.26495	rs145943387	-2.557	rs13169290	0.399	-53.66	1.89	43	43	enet	0.4	0	-1.93452	0.05305
SeqId_19194_9	DTD1	20	18568586	18747288	0.09813	rs7272185	-2.77	rs6081231	0.272812	-44.38	-2.03599	65	65	enet	0.28	0	1.9335	0.05317
SeqId_3640_14	LRPAP1	4	3505339	3534173	0.09284	rs556871906	-1.993	rs16844464	0.057597	-20.44	-1.476	35	35	enet	0.067	2.3E-110	1.9278	0.0539
SeqId_9037_1	STX18	4	4417469	4544073	0.00587	rs1000579	1.923	rs1000579	0.000137	-4.02	1.923	13	1	top1	0.00014	0.16	-1.923	0.0545
SeqId_15560_52	TCN2	22	31002990	31023258	0.22121	rs3887855	-2.14	rs4820885	0.4067	-54.19	-1.878	42	42	enet	0.41	0	1.9161	0.05535
SeqId_6947_4	ST3GAL6	3	98451080	98540045	0.28141	rs191648239	-2.68	rs28489284	0.232	-41.06	1.69	90	90	enet	0.35	0	-1.91405	0.0556
SeqId_2991_9	IL1R1	2	102681004	102796334	0.05475	rs13019803	2.759	rs2287047	0.02085	-12.71	1.603	63	63	enet	0.039	4.4E-65	-1.9089	0.0563
SeqId_2837_3	MET	7	116312250	116438431	0.06393	rs12531787	-2.703	rs41748	0.01832	-11.65	-0.733	65	65	enet	0.04	5.5E-66	1.9041	0.056897
SeqId_18386_36	GLRX	5	95087023	95158430	0.00945	rs145128963	-2.676	rs6556884	0.00312	-6.33	-1.436	17	17	enet	0.0057	9.2E-11	1.89717	0.05781
SeqId_16914_104	CD14	5	140011313	140013286	0.08742	rs2337225	-3.104	rs5744441	0.0357	-16.1	0.216	58	58	enet	0.053	5.7E-87	-1.89694	0.05784
SeqId_2665_26	TNFRSF17	16	12059067	12061925	0.02402	rs117744064	2.61	rs387871	0.01394	-10.14	-1.522	38	38	enet	0.015	9E-26	1.8866	0.0592
SeqId_5475_10	PRKCB	16	23847304	24231932	0.04362	rs7185142	2.77	rs2023671	0.03403	-15.7	2.714	20	20	enet	0.05	1.9E-83	-1.8814	0.0599
SeqId_18172_71	ASF1A	6	119215316	119230336	0.00695	rs118087341	-2.07	rs4946366	0.00399	6.03	-1.879	23	1	top1	0.004	0.000000048	-1.879	0.0602
SeqId_15370_5	BOLA1	1	149859440	149872351	0.08213	rs67567469	2.009	rs1376675	0.165	34.78	-1.903	23	23	enet	0.17	5.7E-288	-1.8747	0.060837
SeqId_18218_48	CNRIP1	2	68511303	68547183	0.166	rs7424155	-3.207	rs7604489	0.16899	-35.26	2.305	48	48	enet	0.19	0	-1.8711	0.0613
SeqId_3581_53	AHSG	3	186330849	186339107	0.17319	rs13322149	-2.5	rs2518134	0.11	29.68	1.95	78	78	enet	0.19	0	1.86941	0.0616
SeqId_5483_1	RGMA	15	93578503	93632433	0.07674	rs4777659	-1.925	rs10852188	0.02502	-14.33	-1.477	57	57	enet	0.051	3.5E-84	1.8676	0.0618
SeqId_6471_53	CFHR4	1	196857144	196887843	0.60863	rs114245464	-3.89	rs10494745	0.165	-34.57	-1.028	124	124	enet	0.53	0	-1.8627	0.062507
SeqId_8957_72	ERLEC1	2	54014181	54045956	0.02738	rs2111853	-1.919	rs2692523	0.01627	-11.31	1.286	38	38	enet	0.017	8.7E-29	-1.8553	0.0636
SeqId_5939_42	TNFSF12	17	7452375	7461207	0.01458	rs141859743	2.375	rs62059804	0.010539	9.47	1.853	15	1	top1	0.011	1.4E-18	1.853	0.06388

SeqId_9197_4	LGALS9	17	25956824	25976586	0.03611	rs11080143	-2.154	rs4239242	0.03653	-17.48	1.3873	18	18	enet	0.05	8.1E-83	-1.8342	0.06662
SeqId_2480_58	TIMP3	22	33197691	33259028	0.14622	rs112339381	2.21	rs5749506	0.17671	-35.82	-1.673	63	63	enet	0.21	0	1.8243	0.06811
SeqId_9369_174	LRRC4C	11	40135753	41481323	0.06141	rs998447	-2.293	rs10837377	0.035788	-16.1	1.989	43	43	enet	0.041	2.3E-68	-1.82077	0.0686
SeqId_17726_3	SAR1A	10	71907045	71930279	0.05317	rs6480445	2.75	rs4746970	0.077617	23.73	1.487	32	32	enet	0.079	2.2E-131	1.820384	0.0687
SeqId_15562_24	GUSB	7	65425671	65447202	0.05011	rs62468669	3.906	rs2949690	0.013504	-10.62	-0.347	65	65	enet	0.024	7.7E-41	1.8204	0.068705
SeqId_15579_26	ENPP6	4	185009859	185142383	0.00734	rs145682264	-2.813	rs28673999	0.002671	5.43	-1.216	8	8	enet	0.003	0.0000021	-1.8174	0.0691
SeqId_16322_10	PACAP	5	138723156	138725770	0.00452	rs116498185	1.901	rs116498185	0.00313	5.69	1.901	25	25	enet	0.0053	3.4E-10	1.81694	0.06923
SeqId_13107_9	LYPD3	19	43964939	43969760	0.04201	rs2854510	2.526	rs11666797	0.011615	-10.66	-0.83	35	35	enet	0.028	6.2E-46	1.80085	0.071727
SeqId_5701_81	CLEC3B	3	45043040	45077563	0.10929	rs76951547	-2.46	rs10865936	0.043	-17.75	-0.528	54	54	enet	0.062	2.2E-103	1.7979	0.0722
SeqId_10832_24	B4GALT6	18	29202209	29265799	0.21317	rs112233114	-2.45	rs113222817	0.16847	-34.98	-1.39	79	79	enet	0.2	0	1.789	0.0737
SeqId_3622_33	LGMN	14	93170152	93215024	0.08404	rs117866656	-2.201	rs148659834	0.019336	-12.04	-0.193	50	50	enet	0.055	5.5E-91	1.7791	0.0752
SeqId_19563_3	SEZ6L	22	26565428	26779563	0.10671	rs738399	-2.65	rs655581	0.01264	-10.84	0.639	96	96	enet	0.078	4E-129	-1.7663	0.07734
SeqId_9341_1	PDGFD	11	103777917	104035107	0.11541	rs116922192	-2.991	rs10791660	0.047001	-18.49	1.205	107	107	enet	0.081	9.8E-135	-1.75887	0.0786
SeqId_15522_2	GLIPR2	9	36136533	36163910	0.06877	rs139631985	3.138	rs10814339	0.04861	-18.83	0.575	47	47	enet	0.055	3.1E-90	-1.7509	0.08
SeqId_9449_150	C4BPA	1	207277578	207318317	0.17266	rs76169194	3.556	rs11120218	0.133	31.67	1.435	49	49	enet	0.15	2.4E-265	1.7481	0.08044
SeqId_11102_22	REG4	1	120336640	120354203	0.02961	rs61598511	2.59	rs2298069	0.016	-10.85	2.552	37	37	enet	0.02	5.8E-33	-1.7456	0.080874
SeqId_7228_2	ST6GALNAC(	9	130647600	130667687	0.00933	rs7873657	3.046	rs61740973	0.00197	-5.47	-1.82	37	37	enet	0.0036	0.00000019	1.7241	0.0847
SeqId_15314_49	CLIC4	1	25071873	25170812	0.05049	rs35068083	-2.555	rs4649017	0.0317	-15.24	1.04	46	46	enet	0.051	5.5E-85	-1.723	0.084893
SeqId_16583_8	NUDT2	9	34329504	34343709	0.31868	rs190476469	-3.263	rs10971956	0.27336	44.92	1.218	52	52	enet	0.48	0	1.7206	0.0853
SeqId_17513_11	ANXA11	10	81910645	81965328	0.31245	rs34965541	-2.11	rs2819952	0.190279	37.17	-1.254	138	138	enet	0.24	0	-1.700277	0.0891
SeqId_13423_94	FAM213A	10	82167585	82196871	0.00709	rs6585994	2.7	rs10788623	0.001725	-5.54	1.693	38	1	top1	0.0017	0.00025	-1.693	0.0905
SeqId_11219_95	FGFBP3	10	93666341	93669243	0.15666	rs61876139	-1.88	rs11186737	0.153907	-33.33	-1.351	52	52	enet	0.17	1E-295	1.686045	0.0918
SeqId_7049_2	ADAM23	2	207308256	207485851	0.42113	rs147157136	-3.405	rs1448903	0.11445	29.55	1.068	99	99	enet	0.3	0	1.6857	0.0918
SeqId_3727_35	PYY	17	42030106	42081837	0.00988	rs9906272	-2.021	rs12449853	0.004902	6.62	-1.682	24	1	top1	0.0049	1.6E-09	-1.682	0.09257
SeqId_17153_46	KIR2DL3	19	55249978	55264504	0.13336	rs74746021	4.046	rs4806594	0.018237	11.7	0.0138	182	182	enet	0.061	6.7E-101	1.68159	0.092648
SeqId_16781_2	ENGASE	17	77070906	77084681	0.31104	rs2612767	3.082	rs56107536	0.106916	27.81	-0.997	53	53	enet	0.27	0	-1.6792	0.0931
SeqId_2611_72	TYRO3	15	41849873	41875787	0.05947	rs142733061	2.831	rs1200353	0.026	-13.76	-1.54	37	37	enet	0.039	1.9E-64	1.6785	0.0932
SeqId_9839_148	TIRAP	11	126152960	126168740	0.20199	rs11605349	3.208	rs8177399	0.08606	-24.95	-0.678	78	78	enet	0.14	2E-230	1.67427	0.0941
SeqId_5452_71	ASGR1	17	7076750	7082883	0.06075	rs62059194	3.972	rs62061425	0.017999	-11.55	0.576	72	72	enet	0.036	2.5E-60	-1.6664	0.09562
SeqId_2843_13	SPINT2	19	38734675	38783255	0.2563	rs4804046	-2.966	rs71354995	0.276703	-44.71	-1.669	91	91	enet	0.31	0	1.66271	0.09637
SeqId_8890_9	TMEM132B	12	125670932	126146923	0.05523	rs7974263	-2.464	rs3825381	0.040488	-17.21	2.241	30	30	enet	0.057	3.7E-94	-1.65933	0.09705
SeqId_15495_9	FOLR3	11	71825915	71850936	0.28828	rs7926875	-2.233	rs146694036	0.177043	35.75	-1.406	47	47	enet	0.24	0	-1.65608	0.0977
SeqId_3311_27	FCGR3B	1	161592986	161601753	0.1357	rs3813621	-3.433	rs10800573	0.0923	25.96	1.524	69	69	enet	0.11	3.9E-185	1.6544	0.098043
SeqId_17739_1	HADH	4	108910870	108956397	0.0211	rs221334	2.268	rs72890511	0.006993	8.47	-1.901	60	60	enet	0.015	4.4E-25	-1.654	0.0981
SeqId_8274_64	STX7	6	132767006	132834337	0.04586	rs72996835	2.46	rs3813356	0.02187	-13.25	1.5867	11	11	enet	0.028	3.4E-47	-1.646	0.0998
SeqId_6223_5	GUCA2B	1	42619081	42621491	0.0149	rs883240	2.27	rs1047047	0.00965	9.29	-1.511	9	9	enet	0.011	1.1E-18	-1.6451	0.099954
SeqId_13671_40	ELANE	19	851014	856247	0.05685	rs111607343	-2.214	rs56283881	0.019409	-12.58	0.8092	41	41	enet	0.033	1.4E-54	-1.64198	0.100594
SeqId_3396_54	REN	1	204123947	204159452	0.01193	rs2796369	-1.801	rs141995914	0.012	9.38	1.249	30	30	enet	0.014	2E-23	1.6393	0.101141
SeqId_9557_5	MANSC1	12	12478990	12503176	0.066	rs117197894	2.615	rs3741798	0.083555	24.69	1.692	33	33	enet	0.084	9.4E-140	1.63525	0.102
SeqId_9870_17	WARS	14	100800127	100843142	0.0749	rs77235285	3.213	rs4905957	0.148254	32.72	-1.636	35	1	top1	0.15	8E-254	-1.636	0.102
SeqId_11424_4	FAH	15	80444832	80479288	0.3787	rs74647991	-2.264	rs11555096	0.11425	-28.74	-1.643	71	71	enet	0.14	1.3E-243	1.633	0.1025
SeqId_12498_12	TAX1BP3	17	3566189	3571973	0.02412	rs222790	-2.787	rs160589	0.010649	8.99	0.043	32	32	enet	0.013	4.8E-23	-1.6327	0.10253
SeqId_9578_263	MANSC4	12	27915360	27933169	0.14736	rs4244857	2.411	rs12368818	0.282782	45.23	1.415	48	48	enet	0.29	0	1.63113	0.10286
SeqId_5124_69	ICAM5	19	10400628	10407454	0.3079	rs8113381	-4.788	rs281440	0.228089	40.6	-2.495	66	66	enet	0.29	0	-1.61856	0.105542
SeqId_3309_2	FCGR2A	1	161475220	161493803	0.44351	rs112402038	2.374	rs1801274	0.487	59.25	-1.461	34	34	enet	0.49	0	-1.6183	0.105602
SeqId_9212_22	CTSF	11	66330935	66336350	0.03495	rs148723727	-2.362	rs1044522	0.031638	15.24	1.349	55	55	enet	0.032	5.2E-53	1.61818	0.106
SeqId_16288_17	EPHA4	2	222282747	222438922	0.07237	rs146004738	3.213	rs12995875	0.02857	-15.54	1.966	104	104	enet	0.078	5.5E-129	-1.6129	0.107
SeqId_8327_26	DPEP2	16	68021297	68034489	0.35112	rs187241218	2.33	rs9928014	0.02773	-14.35	-1.232	193	193	enet	0.077	7.4E-128	1.6057	0.108
SeqId_18225_13	HEBP1	12	13127804	13153199	0.23558	rs11055116	2.149	rs11055175	0.168904	-34.98	1.546	94	94	enet	0.25	0	-1.60571	0.10834
SeqId_3314_74	GFRA1	10	117816436	118036315	0.0641	rs7096877	-1.85	rs11197582	0.046093	18.49	-1.538	33	33	enet	0.053	8.5E-88	-1.60326	0.1089
SeqId_7179_69	NFASC	1	204797779	204991950	0.18841	rs12123647	-2.753	rs6663324	0.129	30.58	0.607	70	70	enet	0.17	2.7E-303	1.5992	0.109769
SeqId_14133_93	IL1R2	2	102608422	102645006	0.14928	rs77403630	2.781	rs2310170	0.12675	-30.4	-1.919	57	57	enet	0.14	8.6E-235	1.596	0.11
SeqId_5346_24	CPNE1	20	34213953	34252878	0.45423	rs73104746	4.3	rs113522583	0.327509	-48.66	0.898	51	51	enet	0.43	0	-1.5953	0.11064

SeqId_2973_15	CD36	7	79998891	80308593	0.0147	rs62464490	-2.333	rs11760281	0.000675	-4.99	-0.4207	51	51	enet	0.0037	0.00000013	1.5948	0.110766
SeqId_13062_4	GMFG	19	39818993	39833012	0.03906	rs1017079	2.598	rs30459	0.022029	-13.11	-1.1446	43	43	enet	0.024	1.7E-40	1.59459	0.110805
SeqId_3235_50	WFIKKN2	17	48912011	48919714	0.15154	rs79839670	2.392	rs11079936	0.218661	-39.74	-1.605	50	50	enet	0.23	0	1.5914	0.11153
SeqId_9849_13	APEX1	14	20923350	20925931	0.05044	rs72672714	-3.276	rs1130409	0.020134	12.21	-1.476	27	27	enet	0.032	6E-53	-1.5889	0.112
SeqId_9744_139	DNAJA4	15	78556428	78574538	0.0062	rs61752771	-1.492	rs11639191	0.00405	5.81	0.346	23	23	enet	0.0047	0.000000003	1.5891	0.112
SeqId_6620_82	LINGO1	15	77905369	78113242	0.02161	rs143893273	3.228	rs62007781	0.00552	7.99	-0.492	68	68	enet	0.014	1.2E-24	-1.5866	0.1126
SeqId_19267_14	HAGH	16	1845621	1877158	0.13001	rs2241071	-3.34	rs116869551	0.07462	23.32	1.04	77	77	enet	0.12	3.4E-197	1.586	0.113
SeqId_3518_54	CPB2	13	46627321	46679168	0.27143	rs4942450	-2.97	rs7988836	0.25895	43.24	-1.692	325	325	enet	0.33	0	-1.581	0.114
SeqId_17391_10	RBM17	10	6130997	6159420	0.05504	rs661985	2.42	rs11256726	0.037729	-17	1.565	37	37	enet	0.038	9.1E-63	-1.579275	0.1143
SeqId_12399_194	CCDC50	3	191046874	191116448	0.01631	rs146170086	-2.35	rs147604673	0.00938	-8.6	-1.575	48	1	top1	0.0094	1E-16	1.575	0.115
SeqId_4467_49	SPARCL1	4	88394487	88452213	0.11936	rs1965907	-2.11	rs7688567	0.181218	37.26	-1.966	81	81	enet	0.23	0	-1.5779	0.115
SeqId_5129_12	SCARF1	17	1537152	1549038	0.40386	rs8076897	4.118	rs2272011	0.332784	-49.01	-0.995	81	81	enet	0.41	0	1.5744	0.1154
SeqId_15480_2	VNN2	6	133065009	133084598	0.39772	rs117715244	-2.75	rs9493425	0.24884	-42.37	-0.528	86	86	enet	0.33	0	1.573	0.116
SeqId_2900_53	CCL14	17	34310324	34314040	0.24117	rs16971802	2.429	rs7222922	0.220023	-39.86	1.93	61	61	enet	0.27	0	-1.5714	0.1161
SeqId_12517_52	PDCD5	19	33072096	33078359	0.2287	rs111413644	2.181	rs4499344	0.212807	39.2	-1.436	27	27	enet	0.27	0	-1.57076	0.116238
SeqId_13242_134	SHANK3	22	51112843	51171726	0.09251	rs9616810	2.43	rs6009946	0.06285	21.34	1.922	25	25	enet	0.091	2.1E-151	1.5693	0.11657
SeqId_4763_31	AFM	4	74347462	74369718	0.06431	rs1894292	-2.202	rs72856662	0.05276	-19.79	1.488	70	70	enet	0.067	3.3E-110	-1.568	0.117
SeqId_17161_1	DDOST	1	20978260	20988037	0.03205	rs150976169	2.493	rs150466875	0.0195	-12.12	-0.996	42	42	enet	0.026	3.1E-44	1.5619	0.1183
SeqId_7808_5	GLCE	15	69452923	69564556	0.16687	rs12148883	-3.014	rs3865014	0.24089	41.79	1.097	88	88	enet	0.25	0	1.5607	0.1186
SeqId_7050_5	NEGR1	1	71861626	72748222	0.04195	rs17516887	-1.937	rs12029938	0.00522	-6.86	-0.04	42	42	enet	0.015	3.9E-26	1.5606	0.118616
SeqId_5636_10	MFAP4	17	19286755	19290503	0.1017	rs1110466	2.115	rs528684420	0.024167	-13.33	-0.8942	29	29	enet	0.031	1.1E-51	1.5573	0.11939
SeqId_9297_12	B3GNT8	19	41931264	41934635	0.15593	rs111403414	-2.279	rs284663	0.331504	48.93	-1.715	30	30	enet	0.34	0	-1.55688	0.119499
SeqId_18881_7	CD97	19	14491313	14519537	0.02348	rs185882225	1.879	rs3786656	0.00351	6.68	-0.7518	56	56	enet	0.011	8.5E-20	-1.5568	0.119518
SeqId_8024_64	TPST2	22	26917962	26992681	0.05591	rs143557022	2.04	rs4275	0.03386	15.87	-1.222	37	37	enet	0.052	8.9E-86	-1.5544	0.12009
SeqId_13132_14	SEMA5A	5	9035145	9546187	0.2794	rs139755478	-2.355	rs56015217	0.145	32.48	0.661	117	117	enet	0.24	0	1.5531	0.1204
SeqId_18397_5	AKR1C4	10	5237425	5260912	0.11617	rs117292237	1.8	rs75811842	0.289145	-45.68	1.48	43	43	enet	0.29	0	-1.551352	0.1208
SeqId_15583_18	FCRLB	1	161691353	161697933	0.099	rs147715895	2.171	rs61801163	0.0479	18.76	1.014	70	70	enet	0.091	2.5E-152	1.549	0.121373
SeqId_12560_9	NT5C	17	73126320	73127852	0.17685	rs138891334	2.473	rs78625720	0.117473	-29.24	-1.822	67	67	enet	0.13	1.1E-224	1.5444	0.12248
SeqId_14151_4	ISG15	1	936518	949920	0.24089	rs4970350	-2.15	rs3121574	0.0378	17.82	1.362	15	15	enet	0.077	7.6E-128	1.5325	0.125397
SeqId_2620_4	IL6ST	5	55230923	55290850	0.10113	rs79297018	2.049	rs13183065	0.0891	25.42	1.575	61	61	enet	0.099	4E-166	1.52739	0.12666
SeqId_5139_32	UNC5C	4	96083655	96470357	0.07867	rs6532537	-1.949	rs1994292	0.044631	18.01	-1.163	78	78	enet	0.082	1.4E-135	-1.5256	0.127
SeqId_3171_57	APP	21	27252861	27543446	0.02284	rs172592	-2.31	rs439826	0.01192	-9.64	1.31	34	34	enet	0.019	6.9E-32	-1.5222	0.128
SeqId_4549_78	FUT5	19	5865837	5870551	0.26913	rs61180947	2.144	rs778809	0.113488	-28.73	-1.177	92	92	enet	0.27	0	1.52189	0.128035
SeqId_2201_17	COL18A1	21	46825080	46933634	0.19676	rs112225010	3.58	rs144147445	0.04222	-17.56	1.07	110	110	enet	0.11	2.2E-176	-1.5181	0.129
SeqId_4155_3	TNC	9	117781854	117880536	0.52315	rs4145474	2.171	rs1138545	0.36783	51.52	-1.794	77	77	enet	0.4	0	-1.5188	0.129
SeqId_3600_2	CHIT1	1	203181955	203242769	0.33584	rs150205774	2.237	rs872583	0.313	-47.57	1.245	62	62	enet	0.38	0	-1.5158	0.12957
SeqId_3290_50	CD109	6	74405508	74538040	0.34542	rs139847894	2.1	rs6925924	0.26694	43.96	1.481	179	179	enet	0.34	0	1.513	0.13
SeqId_10561_5	PGLYRP3	1	153269592	153285428	0.05244	rs55991125	2.109	rs55991125	0.0147	-10.53	2.109	35	35	enet	0.024	7E-40	-1.51	0.131051
SeqId_15604_18	MAPK9	5	179660143	179719099	0.04492	rs116301830	2.671	rs11741142	0.0372	16.47	1.043	41	41	enet	0.046	3.7E-76	1.50894	0.13131
SeqId_15336_7	SELM	22	31500758	31516055	0.01278	rs118006664	-1.8	rs117551362	0.00542	7.38	-1.206	11	11	enet	0.0065	3.9E-12	-1.5079	0.13159
SeqId_7060_2	ERO1L	14	53106633	53162618	0.00421	rs112898150	2.196	rs112898150	0.000838	4.71	2.196	7	7	enet	0.001	0.0037	1.5011	0.133
SeqId_7083_74	MATN4	20	43922085	43937169	0.11691	rs6104018	2.64	rs11906694	0.047275	-18.55	1.789	87	87	enet	0.084	5.1E-140	-1.5021	0.13308
SeqId_11354_21	PLCB1	20	8057898	8949007	0.00434	rs6039069	1.56	rs1033398	0.000245	3.95	1.498	8	1	top1	0.00024	0.096	1.498	0.13413
SeqId_6393_63	HSP90B1	12	104323885	104347709	0.26901	rs7296626	4.076	rs2722192	0.479947	58.9	2.029	85	85	enet	0.51	0	1.49512	0.13488
SeqId_15530_33	EPHB4	7	100400187	100425145	0.0343	rs2553021	1.938	rs314346	0.03065	15.34	1.222	27	27	enet	0.033	4.5E-54	1.4918	0.13576
SeqId_3710_49	PLG	6	161123270	161175129	0.54602	rs41267809	-3.14	rs537579467	0.07847	24.02	-0.2486	166	166	enet	0.23	0	-1.492	0.136
SeqId_11187_11	CLEC12A	12	10103915	10148293	0.3778	rs1797526	2.318	rs1060648	0.529498	-61.83	1.255	52	52	enet	0.55	0	-1.48752	0.13688
SeqId_7841_84	ESAM	11	124622479	124632186	0.0889	rs77328902	1.821	rs12541	0.053816	-19.89	1.593	33	33	enet	0.068	4.7E-112	-1.48759	0.137
SeqId_3431_54	EPHA1	7	143087382	143105949	0.34127	rs1135411	-2.248	rs4421280	0.288917	45.66	1.866	61	61	enet	0.32	0	1.4853	0.137453
SeqId_5069_9	CD55	1	207494877	207560149	0.1014	rs76588278	-2.676	rs1583090	0.235	-41.23	-1.52	66	66	enet	0.24	0	1.4833	0.137994
SeqId_7019_13	SEMA7A	15	74701630	74726299	0.01788	rs147708965	1.951	rs78994380	0.00591	-6.89	-0.988	38	38	enet	0.011	5E-19	1.4808	0.1387
SeqId_7218_87	ATP1B2	17	7549945	7561088	0.11194	rs858518	2.468	rs1642762	0.089559	-25.64	2.0008	47	47	enet	0.14	5.5E-245	-1.4803	0.13881
SeqId_13405_61	SPINK2	4	57676026	57687908	0.0729	rs148321523	1.768	rs7694521	0.016979	13.06	-1.272	37	37	enet	0.054	1E-89	-1.48	0.139

SeqId_14123_34	C10orf54	10	73507313	73533277	0.12251	rs75071136	2.69	rs12415873	0.090054	25.59	0.924	69	69	enet	0.11	2.4E-186	1.477033	0.1397
SeqId_15509_2	NAGLU	17	40688259	40696467	0.17773	rs677960	-2.238	rs41283429	0.096909	26.5	1.509	116	116	enet	0.12	8.3E-211	1.4749	0.14023
SeqId_5542_22	NRP1	10	33466420	33625190	0.0841	rs527870997	-2.37	rs734186	0.072527	-22.9	-1.931	84	84	enet	0.094	1.4E-157	1.467715	0.1422
SeqId_9394_19	CPQ	8	97657470	98161882	0.24923	rs142538426	-2.359	rs148684432	0.073058	-23.01	1.1569	81	81	enet	0.19	0	-1.465	0.14291
SeqId_5451_1	ALCAM	3	105085753	105295744	0.06316	rs115498068	3.66	rs751554	0.0227	-13.42	-1.457	97	97	enet	0.049	1.1E-80	1.46321	0.143
SeqId_19361_78	MATN3	2	20191813	20212429	0.44353	rs143517494	2.515	rs11694716	0.25901	-43.25	1.381	100	100	enet	0.4	0	-1.4564	0.145
SeqId_12475_48	CLIC5	6	45848564	46048085	0.26546	rs6924107	2.91	rs35822882	0.07306	-23.02	-1.117	75	75	enet	0.088	2.3E-147	1.457	0.145
SeqId_3316_58	SERPIND1	22	21128401	21142008	0.0097	rs5749800	-2.62	rs73160986	0.00408	5.72	1.457	9	1	top1	0.0041	0.000000034	1.457	0.14512
SeqId_4407_10	MST1	3	49721380	49726934	0.43104	rs111744323	-3.1	rs3197999	0.444	-56.68	-1.453	73	73	enet	0.52	0	1.45261	0.146
SeqId_12859_33	ECI2	6	4115927	4135831	0.0645	rs6597031	1.58	rs7757606	0.08395	-24.81	1.454	36	1	top1	0.084	9.9E-140	-1.454	0.146
SeqId_11480_1	ALDH3A1	17	19641297	19652256	0.03054	rs1106374	-2.956	rs887241	0.021355	12.46	-1.312	12	12	enet	0.022	4.3E-37	-1.4526	0.14634
SeqId_15453_3	AMBP	9	116822407	116840608	0.01706	rs2636881	-2.02	rs10982050	0.00813	-7.82	-0.335	35	35	enet	0.013	1.6E-22	1.4499	0.147
SeqId_6433_57	FAM20A	17	66531254	66597508	0.05851	rs76321556	4.364	rs12453086	0.027535	-14.24	-1.548	82	82	enet	0.046	1.4E-75	1.4475	0.14777
SeqId_8969_49	CD14	5	140011313	140013286	0.07745	rs2337225	-3.104	rs5744441	0.0346	-16.04	0.216	59	59	enet	0.047	2E-77	-1.44428	0.14866
SeqId_4878_3	F10	13	113777113	113803843	0.1322	rs3211752	2.84	rs3211757	0.03809	16.75	0.829	48	48	enet	0.074	1.6E-123	1.44	0.1499
SeqId_8245_27	ICAM5	19	10400628	10407454	0.28356	rs8113381	-4.788	rs281440	0.218619	39.75	-2.495	58	58	enet	0.28	0	-1.43877	0.150216
SeqId_15558_63	ENPEP	4	111286889	111486441	0.32466	rs33966350	-3.536	rs33966350	0.088416	-25.32	-3.536	95	95	enet	0.23	0	1.4346	0.151
SeqId_3132_1	VEGFC	4	177604689	177714076	0.04985	rs41278571	1.906	rs41278571	0.024438	13.36	1.906	12	12	enet	0.028	4.1E-46	1.4346	0.151
SeqId_17794_6	PMM2	16	8882680	8956391	0.04325	rs3826198	2.3	rs34258285	0.023	-13.17	0.445	24	24	enet	0.035	1.2E-57	-1.4319	0.152
SeqId_13122_19	FLRT2	14	85996488	86120772	0.11287	rs11849838	2.09	rs17796783	0.118124	-29.24	1.331	126	126	enet	0.17	2.9E-297	-1.4178	0.156
SeqId_16594_44	FAIM	3	138327448	138352218	0.35969	rs361058	-2.54	rs641320	0.0684	-22.26	1.199	14	14	enet	0.1	2.9E-174	-1.42001	0.156
SeqId_13381_49	B4GALT1	9	33104080	33167354	0.133	rs140867747	2.75	rs7019909	0.07296	22.97	2.108	59	59	enet	0.094	1.9E-156	1.4177	0.156
SeqId_2946_52	CFD	19	859664	863641	0.10173	rs111607343	-2.214	rs72984031	0.014672	-10.38	0.6033	53	53	enet	0.044	1.2E-72	-1.41368	0.157455
SeqId_6984_6	IGSF8	1	160061130	160068733	0.03038	rs12092798	-2.313	rs12408242	0.0285	14.47	-2.188	18	18	enet	0.031	2.5E-52	-1.4085	0.158997
SeqId_3313_21	FCN2	9	137772656	137779369	0.1694	rs3124956	2.451	rs3124956	0.10512	-27.56	2.451	60	60	enet	0.19	0	-1.4079	0.159
SeqId_13109_82	NEGR1	1	71861626	72748222	0.08639	rs7524126	1.48	rs11209871	0.0129	-9.96	-0.051	40	40	enet	0.028	3E-46	1.4049	0.160043
SeqId_16857_2	RAB2A	8	61429495	61536203	0.00216	rs72652568	-1.526	rs11995335	0.000111	3.86	1.403	13	1	top1	0.00011	0.18	1.403	0.160617
SeqId_2789_26	MMP7	11	102391239	102401481	0.20084	rs140411048	2.09	rs11568819	0.074558	23.22	0.886	87	87	enet	0.15	7E-248	1.40045	0.161
SeqId_19561_216	PLXND1	3	129274057	129325519	0.18218	rs139906131	2.16	rs1108584	0.0497	20.5	-0.526	86	86	enet	0.13	1.2E-212	-1.4013	0.161
SeqId_12612_37	PSMB1	6	170844208	170862395	0.06469	rs9295415	1.55	rs3734763	0.13249	-31.05	-1.383	26	26	enet	0.13	3.6E-228	1.394	0.163
SeqId_18315_38	RTP4	3	187086173	187089864	0.13668	rs17719193	-2.96	rs1878861	0.125	-30.09	1.438	91	91	enet	0.13	7.5E-220	-1.39163	0.164
SeqId_5462_62	FCN3	1	27695601	27701315	0.09392	rs58337722	2.428	rs111257457	0.0497	-19.12	1.068	97	97	enet	0.076	1.8E-126	-1.3908	0.164275
SeqId_8275_31	PEAR1	1	156863490	156886221	0.14601	rs4414033	-1.993	rs12041331	0.0459	-18.25	1.06	56	56	enet	0.093	6.4E-155	-1.3901	0.164495
SeqId_18398_1	AKR1D1	7	137687070	137803051	0.01342	rs28634434	-2.158	rs9642091	0.010617	9.07	1.3895	30	1	top1	0.011	1E-18	1.3895	0.164689
SeqId_18831_6	LRIG1	3	66429221	66551687	0.20682	rs9856474	2.19	rs2306272	0.228	-40.57	1.429	83	83	enet	0.23	0	-1.38555	0.166
SeqId_6629_3	DEFB1	8	6728114	6735458	0.09985	rs2741674	-2.222	rs2741117	0.085581	-25.01	1.6382	67	67	enet	0.11	8.7E-182	-1.3838	0.166406
SeqId_6544_33	NELL1	11	20691097	21597232	0.16197	rs2293241	-2.601	rs8176786	0.108537	28.12	1.307	39	39	enet	0.12	2.1E-200	1.38322	0.167
SeqId_15570_99	CR2	1	207627575	207663240	0.04287	rs61821043	-1.871	rs61821130	0.0093	-8.97	0.487	73	73	enet	0.025	1.5E-42	-1.3806	0.167396
SeqId_8840_61	C1S	12	7096351	7178336	0.31727	rs77365023	1.849	rs73044256	0.248374	2.64	0.438	14	14	enet	0.29	0	-1.38031	0.16749
SeqId_6448_36	SEMA3C	7	80371854	80551675	0.04045	rs62467052	1.783	rs1019016	0.011798	10.58	-1.188	53	53	enet	0.029	1.3E-48	-1.3798	0.167638
SeqId_9580_5	LAMC2	1	183155373	183214262	0.36338	rs3965889	-2.352	rs2276543	0.215	39.37	1.189	57	57	enet	0.24	0	1.3797	0.167685
SeqId_16607_78	GSN	9	123970072	124095121	0.01103	rs116983920	-3.821	rs10985196	0.00782	-7.84	1.377	30	1	top1	0.0078	3.2E-14	-1.377	0.169
SeqId_18876_77	CHST4	16	71559136	71572651	0.04806	rs75541566	2.31	rs16973264	0.01695	11.22	-1.734	27	27	enet	0.019	2.8E-32	-1.3728	0.17
SeqId_18882_7	CLSTN2	3	139654027	140296239	0.12319	rs982074	-2.96	rs9863263	0.0383	-16.85	-0.598	98	98	enet	0.12	1.4E-202	1.37016	0.171
SeqId_13094_75	RSPO3	6	127439816	127520626	0.08081	rs7766106	1.52	rs2154167	0.0982	26.98	0.899	45	45	enet	0.11	2.8E-180	1.368	0.171
SeqId_10514_5	PTGDS	9	139869544	139876194	0.03394	rs925936	-2.308	rs11145948	0.03471	15.88	-1.37	26	1	top1	0.035	1.5E-57	-1.37	0.171
SeqId_17396_23	ADH1A	4	100197526	100212160	0.12179	rs10011147	2.116	rs28364331	0.056308	-20.3	-0.967	34	34	enet	0.065	6E-107	1.3649	0.172
SeqId_15376_134	CTSE	1	NA	206317459	0.0254	rs113012212	-1.989	rs78554767	0.000566	4.5	0.44	49	49	enet	0.0051	8E-10	-1.3633	0.172801
SeqId_17515_6	HSPA13	21	15743436	15755805	0.0312	rs2822638	1.35	rs2822638	0.02628	-13.81	1.355	12	1	top1	0.026	7.1E-44	-1.355	0.175
SeqId_15686_49	INHBC	12	57828567	57845845	0.22299	rs73121617	-1.511	rs2229357	0.49134	-59.54	1.261	35	35	enet	0.49	0	-1.3549	0.17545
SeqId_5248_68	PPIF	10	81107225	81115090	0.01472	rs118016429	1.89	rs79510127	0.005743	-7	-0.748	22	22	enet	0.0084	3.9E-15	1.353441	0.1759
SeqId_4158_54	PLAU	10	75668935	75677254	0.26072	rs72816389	3.32	rs2633322	0.062128	-21.91	-1.563	87	87	enet	0.11	2.6E-176	1.353368	0.1759
SeqId_9986_14	NPW	16	2059927	2070756	0.11251	rs78468043	-3.58	rs111276210	0.00838	-9.04	0.462	63	63	enet	0.029	1.4E-47	-1.354	0.176

SeqId_18841_1	SERPINB13	18	61254223	61271873	0.03032	rs75137858	2.77	rs77157727	0.01306	-10.29	-1.73	33	33	enet	0.015	4.5E-25	1.352	0.1763
SeqId_6521_35	NPTX2	7	98246597	98259181	0.06153	rs11762534	2.408	rs12673464	0.037783	16.56	-0.805	56	56	enet	0.053	1.5E-87	-1.3515	0.176526
SeqId_5637_81	NTNG1	1	107682629	108027545	0.18175	rs3813221	2.447	rs76374975	0.147	32.64	1.092	39	39	enet	0.16	4.4E-276	1.351	0.176698
SeqId_15364_101	APOC1	19	45417504	45422606	0.06832	rs6509208	3.826	rs8106813	0.02889	15.45	1.6711	52	52	enet	0.043	2.3E-71	1.34978	0.177086
SeqId_12508_9	VPS24	2	86730553	86790602	0.02648	rs2044232	-1.818	rs59613942	0.02334	13.25	-1.597	37	37	enet	0.03	1.5E-50	-1.3466	0.178
SeqId_7145_1	ITIH3	3	52828784	52843025	0.37047	rs62253654	-2.54	rs2535629	0.211	39.05	-2.019	51	51	enet	0.29	0	-1.34425	0.179
SeqId_3000_66	MBL2	10	54525140	54532544	0.5289	rs150533883	2.37	rs7899547	0.273513	44.43	-0.454	65	65	enet	0.45	0	-1.343022	0.1793
SeqId_15486_126	AOC1	7	150521715	150558592	0.4716	rs1049742	-3.162	rs10452848	0.288496	45.64	0.286	172	172	enet	0.44	0	1.3415	0.179766
SeqId_11969_5	S100A2	1	153533584	153540366	0.01352	rs116793967	-1.341	rs58056804	0.00869	-8.32	-1.202	16	16	enet	0.0095	7.7E-17	1.341	0.179936
SeqId_3554_24	ADIPOQ	3	186560493	186576252	0.11006	rs5030068	2.95	rs143257534	0.0358	-17.47	-1.95	43	43	enet	0.11	3.3E-176	1.34082	0.18
SeqId_14271_23	RAB6B	3	133543079	133614726	0.11873	rs2715633	2.18	rs9813363	0.125	-30.29	1.266	26	26	enet	0.14	1.5E-231	-1.33794	0.181
SeqId_11696_7	CRABP2	1	156669398	156675608	0.21736	rs61813461	-1.867	rs3806412	0.142	32	-1.421	15	15	enet	0.15	3.4E-263	-1.3335	0.182369
SeqId_3326_58	CADM1	11	115039938	115375675	0.0386	rs150039549	-2.939	rs17564430	0.031743	15.35	-0.873	54	54	enet	0.039	2.9E-65	-1.33287	0.183
SeqId_17460_51	MX1	21	42791947	42841998	0.08458	rs8133682	1.56	rs464783	0.08091	-24.27	1.385	38	38	enet	0.1	2E-175	-1.3305	0.183
SeqId_2436_49	CXCL16	17	4636828	4643223	0.03332	rs11658971	2.479	rs7214635	0.014744	11.13	0.214	36	36	enet	0.034	1.8E-56	1.3291	0.18382
SeqId_18830_1	ITLN1	1	160846330	160854970	0.00918	rs191629893	2.017	rs61803286	0.00341	5.84	0.408	39	39	enet	0.0037	0.00000013	-1.3289	0.183885
SeqId_7856_51	FAM151A	1	55074854	55089229	0.11698	rs10493176	2.615	rs11206398	0.0408	17.31	0.328	143	143	enet	0.083	8.8E-138	-1.3267	0.184602
SeqId_2855_49	MAPK3	16	30125426	30134827	0.08398	rs11865086	1.88	rs9932466	0.17198	35.6	0.931	37	37	enet	0.21	0	1.323	0.186
SeqId_6379_62	ADAMTSL2	9	136397286	136440641	0.02161	rs11244064	4.983	rs10120207	0.00162	-6	-0.097	59	59	enet	0.015	2.2E-25	1.3231	0.186
SeqId_13460_4	CHAD	17	48541857	48546267	0.0134	rs79575045	2.098	rs184613584	0.007647	-7.65	0.326	17	17	enet	0.008	1.5E-14	-1.3198	0.18691
SeqId_5005_4	MAPK12	22	50683879	50700145	0.0347	rs9617126	3.1	rs2272855	0.01889	-11.81	2.003	62	62	enet	0.024	2.5E-40	-1.3095	0.19035
SeqId_9018_38	PCDH10	4	134070449	134129761	0.05546	rs2063156	2.494	rs4864200	0.02446	-13.89	-0.47	86	86	enet	0.06	4.4E-100	-1.309	0.191
SeqId_6920_1	GFRAL	6	55192267	55267291	0.09626	rs4523089	-2.98	rs73439277	0.01379	12.13	-0.755	127	127	enet	0.069	7E-115	-1.309	0.191
SeqId_9754_33	NQO2	6	2988221	3019989	0.43602	rs144691990	2.4	rs9378755	0.18041	-35.35	-1.8622	95	95	enet	0.41	0	1.306	0.191
SeqId_2381_52	C5	9	123714613	123812553	0.09977	rs13290556	2.427	rs1035029	0.02449	13.38	0.104	35	35	enet	0.067	1.8E-111	1.3066	0.191
SeqId_8351_17	PRSS57	19	685546	695498	0.14704	rs112578597	-1.825	rs2301741	0.055174	20.01	1.747	39	39	enet	0.061	4.7E-101	1.30596	0.191566
SeqId_13666_222	CA10	17	49707674	50237377	0.09418	rs12449528	-2.226	rs117399000	0.03123	-15.06	0.559	43	43	enet	0.063	9.5E-104	-1.3001	0.19356
SeqId_11531_24	C10orf54	10	73507313	73533277	0.12444	rs117140087	-2.31	rs10762476	0.082794	24.49	0.999	26	26	enet	0.11	1.4E-189	1.297572	0.1944
SeqId_9468_8	LMAN2	5	176758568	176778669	0.05878	rs10056854	-2.251	rs35582636	0.00311	6.65	1.196	61	61	enet	0.021	1.4E-35	1.29639	0.19484
SeqId_13133_73	LTBP4	19	41098789	41135723	0.03877	rs118163583	-3.564	rs34093919	0.014975	-10.55	-0.946	54	54	enet	0.02	3E-33	-1.29494	0.195341
SeqId_6415_90	CPN2	3	194060497	194072057	0.31589	rs7647309	1.7	rs11711157	0.229	-40.65	1.128	65	65	enet	0.29	0	-1.29169	0.196
SeqId_5089_11	IL7R	5	35852797	35879705	0.00915	rs114880217	2.175	rs10058572	0.00975	-8.75	1.29	16	1	top1	0.0098	2.6E-17	-1.2903	0.19695
SeqId_7810_20	C1QTNF5	11	119209652	119211650	0.04326	rs2248863	-1.275	rs2509656	0.013078	10.8	-1.125	9	9	enet	0.031	1.2E-51	-1.28909	0.197
SeqId_13124_20	ISLR2	15	74392652	74430881	0.09191	rs117625306	-2.32	rs11632698	0.02926	-16.37	0.34	64	64	enet	0.1	1.4E-171	-1.2895	0.1972
SeqId_10627_87	APLP2	11	129938042	130014706	0.01974	rs3808986	-1.43	rs61349706	0.014538	11.13	-1.103	32	32	enet	0.015	1.8E-26	-1.28751	0.198
SeqId_5632_6	CRTAC1	10	99624757	99790585	0.25116	rs146472753	-2.8	rs684225	0.06637	-21.95	-1.764	113	113	enet	0.11	2.1E-190	1.284949	0.1988
SeqId_2813_11	AGRP	16	67516474	67517450	0.05001	rs74900413	2.93	rs114322795	0.03163	15.41	-1.111	28	28	enet	0.045	8.9E-75	-1.2824	0.2
SeqId_4979_34	DPT	1	168664706	168698444	0.16255	rs111307211	4.555	rs1018454	0.0913	25.7	-2.816	121	121	enet	0.16	2.7E-282	-1.2808	0.200277
SeqId_4309_59	TPI1	12	6976283	6980112	0.03596	rs66621175	-1.276	rs66621175	0.051657	-18.47	-1.276	3	1	top1	0.052	2.2E-85	1.276	0.20196
SeqId_2580_83	MPO	17	56347216	56358296	0.12069	rs6503841	2.628	rs75394768	0.029912	-15.37	-0.916	50	50	enet	0.077	4.6E-127	1.2751	0.20229
SeqId_13988_67	NMRAL1	16	4511692	4545764	0.07722	rs2386888	1.94	rs11557236	0.04037	-17.13	-1.314	48	48	enet	0.043	1.6E-70	1.2727	0.203
SeqId_9525_1	PTK7	6	43044045	43129457	0.0594	rs142656572	2.65	rs140927825	0.02079	12.41	0.313	124	124	enet	0.033	2.7E-54	1.274	0.203
SeqId_13093_6	SECTM1	17	80278900	80291950	0.08418	rs73999892	1.718	rs4789684	0.059963	20.87	-0.5825	45	45	enet	0.086	4.6E-143	-1.273	0.20302
SeqId_12569_25	CCT5	5	10250041	10266501	0.00744	rs116470800	1.632	rs3844305	0.00717	-7.44	-1.27	27	1	top1	0.0072	3.5E-13	1.27	0.20408
SeqId_2742_68	SIGLEC7	19	51645556	51656783	0.25818	rs143914429	3.156	rs12983058	0.049431	18.92	-1.077	103	103	enet	0.12	2E-206	-1.26936	0.204313
SeqId_13720_95	PRTN3	19	840999	848175	0.03426	rs1060442	1.877	rs1060442	0.005011	-8.06	1.877	28	28	enet	0.018	4.1E-30	-1.26913	0.204394
SeqId_9715_15	IGSF3	1	117117021	117210377	0.06043	rs6658522	-2.997	rs655735	0.0203	-12.19	1.844	79	79	enet	0.037	1.1E-61	-1.2657	0.205627
SeqId_13697_51	GPD1	12	50497765	50505096	0.04932	rs77546610	3.144	rs75732302	0.003378	-6.28	0.42	121	121	enet	0.011	2.2E-19	-1.26478	0.20595
SeqId_2994_71	IL1RL2	2	102803466	102856462	0.11557	rs1997504	-2.831	rs1960510	0.05147	-19.8	1.504	59	59	enet	0.099	1.9E-165	1.2649	0.206
SeqId_15511_37	NPTXR	22	39214457	39240033	0.14449	rs117773903	-2.6	rs4821839	0.09348	26.21	-2.401	89	89	enet	0.12	2.9E-207	-1.258	0.20838
SeqId_4546_27	ADGRE2	19	14843204	14889372	0.38598	rs116943668	2.415	rs67023356	0.085624	24.9	1.677	107	107	enet	0.16	1.5E-281	1.25554	0.209281
SeqId_13682_47	CSF1R	5	149432854	149492935	0.10384	rs17600407	-2.412	rs572474264	0.021	-12.51	-0.098	56	56	enet	0.055	1E-90	1.25095	0.21095
SeqId_11278_4	COL11A2	6	33130458	33160276	0.21099	rs6903433	-3.7	rs3129205	0.0647	21.99	-0.443	75	75	enet	0.16	2.2E-282	-1.247	0.212

SeqId_3220_40	RET	10	43572512	43625799	0.10968	rs3004258	2.81	rs2506008	0.097101	26.57	-1.737	116	116	enet	0.15	3.4E-250	-1.241139	0.2146
SeqId_19504_22	DTYMK	2	242615162	242626359	0.12837	rs79349366	1.914	rs142497237	0.02931	14.71	-1.161	63	63	enet	0.071	1.4E-117	-1.2369	0.216
SeqId_17345_12	ZADH2	18	72907068	72921094	0.01927	rs12969004	-1.32	rs12969004	0.01097	-9.78	-1.321	3	3	enet	0.012	2.8E-21	1.237	0.2161
SeqId_3066_12	LGALS3	14	55590828	55612141	0.05297	rs79531292	-2.136	rs118083722	0.103074	-27.48	-2.002	48	48	enet	0.12	1.3E-209	1.2309	0.218
SeqId_4962_52	CDNF	10	14861244	14880574	0.14044	rs142562132	2.09	rs188293941	0.017523	-12.66	0.3173	118	118	enet	0.068	2.60E-112	-1.229098	0.219
SeqId_7861_9	ROR2	9	94325373	94712510	0.12891	rs9409462	-2.399	rs10118816	0.04988	19.16	-2.047	59	59	enet	0.12	7.30E-203	-1.2237	2.21E-01
SeqId_4960_72	ANXA1	9	75766781	75785309	0.03397	rs7018979	2.081	rs2795108	0.0122	10.29	1.616	29	29	enet	0.021	6.80E-35	1.2221	2.22E-01
SeqId_4154_57	SELP	1	169558087	169599431	0.3708	rs114137203	9.137	rs6136	8.02E-02	-24.12	-1.324	34	34	enet	0.12	1.60E-204	1.2211	0.222065
SeqId_7933_75	ADAM22	7	87563458	87832204	0.16205	rs149385940	2.658	rs12670575	0.099121	-26.83	1.128	84	84	enet	0.15	7.40E-259	-1.2161	0.223962
SeqId_5085_18	IL20RA	6	137321108	137366317	0.01758	rs78994374	2.61	rs1775296	0.00594	-6.81	-1.259	45	45	enet	0.0098	2.40E-17	1.217	2.24E-01
SeqId_9484_75	DSG2	18	29078140	29128971	0.12695	rs72929966	-2.48	rs1460602	0.05727	-20.58	0.638	91	91	enet	0.1	1.40E-166	-1.212	0.2255
SeqId_8427_118	RSPO3	6	127439816	127520626	0.08413	rs62437084	-2.12	rs1892172	0.12358	29.94	0.915	49	49	enet	0.13	1.60E-229	1.206	2.28E-01
SeqId_17766_5	NCF1	7	74188309	74203720	0.28903	rs62476375	2.701	rs148581667	0.094297	20.11	0.8245	128	128	enet	0.17	2.80E-289	1.2029	0.229031
SeqId_2571_12	IGFBP3	7	45951844	45961473	0.12873	rs1872584	-2.775	rs148562589	0.038664	16.99	0.653	48	48	enet	0.061	3.50E-100	1.2025	0.229175
SeqId_2658_27	NTRK3	15	88402982	88799999	0.08887	rs7180011	-2.743	rs28714295	0.04508	18.08	-0.003	61	61	enet	0.074	1.50E-122	1.2006	0.2299
SeqId_4996_66	HRG	3	186378005	186396023	0.31933	rs112096058	2.94	rs60531302	2.77E-01	43.88	-0.435	79	79	enet	0.36	0	-1.20107	2.30E-01
SeqId_12652_37	AHCYL1	1	110527387	110566364	0.00916	rs2274568	1.914	rs7517624	9.52E-04	5.07	-0.704	24	24	enet	0.0034	3.70E-07	-1.1991	0.230473
SeqId_3299_29	CNTN5	11	98891679	100229616	0.13486	rs1663423	-2.34	rs4528296	0.069459	-22.78	-0.723	56	56	enet	0.12	2.70E-210	1.19813	2.31E-01
SeqId_17694_32	PSME2	14	24612571	24616779	0.06574	rs137918735	-1.863	rs2236352	0.035439	16.46	-1.524	28	28	enet	0.044	2.70E-72	-1.1987	2.31E-01
SeqId_2977_7	EDAR	2	109510927	109605828	0.14637	rs62152245	2.388	rs75147553	0.061	20.99	1.866	63	63	enet	0.11	2.60E-193	1.1983	2.31E-01
SeqId_5076_53	EPHA10	1	38179552	38230805	0.02052	rs1809328	1.986	rs12074120	7.85E-03	8.57	-0.432	43	43	enet	0.015	2.10E-26	-1.1968	0.23139
SeqId_17156_72	DCLK1	13	36342789	36705519	0.04553	rs9546331	2.13	rs7339267	0.02836	15.24	1.282	67	67	enet	0.041	4.90E-67	1.197	0.2315
SeqId_6617_12	FCRL6	1	159770301	159786047	0.06467	rs876538	-1.929	rs6656979	6.62E-02	-22.28	1.253	46	46	enet	0.077	6.90E-128	-1.1963	0.231591
SeqId_16307_22	UNC5D	8	35092993	35654068	0.06753	rs193231189	2.119	rs3108622	0.037601	-17.3	1.043	59	59	enet	0.061	5.60E-101	-1.1955	0.23189
SeqId_12738_43	NISCH	3	52489134	52527084	0.00337	rs143380194	2.25	rs4687616	7.84E-04	3.95	-1.196	31	1	top1	0.00078	0.0099	-1.196	2.32E-01
SeqId_2737_22	NOV	8	120428686	120436674	0.01813	rs17793097	2.414	rs17793097	0.011459	9.81	2.414	27	27	enet	0.016	1.10E-27	1.1946	0.232235
SeqId_7916_10	S100A7	1	153430220	153433127	0.33112	rs12408942	2.304	rs9436093	2.13E-01	-39.24	1.069	37	37	enet	0.23	0	-1.1928	0.232932
SeqId_3806_55	EPHA5	4	66185281	66536213	0.04873	rs60472024	2.322	rs28542715	0.017039	12.72	-1.47	56	56	enet	0.044	1.40E-72	-1.1934	2.33E-01
SeqId_10708_3	GNRH2	20	3024268	3026393	0.00882	rs6084207	-1.55	rs6138990	0.006124	-6.98	-1.191	37	1	top1	0.0061	1.70E-11	1.191	0.23365
SeqId_17329_2	BDH2	4	103998749	104021027	0.04677	rs78336913	2.068	rs78336913	0.031242	16.45	2.068	57	57	enet	0.045	8.40E-74	1.1909	2.34E-01
SeqId_15300_66	CCDC134	22	42196683	42228168	0.07995	rs75762735	-2.12	rs11090057	0.05864	20.86	0.913	40	40	enet	0.079	1.80E-130	1.1894	0.23428
SeqId_9326_33	ITIH2	10	7745279	7791483	0.15906	rs77086133	2.69	rs17142907	0.225512	40.37	1.076	53	53	enet	0.23	0	1.188325	0.2347
SeqId_14246_50	DOK2	8	21766384	21771201	0.01841	rs112252411	-2.849	rs2054713	0.002794	6.63	1.01	60	60	enet	0.0088	1.00E-15	-1.1879	0.234858
SeqId_12378_71	TAPBP	6	33267471	33282164	0.33074	rs2071889	2.23	rs1014779	0.09983	-27.11	1.98	122	122	enet	0.17	4.70E-303	-1.187	2.35E-01
SeqId_18222_34	SH3GLB2	9	131769315	131790610	0.10832	rs35106422	2.564	rs11537529	0.16306	-34.34	-0.826	46	46	enet	0.17	1.00E-301	1.1887	2.35E-01
SeqId_2744_57	|IGHG3|IGHG	14	106202680	106209408	0.01878	rs6576110	-2.434	rs11621145	0.013447	9.98	-1.016	16	16	enet	0.015	3.80E-26	-1.1827	2.37E-01
SeqId_9185_15	TFF1	21	43782391	43786644	0.0749	rs34333540	1.28	rs3761376	0.04698	-18.44	1.08	33	33	enet	0.053	5.30E-87	-1.1835	0.237
SeqId_15585_304	FBLN5	14	92335755	92414331	0.04077	rs12589716	-3.949	rs2267994	0.009389	8.54	1.1076	75	75	enet	0.022	8.10E-37	1.1809	2.38E-01
SeqId_17672_184	GIF	11	59596746	59612972	0.00435	rs150884181	1.176	rs150884181	0.005492	-6.49	1.176	4	1	top1	0.0055	1.80E-10	-1.176	2.40E-01
SeqId_11516_7	FABP1	2	88422501	88427581	0.04625	rs79992736	1.788	rs2241883	0.0356	-16.14	-1.174	5	1	top1	0.036	5.20E-59	1.174	2.40E-01
SeqId_3495_15	CXCL6	4	74702357	74714781	0.26505	rs62312151	-3.17	rs16850073	0.179899	36.05	0.719	38	38	enet	0.19	0	1.1728	2.41E-01
SeqId_12386_11	RNPEP	1	201951500	201975275	0.38573	rs12086240	-3.468	rs6689634	1.22E-01	29.8	0.578	130	130	enet	0.3	0	-1.1712	0.241529
SeqId_4479_14	SERPING1	11	57364860	57386644	0.28042	rs144052446	2.615	rs10896631	0.3255	-48.49	1.142	58	58	enet	0.35	0	-1.16688	2.43E-01
SeqId_2925_9	SERPINE1	7	100770385	100782528	0.02664	rs221786	3.844	rs2227631	0.016395	-10.97	0.51	59	59	enet	0.02	4.60E-34	-1.1668	0.243284
SeqId_16809_1	NME4	16	446725	460367	0.04953	rs7188886	-2.35	rs6600214	0.01225	9.79	-0.25	66	66	enet	0.027	4.40E-45	-1.164	2.44E-01
SeqId_6706_18	ALPPL2	2	233271554	233275424	0.00999	rs12478529	1.284	rs35458538	0.0059	7.04	1.166	9	1	top1	0.0059	3.90E-11	1.1655	2.44E-01
SeqId_10818_36	SMPD1	11	6411670	6416228	0.26871	rs117842668	2.175	rs1050239	0.129988	-30.64	0.607	63	63	enet	0.16	3.10E-282	-1.16312	2.45E-01
SeqId_13740_51	FRZB	2	183698002	183731365	0.42053	rs189723305	2.555	rs288326	0.08248	24.48	0.279	123	123	enet	0.23	0	-1.1618	2.45E-01
SeqId_17164_15	ANXA4	2	69871557	70054244	0.04655	rs62135760	-1.992	rs2228203	0.08322	-24.69	-0.852	34	34	enet	0.085	2.80E-142	1.1631	2.45E-01
SeqId_5132_71	IL27RA	19	14142574	14164030	0.46807	rs12609197	2.542	rs35026308	0.279426	-44.92	-1.116	66	66	enet	0.29	0	1.15825	0.246764
SeqId_3003_29	NCR3	6	31556672	31560783	0.27617	rs2106074	-4.29	rs986475	0.06555	-21.8	-0.942	107	107	enet	0.11	5.60E-188	1.157	2.47E-01
SeqId_18182_24	PCK1	20	56136136	56143177	0.04566	rs6128058	-1.9	rs2070756	0.022954	13.59	-0.997	20	20	enet	0.041	1.80E-68	-1.1576	0.24704
SeqId_16918_198	TLR3	4	186990309	187009227	0.36105	rs6839415	-5.732	rs3775291	0.255351	-42.95	1.035	73	73	enet	0.34	0	-1.1563	2.48E-01

SeqId_12703_6	NEK7	1	198126124	198291550	0.04297	rs12080584	2.022	rs142662761	1.89E-02	-11.8	-1.152	11	1	top1	0.019	6.50E-32	1.152	0.249321
SeqId_8013_9	LMAN2L	2	97371666	97405801	0.02994	rs2280355	-1.722	rs58361269	0.02616	14.04	-1.152	9	9	enet	0.026	8.60E-44	-1.1476	2.51E-01
SeqId_14131_37	EFNB2	13	107142093	107188010	0.04744	rs1485626	-2.24	rs59166663	0.018	11.75	2.042	92	92	enet	0.041	1.80E-68	1.145	0.2521
SeqId_6461_54	APOC3	11	116700422	116703788	0.01383	rs3825041	2.231	rs5141	0.003457	-6.93	1.2	20	20	enet	0.0093	1.60E-16	-1.14171	2.54E-01
SeqId_9962_1	CDHR5	11	616577	626078	0.02256	rs74046658	-2.257	rs7108757	0.014275	10.59	-0.633	33	33	enet	0.017	2.50E-29	-1.13424	2.57E-01
SeqId_8299_66	LILRA4	19	54844456	54850433	0.21418	rs147598737	2.616	rs12976217	0.07329	23.25	0.879	38	38	enet	0.093	4.40E-155	1.13263	0.257371
SeqId_9906_21	SPOCK3	4	167654535	168155947	0.21002	rs1600103	3.151	rs35065151	0.224187	40.26	1.311	69	69	enet	0.25	0	1.1309	2.58E-01
SeqId_14618_26	VOPP1	7	55503749	55640681	0.01666	rs9642585	3.26	rs117042408	0.014339	-10.22	-0.073	20	20	enet	0.017	7.50E-29	1.1308	0.258121
SeqId_8973_23	FCRL4	1	157543537	157567875	0.40663	rs115489340	1.985	rs11582663	2.64E-01	-43.69	-0.861	58	58	enet	0.3	0	1.1302	0.258398
SeqId_13118_5	SMOC1	14	70320848	70499083	0.13356	rs1275828	2.35	rs1958078	0.071051	22.67	-0.5811	96	96	enet	0.12	5.90E-210	1.1281	2.59E-01
SeqId_3389_7	SERPINA5	14	95027779	95059455	0.026	rs28929474	3.884	rs10133793	0.010214	8.82	-0.181	48	48	enet	0.016	6.30E-28	1.1288	2.59E-01
SeqId_6462_12	TIMP4	3	12194568	12200412	0.07576	rs111833688	-2	rs184262	8.61E-02	25.12	-1.213	46	46	enet	0.11	1.50E-192	-1.12897	2.59E-01
SeqId_11388_75	WFDC2	20	44098393	44110172	0.01647	rs6032344	2.41	rs6032226	0.003094	-6.07	0.329	28	28	enet	0.0093	1.30E-16	-1.1286	0.25908
SeqId_15514_26	BCHE	3	165490692	165555250	0.06265	rs143756012	1.8	rs73165060	5.83E-02	-21.34	0.716	51	51	enet	0.093	5.20E-156	-1.12711	2.60E-01
SeqId_18373_13	RDX	11	109735021	110167437	0.01341	rs9633913	-2.105	rs7935163	0.002671	-5.48	-0.815	51	51	enet	0.0055	1.90E-10	1.12304	2.61E-01
SeqId_17782_23	ACAA1	3	38144620	38178733	0.09243	rs116805069	-2.6	rs2229528	4.29E-02	-17.66	1.063	49	49	enet	0.046	5.20E-76	-1.123	2.61E-01
SeqId_13624_17	NADK	1	1682671	1711896	0.0276	rs12567685	1.289	rs4648629	4.58E-02	-18.51	-0.952	29	29	enet	0.047	7.00E-77	1.1207	0.262436
SeqId_18295_102	GRHPR	9	37422663	37436987	0.09237	rs12379335	-2.14	rs4878690	0.05844	20.59	-0.8	60	60	enet	0.064	1.10E-105	-1.1203	2.63E-01
SeqId_4220_39	FER	5	108083542	108532542	0.03782	rs62362299	-2.186	rs79536589	1.97E-02	12.42	0.684	52	52	enet	0.03	1.20E-50	1.11767	0.26371
SeqId_7980_72	B3GNT2	2	62423250	62451866	0.04809	rs62179413	2.629	rs34361686	0.02153	13.95	1.023	72	72	enet	0.051	2.90E-84	-1.1145	2.65E-01
SeqId_5509_7	EGF	4	110834039	110934922	0.05944	rs74344680	-2.523	rs10029654	0.064586	21.75	-1.139	38	38	enet	0.068	5.40E-113	-1.1141	2.65E-01
SeqId_9266_1	TREM1	6	41235664	41254420	0.18034	rs78433713	2.23	rs2234243	0.12285	-29.82	-0.557	107	107	enet	0.23	0	1.11	2.67E-01
SeqId_17384_110	PFKM	12	48498922	48540187	0.00611	rs7960122	-1.636	rs3742074	0.003959	6.37	-0.772	29	29	enet	0.0069	9.20E-13	-1.10697	0.26831
SeqId_8989_40	SCUBE1	22	43593286	43739378	0.05513	rs150081595	-2.46	rs13053545	0.01369	10.9	0.023	68	68	enet	0.038	5.60E-63	-1.1061	0.26867
SeqId_9251_28	MAN2B2	4	6576916	6625089	0.31372	rs7685745	3.088	rs2301790	0.416168	54.8	-1.248	39	39	enet	0.44	0	-1.106	2.69E-01
SeqId_13472_35	HDHD2	18	44633779	44676871	0.17178	rs191751997	-3.06	rs79231363	0.18418	36.68	1.637	106	106	enet	0.23	0	1.102	0.2704
SeqId_19372_7	MDGA2	14	47308832	48144808	0.31916	rs113731710	-2.856	rs145891385	0.06205	21.22	1.051	218	218	enet	0.1	6.50E-171	1.0987	2.72E-01
SeqId_3173_49	NAAA	4	76834813	76862166	0.25311	rs66828967	-3.072	rs28451731	0.243323	-41.99	-1.48	114	114	enet	0.37	0	1.0968	2.73E-01
SeqId_5634_39	POFUT1	20	30795683	30826461	0.25401	rs57532134	1.97	rs76143353	0.124336	-29.97	-1.227	18	18	enet	0.13	1.40E-213	1.0949	0.27355
SeqId_15367_38	BPIFB1	20	31861286	31897677	0.06088	rs6087517	2.15	rs2424963	0.047786	-18.88	0.88182	65	65	enet	0.059	2.80E-98	-1.0942	0.27385
SeqId_8296_117	KDELC2	11	108342839	108369111	0.28739	rs76395038	-2.257	rs141379009	0.109415	-28.42	-0.559	76	76	enet	0.15	8.20E-261	1.09195	2.75E-01
SeqId_12524_18	SAT2	17	7529552	7531194	0.02866	rs3027231	-2.252	rs13894	0.008789	-9.71	-1.352	18	18	enet	0.024	1.10E-40	1.0902	0.27563
SeqId_2677_1	EGFR	7	55086710	55279321	0.09007	rs9642585	3.26	rs75059484	0.010444	-10.5	0.599	76	76	enet	0.058	9.60E-96	-1.09	0.275695
SeqId_5663_18	PF4V1	4	74719013	74720200	0.00674	rs11729931	3.242	rs872914	0.006023	-6.69	1.086	16	1	top1	0.006	2.50E-11	-1.086	2.77E-01
SeqId_9950_229	LAG3	12	6881678	6887621	0.03133	rs3782735	2.246	rs3782735	0.012072	10.06	2.246	23	23	enet	0.017	1.60E-29	1.08509	0.27788
SeqId_15395_15	GSTM1	1	110230436	110251661	0.37219	rs116377258	3.336	rs115929572	7.54E-02	24.34	-0.989	182	182	enet	0.3	0	1.0827	0.278955
SeqId_3366_51	ECM1	1	150480538	150486265	0.26156	rs4970935	-2.343	rs3737240	3.48E-01	-50.13	-1.739	49	49	enet	0.4	0	1.0792	0.28049
SeqId_6359_50	POMGNT2	3	43120724	43147571	0.06195	rs13433902	2.74	rs2002182	7.94E-02	-24.04	-1.049	19	19	enet	0.08	4.70E-133	1.07742	2.81E-01
SeqId_6520_87	MGP	12	15033798	15038791	0.05311	rs12832328	2.935	rs2900342	0.083105	-25.06	0.566	74	74	enet	0.092	4.80E-154	-1.07619	0.28184
SeqId_9312_8	AZGP1	7	99564343	99573665	0.09743	rs79250696	2.52	rs1981550	0.112418	-28.56	-1.1293	21	21	enet	0.11	6.00E-193	1.0731	0.283248
SeqId_15615_8	LILRB3	19	54720147	54727378	0.38178	rs41514951	2.812	rs12985875	0.093072	26.04	0.2242	275	275	enet	0.25	0	1.07285	0.283336
SeqId_3045_72	PTN	7	136912088	137028520	0.05357	rs3800642	-3.096	rs10255150	0.040897	17.31	-0.32	69	69	enet	0.051	1.20E-83	-1.0726	0.283457
SeqId_5105_2	RTN4R	22	20228938	20270769	0.16685	rs117474730	-2.94	rs75766	0.06115	-21.05	-1.464	73	73	enet	0.13	5.20E-217	-1.0693	0.28496
SeqId_2974_61	CNTN1	12	41086241	41466217	0.163	rs4768311	2.974	rs11177604	0.036702	16.97	0.097	81	81	enet	0.11	3.20E-187	-1.06483	0.28695
SeqId_8346_9	DPP7	9	140004998	140009629	0.15319	rs34223864	-3.437	rs4880198	0.09399	-26.1	-0.765	105	105	enet	0.11	1.00E-182	1.0636	2.88E-01
SeqId_6388_21	CCDC126	7	23637001	23684327	0.08979	rs112506627	-1.359	rs35121828	0.092782	26.08	0.6	27	27	enet	0.11	9.70E-181	1.0619	0.288263
SeqId_2834_54	KLK8	19	51499263	51505967	0.11625	rs17800825	-2.688	rs10410942	0.07841	-23.88	1.153	70	70	enet	0.11	4.40E-180	-1.06121	0.288593
SeqId_9282_12	CRISP2	6	49660071	49681303	0.22014	rs188220503	-1.89	rs555247	0.12361	29.92	0.356	82	82	enet	0.24	0	-1.056	2.91E-01
SeqId_10754_113	PROK2	3	71820806	71834357	0.16007	rs73092567	-1.72	rs7644362	1.70E-01	-35.01	1.027	22	22	enet	0.19	0	-1.05326	2.92E-01
SeqId_13463_1	PXDN	2	1635659	1748624	0.03503	rs143118143	2.222	rs10185594	0.02213	12.78	-0.65	49	49	enet	0.032	5.50E-53	-1.0509	2.93E-01
SeqId_4984_83	ESD	13	47345391	47371555	0.30363	rs73491331	2.37	rs9778	0.12571	-30.14	-0.813	43	43	enet	0.2	0	1.05	0.2939
SeqId_8304_50	TNFRSF11B	8	119935796	119964124	0.06053	rs117962464	-2.863	rs2468184	0.007545	8.38	-0.03	92	92	enet	0.044	2.80E-73	-1.0491	0.294122
SeqId_12571_14	ARL3	10	104433488	104474154	0.16645	rs11191349	2.8	rs8354	0.088622	-25.42	1.16	46	46	enet	0.16	6.50E-274	-1.048998	0.2942

SeqId_4455_89	MFGE8	15	89441914	89456612	0.06656	rs6496536	-1.42	rs12911703	0.07782	23.78	0.986	33	33	enet	0.092	4.70E-153	1.049	0.2942
SeqId_7059_14	LILRA6	19	54740468	54746640	0.46874	rs12975366	-2.502	rs34810796	0.191227	37.18	0.841	152	152	enet	0.32	0	1.04359	0.296676
SeqId_6425_87	MMP19	12	56229217	56236750	0.10332	rs145081572	1.979	rs56180965	0.008361	-8.18	-1.172	65	65	enet	0.021	1.80E-35	1.04296	0.29697
SeqId_17832_12	IDI2	10	1064850	1071799	0.04554	rs187940740	2.38	rs1044261	0.04325	-17.74	0.744	28	28	enet	0.056	2.60E-92	-1.035213	0.3006
SeqId_8337_65	PTPRU	1	29563028	29653325	0.07169	rs271381	1.889	rs2179795	2.53E-02	-13.69	1.169	89	89	enet	0.048	3.00E-79	-1.0349	0.300705
SeqId_8300_82	PEX14	1	10532345	10690815	0.00692	rs75827648	1.389	rs184041999	1.51E-03	-5.01	-1.176	17	17	enet	0.0038	1.10E-07	1.0346	0.300835
SeqId_3009_3	TGFBR3	1	92145900	92371892	0.04179	rs284184	-2.837	rs79859654	1.10E-02	9.34	-1.288	75	75	enet	0.029	6.70E-49	-1.0325	0.301853
SeqId_5742_14	ACP6	1	147101453	147142646	0.60994	rs2236570	2.049	rs12119079	3.50E-01	49.94	1.217	49	49	enet	0.51	0	1.0322	0.301963
SeqId_15627_83	AKT1	14	105235686	105262088	0.00475	rs34207932	1.903	rs2494741	0.000426	-4.62	-1.286	21	21	enet	0.0028	4.60E-06	1.0309	3.03E-01
SeqId_4908_6	ENG	9	130577292	130616937	0.08494	rs113453017	2.433	rs11794565	0.02918	14.55	-0.602	29	29	enet	0.041	3.70E-67	-1.0291	3.03E-01
SeqId_17764_108	RHOC	1	113243749	113250056	0.0367	rs6537749	1.453	rs7415820	2.34E-02	-13.13	1.143	42	42	enet	0.029	7.20E-48	-1.0296	0.303181
SeqId_2730_58	MICA	6	31367561	31383092	0.41169	rs2442719	-4.34	rs2523496	0.33362	-49.08	-3.0788	158	158	enet	0.5	0	1.027	3.04E-01
SeqId_18895_54	GSTM4	1	110198721	110217458	0.30574	rs116831366	3.46	rs687643	6.85E-02	-23.38	-0.089	186	186	enet	0.24	0	1.0279	0.304019
SeqId_5586_66	MINPP1	10	89264632	89313218	0.02581	rs183082741	3.29	rs59980852	0.01089	-9	-0.473	45	45	enet	0.015	4.90E-26	1.025361	0.3052
SeqId_8005_1	MXRA7	17	74668633	74707086	0.14886	rs629896	2.053	rs4789345	0.112949	28.82	1.3257	70	70	enet	0.16	2.10E-282	1.0226	0.30651
SeqId_5601_2	PGLYRP2	19	15579456	15609767	0.13338	rs4808309	-3.404	rs10164310	0.044805	-18.08	1.7802	110	110	enet	0.062	2.10E-102	-1.02235	0.306614
SeqId_14048_7	IL1RAP	3	190231840	190377539	0.40058	rs4687162	-3.04	rs7611887	4.19E-01	-55.02	-0.837	69	69	enet	0.49	0	1.0186	3.08E-01
SeqId_5128_53	SLAMF6	1	160454820	160493052	0.06053	rs12144820	1.47	rs1041067	4.66E-02	-18.66	-1.022	21	21	enet	0.048	2.70E-79	1.0187	0.308343
SeqId_15384_15	KL	13	33590561	33640280	0.04859	rs147014069	2.71	rs73176836	0.00917	9.32	-1.639	96	96	enet	0.03	1.00E-50	-1.014	0.3104
SeqId_4969_2	CA1	8	86239837	86291243	0.08683	rs144801746	-2.098	rs116866430	0.045104	-18.24	0.3784	41	41	enet	0.064	1.30E-106	-1.0134	0.310876
SeqId_9267_2	CPA4	7	129932974	129964020	0.42622	rs78828774	-2.766	rs34587586	0.46478	-57.9	1.012	77	77	enet	0.48	0	-1.0124	0.311354
SeqId_15503_20	LEFTY2	1	226124298	226129083	0.24944	rs138459974	2.707	rs360077	2.59E-01	-43.22	1.062	68	68	enet	0.34	0	-1.0115	0.311763
SeqId_11493_169	DYNLL2	17	56160780	56172903	0.07713	rs117312367	-2.247	rs9902118	0.064374	21.65	-1.45	36	36	enet	0.084	1.70E-139	-1.011	0.31201
SeqId_8465_52	CTSH	15	79213400	79241916	0.39211	rs55919125	2.759	rs2289702	0.30453	-46.9	1.248	77	77	enet	0.38	0	-1.0099	0.3125
SeqId_9738_7	FAF2	5	175874629	175937075	0.00325	rs72807229	1.126	rs1366240	1.73E-04	4.17	1.018	4	4	enet	0.00028	0.083	1.00814	0.31339
SeqId_8759_29	A4GALT	22	43088127	43117304	0.07726	rs5758893	3.23	rs8138197	0.06816	-22.27	0.698	48	48	enet	0.072	3.10E-119	-1.007	0.31391
SeqId_16851_50	SCO2	22	50961997	50964890	0.01816	rs131811	2.96	rs11479	0.0118	-9.55	-1.158	11	11	enet	0.012	5.10E-21	1.0039	0.31543
SeqId_6086_15	CHRDL2	11	74407474	74442471	0.09653	rs55837439	2.244	rs61389091	0.044891	-18.03	-0.173	64	64	enet	0.057	8.30E-95	1.00286	3.16E-01
SeqId_9021_1	HAVCR1	5	156456424	156486130	0.09816	rs112487859	2.702	rs13173581	8.67E-02	-25.06	0.287	47	47	enet	0.12	7.40E-195	0.99795	0.3183
SeqId_3352_80	CA6	1	9005926	9035151	0.43027	rs72645559	2.393	rs3765963	2.02E-01	38.17	0.071	63	63	enet	0.35	0	-0.9973	0.318639
SeqId_3331_8	RGMB	5	98104354	98134347	0.06865	rs2369266	-2.085	rs2368553	5.70E-02	20.4	-0.674	61	61	enet	0.082	1.60E-136	-0.99708	0.31873
SeqId_4929_55	SHBG	17	7517264	7536700	0.05799	rs7223364	2.917	rs11651783	0.030256	-15.03	-0.462	100	100	enet	0.065	7.10E-108	0.9958	0.31934
SeqId_16609_106	KIRREL2	19	36346763	36358038	0.01776	rs1005194	2.558	rs35854130	0.004491	-8.34	-0.978	34	34	enet	0.015	2.00E-25	-0.99487	0.319798
SeqId_3041_55	MRC2	17	60705031	60770958	0.27258	rs146385050	-2.812	rs146385050	0.052744	-19.64	-2.812	97	97	enet	0.12	1.00E-207	0.9925	0.32098
SeqId_2999_6	LSAMP	3	115521210	116858236	0.08755	rs115930484	-2.73	rs1835674	3.31E-02	15.94	0.118	70	70	enet	0.054	8.80E-89	-0.9899	3.22E-01
SeqId_12549_33	HPGDS	4	95219686	95264027	0.20218	rs72665697	2.127	rs10033662	0.153231	33.27	0.037	63	63	enet	0.25	0	-0.9906	3.22E-01
SeqId_2948_58	GHR	5	42423541	42721980	0.1449	rs4146624	-1.781	rs4866942	5.56E-02	-20.26	-0.767	26	26	enet	0.1	2.10E-175	0.99027	0.32204
SeqId_9459_7	FAS	10	90750288	90776816	0.15623	rs117578788	2.25	rs7911226	0.078941	-23.93	-1.437	106	106	enet	0.11	8.70E-189	0.987807	0.3232
SeqId_19560_23	PLXNA4	7	131808091	132333447	0.04441	rs62467596	-2.255	rs62622406	0.013922	-10.16	-1.073	81	81	enet	0.026	2.90E-43	0.985	0.324644
SeqId_19254_125	GMPR	6	16238818	16295780	0.2731	rs62387699	-1.81	rs6459467	0.42834	55.59	0.97	29	29	enet	0.43	0	0.984	3.25E-01
SeqId_17692_2	BTN3A3	6	26440700	26453643	0.4582	rs117225308	2.43	rs9393711	0.2584	-43.2	-1.236	116	116	enet	0.47	0	0.985	0.325
SeqId_5060_62	CD274	9	5450503	5470566	0.07503	rs71498619	-1.604	rs822342	0.04959	19.08	-1.285	23	23	enet	0.057	1.70E-93	-0.9795	3.27E-01
SeqId_3617_80	HGFAC	4	3443695	3451213	0.52478	rs13137747	4.081	rs2498323	0.311692	-47.42	0.527	79	79	enet	0.39	0	0.9757	3.29E-01
SeqId_7999_23	ENTPD1	10	97471536	97637023	0.03226	rs17111247	2.56	rs11598475	0.037809	16.64	0.601	62	62	enet	0.045	2.70E-74	0.975936	0.3291
SeqId_19161_1	USP15	12	62654119	62811211	0.09869	rs71465154	-2.986	rs11612349	0.140268	32.01	1.15	64	64	enet	0.21	0	0.97579	0.32917
SeqId_4548_4	FUT3	19	5842899	5851485	0.25994	rs12460963	2.957	rs708686	0.189613	-37.04	-1.045	116	116	enet	0.22	0	0.97502	0.32955
SeqId_9241_40	SIRPG	20	1609798	1638425	0.08429	rs2144546	1.59	rs6043409	0.108276	28.06	-1.139	30	30	enet	0.11	1.50E-185	-0.9718	0.33116
SeqId_12396_19	HIBCH	2	191054461	191208919	0.14258	rs291431	1.224	rs291466	0.33359	49.07	1.037	30	30	enet	0.33	0	0.9643	3.35E-01
SeqId_15523_9	ALAD	9	116148592	116163570	0.37231	rs78352057	3.036	rs8177797	0.0642	-21.56	1.914	135	135	enet	0.16	5.40E-283	-0.9643	3.35E-01
SeqId_19130_81	SERPINB8	18	61637263	61687013	0.39607	rs1363002	2.56	rs3826616	0.08947	-25.49	0.675	54	54	enet	0.12	6.30E-204	-0.963	0.3354
SeqId_4992_49	GRN	17	42422614	42430474	0.0453	rs187822209	-1.678	rs5848	0.024947	-13.49	-0.9508	28	28	enet	0.028	1.30E-46	0.9598	0.33715
SeqId_5392_73	FAS	10	90750288	90776816	0.15453	rs117578788	2.25	rs7911226	0.069757	-22.48	-1.437	96	96	enet	0.11	2.30E-177	0.958715	0.3377
SeqId_3151_6	IL2RA	10	6052652	6104333	0.01792	rs12778662	2.17	rs12722497	0.008421	8	0.958	13	1	top1	0.0084	3.50E-15	0.958	0.3381

SeqId_4250_23	NSFL1C	20	1422807	1454487	0.0254	rs77123904	-1.67	rs4814391	0.003087	7.54	-0.753	50	50	enet	0.016	7.40E-27	-0.9557	0.33921
SeqId_3340_53	THBS4	5	79287134	79379110	0.12817	rs7725944	2.463	rs13167730	7.16E-02	22.77	-1.356	48	48	enet	0.12	3.10E-206	-0.95489	0.33963
SeqId_18206_18	ADH6	4	100123795	100140694	0.07629	rs144053138	-2.642	rs62325239	0.097044	-26.54	-0.366	74	74	enet	0.14	5.60E-233	0.9546	3.40E-01
SeqId_3499_77	IL17B	5	148750887	148783765	0.01892	rs1438688	-2.061	rs6862301	1.46E-02	10.38	0.954	11	1	top1	0.015	3.60E-25	0.954	0.34008
SeqId_18173_11	AKR7A3	1	19609067	19615264	0.26029	rs56070474	-2.335	rs2231198	1.07E-01	-24.06	1.216	117	117	enet	0.23	0	0.9518	0.341201
SeqId_9536_16	EDDM3A	14	21214046	21216539	0.01405	rs34552133	0.948	rs34552133	0.008686	-8.25	0.948	3	1	top1	0.0087	1.30E-15	-0.948	3.43E-01
SeqId_12575_30	MTHFD1	14	64854749	64930175	0.03986	rs3783731	-2.302	rs10146204	0.016785	-11.12	-0.646	50	50	enet	0.031	9.20E-51	0.9463	3.44E-01
SeqId_17702_53	UGT1A1	2	234668916	234681946	0.08571	rs113868955	2.15	rs887829	0.16126	-34.14	0.771	32	32	enet	0.17	2.70E-289	-0.9456	3.44E-01
SeqId_10737_96	SERPINB1	6	2832566	2842193	0.0611	rs17214342	2.45	rs316343	0.0432	18.1	0.665	25	25	enet	0.051	6.60E-85	0.946	0.344
SeqId_7918_114	AMY1A	1	104198382	104207176	0.40275	rs113091904	3.12	rs78811372	3.70E-02	-16.57	0.849	174	174	enet	0.14	7.40E-231	0.9462	0.344048
SeqId_10924_258	CYB5D2	17	4046462	4090605	0.1055	rs72833799	-2.292	rs183282334	0.049895	-19.13	-0.1746	34	34	enet	0.088	7.10E-147	0.946	0.34416
SeqId_17456_53	GOLM1	9	88641061	88715088	0.07783	rs149100304	-2.519	rs138447426	0.04061	18.04	0.029	40	40	enet	0.075	4.70E-125	0.9428	3.46E-01
SeqId_2590_69	ROR1	1	64239688	64647181	0.256	rs855312	-3.385	rs2224876	1.22E-01	-29.64	0.978	134	134	enet	0.2	0	-0.9394	0.347524
SeqId_2985_35	CXCL1	4	74735110	74737025	0.1322	rs6832422	-3.255	rs1366946	0.132887	31	0.806	42	42	enet	0.17	2.00E-295	0.9378	3.48E-01
SeqId_6965_19	CNTNAP2	7	145813094	148118090	0.1456	rs188929111	-2.416	rs6951436	0.228564	-40.76	-1.167	85	85	enet	0.25	0	0.932	0.351356
SeqId_7244_16	IGFLR1	19	36229702	36233354	0.21721	rs112050622	2.262	rs12459634	0.25179	-42.63	-0.781	68	68	enet	0.31	0	0.93001	0.352368
SeqId_11208_15	NAGPA	16	5074845	5084142	0.2774	rs7197168	1.72	rs12599777	0.10104	-27.07	-0.377	92	92	enet	0.19	0	0.9251	3.55E-01
SeqId_8866_53	QPCTL	19	46195661	46207247	0.14485	rs11083768	2.993	rs17850756	0.098341	-26.66	1.6904	103	103	enet	0.11	4.80E-189	-0.92471	0.355118
SeqId_19590_46	SFTPD	10	81697223	81742370	0.15866	rs150481960	-2.64	rs7084667	0.124325	30.06	0.177	112	112	enet	0.16	7.90E-279	0.922852	0.3561
SeqId_15617_8	XCL2	1	168510006	168513235	0.02332	rs10429892	1.429	rs1998510	1.54E-02	10.68	0.922	12	1	top1	0.015	2.20E-26	0.922	0.356529
SeqId_7926_13	SPINT3	20	44141101	44144262	0.05571	rs7272717	-2.38	rs6032259	0.081334	24.43	1.327	33	33	enet	0.084	1.60E-140	0.9197	0.35771
SeqId_5034_79	PRSS2	7	142468247	NA	0.27186	rs75940454	-3.382	rs1799886	0.074061	23.19	-1.2962	49	49	enet	0.16	1.80E-271	-0.9187	0.358247
SeqId_3324_51	LY9	1	160765864	160798051	0.14097	rs723155	2.558	rs12128261	1.24E-01	30.13	1.764	94	94	enet	0.15	2.30E-262	0.916	0.359655
SeqId_6404_20	C1QL1	17	43037061	43045671	0.07454	rs1230084	-3.275	rs7225162	0.054374	-19.9	0.776	56	56	enet	0.081	6.10E-134	-0.9159	0.3597
SeqId_5803_24	C3	19	6677715	6730573	0.0332	rs143835362	1.298	rs11569415	0.009865	10.79	-0.5454	33	33	enet	0.025	1.60E-41	-0.91219	0.361668
SeqId_10445_20	APOM	6	31620193	31625987	0.10012	rs2855811	-2.77	rs805264	0.04408	-18.05	-0.511	40	40	enet	0.051	1.20E-83	0.912	3.62E-01
SeqId_7945_10	SEMA6A	5	115779251	115910519	0.08417	rs111705252	-3.101	rs3733724	2.27E-02	12.89	1.626	72	72	enet	0.052	4.40E-86	0.91141	0.36208
SeqId_16872_248	GSTZ1	14	77787339	77797940	0.26234	rs11850771	-2.278	rs7975	0.302694	46.75	0.505	69	69	enet	0.33	0	0.9071	3.64E-01
SeqId_11681_8	AGFG1	2	228336868	228425933	0.05258	rs3769641	-2.032	rs11679534	0.07358	-23.08	0.907	21	1	top1	0.074	4.40E-122	-0.907	3.64E-01
SeqId_8097_77	LIPN	10	90519739	90539383	0.21984	rs72809325	2.48	rs10509554	0.276844	44.7	0.306	25	25	enet	0.31	0	0.907055	0.3644
SeqId_3283_21	TGFBI	5	135364677	135399507	0.21024	rs77410943	-2.659	rs13159365	2.21E-01	-39.94	-0.744	106	106	enet	0.24	0	0.9034	0.36631
SeqId_18387_7	ST13	22	41220539	41252643	0.03693	rs9611338	-1.68	rs138337	0.0379	16.86	-0.937	21	21	enet	0.039	2.80E-64	-0.9007	0.36774
SeqId_14708_59	C8G	9	139839637	139841427	0.12842	rs4880088	1.576	rs7862602	0.12423	-30.11	1.336	10	10	enet	0.15	7.50E-253	-0.8999	3.68E-01
SeqId_7246_4	FREM2	13	39261214	39461268	0.01892	rs149141963	2.13	rs9603418	0.00737	8.64	0.591	37	37	enet	0.0082	7.30E-15	0.899	0.3688
SeqId_5349_69	DLL1	6	170591294	170615653	0.05426	rs112066611	2.07	rs4710790	0.01431	10.45	-0.858	51	51	enet	0.033	2.20E-54	-0.899	3.69E-01
SeqId_14684_17	CAPN2	1	223889295	223963720	0.13521	rs11580460	-2.193	rs188530181	4.39E-02	-17.83	0.86	86	86	enet	0.066	5.10E-110	-0.8926	0.372068
SeqId_13090_17	S100A6	1	153507075	153508720	0.03502	rs116793967	-1.341	rs60969679	1.97E-02	12.14	-0.485	24	24	enet	0.029	1.00E-47	-0.8909	0.372963
SeqId_15435_4	PNP	14	20937113	20945248	0.25605	rs17277543	2.583	rs1049564	0.127699	-30.47	-0.689	142	142	enet	0.18	2.30E-305	0.8901	3.73E-01
SeqId_13998_26	ADSSL1	14	105190566	105213662	0.01694	rs61758463	-2.417	rs10142660	0.005586	7.35	0.986	38	38	enet	0.0093	1.40E-16	0.8896	3.74E-01
SeqId_15610_72	LAP3	4	17578821	17609595	0.02543	rs77726502	1.641	rs114838597	0.013428	9.92	0.1	25	25	enet	0.02	4.80E-33	-0.8863	3.75E-01
SeqId_10938_13	CD58	1	117057156	117113661	0.03207	rs143770903	2.147	rs60612523	1.55E-02	10.81	1.005	20	20	enet	0.016	4.20E-27	0.8862	0.375518
SeqId_3519_3	CCL17	16	57438679	57449975	0.05517	rs9922349	2.72	rs4396523	0.04	17.09	-0.34	50	50	enet	0.061	1.90E-101	-0.8857	3.76E-01
SeqId_9826_135	FHIT	3	59733003	61237133	0.01434	rs189635795	-2.08	rs13070475	1.00E-02	8.93	-1.291	25	25	enet	0.011	1.70E-19	-0.8856	3.76E-01
SeqId_3445_53	IL15RA	10	5985602	6020150	0.02793	rs10508303	-2.55	rs8177643	0.010609	8.99	-0.233	31	31	enet	0.018	9.70E-31	-0.884396	0.3765
SeqId_13669_6	FGFR3	4	1795020	1810599	0.11668	rs188698778	-2.585	rs79825628	0.03711	17.01	0.142	85	85	enet	0.089	3.90E-149	0.8831	3.77E-01
SeqId_12343_14	ACAP2	3	194995474	195163773	0.00509	rs9886959	2.5	rs1832666	9.53E-04	-4.47	0.882	37	1	top1	0.00095	0.005	-0.882	3.78E-01
SeqId_5581_28	FGL1	8	17721889	17767874	0.17575	rs6586641	-3.009	rs3739406	0.220796	40.11	0.743	51	51	enet	0.3	0	0.8808	0.378417
SeqId_8476_11	CHGA	14	93389495	93401630	0.05369	rs35665900	-1.891	rs729940	0.026188	-13.88	0.564	23	23	enet	0.033	8.40E-55	-0.8797	3.79E-01
SeqId_2851_63	C5	9	123714613	123812553	0.04546	rs143406446	-2.049	rs17220750	0.01522	10.69	0.355	28	28	enet	0.035	1.60E-58	0.8768	3.81E-01
SeqId_18884_22	DNAJB4	1	78444859	78483648	0.08837	rs278853	2.436	rs7514180	3.96E-02	-17.17	-2.128	44	44	enet	0.074	3.00E-123	0.8746	0.381785
SeqId_11227_31	TBL2	7	72981867	72993121	0.04047	rs4717865	2.704	rs73134986	0.013668	10.12	0.729	27	27	enet	0.014	8.00E-24	-0.8742	0.38202
SeqId_9002_36	SERPINA11	14	94908801	94919137	0.09915	rs28929474	3.884	rs55911632	0.030851	-15.12	-0.074	95	95	enet	0.075	1.30E-124	-0.8732	3.83E-01
SeqId_10907_116	NTM	11	131240373	132206716	0.1924	rs585512	2.921	rs2511781	0.160317	34.04	-0.515	92	92	enet	0.2	0	-0.86794	3.85E-01

SeqId_5102_55	MICB	6	31462658	31478901	0.43328	rs3130062	-4.45	rs3094011	0.16346	-34.42	-0.071	159	159	enet	0.36	0	-0.869	3.85E-01
SeqId_12687_2	DECR1	8	91013633	91065861	0.02246	rs117770316	2.406	rs1805806	0.015824	-11.25	0.041	29	29	enet	0.023	6.60E-38	-0.8672	0.385812
SeqId_9264_11	CTSO	4	156842732	156875064	0.02782	rs114671994	1.559	rs2334114	0.009841	-10.47	0.477	67	67	enet	0.029	1.70E-47	-0.8665	3.86E-01
SeqId_13931_22	PSMD9	12	122326638	122356203	0.01611	rs111773380	-1.53	rs10743185	0.020002	12.18	0.866	13	1	top1	0.02	9.20E-34	0.866	0.38649
SeqId_10451_11	NUCB1	19	49403569	49426629	0.04165	rs34332722	-2.343	rs28450126	0.031031	-15.1	-1.124	32	32	enet	0.039	3.50E-64	0.86472	0.387191
SeqId_10816_150	PILRA	7	99965153	99997719	0.4249	rs34919929	1.21	rs1859788	0.515673	-61.01	0.869	11	11	enet	0.51	0	-0.8621	0.388642
SeqId_9837_60	NQO1	16	69740899	69760571	0.13816	rs149992071	-2.3	rs57488237	0.1912	-37.23	-1.194	42	42	enet	0.23	0	0.8615	3.89E-01
SeqId_19556_12	CR1	1	207669492	207815110	0.33889	rs1932817	-3.543	rs679515	1.14E-01	-28.77	-0.456	168	168	enet	0.21	0	0.86	0.389806
SeqId_9370_69	GGH	8	63927440	63951966	0.14682	rs6472077	3.091	rs4739037	0.099957	27.29	-1.802	83	83	enet	0.19	4e-323	-0.8588	0.390448
SeqId_18235_16	PGP	16	2261594	2264841	0.09732	rs4787270	2.38	rs116977380	0.05448	19.9	0.488	31	31	enet	0.06	4.70E-99	0.8587	3.91E-01
SeqId_6467_65	POGLUT1	3	119187810	119213555	0.16294	rs114040582	2.79	rs17203139	1.56E-01	34.17	-0.515	58	58	enet	0.25	0	-0.85785	3.91E-01
SeqId_12619_14	PPM1A	14	60712470	60765805	0.01405	rs148399942	2.598	rs183404450	0.002548	-6.35	0.0928	14	14	enet	0.0067	2.10E-12	-0.8553	3.92E-01
SeqId_3320_49	IGFBP7	4	57896939	57976551	0.1029	rs11573114	1.719	rs1718860	0.101743	-27.56	0.451	72	72	enet	0.13	2.10E-217	-0.8567	3.92E-01
SeqId_9409_11	TPSAB1	16	1290380	1292555	0.30187	rs117631661	2.38	rs35116045	0.04873	20.87	0.711	124	124	enet	0.21	0	0.8541	3.93E-01
SeqId_7970_315	ART3	4	76932337	77033955	0.09853	rs4859610	-2.565	rs4859610	0.056013	-20.16	-2.565	92	92	enet	0.11	1.30E-176	0.8542	3.93E-01
SeqId_6402_8	PILRA	7	99965153	99997719	0.37083	rs117864261	2.031	rs1859788	0.465302	-57.95	0.869	15	15	enet	0.46	0	-0.8534	0.393444
SeqId_14107_1	MTHFS	15	80125927	80189721	0.41199	rs2562749	2.334	rs8039272	0.23231	-40.99	-0.45	81	81	enet	0.44	0	-0.851	0.3947
SeqId_8794_13	DPEP1	16	89679716	89704864	0.16532	rs61754918	-2.89	rs409170	0.13083	31.18	-0.218	73	73	enet	0.18	8.3e-310	-0.8506	3.95E-01
SeqId_3420_21	CA13	8	86132816	86196302	0.14248	rs2279701	1.367	rs113014331	0.176119	-35.67	-0.294	44	44	enet	0.26	0	0.8482	0.396339
SeqId_2849_49	AIF1	6	31582994	31584798	0.04494	rs9266302	-3.49	rs3130623	0.00865	9.37	0.874	50	50	enet	0.027	8.00E-46	0.845	3.98E-01
SeqId_6373_54	DLK1	14	101192042	101204561	0.08551	rs75741145	3.308	rs4905998	0.097793	26.91	-0.1	80	80	enet	0.12	3.60E-196	-0.8433	3.99E-01
SeqId_17802_4	NANS	9	100819014	100845359	0.0435	rs774330	2.575	rs7855984	0.01407	10.44	0.373	95	95	enet	0.035	7.70E-58	0.8432	3.99E-01
SeqId_5657_28	ST3GAL1	8	134467091	134584183	0.10361	rs2702970	1.825	rs9643300	0.060857	-21.08	1.399	65	65	enet	0.082	4.70E-136	-0.8422	0.399703
SeqId_15391_114	GAS6	13	114523522	114567049	0.08791	rs9604570	-3.8	rs6602909	0.0715	-22.74	0.151	35	35	enet	0.078	3.00E-129	-0.842	0.3999
SeqId_4459_68	PCSK7	11	117075053	117103241	0.19654	rs12802202	-2.116	rs236910	0.339376	-49.49	-0.9	40	40	enet	0.35	0	0.84144	4.00E-01
SeqId_15441_6	GM2A	5	150591711	150650001	0.25278	rs59201789	-2.275	rs72794132	1.24E-01	31.46	1.339	65	65	enet	0.26	0	0.84119	0.40024
SeqId_16616_137	ENO3	17	4851387	4860426	0.03575	rs8067142	2.247	rs238238	0.052059	-19.77	-0.6	11	11	enet	0.056	5.30E-93	0.8378	0.40212
SeqId_5858_6	YWHAZ	8	101928751	101965616	0.01463	rs34041620	1.987	rs12335167	0.001866	5.2	-0.528	43	43	enet	0.0049	1.60E-09	-0.8361	0.403086
SeqId_15513_108	PRSS8	16	31142754	31147048	0.01243	rs12925010	1.92	rs1060506	0.00544	6.81	0.835	25	1	top1	0.0054	2.10E-10	0.835	4.04E-01
SeqId_3820_68	MAPKAPK2	1	206858250	206907626	0.05599	rs78702774	-2.606	rs4256810	4.66E-02	18.42	0.833	34	1	top1	0.047	4.50E-77	0.833	0.404845
SeqId_14112_40	RELT	11	73087444	73108519	0.00613	rs12365017	3.13	rs56801796	0.006252	7.73	0.832	14	1	top1	0.0063	1.10E-11	0.832	4.05E-01
SeqId_4534_10	PRSS22	16	2902731	2908171	0.07031	rs12927970	3.33	rs7204669	0.03963	17.13	-0.625	65	65	enet	0.06	7.80E-100	0.8314	4.06E-01
SeqId_11649_3	SMAP1	6	71377479	71571714	0.2703	rs150032833	2.35	rs1917342	0.2381	-41.47	1.009	89	89	enet	0.32	0	-0.831	4.06E-01
SeqId_9183_7	IFNAR1	21	34696734	34732170	0.23867	rs62226371	-1.84	rs2211687	0.2283	-40.65	-0.613	75	75	enet	0.26	0	0.8259	0.409
SeqId_7813_6	ALPP	2	233243434	233247599	0.13737	rs75668915	2.285	rs35458538	0.05541	20.1	0.927	57	57	enet	0.098	4.20E-164	0.8246	4.10E-01
SeqId_10440_26	CLMP	11	122940580	123065956	0.07886	rs11218797	2.583	rs2302605	0.093958	26.22	0.941	55	55	enet	0.1	2.40E-171	0.81467	4.15E-01
SeqId_3073_51	IL18BP	11	71709659	71716761	0.00883	rs72958308	1.826	rs17884883	0.004719	-5.98	0.814	40	1	top1	0.0047	3.10E-09	-0.814	4.16E-01
SeqId_8309_12	HYAL1	3	50337321	50349812	0.0455	rs143885784	-3.29	rs116482870	1.52E-02	-10.72	-0.546	34	34	enet	0.021	1.40E-34	0.81295	4.16E-01
SeqId_9748_31	GSTM3	1	110276554	110283660	0.2599	rs17024393	3.255	rs1292096	9.45E-02	-26.32	-0.479	113	113	enet	0.23	0	0.812	0.416818
SeqId_11103_24	HSPB1	7	75931990	75933612	0.28721	rs112829231	3.662	rs2908201	0.241565	-41.81	1.484	83	83	enet	0.3	0	-0.8104	0.417723
SeqId_6919_3	HBZ	16	192727	204502	0.28302	rs891145	2.89	rs2461286	0.2181	-39.69	0.976	55	55	enet	0.25	0	-0.8097	4.18E-01
SeqId_4775_34	GSN	9	123970072	124095121	0.06051	rs41296071	-2.852	rs78573713	0.01968	-12.34	0.34	44	44	enet	0.045	6.60E-74	0.8076	4.19E-01
SeqId_5744_12	MENT	1	151020227	151024462	0.0072	rs4589116	1.91	rs34725710	-1.35E-04	-3.68	1.479	21	21	enet	0.0013	0.0012	-0.807	0.419645
SeqId_15525_294	ADH1C	4	100257654	100273903	0.12968	rs10006545	-1.742	rs283415	0.144197	32.27	-0.755	117	117	enet	0.17	7.50E-300	-0.8061	4.20E-01
SeqId_12449_16	PPIH	1	43124006	43142429	0.00868	rs912074	1.638	rs78676038	3.11E-03	6.67	-0.337	18	18	enet	0.0074	1.40E-13	0.8046	0.421053
SeqId_8368_102	TNFRSF1B	1	12227048	12269285	0.10458	rs41279452	-2.175	rs519064	8.16E-02	-24.77	0.517	42	42	enet	0.13	4.80E-217	-0.801	0.423146
SeqId_17325_10	GUK1	1	228327663	228336685	0.09996	rs4653973	2.69	rs61825157	1.43E-01	32.17	1.229	53	53	enet	0.15	3.90E-252	0.7978	0.424999
SeqId_9385_4	GAA	17	78075355	78093680	0.11264	rs6565696	2.979	rs2304850	0.053004	-19.65	0.4182	41	41	enet	0.081	4.40E-135	-0.7978	0.425
SeqId_17756_69	DCTD	4	183811213	183839089	0.00563	rs66539904	2.206	rs6552631	0.000739	4.41	-0.567	72	72	enet	0.0012	0.0016	-0.7983	4.25E-01
SeqId_17748_21	TP53I3	2	24300303	24308731	0.23754	rs6756785	-2.785	rs10191964	0.26518	-43.86	-0.93	70	70	enet	0.3	0	0.7965	4.26E-01
SeqId_4328_2	BOC	3	112929850	113006306	0.07114	rs13085192	2.48	rs3856718	3.36E-02	16.16	1.174	88	88	enet	0.045	9.50E-74	0.79529	4.26E-01
SeqId_4991_12	GPC5	13	92050875	93525935	0.14702	rs79825477	-2.63	rs342706	0.1875	-36.81	0.817	66	66	enet	0.24	0	-0.794	0.427
SeqId_19488_1	PDCD6	5	271736	315089	0.03902	rs4956935	-2.521	rs56075848	5.82E-03	6.93	-0.444	39	39	enet	0.014	1.30E-23	0.79423	0.42706

SeqId_8248_222	SIGLEC14	19	52145806	52150054	0.52882	rs71358836	2.485	rs872629	0.214229	-39.37	-0.776	214	214	enet	0.34	0	0.79125	0.428799
SeqId_5671_1	CTRB1	16	75252886	75260236	0.16513	rs188133085	2.45	rs8057145	0.11474	28.92	-1.038	62	62	enet	0.21	0	-0.7903	4.29E-01
SeqId_5687_5	PRKCSH	19	11546105	11561783	0.05246	rs12983706	-2.502	rs11557488	0.02712	14.05	-0.244	33	33	enet	0.036	1.10E-59	0.79007	0.429488
SeqId_15474_7	PIN1	19	9945933	9960365	0.01415	rs144087245	2.396	rs730554	0.001159	-5.1	-0.058	43	43	enet	0.0026	8.50E-06	0.78923	0.429979
SeqId_3024_18	SERPINF2	17	1646130	1658559	0.10113	rs2957924	4.171	rs11078596	0.030707	-16.14	0.7396	121	121	enet	0.055	2.30E-91	0.7852	0.43233
SeqId_3894_15	NAGK	2	71291474	71306938	0.10153	rs12611717	1.351	rs11680831	0.12057	-29.53	0.447	33	33	enet	0.13	3.40E-227	-0.7821	4.34E-01
SeqId_3292_75	CD48	1	160648536	160681621	0.1763	rs790626	1.86	rs352684	5.78E-02	20.98	1.774	42	42	enet	0.12	4.80E-205	0.7819	0.434288
SeqId_18214_2	GCLM	1	94350755	94375012	0.05508	rs17398377	2.231	rs7521934	2.52E-02	-13.98	0.311	37	37	enet	0.054	5.50E-90	-0.7794	0.435719
SeqId_17170_15	CALCB	11	14926543	15103888	0.02739	rs4757301	1.48	rs7104596	0.033138	-15.97	0.853	30	30	enet	0.036	4.00E-59	-0.77968	4.36E-01
SeqId_8258_22	UXS1	2	106709764	106810757	0.03886	rs74770020	2.396	rs7591409	0.02703	-14.12	-1.574	44	44	enet	0.031	5.60E-52	0.777	4.37E-01
SeqId_3285_23	C1R	12	7187518	7245136	0.07994	rs7313471	0.987	rs143238956	0.080694	-2.58	-0.388	5	5	enet	0.085	7.80E-142	0.77613	0.43767
SeqId_5480_49	CCL5	17	34198495	34207797	0.05681	rs117276112	-2.442	rs2107538	0.106811	-28.09	-0.829	50	50	enet	0.11	2.50E-185	0.7744	0.4387
SeqId_11098_1	PDXK	21	45138975	45182188	0.03824	rs113597750	1.6	rs79343092	0.0136	-10.13	-0.125	28	28	enet	0.024	1.70E-39	-0.7739	0.439
SeqId_9894_13	MYO6	6	76458909	76629254	0.03649	rs80009052	-2.07	rs41269323	0.01579	-10.88	0.8788	18	18	enet	0.017	2.00E-28	-0.774	4.39E-01
SeqId_17773_26	NAPG	18	10525902	10552761	0.00417	rs71364220	1.08	rs518978	0.0031	-5.09	0.772	6	1	top1	0.0031	1.30E-06	-0.772	0.4401
SeqId_3305_6	DLL4	15	41221538	41231271	0.00881	rs11634187	2.839	rs117454282	0.00515	6.47	-0.029	20	20	enet	0.0054	2.40E-10	-0.7692	0.4418
SeqId_10940_25	SRL	16	4239375	4292081	0.02021	rs130052	3	rs9930802	0.0126	-10.1	-0.763	17	1	top1	0.013	6.90E-22	0.763	4.45E-01
SeqId_3435_53	FN1	2	216225163	216300796	0.04318	rs12694363	-2.462	rs1250258	0.03081	15.11	0.337	24	24	enet	0.034	3.30E-57	0.7643	4.45E-01
SeqId_9401_57	TRABD2A	2	85048774	85134132	0.00487	rs17025827	-2.273	rs80090251	0.004	5.66	0.763	18	1	top1	0.004	4.50E-08	0.763	4.45E-01
SeqId_13114_50	LUM	12	91496406	91505271	0.12144	rs143053515	-2.302	rs3782580	0.014368	-11.95	0.743	137	137	enet	0.054	1.80E-89	-0.76237	0.44584
SeqId_5238_26	PPIE	1	40157854	40229586	0.1455	rs146327150	-1.141	rs1046988	1.61E-01	-34.36	-0.994	43	43	enet	0.19	0	0.7623	0.445873
SeqId_10800_15	SERPINH1	11	75273101	75283828	0.05811	rs59368208	-2.181	rs599816	0.02143	12.66	0.792	62	62	enet	0.039	3.80E-65	-0.76127	4.46E-01
SeqId_4976_57	CRK	17	1323983	1366456	0.02293	rs8066044	1.762	rs12936625	0.01823	-11.61	1.375	32	32	enet	0.019	6.00E-33	-0.7596	0.44751
SeqId_5620_13	PAM	5	102089685	102365434	0.5164	rs72781852	-2.636	rs3733939	1.29E-01	-32.5	0.146	89	89	enet	0.32	0	-0.75717	0.44895
SeqId_4866_59	NTRK2	9	87283466	87641969	0.02317	rs4381025	-3.954	rs11140774	0.00558	-6.87	-2.334	98	98	enet	0.012	2.40E-21	0.7571	4.49E-01
SeqId_15544_25	KLK14	19	51580752	51587502	0.16196	rs190552	-1.843	rs11666870	0.029998	14.88	-0.8212	104	104	enet	0.11	4.70E-178	0.75535	0.450038
SeqId_3168_8	ADAMTS5	21	28290241	28339407	0.27228	rs147421162	-2.06	rs2830585	0.22635	-40.42	0.922	47	47	enet	0.27	0	-0.7542	0.451
SeqId_16318_12	ACVRL1	12	52300692	52317145	0.06622	rs303765	2.964	rs78197611	0.039205	17.06	1.894	42	42	enet	0.05	2.90E-83	0.75211	0.45199
SeqId_18319_7	PDHX	11	34937376	35042138	0.07716	rs56410006	-2.034	rs2915221	0.046208	-18.83	-1.032	143	143	enet	0.09	3.20E-150	0.75008	4.53E-01
SeqId_10672_75	SFTPB	2	85884437	85895864	0.41198	rs77433347	2.708	rs1130866	0.13379	-31.1	-1.046	68	68	enet	0.14	9.00E-244	0.7506	4.53E-01
SeqId_15457_14	ANPEP	15	90328126	90358633	0.00994	rs79483623	2.368	rs1042499	0.00495	6.2	-0.75	24	1	top1	0.005	1.30E-09	-0.75	0.4533
SeqId_6496_60	DLK1	14	101192042	101204561	0.08558	rs75741145	3.308	rs4905998	0.094834	26.48	-0.1	60	60	enet	0.11	3.70E-192	-0.7472	4.55E-01
SeqId_4435_66	ENPP7	17	77704705	77716018	0.2973	rs141015675	2.146	rs8077074	0.370276	52.08	0.498	75	75	enet	0.42	0	0.7463	0.45548
SeqId_14100_63	C1QC	1	22970126	22974603	0.29541	rs139801860	-2.378	rs75380810	6.90E-02	22.37	-0.53	159	159	enet	0.19	0	0.7455	0.455957
SeqId_9201_13	TAGLN2	1	159887897	159895297	0.04108	rs8192602	-2.002	rs2789422	2.77E-02	-14.29	0.113	46	46	enet	0.029	1.00E-48	-0.7451	0.45623
SeqId_13112_179	FSTL1	3	120111140	120169840	0.03726	rs146626560	-3.05	rs916510	1.06E-02	-9.03	0.503	87	87	enet	0.018	2.00E-30	-0.74375	4.57E-01
SeqId_8336_267	GRAMD1C	3	113547029	113666021	0.08358	rs4339084	-1.76	rs61634901	4.94E-02	19.01	-0.309	24	24	enet	0.061	2.60E-100	-0.7441	4.57E-01
SeqId_6649_51	NTN1	17	8924827	9147317	0.13331	rs77511930	-2.757	rs9897200	0.087398	-25.17	0.891	78	78	enet	0.16	1.10E-277	-0.7431	0.45741
SeqId_11510_31	APOL1	22	36649117	36663576	0.12171	rs9607318	-2.38	rs10854688	0.07552	23.54	-0.804	57	57	enet	0.1	6.40E-169	-0.7425	0.45777
SeqId_4886_3	CCL7	17	32597240	32599261	0.17405	rs2530797	-1.911	rs3138037	0.118813	-29.31	0.109	35	35	enet	0.18	2.50E-307	-0.7414	0.45845
SeqId_6897_38	B3GAT3	11	62382768	62389626	0.06405	rs11231079	-2.26	rs7122950	0.068374	-22.62	0.151	47	47	enet	0.074	1.70E-123	-0.73991	4.59E-01
SeqId_19277_4	TSTD1	1	161007421	161008767	0.26994	rs74879368	2.231	rs11580071	1.76E-01	35.71	0.376	52	52	enet	0.24	0	0.7403	0.459099
SeqId_2731_29	POR	7	75528518	75616173	0.01521	rs239958	-1.944	rs59882870	0.00733	7.61	1.362	29	29	enet	0.012	4.40E-21	0.7363	0.461556
SeqId_7928_183	TPST1	7	65670304	65885530	0.04976	rs12539696	3.777	rs313829	0.054094	20.69	0.022	56	56	enet	0.096	4.80E-161	-0.7343	0.462788
SeqId_3367_8	FETUB	3	186353758	186370930	0.25019	rs13070687	-2.41	rs5030103	4.16E-02	-19.72	1.341	88	88	enet	0.14	3.50E-235	-0.73399	4.63E-01
SeqId_8345_27	GPX7	1	53068043	53074723	0.13396	rs835352	1.169	rs6669154	1.43E-01	-32.23	-0.654	16	16	enet	0.17	3.10E-289	0.731	0.464785
SeqId_11514_196	CD59	11	33724556	33758025	0.07006	rs145951489	-1.752	rs831630	0.066144	-22.11	0.33	44	44	enet	0.085	4.90E-142	-0.72918	4.66E-01
SeqId_11212_7	TXNDC5	6	7881750	7911021	0.08261	rs111331197	1.98	rs111331197	0.03401	15.78	1.981	28	28	enet	0.06	1.10E-98	0.73	4.66E-01
SeqId_15385_116	FABP2	4	120238405	120243293	0.17634	rs7665125	-2.432	rs1799883	0.325812	48.49	-0.645	37	37	enet	0.33	0	-0.7275	4.67E-01
SeqId_15304_1	REG3A	2	79384129	79386879	0.06516	rs1261238	-2.282	rs116652994	0.02359	13.12	-0.438	41	41	enet	0.048	3.40E-79	0.7219	4.70E-01
SeqId_9910_9	MTAP	9	21802635	21937650	0.00287	rs1345026	0.723	rs1345026	0.00187	-5.15	0.723	4	1	top1	0.0019	0.00014	-0.723	4.70E-01
SeqId_18183_3	LDLRAP1	1	25870097	25895377	0.12575	rs76148397	-2.519	rs4523506	6.09E-02	-21.29	-1.513	33	33	enet	0.099	1.10E-165	0.7218	0.470443
SeqId_13930_3	APOBEC3G	22	39473072	39483748	0.04197	rs1043395	2.03	rs17537581	0.01773	-12.03	0.195	35	35	enet	0.032	7.00E-54	0.7217	0.47046

SeqId_2614_28	EFNA4	1	155036213	155042029	0.01414	rs9426832	-1.977	rs3806256	3.65E-03	6.1	0.719	12	1	top1	0.0037	1.70E-07	0.719	0.472141
SeqId_19448_104	UPP1	7	48128225	48148333	0.01051	rs3763504	0.989	rs3763505	0.008189	8.58	0.716	10	1	top1	0.0082	8.20E-15	0.716	0.473991
SeqId_3004_67	PDCD1LG2	9	5510531	5571282	0.2984	rs117058003	2.422	rs16923189	0.19427	37.44	1.458	36	36	enet	0.28	0	0.7168	4.74E-01
SeqId_5609_92	FAM19A5	22	48885365	49246724	0.01624	rs113299767	3.06	rs5768649	0.00184	-6.42	0.362	33	33	enet	0.0061	2.10E-11	0.7141	0.47515
SeqId_19606_28	IHH	2	219919146	219925643	0.0052	rs55838820	0.758	rs55836193	0.00205	-4.66	0.713	7	1	top1	0.002	7.20E-05	-0.713	4.76E-01
SeqId_6255_74	CPXM1	20	2774720	2781273	0.4105	rs214808	-2.45	rs11697820	0.245067	42.06	1.21474	90	90	enet	0.36	0	0.7112	0.47699
SeqId_16057_6	IGF2R	6	160390114	160534539	0.34715	rs146785976	2.96	rs2282140	0.17549	35.59	-0.945	82	82	enet	0.29	0	-0.71	4.77E-01
SeqId_10666_7	GNPTG	16	1401932	1415738	0.1391	rs35903896	-2.17	rs6600137	0.08929	-25.45	1.14	59	59	enet	0.13	4.10E-216	-0.7094	4.78E-01
SeqId_17138_8	GSTA1	6	52656169	52668658	0.1122	rs61444306	1.21	rs4715316	0.26096	43.6	-0.628	64	64	enet	0.28	0	-0.71	4.78E-01
SeqId_15644_1	BTD	3	15642848	15763818	0.35051	rs9855575	2.51	rs71627145	1.58E-01	-34.12	-0.162	108	108	enet	0.26	0	0.70734	4.79E-01
SeqId_16753_46	COL6A2	21	47518026	47552762	0.18107	rs148946852	-2.3	rs35548026	0.04705	-18.56	-0.579	73	73	enet	0.086	6.70E-143	0.7067	0.48
SeqId_13676_46	INHBB	2	121103712	121109379	0.13809	rs56302060	1.912	rs17050272	0.08664	25.11	0.724	29	29	enet	0.089	2.80E-148	0.7048	4.81E-01
SeqId_19187_21	STAMBP	2	74056043	74100786	0.01159	rs7419346	-2.765	rs77000353	0.00392	5.82	0.704	25	25	enet	0.0053	3.90E-10	-0.7046	4.81E-01
SeqId_3348_49	BMP1	8	22022653	22069839	0.02845	rs146424305	1.487	rs1126931	0.008018	-8.93	0.4515	25	25	enet	0.015	2.90E-26	-0.7011	0.483247
SeqId_5465_32	HS6ST1	2	128994290	129076442	0.0792	rs190342391	3.255	rs4662790	0.02909	-14.99	0.071	99	99	enet	0.054	1.20E-88	-0.6997	4.84E-01
SeqId_19377_14	OLFM2	19	9964394	10047191	0.32232	rs34561079	-2.464	rs62104305	0.313106	47.61	0.605	70	70	enet	0.33	0	0.69818	0.485067
SeqId_13657_2	PNKP	19	50363139	50381608	0.01334	rs117347783	-1.54	rs2290774	0.003347	-5.93	-0.086	17	17	enet	0.007	5.70E-13	0.69726	0.485643
SeqId_19279_42	RBP1	3	139236276	139258671	0.01923	rs16849559	-2.89	rs2071387	1.09E-02	9.37	-0.514	54	54	enet	0.014	1.60E-23	0.69605	4.86E-01
SeqId_5593_11	PDIA5	3	122785872	122944074	0.36847	rs115111146	2.17	rs3804749	4.82E-01	-58.98	-0.709	102	102	enet	0.5	0	0.69614	4.86E-01
SeqId_8900_28	NEO1	15	73344051	73597546	0.07191	rs117366855	-2.506	rs13379875	0.05916	-20.7	0.501	34	34	enet	0.08	4.20E-133	-0.6957	0.4866
SeqId_5400_52	LEPR	1	65886335	66107242	0.17	rs75148473	1.956	rs2376018	4.54E-01	-57.24	0.615	74	74	enet	0.47	0	-0.6924	0.488717
SeqId_3028_36	CCL23	17	34340096	34345009	0.41392	rs113688187	2.983	rs712048	0.17946	-36.04	1.026	127	127	enet	0.3	0	-0.6922	0.48884
SeqId_5630_48	CD300A	17	72462538	72480933	0.23383	rs62063449	2.06	rs2272111	0.245113	-42.06	-0.641	80	80	enet	0.29	0	0.691	0.48959
SeqId_7891_45	UGT1A6	2	234600253	234681946	0.06011	rs17868299	-1.934	rs887829	0.06198	-21.31	0.771	39	39	enet	0.079	8.00E-131	-0.6861	4.93E-01
SeqId_3651_50	KDR	4	55944648	55991762	0.42042	rs10434440	2.958	rs34231037	0.072418	-22.98	-0.564	67	67	enet	0.16	4.70E-268	0.6856	4.93E-01
SeqId_14116_129	S100A4	1	153516089	153522612	0.01561	rs150059283	-3.009	rs79399329	4.38E-03	6.82	-1.172	45	45	enet	0.0094	8.20E-17	0.6848	0.493458
SeqId_15602_43	IL6R	1	154377669	154441926	0.38332	rs41308403	-2.763	rs4129267	5.28E-01	61.74	1.212	49	49	enet	0.57	0	0.6809	0.495964
SeqId_4125_52	AGER	6	32148745	32152099	0.1132	rs204999	-1.41	rs2070600	0.04918	-18.86	-0.681	17	1	top1	0.049	2.70E-81	0.681	4.96E-01
SeqId_8428_102	NTM	11	131240373	132206716	0.22768	rs7938687	-2.817	rs2511781	0.187727	36.83	-0.515	121	121	enet	0.25	0	-0.67864	4.97E-01
SeqId_5430_66	SIRPA	20	1874813	1921238	0.29157	rs35556047	-2.39	rs6136376	0.524266	-61.53	-0.671	51	51	enet	0.53	0	0.6745	0.49997
SeqId_17761_2	NUDT5	10	12207329	12238143	0.00659	rs7895525	1.93	rs7099650	0.00191	-5.99	-0.182	8	8	enet	0.0042	2.40E-08	0.674126	0.5002
SeqId_4487_1	FGF7	15	49715434	49780972	0.09684	rs143076571	-2.127	rs7161744	0.01258	-10.97	-0.124	46	46	enet	0.038	1.40E-62	0.6737	0.5005
SeqId_11369_23	ADH5	4	99992129	100009952	0.06273	rs72681991	-1.55	rs62325239	0.069219	-22.43	-0.366	66	66	enet	0.091	2.00E-152	0.6692	5.03E-01
SeqId_3166_92	CD33	19	51728320	51747115	0.5964	rs75883262	-3.236	rs3865444	0.393413	-53.32	-0.319	149	149	enet	0.5	0	0.66898	0.503508
SeqId_5121_3	SEMA6B	19	4542605	4581788	0.02603	rs10415374	-1.845	rs4353572	0.009394	8.38	0.7961	46	46	enet	0.01	3.10E-18	0.66827	0.503963
SeqId_16763_11	LECT2	5	135257968	135290672	0.50447	rs143103412	-1.945	rs2526147	2.31E-01	-40.92	-0.795	79	79	enet	0.43	0	0.66714	0.50468
SeqId_18408_26	ARF4	3	57557090	57583947	0.01704	rs144612862	-1.42	rs141818286	5.25E-03	6.5	0.307	10	10	enet	0.0081	1.30E-14	0.66596	5.05E-01
SeqId_12534_10	CALCOCO2	17	46908380	46943884	0.02518	rs688945	-2.13	rs318100	0.020006	-12.47	-0.42	15	15	enet	0.023	2.70E-38	0.6641	0.50662
SeqId_10948_14	PLD3	19	40854363	40886346	0.03145	rs535840	2.688	rs4803321	0.020549	12.41	-0.664	50	1	top1	0.021	1.20E-34	-0.664	0.50669
SeqId_8970_9	RIPK2	8	90770034	90803292	0.00492	rs13267411	1.224	rs34211510	0.004766	6.2	0.664	5	1	top1	0.0048	2.60E-09	0.664	0.50669
SeqId_5353_89	IL1RN	2	113864791	113891593	0.07229	rs2515391	-2.246	rs55709272	0.06882	-22.37	0.107	81	81	enet	0.12	4.50E-197	0.663	5.07E-01
SeqId_6715_63	ALPPL2	2	233271554	233275424	0.0988	rs11688622	2.446	rs35458538	0.03769	16.62	0.949	45	45	enet	0.066	2.00E-109	0.6639	5.07E-01
SeqId_11377_19	ADH7	4	100333418	100356667	0.07908	rs114002582	-2.321	rs17529509	0.035284	-16.11	0.855	83	83	enet	0.048	2.50E-79	-0.6607	5.09E-01
SeqId_6927_7	NDST1	5	149865381	149937773	0.05693	rs11749324	1.453	rs11746198	2.39E-02	13.43	-0.347	33	33	enet	0.04	5.20E-67	-0.66014	0.50917
SeqId_11615_16	DAPP1	4	100737984	100791347	0.09032	rs7666555	1.864	rs3822103	0.058752	-20.68	0.108	80	80	enet	0.07	7.30E-117	-0.6572	5.11E-01
SeqId_8307_47	C11orf68	11	65684281	65686533	0.07142	rs565972	2.236	rs554169857	0.031177	15.05	0.266	26	26	enet	0.034	2.60E-56	0.65553	5.12E-01
SeqId_4874_3	ANG	14	21152336	21167130	0.29317	rs72672714	-3.276	rs4982325	0.136212	32.27	-1.066	98	98	enet	0.31	0	-0.6551	5.12E-01
SeqId_18243_9	CMPK1	1	47799462	47844511	0.10147	rs1249089	-2.981	rs35687416	3.77E-02	-16.54	-0.628	54	54	enet	0.052	1.90E-85	0.6547	0.512686
SeqId_11656_110	EVL	14	100437786	100610573	0.02878	rs71422786	2.89	rs34612764	0.008856	9.26	0.409	68	68	enet	0.021	4.80E-36	0.6546	5.13E-01
SeqId_9357_4	CREG1	1	167498354	167523042	0.05114	rs113928789	1.49	rs7513428	2.85E-02	-14.37	-0.692	19	19	enet	0.035	6.30E-58	0.6516	0.514669
SeqId_4374_45	GDF15	19	18485541	18499986	0.28223	rs76580597	1.383	rs16982345	0.176533	35.78	-0.892	46	46	enet	0.27	0	-0.64793	0.517032
SeqId_12812_25	ACYP2	2	54197975	54532437	0.07498	rs10427395	2.636	rs11125524	0.07379	23.12	-0.443	65	65	enet	0.11	8.70E-179	-0.6468	5.18E-01
SeqId_11257_1	QDPR	4	17461884	17513829	0.27052	rs187658266	-2.212	rs7661303	0.200076	-38.01	0.622	67	67	enet	0.26	0	-0.646	5.18E-01

SeqId_17447_52	SFRP4	7	37945534	38065297	0.07458	rs17171306	-2.361	rs2598109	0.029307	-14.92	0.72	55	55	enet	0.049	1.10E-80	-0.6459	0.518332
SeqId_17148_7	BLVRB	19	40953672	40971671	0.08382	rs111533484	-2.666	rs184723794	0.026996	-14.13	1.873	56	56	enet	0.032	6.10E-53	-0.64341	0.519959
SeqId_5532_53	FGFR1	8	38257733	38326352	0.03695	rs6985355	1.829	rs11997244	0.007566	-8.88	0.838	42	42	enet	0.028	7.50E-47	-0.6397	0.522386
SeqId_15503_15	LEFTY2	1	226124298	226129083	0.0791	rs4653426	2.101	rs360078	4.83E-02	-19.06	0.388	59	59	enet	0.066	7.60E-109	-0.6372	0.523973
SeqId_3060_43	C9	5	39284242	39424970	0.11258	rs41302832	-2.749	rs696766	2.22E-02	-12.71	-1.475	135	135	enet	0.067	3.90E-110	0.63656	0.52441
SeqId_11636_33	TAGLN2	1	159887897	159895297	0.04534	rs73021687	-2.183	rs2789422	3.13E-02	-15.09	0.113	36	36	enet	0.033	5.10E-54	-0.6346	0.525699
SeqId_7968_15	CRTAM	11	122709208	122743351	0.11801	rs140345204	-1.999	rs2370794	0.040466	17.16	-0.078	34	34	enet	0.08	3.80E-132	0.63269	5.27E-01
SeqId_13929_27	CROT	7	86974980	87029110	0.08529	rs34110100	1.753	rs31659	0.06532	22.33	0.686	23	23	enet	0.078	1.60E-129	0.6314	0.527761
SeqId_5355_69	TNFSF14	19	6661264	6670599	0.00851	rs74585960	3.025	rs413141	0.000351	-4.85	-0.283	53	53	enet	0.0048	2.30E-09	0.63046	0.528396
SeqId_3799_11	CA3	8	86285665	86361269	0.04038	rs74603761	-2.686	rs1543852	0.03604	16.76	1.061	29	29	enet	0.044	1.90E-73	0.628	0.529973
SeqId_6472_40	PMEL	12	56347889	56367101	0.02901	rs3213122	0.628	rs3213122	0.027629	14.4	0.628	5	1	top1	0.028	4.60E-46	0.628	0.53
SeqId_8606_39	GPNMB	7	23275586	23314727	0.17268	rs1542608	3.005	rs2268748	0.036597	16.29	1.094	98	98	enet	0.067	3.40E-111	0.6263	0.531132
SeqId_2212_69	PLAT	8	42032236	42065227	0.00992	rs2020921	-0.796	rs77346091	0.011275	-9.23	-0.623	4	1	top1	0.011	9.30E-20	0.623	0.533285
SeqId_8409_3	RSPO2	8	108911544	109095870	0.00231	rs78052592	-0.876	rs640068	0.002116	-4.47	0.622	10	1	top1	0.0021	5.50E-05	-0.622	0.533942
SeqId_8842_16	GRAMD1C	3	113547029	113666021	0.19339	rs4339084	-1.76	rs61634901	1.45E-01	32.48	-0.309	43	43	enet	0.19	0	-0.62126	5.34E-01
SeqId_11431_235	RECQL	12	21621844	21654603	0.03608	rs76497895	3.424	rs74626198	0.005838	-7.7	0.648	78	78	enet	0.02	8.70E-34	0.62181	0.53407
SeqId_8364_74	UST	6	149068166	149398126	0.04723	rs1768328	-2.46	rs7764228	0.009	-9.26	0.0494	86	86	enet	0.033	2.50E-55	-0.621	5.35E-01
SeqId_12381_26	CBR1	21	37442239	37445464	0.1335	rs2835230	2.82	rs16993864	0.08399	-24.73	0.663	28	28	enet	0.093	3.50E-155	-0.6184	0.536
SeqId_3074_6	LBP	20	36974885	37005655	0.40262	rs1739655	2.61	rs2232613	0.216406	-39.54	-0.76	64	64	enet	0.28	0	-0.6182	0.53643
SeqId_19446_1	GMPR2	14	24701628	24708448	0.17871	rs144673701	-2.496	rs34354104	0.079308	-23.95	-0.463	54	54	enet	0.09	1.00E-149	0.6176	5.37E-01
SeqId_4337_49	CRP	1	159682079	159684379	0.06035	rs876538	-1.929	rs2794520	3.24E-02	-15.46	-0.961	36	36	enet	0.047	2.20E-78	0.6166	0.53751
SeqId_3773_15	TEK	9	27109139	27230172	0.15471	rs10967916	2.29	rs35030851	0.06059	21.06	0.399	35	35	enet	0.11	4.70E-179	-0.6155	5.38E-01
SeqId_17836_17	S100A16	1	153579362	153585621	0.06095	rs188550783	3.417	rs9729773	4.84E-02	19.33	0.146	45	45	enet	0.057	1.60E-94	0.615	0.538557
SeqId_11634_32	RGS10	10	121259329	121302231	0.01567	rs117261018	-2.5	rs61874690	0.001397	5.39	-0.621	39	39	enet	0.0035	3.50E-07	-0.613159	0.5398
SeqId_8786_6	FAM171B	2	187558787	187630686	0.03568	rs17334436	-1.487	rs12621915	0.02092	-12.37	-0.667	42	42	enet	0.028	1.70E-46	0.6134	5.40E-01
SeqId_14158_17	ANXA5	4	122589101	122618150	0.00637	rs10034975	-1.642	rs36177180	0.00096	-4.77	0.094	11	11	enet	0.0015	0.00056	-0.6116	5.41E-01
SeqId_4535_50	BST1	4	15704688	15739936	0.29954	rs34921119	-2.387	rs6842052	0.392827	-53.28	0.115	103	103	enet	0.48	0	-0.6106	5.41E-01
SeqId_18342_2	PSAT1	9	80912041	80945009	0.06013	rs7858423	2.226	rs1929402	0.04883	19	-0.194	18	18	enet	0.062	2.80E-103	0.6118	5.41E-01
SeqId_11142_11	ANGPTL1	1	178818670	178840212	0.12965	rs146233229	-1.57	rs10913568	9.44E-02	-26.19	0.738	89	89	enet	0.17	3.10E-287	-0.6107	0.541384
SeqId_11211_7	TBCE	1	235491885	235615756	0.16991	rs113752657	-2.543	rs4469707	7.33E-02	-23.07	0.478	89	89	enet	0.15	3.70E-261	0.6105	0.54151
SeqId_12641_3	INPP5B	1	38326369	38412729	0.12002	rs150788669	1.753	rs112836202	2.00E-01	-38.17	-1.134	71	71	enet	0.25	0	0.6076	0.543444
SeqId_6713_4	LRP11	6	150139893	150185495	0.21424	rs111576955	-2.36	rs1889471	0.49903	60.28	0.6212	88	88	enet	0.56	0	0.607	5.44E-01
SeqId_2647_66	GDI2	10	5807186	5884095	0.07521	rs12722563	-2.77	rs55913768	0.097712	-26.73	-1.227	79	79	enet	0.13	3.20E-212	0.605949	0.5445
SeqId_8269_327	ARSK	5	94890805	94940806	0.01586	rs7737930	-1.763	rs956274	8.19E-03	-7.78	0.949	17	17	enet	0.0087	1.40E-15	-0.60473	0.54536
SeqId_8932_1	ENTPD6	20	25176329	25208711	0.05151	rs78827294	2.64	rs6050446	0.018226	12.13	0.956	44	44	enet	0.019	1.60E-32	0.6045	0.54548
SeqId_4559_64	KYNU	2	143635226	143813402	0.12654	rs35055777	1.804	rs112211045	0.22066	-39.97	-0.704	59	59	enet	0.23	0	0.6034	5.46E-01
SeqId_3516_60	CXCL12	10	44865613	44881941	0.03624	rs79775926	1.85	rs1023264	0.012705	-9.8	-0.116	24	24	enet	0.021	1.10E-34	-0.602091	0.5471
SeqId_5491_12	SPOCK2	10	73818792	73848790	0.0272	rs117273499	1.58	rs3312	0.029813	15.13	0.822	28	28	enet	0.03	5.70E-50	0.601625	0.5474
SeqId_2741_22	SIGLEC6	19	52021073	52035110	0.24169	rs145673783	3.116	rs4802807	0.175583	-35.67	-1.393	98	98	enet	0.29	0	0.60025	0.548341
SeqId_12662_82	ECH1	19	39306062	39322481	0.13938	rs117807208	-1.769	rs2229259	0.095574	26.32	-0.599	10	1	top1	0.096	9.30E-160	-0.599	0.549173
SeqId_6409_57	ADGRF5	6	46820259	46922680	0.1501	rs114374752	-1.94	rs586024	0.23165	40.97	0.249	59	59	enet	0.26	0	0.596	5.51E-01
SeqId_10521_10	MXRA8	1	1288069	1297157	0.00991	rs17162854	1.362	rs1240713	4.87E-03	-7.46	0.006	26	26	enet	0.0078	3.80E-14	-0.5955	0.551541
SeqId_16900_29	MDGA1	6	37598455	37667082	0.57444	rs11755738	2.2	rs9349050	0.50047	-60.11	0.434	173	173	enet	0.55	0	-0.595	5.52E-01
SeqId_15620_4	NLGN1	3	173114074	174004434	0.05058	rs11709498	-2.43	rs497383	2.04E-02	12.35	0.385	49	49	enet	0.036	4.90E-60	-0.5939	5.53E-01
SeqId_17821_20	NMT2	10	15144583	15210692	0.05676	rs11592551	-1.82	rs11259539	0.024348	-13.5	0.73	20	20	enet	0.038	1.20E-62	-0.592315	0.5536
SeqId_5091_28	LILRB2	19	54777666	54785039	0.33336	rs11668189	-1.602	rs383925	0.372729	51.87	-0.692	47	47	enet	0.41	0	-0.59155	0.554152
SeqId_3535_84	DKK1	10	54074041	54077802	0.1963	rs1733725	-2.45	rs11003047	0.055424	20.94	0.514	77	77	enet	0.14	8.80E-243	0.591103	0.5545
SeqId_6576_1	ART4	12	14978503	14996460	0.16954	rs2233623	1.718	rs7311924	0.444401	56.72	0.557	87	87	enet	0.45	0	0.59013	0.5551
SeqId_5648_28	CTRB2	16	75238001	75241059	0.38782	rs4888354	-1.89	rs1808427	0.28264	-45.29	-1.173	56	56	enet	0.42	0	0.5882	5.56E-01
SeqId_5404_53	TNFRSF21	6	47199268	47277641	0.10755	rs1884046	2.08	rs6458555	0.03594	16.25	0.932	92	92	enet	0.071	1.90E-118	0.584	5.59E-01
SeqId_13717_15	FCN2	9	137772656	137779369	0.25372	rs3124956	2.451	rs11103563	0.19802	-37.89	-0.346	96	96	enet	0.29	0	-0.5835	5.60E-01
SeqId_10605_22	APMAP	20	24943561	24973387	0.02571	rs35378778	1.99	rs6036977	0.009598	-8.88	-0.526	21	21	enet	0.014	2.20E-24	-0.58	0.56189
SeqId_6234_74	VIT	2	36923833	37041935	0.30328	rs78196400	-3.041	rs1468810	0.12902	-30.58	-0.201	111	111	enet	0.23	0	0.5793	5.62E-01

SeqId_12469_19	MAPRE1	20	31407760	31438211	0.08465	rs6058900	2.58	rs414049	0.111524	-28.5	0.116	29	29	enet	0.13	3.50E-223	-0.5762	0.56451
SeqId_17676_13	TRIP10	19	6737936	6751541	0.06296	rs2018815	2.314	rs8106212	0.008587	7.98	-0.196	80	80	enet	0.024	1.50E-40	0.57403	0.56595
SeqId_19289_29	UROD	1	45476622	45481247	0.09849	rs1152017	-1.205	rs12749939	2.38E-01	-41.56	-0.532	44	44	enet	0.24	0	0.5731	0.566555
SeqId_13427_66	MAN1C1	1	25943282	26112697	0.0733	rs181237797	2.291	rs11247595	4.33E-02	-17.72	0.211	50	50	enet	0.067	1.10E-111	-0.5714	0.567743
SeqId_15499_11	ATRN	20	3451665	3631765	0.25444	rs75221813	2.99	rs118065662	0.092487	-25.88	-0.833	69	69	enet	0.16	6.60E-284	0.5687	0.56957
SeqId_12563_2	TNFAIP8	5	118604387	118735383	0.07231	rs79013506	-1.617	rs1035376	5.05E-02	19.14	0.791	61	61	enet	0.074	3.40E-123	0.56799	0.57004
SeqId_10603_1	HTN3	4	70894172	70902255	0.01781	rs17644018	-1.609	rs78169960	0.0117	-9.35	-0.013	16	16	enet	0.013	2.10E-22	-0.5652	5.72E-01
SeqId_5605_77	MFNG	22	37865101	37882431	0.01482	rs56272819	1.44	rs8192548	0.01514	-11.15	-0.308	13	13	enet	0.016	2.50E-27	0.5639	0.57283
SeqId_5090_49	LILRB1	19	55128612	55149473	0.1176	rs79648432	2.264	rs2098953	0.225319	-40.34	1.052	67	67	enet	0.3	0	-0.56308	0.573382
SeqId_15471_29	PNLIPRP2	10	118380465	118404654	0.28711	rs7071432	1.77	rs4751995	0.36881	51.59	-0.543	23	23	enet	0.37	0	-0.563106	0.5734
SeqId_17799_9	PGLS	19	17622445	17632097	0.11908	rs28400331	3.146	rs73020480	0.055315	20.13	1.326	63	63	enet	0.079	1.70E-130	0.56229	0.573918
SeqId_3186_2	C2	6	31865562	31913449	0.22122	rs3094738	-3.68	rs115204832	0.03112	-16.56	-0.7613	137	137	enet	0.12	2.10E-204	-0.558	5.77E-01
SeqId_6551_94	SERPINA12	14	94953611	94984181	0.21298	rs149206929	-2.767	rs4900236	0.098191	26.78	0.69	55	55	enet	0.15	2.40E-258	0.5563	5.78E-01
SeqId_18896_23	HS6ST3	13	96742361	97491816	0.00977	rs61968033	-1.34	rs12860877	0.00656	7.68	-0.431	21	21	enet	0.0082	7.40E-15	-0.554	0.5798
SeqId_9337_43	TAC1	7	97361396	97369784	0.04265	rs6950795	2.302	rs2072099	0.019149	-12.12	1.074	53	53	enet	0.034	3.80E-57	-0.5535	0.57994
SeqId_8288_27	APOH	17	64208151	64252643	0.37179	rs76413180	-2.462	rs1801689	0.148047	-32.7	-0.92	53	53	enet	0.17	2.70E-294	0.5525	0.58058
SeqId_4294_16	SPHK1	17	74372665	74383941	0.0322	rs140293715	-3.363	rs12941069	0.010233	8.77	0.1387	76	76	enet	0.015	5.40E-26	0.5514	0.58137
SeqId_7156_2	FUT10	8	33228342	33330664	0.23747	rs112753196	-2.354	rs2732317	0.297514	-46.34	0.455	91	91	enet	0.41	0	-0.5487	0.583235
SeqId_19143_38	CYB5R2	11	7686331	7698453	0.14049	rs4078281	-2.335	rs11041504	0.129922	30.75	0.164	95	95	enet	0.17	1.40E-287	0.54671	5.85E-01
SeqId_9940_35	DUSP28	2	241499471	241504673	0.09634	rs147468658	-2.591	rs112548426	0.04681	-18.57	0.394	49	49	enet	0.075	3.50E-125	0.5447	5.86E-01
SeqId_4961_17	ANXA2	15	60639333	60695082	0.04041	rs62003317	-2.674	rs12440452	0.02174	-13.49	0.026	43	43	enet	0.04	1.30E-65	0.5444	0.5861
SeqId_15363_32	APOA5	11	116660083	116663136	0.15951	rs10750096	1.95	rs35120633	0.21183	39.14	0.441	17	17	enet	0.22	0	0.54095	5.89E-01
SeqId_2968_61	TNFSF15	9	117546932	117568319	0.01527	rs10114470	2.094	rs56339337	0.00377	-6.71	-0.198	36	36	enet	0.0091	2.50E-16	0.5404	5.89E-01
SeqId_13969_24	KPNA6	1	32573657	32642164	0.03535	rs72664935	2.136	rs72666741	3.99E-02	17.09	-0.54	19	1	top1	0.04	4.50E-66	-0.54	0.589197
SeqId_18819_21	PPIC	5	122358945	122372420	0.45101	rs145479776	-3.008	rs17388251	2.71E-01	-44.22	-0.307	106	106	enet	0.36	0	0.53917	0.58977
SeqId_9175_48	DSCAM	21	41382926	42219084	0.02924	rs79908646	1.69	rs78111814	0.01894	11.89	0.589	41	41	enet	0.023	2.60E-39	0.5389	0.59
SeqId_17450_51	HARS	5	140052620	140071122	0.05935	rs186566632	-1.784	rs112352450	7.67E-03	7.68	-0.662	22	22	enet	0.012	6.00E-21	-0.53833	0.59035
SeqId_6227_1	KLK10	19	51515995	51523431	0.31779	rs116958492	3.336	rs2569454	0.210947	39.05	1.7405	77	77	enet	0.32	0	0.53812	0.590498
SeqId_4430_44	COLEC11	2	3642422	3692234	0.28181	rs112087264	2.24	rs6542680	0.13339	-31.11	-1.469	81	81	enet	0.18	4.5e-312	0.5368	5.91E-01
SeqId_19578_19	DOK2	8	21766384	21771201	0.1036	rs11783414	2.692	rs17615788	0.016655	12.07	1.15	111	111	enet	0.061	4.30E-101	-0.5365	0.591582
SeqId_19124_9	UBLCP1	5	158690298	158713044	0.00783	rs1363561	0.914	rs71593333	1.10E-02	-9.2	-0.533	12	1	top1	0.011	2.20E-19	0.533	0.59403
SeqId_14121_24	TNFRSF10D	8	22993101	23021540	0.00818	rs73222535	-1.921	rs4460371	0.004982	-6.16	0.0824	12	12	enet	0.0051	6.60E-10	-0.5317	0.594904
SeqId_5107_7	NOTCH1	9	139388885	139440500	0.03739	rs79265255	-2.883	rs9411207	0.01574	10.73	0.667	32	32	enet	0.027	1.20E-45	0.5294	5.97E-01
SeqId_8958_51	CHL1	3	238446	451100	0.07969	rs145183044	2.54	rs990284	4.27E-02	-17.68	1.579	90	90	enet	0.082	4.20E-136	-0.52664	5.98E-01
SeqId_19557_3	KLB	4	39408550	39453153	0.34112	rs151161244	-2.389	rs13112140	0.183892	-36.47	0.073	90	90	enet	0.31	0	-0.5276	5.98E-01
SeqId_10391_1	ANGPTL3	1	63063191	63071984	0.02604	rs7555577	0.615	rs10889333	4.97E-02	-19.2	-0.299	26	26	enet	0.055	2.50E-91	0.5262	0.598718
SeqId_16907_3	CADM2	3	85008140	86123579	0.02	rs112333528	1.19	rs9880919	6.55E-03	7.89	0.558	23	23	enet	0.0078	3.20E-14	0.5252	5.99E-01
SeqId_19238_12	GLUL	1	182347233	182361341	0.00748	rs594066	-2.108	rs61805076	4.99E-03	6.47	0.525	6	1	top1	0.005	1.10E-09	0.525	0.599583
SeqId_8983_7	GOLM1	9	88641061	88715088	0.04087	rs17430361	2.177	rs138447426	0.0181	11.59	0.029	43	43	enet	0.025	7.90E-42	0.5179	6.05E-01
SeqId_2654_19	TNFRSF1A	12	6437923	6451280	0.02826	rs113445398	3.326	rs1800693	0.017409	-11.41	-0.516	20	1	top1	0.017	1.30E-29	0.516	0.60585
SeqId_4437_56	ENTPD5	14	74424713	74486102	0.13505	rs150999504	-2.794	rs73301475	0.166056	-34.85	0.144	134	134	enet	0.2	0	-0.5161	6.06E-01
SeqId_6557_50	LRRC15	3	194075978	194090472	0.1651	rs71316299	-2.35	rs923930	1.38E-01	31.69	-0.102	52	52	enet	0.2	0	-0.51626	6.06E-01
SeqId_2697_7	PF4	4	74846528	74847744	0.02601	rs147841901	-2.271	rs199877023	0.016767	-11.32	-0.133	30	30	enet	0.02	3.40E-33	0.5154	6.06E-01
SeqId_19523_215	PARK7	1	8014351	8045565	0.11256	rs228684	-1.426	rs17523802	1.79E-01	36.04	-0.63	29	29	enet	0.21	0	-0.5156	0.606148
SeqId_9470_15	METTL24	6	110565143	110679552	0.05212	rs62420292	-3.14	rs7767851	0.01198	10.14	-0.299	38	38	enet	0.028	5.70E-47	-0.514	6.07E-01
SeqId_6416_8	GKN2	2	69172364	69180025	0.20397	rs6726044	-2.356	rs62133344	0.0949	-26.21	-0.332	37	37	enet	0.11	4.00E-181	0.5126	6.08E-01
SeqId_7110_2	DNAJB11	3	186285192	186303589	0.0555	rs6804130	-1.96	rs56227532	4.58E-02	-18.27	-0.413	21	21	enet	0.047	1.00E-77	0.51217	6.09E-01
SeqId_5852_6	S100A12	1	153346187	153348097	0.05795	rs566572	2.103	rs3014874	2.89E-02	-14.53	0.255	28	28	enet	0.036	6.20E-60	-0.5098	0.610193
SeqId_7096_30	RMDN1	8	87480486	87526586	0.21384	rs183776621	-1.987	rs7818213	0.449132	-56.94	-0.106	62	62	enet	0.5	0	0.5087	0.610995
SeqId_17814_8	BPNT1	1	220230824	220263804	0.02611	rs17006829	2.684	rs7530481	2.24E-02	-12.83	-0.432	35	35	enet	0.025	1.20E-41	0.5086	0.61102
SeqId_5108_72	NOTCH3	19	15269849	15311806	0.01756	rs4808310	-2.968	rs4808238	0.011483	9.31	-0.336	38	38	enet	0.013	4.20E-22	-0.50785	0.611559
SeqId_11361_73	TYMP	22	50964181	50968461	0.05174	rs13054036	-2.37	rs131805	0.04514	18.15	0.278	22	22	enet	0.05	1.90E-82	0.5076	0.61176
SeqId_11178_21	SVEP1	9	113127528	113342160	0.20286	rs77229776	3.414	rs78742138	0.05747	20.4	0.812	132	132	enet	0.13	5.00E-221	0.5051	6.13E-01

SeqId_11568_2	FKBP1B	2	24272571	24286551	0.08239	rs13397566	-2.703	rs72781698	0.13115	-30.89	-0.038	36	36	enet	0.17	5.10E-291	-0.501	6.16E-01
SeqId_4145_58	NTF3	12	5541274	5630702	0.01927	rs117715128	-1.947	rs67394720	0.002991	-6.73	-0.146	26	26	enet	0.01	8.50E-18	-0.50091	0.61643
SeqId_10903_50	STX8	17	9153788	9479908	0.04359	rs147422219	1.758	rs35082231	0.038385	16.84	0.771	48	48	enet	0.041	2.60E-68	0.4989	0.61788
SeqId_11109_56	SVEP1	9	113127528	113342160	0.21226	rs79564263	3.084	rs78742138	0.04967	19	0.812	137	137	enet	0.12	1.10E-210	0.4964	6.20E-01
SeqId_12395_86	DARS2	1	173793791	173827946	0.04659	rs9425772	-2.231	rs12564699	3.08E-02	-16.26	-0.444	35	35	enet	0.051	4.60E-85	0.4948	0.620752
SeqId_16890_37	ADAMTSL1	9	17906561	18910948	0.0467	rs58879811	-3.495	rs117766170	0.01414	10.41	-0.414	52	52	enet	0.022	6.30E-37	-0.4937	6.21E-01
SeqId_5272_55	SHC1	1	154934774	154946871	0.00997	rs954785	1.656	rs114272540	2.98E-03	-5.46	0.398	23	23	enet	0.0035	2.70E-07	-0.4914	0.623108
SeqId_14101_2	CNTFR	9	34551430	34590138	0.11745	rs58616892	-1.864	rs73645429	0.0362	-16.15	0.053	58	58	enet	0.063	2.40E-104	0.4894	6.25E-01
SeqId_8956_96	SCARF2	22	20778874	20792113	0.07979	rs62222175	2.57	rs5763025	0.04055	17.25	-1.362	36	36	enet	0.062	8.30E-102	-0.4868	0.62643
SeqId_5036_50	TNFAIP6	2	152214106	152236560	0.14032	rs71415133	-2.821	rs2278089	0.10268	-27.24	0.431	72	72	enet	0.19	0	-0.4824	6.30E-01
SeqId_4188_1	AKR7A2	1	19630459	19638638	0.33359	rs76539818	2.891	rs11800204	4.39E-02	-18.34	-0.099	134	134	enet	0.22	0	-0.4804	0.630921
SeqId_12594_5	GCA	2	163175350	163228105	0.19376	rs1032411	-2.031	rs17783344	0.07967	-24.11	-0.48	10	1	top1	0.08	2.00E-132	0.48	6.31E-01
SeqId_6627_25	PNLIPRP1	10	118349897	118368687	0.07206	rs7912020	-3.14	rs7906926	0.021035	-12.48	-1.419	76	76	enet	0.043	2.00E-71	0.479977	0.6312
SeqId_13384_110	FH	1	241660811	241683099	0.01261	rs7550909	-1.136	rs10926507	1.27E-02	10.1	-0.522	8	8	enet	0.013	8.10E-23	-0.4778	0.63276
SeqId_15447_45	SORD	15	45315345	45369383	0.18178	rs2443978	3.188	rs56060952	0.04549	18.25	-0.219	74	74	enet	0.082	5.60E-136	0.4701	0.6383
SeqId_17685_9	APOA4	11	116691416	116694020	0.05273	rs112357006	1.296	rs12721043	0.031056	-15.07	-0.546	20	20	enet	0.04	3.90E-66	0.46891	6.39E-01
SeqId_18174_79	PDCD6IP	3	33840063	33911199	0.10502	rs11928797	-2.73	rs1141054	1.76E-01	-35.68	0.053	109	109	enet	0.19	0	0.46909	6.39E-01
SeqId_18916_25	ITPA	20	3189514	3204516	0.42794	rs6051329	-2.3	rs67002563	0.251905	-42.75	-0.707	83	83	enet	0.44	0	0.4683	0.63958
SeqId_4925_54	MMP13	11	102813724	102826461	0.00759	rs498614	1.291	rs655316	0.00661	-7.47	-0.468	13	1	top1	0.0066	2.80E-12	0.46826	6.40E-01
SeqId_4126_22	BPI	20	36932552	36965907	0.26323	rs41310809	-2.66	rs6127742	0.093459	-27.19	-0.252	186	186	enet	0.25	0	-0.464	0.64262
SeqId_11104_13	CHI3L1	1	203148059	203155832	0.44056	rs12755165	-1.785	rs946259	3.45E-01	-49.9	-0.258	60	60	enet	0.45	0	0.4637	0.642851
SeqId_2938_55	APOE	19	45409048	45412650	0.00984	rs111794050	2.157	rs5117	0.002029	5.85	0.708	17	17	enet	0.003	2.20E-06	0.46104	0.644773
SeqId_3866_7	SBDS	7	66452667	66460574	0.19429	rs149507545	3.775	rs79344818	0.09158	-26.4	-0.1878	67	67	enet	0.095	2.80E-158	0.4577	0.647151
SeqId_3803_10	CST5	20	23856571	23860385	0.16751	rs6036561	1.13	rs6138152	0.154322	33.58	0.828	28	28	enet	0.2	0	0.4565	0.648
SeqId_12020_39	BPGM	7	134331563	134364568	0.00591	rs12669278	-0.535	rs4732044	0.005718	6.66	-0.455	21	1	top1	0.0057	7.60E-11	-0.455	0.649109
SeqId_16758_96	HDGF	1	156711901	156736717	0.20278	rs12401436	1.62	rs12145743	2.76E-01	-44.65	0.407	26	26	enet	0.28	0	-0.4549	0.649181
SeqId_8464_31	RSPO4	20	939095	982954	0.04057	rs73892620	1.49	rs75904281	0.009482	-9.82	0.021	43	43	enet	0.035	8.50E-58	0.4549	0.64921
SeqId_6431_68	PCYOX1	2	70484518	70508317	0.36554	rs72839899	2.316	rs2706762	0.22805	-40.61	0.341	48	48	enet	0.24	0	-0.4531	6.50E-01
SeqId_13544_9	HMHA1	19	1065922	1086627	0.05755	rs72981685	-1.322	rs2240051	0.051829	20.31	0.713	24	24	enet	0.072	6.80E-120	0.45314	0.65045
SeqId_5688_65	CBLN4	20	54572413	54580575	0.08579	rs6064313	2.63	rs4811612	0.025308	13.56	-0.501	120	120	enet	0.085	4.90E-142	-0.4519	0.65131
SeqId_5087_5	IL22RA2	6	137464957	137494785	0.04763	rs74654606	-3.05	rs6917836	0.00776	-8.28	-0.618	46	46	enet	0.032	2.60E-53	-0.45	6.52E-01
SeqId_13692_154	WISP1	8	134203282	134243933	0.08165	rs118029191	2.112	rs60282000	0.037253	-16.59	-0.3469	56	56	enet	0.079	1.20E-130	-0.446	0.655564
SeqId_18289_16	CCL15	17	34323476	34328592	0.19092	rs2075746	2.476	rs854624	0.286256	45.51	-0.76	32	32	enet	0.3	0	-0.4444	0.65674
SeqId_16605_2	C1QTNF9	13	24881304	24896673	0.06016	rs9578655	1.9	rs56069023	0.04961	-19	0.438	48	48	enet	0.063	1.70E-104	-0.441	0.6589
SeqId_3044_3	CCL18	17	34391632	34399392	0.23388	rs112689088	2.445	rs2015086	0.173953	35.46	-0.059	63	63	enet	0.22	0	-0.4393	0.66043
SeqId_2888_49	C7	5	40909599	40984745	0.6453	rs184457268	1.856	rs74480769	1.48E-01	-32.73	0.947	86	86	enet	0.31	0	-0.4384	0.6611
SeqId_5363_51	SEMA3E	7	82992554	83278455	0.29888	rs7458708	-1.885	rs3801499	0.246002	-42.17	-0.339	72	72	enet	0.39	0	0.4376	0.661644
SeqId_10396_6	MCL1	1	150533371	150552214	0.09241	rs140520803	-1.698	rs190596489	4.21E-02	-17.73	0.249	37	37	enet	0.043	2.80E-71	-0.4358	0.662951
SeqId_3376_49	IL17RD	3	57124010	57204334	0.12749	rs139950577	1.36	rs6776722	1.55E-01	-34.67	-0.15	34	34	enet	0.2	0	-0.43641	6.63E-01
SeqId_6609_22	CNP	17	40118781	40129758	0.03523	rs3760522	1.835	rs4432296	0.030978	15.14	-0.946	42	42	enet	0.037	2.80E-61	-0.4351	0.6635
SeqId_11333_82	ARHGAP25	2	68906733	69053965	0.12147	rs147495962	-2.247	rs13413887	0.03282	15.59	-0.178	57	57	enet	0.077	3.40E-127	-0.435	6.64E-01
SeqId_12486_8	GLRX2	1	193059996	193075244	0.11091	rs10801173	-2.104	rs143131184	3.61E-02	16.2	0.739	28	28	enet	0.04	1.50E-66	0.4342	0.664176
SeqId_6364_7	TAPBPL	12	6560856	6575683	0.29285	rs2041388	0.613	rs2534709	0.485497	59.27	0.116	29	29	enet	0.54	0	0.43283	0.66513
SeqId_16300_4	TREM2	6	41126244	41130924	0.10774	rs60991481	2.12	rs143332484	0.04849	-18.74	-0.4755	39	39	enet	0.053	2.30E-87	0.432	6.66E-01
SeqId_4240_31	PKM2	15	72491370	72524163	0.00429	rs117900766	1.808	rs28379368	0.00208	-5.35	-0.233	7	7	enet	0.0023	2.50E-05	-0.431	0.6665
SeqId_4971_1	CTSZ	20	57560741	57583293	0.02545	rs79843552	-2.31	rs75940688	0.019924	-12.14	-0.39	27	27	enet	0.031	4.80E-52	0.4295	0.66754
SeqId_17377_1	AKR1C3	10	5077546	5149878	0.01239	rs4491123	1.57	rs4880709	0.007828	-7.76	0.014	15	15	enet	0.0082	8.90E-15	-0.429205	0.6678
SeqId_15472_16	LRP11	6	150139893	150185495	0.24207	rs111576955	-2.36	rs3805753	0.38271	52.63	0.459	146	146	enet	0.42	0	0.429	6.68E-01
SeqId_2692_74	PLA2G2A	1	20301924	20306909	0.27634	rs77277881	2.223	rs2307246	2.23E-01	40.15	-0.204	54	54	enet	0.25	0	-0.427	0.669388
SeqId_16614_27	RSPO1	1	38076951	38100564	0.07475	rs115228768	2.03	rs36043533	5.58E-02	20.11	0.296	26	26	enet	0.07	1.10E-115	0.4222	0.672886
SeqId_8403_18	FASN	17	80036214	80056170	0.0082	rs147067974	-2.527	rs62078746	0.013469	-10.88	-0.578	25	25	enet	0.014	2.50E-24	0.4218	0.67315
SeqId_15614_168	LILRA2	19	55084387	55101752	0.12131	rs1626971	2.849	rs2151868	0.064809	-21.71	-0.0148	59	59	enet	0.093	1.10E-155	0.42012	0.674397
SeqId_4192_10	AKR1A1	1	46016215	46035721	0.3428	rs148417473	-2.712	rs2229540	2.31E-01	-40.86	-0.653	51	51	enet	0.27	0	0.4182	0.675818

SeqId_4563_61	PLCG1	20	39765600	39825441	0.01564	rs34423638	2.35	rs753381	0.01116	9.23	-0.418	22	1	top1	0.011	1.40E-19	-0.418	0.67595
SeqId_13102_1	FAM3D	3	58619673	58652561	0.05865	rs56391639	-1.87	rs7433100	3.31E-02	15.87	-1.271	26	26	enet	0.042	1.00E-68	-0.41816	6.76E-01
SeqId_3488_64	CAT	11	34460481	34493607	0.09623	rs11032656	1.956	rs4756148	0.042283	-17.58	-0.669	63	63	enet	0.084	4.80E-139	-0.41472	6.78E-01
SeqId_8358_30	PRDX3	10	120927232	120938324	0.0056	rs10886323	-1.95	rs1810159	0.005467	-6.56	-0.415	10	1	top1	0.0055	1.90E-10	0.415	0.6781
SeqId_2844_53	TIE1	1	43766653	43788779	0.02963	rs12145511	2.02	rs1556580	2.87E-02	15.13	-0.67	27	27	enet	0.031	5.10E-52	-0.4148	0.678315
SeqId_7948_129	GLTPD2	17	4692259	4693884	0.09076	rs543281879	-1.878	rs34460487	0.036337	16.33	0.059	45	45	enet	0.061	5.80E-101	-0.4147	0.67839
SeqId_17332_3	ADPRHL2	1	36554493	36559533	0.01837	rs12567252	2.265	rs58350664	1.72E-03	5.19	-0.238	59	59	enet	0.0028	3.50E-06	-0.4116	0.680614
SeqId_15516_12	SAA4	11	18252902	18258349	0.39314	rs144559255	-3.107	rs4757628	0.164993	-35.62	1.06	138	138	enet	0.36	0	-0.41075	6.81E-01
SeqId_5460_60	DDX19B	16	70323566	70369208	0.03402	rs34608864	-2.28	rs138325325	0.01178	-9.86	-0.925	18	18	enet	0.012	1.00E-21	0.4109	6.81E-01
SeqId_4234_8	IL1RL1	2	102927962	102968497	0.29196	rs3917329	-2.456	rs950880	0.18228	-38.27	-0.303	54	54	enet	0.3	0	0.4117	6.81E-01
SeqId_6556_5	ENPP5	6	46126922	46138717	0.33537	rs7758684	2.25	rs1047153	0.29297	-46	-0.35	74	74	enet	0.46	0	0.411	6.81E-01
SeqId_16792_4	SIGLEC5	19	52115180	52148798	0.58557	rs55912643	-2.427	rs872629	0.208728	-38.85	-0.776	270	270	enet	0.4	0	0.41094	0.681114
SeqId_8479_4	MMP10	11	102641233	102651359	0.26282	rs17293152	-2.506	rs17860955	0.035215	-16.9	0.105	168	168	enet	0.13	2.00E-212	0.41026	6.82E-01
SeqId_8778_3	NOG	17	54671060	54672972	0.02426	rs190238343	-2.592	rs227715	0.007837	-8.78	-0.2825	76	76	enet	0.016	4.90E-27	0.4086	0.68286
SeqId_4911_49	GSTP1	11	67351213	67354127	0.24537	rs2514022	1.723	rs1695	0.153121	-33.25	-0.344	48	48	enet	0.2	0	-0.40844	6.83E-01
SeqId_12714_38	AP1G2	14	24028774	24037279	0.07426	rs74840007	-3.024	rs12886523	0.031243	-15.15	0.421	94	94	enet	0.039	1.60E-64	-0.4089	6.83E-01
SeqId_5837_49	LIFR	5	38474770	38608456	0.07727	rs7733185	3.601	rs3729741	2.76E-02	14.41	-0.437	58	58	enet	0.065	6.50E-108	-0.40384	0.68633
SeqId_16919_1	DBI	2	120124500	120130126	0.10727	rs55636627	1.685	rs6714264	0.08616	-25.15	0.101	94	94	enet	0.13	2.90E-217	0.4035	6.87E-01
SeqId_15635_4	SMOC2	6	168841831	169073984	0.1638	rs182866664	-2.63	rs56296467	0.08756	-25.22	0.52	122	122	enet	0.16	1.00E-277	-0.403	6.87E-01
SeqId_9416_77	CPM	12	69235977	69365350	0.04146	rs12303166	1.976	rs8181716	0.013814	-10.21	0.808	25	25	enet	0.027	2.20E-45	0.40207	0.68763
SeqId_18891_98	GBP2	1	89571815	89616139	0.02062	rs147641749	-2.605	rs10922556	7.59E-03	7.97	-1.204	25	25	enet	0.011	3.00E-19	-0.4021	0.687641
SeqId_9482_110	NUDT9	4	88343725	88380607	0.06864	rs35679067	1.366	rs28805573	0.047193	-19.04	0.02	35	35	enet	0.067	1.40E-110	0.4	6.89E-01
SeqId_18310_26	SEPW1	19	48281842	48287943	0.05491	rs191007172	-2.135	rs11671137	0.04195	18.53	-0.0353	17	17	enet	0.064	2.60E-106	0.39957	0.689471
SeqId_3040_59	CCL3	17	34415602	34417515	0.134	rs139881749	-2.289	rs2015086	0.088204	25.26	-0.059	72	72	enet	0.11	8.90E-179	-0.3991	0.68984
SeqId_4124_24	HSPA1A	6	31783320	31785723	0.01794	rs9265870	-3.46	rs74434374	0.0058	-7.14	-0.072	56	56	enet	0.009	4.10E-16	-0.395	6.93E-01
SeqId_5628_21	SEMA3G	3	52467051	52479119	0.05732	rs111383873	2.72	rs2016575	3.58E-02	-16.16	0.409	53	53	enet	0.05	4.70E-82	-0.38968	6.97E-01
SeqId_2631_50	IL10RB	21	34638672	34682492	0.04928	rs11702633	1.08	rs2515717	0.02472	13.95	0.572	21	21	enet	0.045	2.50E-74	0.3886	0.698
SeqId_3481_87	XPNPEP1	10	111624524	111683311	0.0337	rs67574388	-1.82	rs3862006	0.033955	16.57	-0.651	31	31	enet	0.044	3.40E-73	-0.387914	0.6981
SeqId_3206_4	LYVE1	11	10578513	10633236	0.05668	rs16907959	2.423	rs114758648	0.018757	11.85	-0.477	50	50	enet	0.039	8.80E-64	-0.38451	7.01E-01
SeqId_18241_18	CPOX	3	98298290	98312492	0.06686	rs191648239	-2.68	rs1675513	1.85E-02	-11.67	-0.941	44	44	enet	0.038	2.70E-62	-0.38341	7.01E-01
SeqId_9005_16	PLXNA1	3	126701536	126756232	0.06548	rs28488422	2.29	rs9822602	8.07E-02	-24.2	-0.126	55	55	enet	0.092	2.00E-153	0.38438	7.01E-01
SeqId_5459_33	CST1	20	23728199	23731905	0.124	rs150615439	2.25	rs4260306	0.102783	-27.27	-0.75	31	31	enet	0.12	2.10E-197	0.3837	0.70118
SeqId_3593_72	CASP3	4	185548850	185570663	0.08534	rs13435690	-2.075	rs72689255	0.11319	-28.79	1.056	49	49	enet	0.15	1.30E-262	-0.3817	7.03E-01
SeqId_7757_5	HLA-DQA2	6	32709168	32714975	0.17668	rs28746898	-3.09	rs9272014	0.14885	-32.9	-0.417	83	83	enet	0.24	0	0.381	7.03E-01
SeqId_13416_8	TMEM132D	12	129556270	130388570	0.04722	rs74322448	4.442	rs9804953	0.009253	-9.14	0.458	97	97	enet	0.031	1.30E-51	-0.38016	0.70383
SeqId_4159_130	CFH	1	196621173	196716634	0.13146	rs72732292	-3.585	rs1048663	9.89E-02	-26.86	0.463	62	62	enet	0.14	1.10E-230	0.3783	0.705232
SeqId_8243_55	SPINK1	5	147204135	147211234	0.02574	rs72827192	2.714	rs6580502	1.23E-02	-9.69	-0.209	39	39	enet	0.016	4.80E-28	-0.37725	0.70599
SeqId_6408_2	INHBC	12	57828567	57845845	0.00893	rs3024981	-0.857	rs6581138	0.004272	5.69	-0.377	17	1	top1	0.0043	1.60E-08	-0.377	0.70617
SeqId_10558_26	PCDH9	13	66876966	67804577	0.04198	rs117905204	-3.49	rs4884685	0.00483	-7.32	-0.942	58	58	enet	0.019	2.50E-32	-0.377	0.7062
SeqId_7161_25	H6PD	1	9294833	9331396	0.18639	rs193065720	2.565	rs34603401	1.33E-01	30.96	-0.215	59	59	enet	0.22	0	-0.3766	0.706434
SeqId_17490_4	SH3BGRL3	1	26606577	26608013	0.09094	rs4970503	2.528	rs4659423	1.81E-01	-36.3	1.473	61	61	enet	0.21	0	-0.3763	0.706708
SeqId_2754_50	C3	19	6677715	6730573	0.0069	rs34090333	-1.518	rs11569479	0.005016	-6.79	0.135	7	7	enet	0.0054	2.40E-10	-0.37574	0.707109
SeqId_11428_31	PDLIM1	10	96997329	97050769	0.13847	rs7079728	-2.25	rs1328599	0.030062	-14.79	-1.106	52	52	enet	0.044	5.30E-72	-0.374427	0.7081
SeqId_10781_19	CLEC4G	19	7793844	7798792	0.05853	rs11260029	-3.486	rs1010046	0.0395	17.1	-1.174	62	62	enet	0.04	3.50E-66	-0.37374	0.708601
SeqId_3293_2	CD5L	1	157800701	157868046	0.12573	rs9970909	-2.718	rs2765501	6.80E-02	22.19	0.448	42	42	enet	0.11	1.10E-186	-0.3715	0.710233
SeqId_11192_168	TINAGL1	1	32042086	32053287	0.01705	rs116787462	-2.162	rs9425884	2.29E-03	-5.69	0.644	42	42	enet	0.0066	2.70E-12	-0.3712	0.710525
SeqId_14114_18	PIANP	12	6802957	6809981	0.05121	rs186487833	-1.91	rs7959658	0.018056	11.61	-1.022	39	39	enet	0.026	3.70E-43	-0.3705	0.71101
SeqId_18220_141	SRA1	5	139916925	139937262	0.13304	rs73267612	-3.094	rs801460	1.06E-01	27.83	0.382	78	78	enet	0.13	1.70E-218	0.36945	0.71179
SeqId_17755_5	UGDH	4	39500375	39529931	0.10227	rs115993543	-1.187	rs1138891	0.095598	26.39	0.384	34	34	enet	0.12	1.40E-199	-0.3686	7.12E-01
SeqId_15308_108	VWC2	7	49813234	49961546	0.34007	rs2366025	2.414	rs73347621	0.100015	-27.08	-0.156	119	119	enet	0.21	0	-0.3683	0.712642
SeqId_16828_8	COL6A1	21	47401684	47424964	0.42625	rs55896864	2.02	rs1053312	0.50453	-60.33	0.349	53	53	enet	0.52	0	-0.3683	0.713
SeqId_8953_47	HDGF	1	156711901	156736717	0.13553	rs74119208	2.082	rs4399146	1.78E-01	-36.01	0.383	27	27	enet	0.18	4.6e-311	-0.3669	0.713663
SeqId_4187_49	PGD	1	10458649	10480568	0.02891	rs10864459	2.448	rs111782199	3.37E-02	-15.77	-0.366	18	1	top1	0.034	5.40E-56	0.366	0.714365

SeqId_3054_3	HP	16	72088404	72094954	0.41047	rs117784872	2.32	rs77303550	0.1425	32.11	-1.725	89	89	enet	0.37	0	-0.3648	7.15E-01
SeqId_18280_29	DTD2	14	31915242	31926712	0.14497	rs79740809	-2.111	rs17097898	0.046087	-18.44	-0.802	67	67	enet	0.084	8.30E-140	0.3632	7.16E-01
SeqId_4127_75	C6	5	41142218	41261540	0.29663	rs114987985	2.023	rs191791682	5.52E-02	-20	-0.288	130	130	enet	0.12	1.50E-201	-0.36348	0.71625
SeqId_19213_1	SPINK4	9	33218363	33248565	0.10448	rs139127480	-1.845	rs1630171	0.16432	34.46	-0.7	57	57	enet	0.18	2.30E-306	-0.3605	7.18E-01
SeqId_12436_84	GSTO1	10	105995114	106027217	0.27098	rs117527269	-2.67	rs641071	0.404319	-54.02	-0.427	33	33	enet	0.44	0	0.359903	0.7189
SeqId_6414_8	OAF	11	120081740	120101043	0.20917	rs141469382	-3.851	rs2508490	0.159983	-34.02	-0.916	79	79	enet	0.27	0	0.35875	7.20E-01
SeqId_6385_63	VWA1	1	1370241	1378262	0.06831	rs111615792	-1.487	rs116279124	6.68E-02	-22.28	0.564	24	24	enet	0.072	8.50E-120	-0.3571	0.721018
SeqId_2683_1	C3	19	6677715	6730573	0.00759	rs1975159	-1.932	rs11569479	0.003991	-6.51	0.135	20	20	enet	0.0042	2.60E-08	0.35651	0.72146
SeqId_9244_27	PPT1	1	40538382	40562932	0.14171	rs41268047	-2.757	rs7533094	1.14E-01	-28.64	-0.217	52	52	enet	0.15	3.50E-259	0.3545	0.722971
SeqId_9231_23	IMPAD1	8	57870490	57906426	0.19565	rs10093153	1.781	rs112433249	0.105474	-27.67	-0.742	72	72	enet	0.14	1.70E-233	0.3535	0.723697
SeqId_10346_5	STAT3	17	40465342	40540558	0.05612	rs12949918	-0.492	rs4796791	0.082651	24.5	-0.351	29	1	top1	0.083	1.60E-137	-0.351	0.72559
SeqId_6392_7	WISP2	20	43343485	43357150	0.15766	rs6073532	-2.41	rs754077	0.057981	20.66	0.321	62	62	enet	0.12	1.60E-209	-0.3493	0.72688
SeqId_3213_65	NID1	1	236139130	236228409	0.06636	rs61833977	2.448	rs2734807	2.45E-02	13.42	-0.97	70	70	enet	0.028	8.20E-47	-0.3493	0.726887
SeqId_16015_19	GPT	8	145728356	145732557	0.01142	rs78425244	-2.148	rs4244610	0.007822	-8.28	0.781	22	22	enet	0.0084	3.40E-15	-0.347	0.728578
SeqId_12573_80	TRIM3	11	6469843	6495689	0.0108	rs187933066	-2.043	rs2344827	0.005393	-6.69	-0.346	25	1	top1	0.0054	2.60E-10	0.346	7.29E-01
SeqId_15316_262	TXNL4B	16	72078188	72128330	0.12961	rs72793223	-2.26	rs116891509	0.04058	17.51	-0.899	40	40	enet	0.053	3.20E-88	-0.3464	7.29E-01
SeqId_17843_30	PPCS	1	42921788	42939056	0.03906	rs1098789	-2.12	rs4660635	9.72E-02	-26.53	-0.052	12	12	enet	0.098	2.30E-164	0.3455	0.729751
SeqId_9772_153	NLGN2	17	7308193	7323179	0.05043	rs4791806	-3.46	rs150452493	0.022901	-13.37	-0.61	16	16	enet	0.029	1.50E-48	0.3429	0.7317
SeqId_9713_67	PDGFRL	8	17433942	17501580	0.09564	rs77363821	2.264	rs78243536	0.044175	-18.41	-0.23	69	69	enet	0.074	4.30E-123	-0.3419	0.732454
SeqId_2982_82	LGALS4	19	39292311	39303585	0.0655	rs117087498	-1.913	rs9304578	0.037901	-16.58	1.051	44	44	enet	0.064	1.60E-105	-0.33848	0.734998
SeqId_4440_15	FCRL3	1	157644111	157670559	0.13787	rs79756905	2.038	rs3761959	2.49E-01	42.51	-0.276	11	11	enet	0.26	0	-0.338	0.735351
SeqId_3216_2	PIGR	1	207101867	207119811	0.02196	rs2808467	1.801	rs6540730	1.89E-02	-12.26	-1.162	39	39	enet	0.023	2.60E-39	0.3378	0.735531
SeqId_3195_50	GNLY	2	85912298	85925975	0.27148	rs13419986	-2.392	rs12151742	0.20782	38.73	-0.618	72	72	enet	0.24	0	-0.3367	7.36E-01
SeqId_14076_74	CST4	20	23666277	23669675	0.05458	rs4290725	-1.59	rs7263473	0.037751	-16.7	-0.319	23	23	enet	0.053	2.60E-88	-0.3363	0.73668
SeqId_10630_5	HTATIP2	11	20385231	20405328	0.04717	rs61100079	-1.114	rs10437608	0.043058	17.88	0.336	10	1	top1	0.043	3.30E-71	0.336	7.37E-01
SeqId_9931_20	KRT1	12	53068520	53074191	0.01448	rs650694	1.806	rs117254202	0.005531	6.62	-0.335	43	1	top1	0.0055	1.50E-10	-0.335	0.73763
SeqId_9359_9	DLK2	6	43418090	43424370	0.11208	rs2277126	2.23	rs2125739	0.06711	-22.09	0.352	56	56	enet	0.081	2.20E-134	-0.334	7.38E-01
SeqId_3323_37	LRP8	1	53708036	53794141	0.06527	rs111536237	-1.939	rs7542607	3.00E-02	15.09	0.197	59	59	enet	0.05	1.10E-82	0.331	0.740646
SeqId_6077_63	CECR1	22	17659680	17739125	0.66613	rs174345	2.4	rs2231495	0.31846	-47.95	-0.377	55	55	enet	0.36	0	0.3295	0.74178
SeqId_7957_2	SCG3	15	51973689	52013223	0.20943	rs150559389	2.784	rs2607116	0.23798	41.46	0.365	89	89	enet	0.28	0	0.3283	0.7427
SeqId_17682_1	CD46	1	207925399	207968858	0.10368	rs151105668	-1.752	rs2488252	1.72E-01	-35.29	-0.388	48	48	enet	0.21	0	0.3281	0.742862
SeqId_7905_30	HP	16	72088404	72094954	0.29605	rs217181	-1.73	rs11648003	0.08588	-26.07	0.056	49	49	enet	0.25	0	-0.3269	7.44E-01
SeqId_8099_42	SPON2	4	1160720	1202750	0.12827	rs112069922	-1.782	rs11247975	0.153872	-33.39	0.463	53	53	enet	0.19	0	-0.3268	7.44E-01
SeqId_6247_9	SIRPB1	20	1542031	1600707	0.4662	rs181600116	3.53	rs12480515	0.269027	44.06	0.986	192	192	enet	0.52	0	0.3239	0.74599
SeqId_9316_67	WFDC1	16	84328452	84363450	0.28646	rs9928060	-2.46	rs400345	0.21547	-39.44	0.15	135	135	enet	0.26	0	0.3223	7.47E-01
SeqId_7871_16	TMEM132A	11	60691932	60704631	0.28003	rs187722597	3.389	rs555835	0.23082	40.81	-1.314	53	53	enet	0.36	0	-0.32169	7.48E-01
SeqId_12677_164	FLII	17	18148129	18162052	0.05435	rs143583729	0.876	rs145840264	0.018475	11.67	0.321	3	1	top1	0.018	2.60E-31	0.321	0.74821
SeqId_3329_14	PGLYRP1	19	46522411	46526311	0.02131	rs45587933	0.839	rs2072563	0.019025	-11.89	0.321	11	1	top1	0.019	3.40E-32	-0.321	0.74821
SeqId_5736_1	TREML2	6	41157487	41168887	0.18912	rs9381022	-3.03	rs4714431	0.11822	29.23	0.948	64	64	enet	0.18	1.3e-314	-0.319	7.49E-01
SeqId_14156_33	YWHAB	20	43514343	43537173	0.19778	rs76480930	2.59	rs6031847	0.322603	-48.35	0.358	46	46	enet	0.33	0	-0.3194	0.74944
SeqId_13983_27	CRYZ	1	75171166	75199092	0.18276	rs138967176	2.105	rs3819946	2.76E-01	44.63	0.289	52	52	enet	0.32	0	-0.3184	0.75016
SeqId_19334_62	TXNDC12	1	52485803	52520863	0.10222	rs548304890	-1.57	rs6686632	5.17E-02	-19.94	0.068	47	47	enet	0.058	1.40E-96	-0.3184	0.750175
SeqId_5456_59	CNDP1	18	72201735	72254448	0.11812	rs11660249	-3.23	rs17817077	0.02834	14.38	1.058	57	57	enet	0.056	4.30E-92	0.317	0.7515
SeqId_5728_60	FCRL1	1	157764193	157789910	0.08298	rs12118214	-2.179	rs4971154	8.29E-02	-24.53	0.374	54	54	enet	0.09	8.60E-150	-0.3151	0.752675
SeqId_9995_6	DUT	15	48623208	48635570	0.00471	rs12594187	0.478	rs8039702	0.00502	-6.32	0.314	9	1	top1	0.005	1.00E-09	-0.314	0.7535
SeqId_8894_80	HNRNPAB	5	177631534	177638164	0.01276	rs72648830	0.88	rs72648830	4.40E-03	6.76	0.88	7	7	enet	0.01	1.00E-17	0.31347	0.75393
SeqId_5631_83	MLN	6	33762450	33771780	0.0743	rs499384	2.69	rs9394169	0.06443	-23.05	0.299	51	51	enet	0.1	8.70E-168	0.312	7.55E-01
SeqId_13719_19	PAK4	19	39616410	39673456	0.01676	rs11668803	-1.771	rs11667387	0.002555	-6.49	-0.671	17	17	enet	0.0057	8.10E-11	-0.30971	0.756782
SeqId_7852_9	MTIF3	13	28009780	28024728	0.01487	rs9512819	2.22	rs1218802	0.00628	-6.95	-0.306	25	1	top1	0.0063	9.60E-12	0.306	0.7596
SeqId_3148_49	CXCL2	4	74962757	74964913	0.00617	rs3806792	-0.733	rs1893319	0.003955	6.54	0.441	6	6	enet	0.004	4.10E-08	0.304	7.61E-01
SeqId_9256_78	NPTX1	17	78440634	78451643	0.07675	rs77877786	-2.925	rs62068268	0.06707	22.36	0.308	115	115	enet	0.092	3.20E-154	-0.3036	0.76143
SeqId_5092_51	JAG1	20	10618332	10654647	0.01592	rs6033029	-2.97	rs78896696	0.003641	5.97	-0.242	39	39	enet	0.0077	4.20E-14	-0.3002	0.76399
SeqId_7922_5	ADM	11	10326620	10328944	0.00613	rs2403291	-1.401	rs2923098	0.000331	5.18	1.061	15	15	enet	0.0017	0.00024	0.29968	7.64E-01

SeqId_14091_42	CBR3	21	37507377	37518860	0.23081	rs113382956	-1.79	rs60376898	0.32543	-48.63	0.443	74	74	enet	0.35	0	-0.3002	0.764
SeqId_5644_60	RNASE4	14	21152719	21169375	0.23615	rs77871000	-2.226	rs1888560	0.112673	-28.53	0.0418	95	95	enet	0.19	0	-0.299	7.65E-01
SeqId_3029_52	CD209	19	7804879	7812450	0.11629	rs594793	-3.175	rs2335525	0.058734	-20.9	-0.366	113	113	enet	0.1	7.30E-170	0.29644	0.766895
SeqId_9793_145	IGDCC4	15	65673822	65715285	0.24044	rs72740703	-3.107	rs8036706	0.02824	15.37	0.983	93	93	enet	0.11	8.20E-185	-0.2932	0.7693
SeqId_13615_60	NCK2	2	106361369	106510730	0.04001	rs188514627	2.151	rs933793	0.008	-10.19	-0.73	62	62	enet	0.033	5.80E-55	-0.2925	7.70E-01
SeqId_15596_7	HEXIM1	17	43225842	43229466	0.02004	rs2304987	1.765	rs12051846	0.015567	11.01	0.739	29	29	enet	0.017	3.20E-29	0.2886	0.77285
SeqId_8469_41	IGFBP2	2	217497551	217529159	0.01589	rs7573644	-2.706	rs4674091	0.00438	-6.24	-0.37	34	34	enet	0.011	3.80E-19	-0.2865	7.74E-01
SeqId_9545_156	GZMK	5	54320084	54330398	0.03102	rs77783349	-2.103	rs2407827	1.22E-02	10.03	0.378	21	21	enet	0.017	1.20E-29	-0.28636	0.7746
SeqId_10847_1	SIGLEC15	18	43405640	43424059	0.01007	rs12608098	-1	rs515373	0.00838	8.07	0.755	16	16	enet	0.009	3.80E-16	0.285	0.7754
SeqId_2515_14	GFRA2	8	21547910	21669869	0.15995	rs1479057	-2.081	rs15881	0.062737	21.31	-1.096	80	80	enet	0.16	2.90E-271	-0.2846	0.775935
SeqId_7198_197	FAM20B	1	178994939	179045702	0.04117	rs149522945	-1.924	rs12568291	1.17E-02	9.61	0.016	59	59	enet	0.018	1.10E-30	0.2838	0.776568
SeqId_12459_13	PLEKHA1	10	124134212	124202118	0.02026	rs2672592	2.29	rs10788274	0.025208	-13.69	-0.623	18	18	enet	0.028	4.60E-46	0.278092	0.7809
SeqId_2579_17	MMP9	20	44637547	44645200	0.04624	rs73120401	-1.59	rs8113877	0.035061	-16.28	-0.538	20	20	enet	0.042	1.40E-69	0.2753	0.78307
SeqId_5134_52	HAVCR2	5	156512843	156569880	0.34797	rs116708816	3.06	rs1432872	2.74E-01	-44.54	-0.559	252	252	enet	0.29	0	0.27426	0.78388
SeqId_18832_65	SAA2	11	18260770	18270215	0.11297	rs2158395	1.763	rs11024589	0.063297	-21.41	-0.099	18	18	enet	0.085	9.80E-141	-0.27389	7.84E-01
SeqId_5337_64	CD86	3	121774209	121839986	0.01529	rs17742642	2.61	rs77832668	-3.80E-05	4.21	-0.604	41	41	enet	0.002	1.00E-04	-0.27466	7.84E-01
SeqId_12727_7	PTGFRN	1	117452538	117532975	0.38031	rs34403643	-1.914	rs4233450	2.99E-01	46.48	0.299	71	71	enet	0.39	0	0.274	0.784077
SeqId_10714_7	ACE	17	61554422	61575741	0.30581	rs76793890	1.932	rs4353	0.281192	-45.05	-0.442	41	41	enet	0.32	0	0.2734	0.78454
SeqId_3280_49	ACAN	15	89346667	89418584	0.03556	rs12913320	1.795	rs34949187	0.00803	-10.06	-0.217	59	59	enet	0.025	5.40E-42	0.2731	0.7848
SeqId_5900_11	HINT1	5	130491076	130560161	0.01974	rs113642581	-0.873	rs12332117	3.63E-02	16.31	-0.252	29	29	enet	0.036	2.20E-60	-0.27278	0.78503
SeqId_2474_54	APCS	1	159557616	159558655	0.06347	rs114735981	-2.463	rs77672383	3.75E-02	-16.88	1.514	57	57	enet	0.065	6.70E-107	-0.2725	0.785246
SeqId_3077_66	F10	13	113777113	113803843	0.12796	rs9577464	2.57	rs3211757	0.03647	16.42	-0.139	58	58	enet	0.069	4.90E-114	0.272	0.786
SeqId_12956_40	KIAA1279	10	70748559	70803300	0.00946	rs75583483	-10.59	rs17471869	0.000542	5.68	0.877	16	16	enet	0.0023	3.00E-05	0.27128	0.7862
SeqId_3302_58	CST7	20	24929905	24940564	0.27104	rs11907788	2.63	rs4462848	0.109636	28.33	0.50508	176	176	enet	0.18	5.1e-312	-0.2709	0.78647
SeqId_16818_200	CDCP1	3	45123769	45187914	0.12491	rs138293488	2.07	rs62244469	3.89E-02	-17.06	-0.56	73	73	enet	0.09	1.20E-149	-0.26986	7.87E-01
SeqId_3296_92	CNTN2	1	205012065	205047417	0.34906	rs58145004	1.74	rs3753847	1.88E-01	-36.86	0.617	115	115	enet	0.37	0	-0.27	0.787181
SeqId_19273_3	GSR	8	30535583	30585363	0.06774	rs17651216	3.24	rs2551710	0.012201	-9.99	-0.3032	43	43	enet	0.028	1.70E-46	-0.2675	0.789081
SeqId_6060_2	PIP	7	142829170	142836832	0.23817	rs75940454	-3.382	rs4726600	0.03131	16.09	0.0126	203	203	enet	0.089	3.10E-148	0.2673	0.789247
SeqId_9348_1	C1RL	12	7242183	7261834	0.28526	rs150452082	-1.908	rs146111161	0.044313	-3.8	0.126	18	18	enet	0.17	2.90E-285	-0.26697	0.78949
SeqId_15394_79	UNC5B	10	72972327	73062621	0.13879	rs1417913	2.41	rs7907590	0.011618	-11.11	0.83	118	118	enet	0.081	2.90E-135	-0.265863	0.7903
SeqId_4249_64	NME2	17	49242796	49249105	0.247	rs190391283	-1.616	rs115848216	0.147635	-32.74	0.232	61	61	enet	0.23	0	0.2655	0.7906
SeqId_4811_33	ITIH4	3	52846978	52864704	0.03165	rs142796438	3.31	rs3617	1.07E-02	9.69	-0.906	37	37	enet	0.017	6.30E-29	-0.26442	7.91E-01
SeqId_2816_50	BCAM	19	45312328	45324678	0.10822	rs10412857	-3.853	rs563366239	0.038406	16.73	-0.521	87	87	enet	0.053	6.80E-87	0.2643	0.791551
SeqId_11145_72	KIAA1549L	11	33397654	33695648	0.19393	rs3847595	2.488	rs2753408	0.147915	-32.68	-0.265	30	30	enet	0.18	9.6e-319	0.26436	7.92E-01
SeqId_3403_1	TPSB2	16	1277272	1280185	0.42011	rs188109028	-2.85	rs3874639	0.31838	-47.93	-0.538	153	153	enet	0.39	0	0.2615	7.94E-01
SeqId_15462_28	CD8A	2	87011733	87035519	0.11561	rs77940787	1.772	rs3020726	0.07079	22.64	0.089	53	53	enet	0.087	1.50E-145	0.2604	7.95E-01
SeqId_4276_10	PEBP1	12	118573929	118583389	0.14872	rs4766885	2.393	rs1050625	0.086526	-25	0.951	54	54	enet	0.13	6.40E-213	0.25766	0.79667
SeqId_5682_13	VASN	16	4421849	4433539	0.02132	rs6500586	1.66	rs757593	0.00655	7.12	0.237	20	20	enet	0.01	4.40E-18	0.2555	7.98E-01
SeqId_9077_10	MAN1A2	1	117910071	118071494	0.11284	rs6666558	-1.152	rs4659047	6.31E-02	-21.84	-0.471	60	60	enet	0.14	3.10E-237	0.2559	0.798023
SeqId_2743_5	SHH	7	155592674	155605157	0.02419	rs116893723	-2.719	rs872723	0.009403	-8.47	-0.5787	41	41	enet	0.012	3.90E-21	0.2553	0.798463
SeqId_19622_7	INHBA	7	41706766	41745432	0.00859	rs273118	2.458	rs1122291	0.004008	-5.79	-0.253	18	1	top1	0.004	4.40E-08	0.253	0.800268
SeqId_8043_153	COMP	19	18893583	18902114	0.06506	rs11672508	3.003	rs12974746	0.024051	-13.25	-0.276	78	78	enet	0.034	8.20E-56	0.25276	0.800456
SeqId_15466_30	COL9A1	6	70925743	71012787	0.02442	rs76893750	2.28	rs13199337	0.00261	6.54	-0.7718	51	51	enet	0.014	2.40E-24	-0.253	8.01E-01
SeqId_15587_20	FOLR2	11	71927645	71932994	0.02521	rs365274	0.85	rs74603010	0.00685	7.41	0.156	28	28	enet	0.0086	2.00E-15	0.25029	8.02E-01
SeqId_3046_31	RETN	19	7733935	7735341	0.15585	rs10407033	2.753	rs34124816	0.030508	-14.93	0.735	112	112	enet	0.053	3.20E-88	-0.24998	0.8026
SeqId_3169_70	IDUA	4	980785	998352	0.44614	rs34311866	-2.743	rs3796622	0.177052	35.77	-0.115	219	219	enet	0.34	0	-0.2491	8.03E-01
SeqId_8233_2	ITIH5	10	7601232	7708961	0.28404	rs60815829	2.74	rs6602258	0.069294	22.46	0.0281	135	135	enet	0.21	0	-0.248809	0.8035
SeqId_10851_77	EBI3	19	4229520	4237525	0.21208	rs58237134	1.263	rs4905	0.44013	-56.36	0.338	21	21	enet	0.44	0	-0.24785	0.804251
SeqId_3291_30	FCER2	19	7753644	7767032	0.10375	rs1155715	-2.419	rs62110713	0.046932	-18.52	0.164	48	48	enet	0.088	1.10E-146	-0.24646	0.80533
SeqId_12411_60	MAX	14	65472892	65569413	0.04357	rs149737749	-1.783	rs762810	0.069752	-22.63	0.307	19	19	enet	0.071	1.80E-118	-0.2449	8.07E-01
SeqId_17380_2	UBE2K	4	39699729	39784412	0.02218	rs183630707	1.989	rs58339883	0.014745	10.77	-0.152	28	28	enet	0.019	2.10E-32	-0.2439	8.07E-01
SeqId_15368_3	BMPER	7	33943920	34196039	0.0966	rs10240704	-1.903	rs16879245	0.016771	-12.73	-1.199	72	72	enet	0.072	1.20E-118	0.2438	0.807412
SeqId_3221_54	SFRP1	8	41119481	41166992	0.08218	rs79902170	-3.13	rs72643820	0.020698	-12.33	-0.685	101	101	enet	0.048	4.00E-79	-0.2427	0.8082

SeqId_4541_49	CDON	11	125825691	125933230	0.18078	rs11220352	-2.689	rs112978541	0.171934	-35.27	0.021	112	112	enet	0.2	0	0.24222	8.09E-01
SeqId_5698_60	TNXB	6	32008930	32083111	0.39131	rs2857709	-3.98	rs116298992	0.12549	-30.11	-1.233	100	100	enet	0.25	0	0.242	8.09E-01
SeqId_6451_64	ASPN	9	95218487	95244788	0.20939	rs75491213	-1.954	rs2761681	0.116	-28.97	-0.184	66	66	enet	0.18	1.40E-307	0.2413	8.09E-01
SeqId_17765_3	SNUPN	15	75890424	75918810	0.06007	rs72732875	-2.836	rs7170787	0.04807	18.69	1.581	87	87	enet	0.064	1.00E-105	0.2415	0.8092
SeqId_3449_58	SERPINA4	14	95027779	95036250	0.3268	rs2236240	-3.034	rs10139745	0.203395	38.37	-1.059	141	141	enet	0.3	0	-0.2406	8.10E-01
SeqId_2687_2	MIA	19	41277553	41283395	0.14926	rs2290693	2.629	rs2607421	0.244003	42.12	0.16	72	72	enet	0.28	0	-0.24038	0.810036
SeqId_6367_66	FMOD	1	203309756	203320886	0.07298	rs1361598	3.695	rs10920616	4.07E-02	-17.54	-0.254	36	36	enet	0.056	1.00E-92	0.24	0.810296
SeqId_3059_50	TNFSF13B	13	108903588	108960832	0.01004	rs117306149	2.3	rs150861794	0.00305	6.06	-0.678	22	22	enet	0.0074	1.60E-13	-0.236	0.8131
SeqId_18186_15	TARS	5	33440802	33468196	0.03555	rs187381727	-1.444	rs3777083	1.42E-02	-10.3	0.324	51	51	enet	0.022	1.60E-37	-0.23586	0.81354
SeqId_6382_17	MANBA	4	103551927	103682151	0.11053	rs75077651	1.417	rs223490	0.212453	39.97	0.475	45	45	enet	0.25	0	0.2351	8.14E-01
SeqId_6626_81	CHST12	7	2443223	2488119	0.06356	rs10229817	2.713	rs884566	0.041019	17.38	-1.319	47	47	enet	0.052	1.10E-85	-0.2348	0.814329
SeqId_3796_79	ANGPTL4	19	8428173	8439254	0.00643	rs77941029	1.305	rs116843064	0.014613	-10.33	-0.429	9	9	enet	0.018	3.10E-30	0.23216	0.816412
SeqId_10442_1	TMEM190	19	55888194	55889614	0.28244	rs62124644	2.036	rs4806666	0.342338	-49.7	0.276	46	46	enet	0.35	0	-0.22847	0.819282
SeqId_5731_1	SPINK6	5	147582357	147594700	0.08603	rs75816854	1.938	rs11741093	1.40E-01	31.82	-0.568	60	60	enet	0.18	1.3e-312	0.22791	0.81972
SeqId_4924_32	MMP1	11	102660132	102668891	0.21868	rs143456145	2.569	rs1144396	0.047168	18.6	-1.263	126	126	enet	0.13	9.50E-215	-0.22816	8.20E-01
SeqId_7551_33	LRRC32	11	76368568	76381791	0.01272	rs149028714	2.888	rs12275758	0.00241	-5.48	0.114	39	39	enet	0.0073	2.00E-13	0.22801	8.20E-01
SeqId_15641_20	TMEFF1	9	103235431	103339918	0.03143	rs118169670	1.725	rs10989102	0.01112	-9.47	0.136	27	27	enet	0.012	1.30E-20	-0.2282	8.20E-01
SeqId_17819_30	FAHD1	16	1876942	1890208	0.01395	rs3874648	1.27	rs3743853	0.02801	-14.41	-0.225	15	1	top1	0.028	1.10E-46	0.225	8.22E-01
SeqId_15387_44	NRP2	2	206546714	206662857	0.15705	rs2336600	2.982	rs62172729	0.0164	-11.88	-0.376	152	152	enet	0.066	9.80E-110	-0.225	8.22E-01
SeqId_5598_3	GREM2	1	240652873	240775455	0.0076	rs12410581	-1.178	rs116135446	3.70E-03	5.48	-0.223	14	1	top1	0.0037	1.40E-07	-0.223	0.823536
SeqId_4771_10	SMPDL3A	6	123110194	123130865	0.10625	rs11751996	-1.31	rs28385609	0.05807	-20.54	-0.07	11	11	enet	0.063	2.00E-104	-0.222	8.24E-01
SeqId_11659_31	CLINT1	5	157212751	157286153	0.02295	rs17598343	2.775	rs12284	1.39E-02	-10.26	-0.231	21	21	enet	0.015	4.70E-26	-0.22215	0.8242
SeqId_8766_29	LILRA5	19	54818353	54824436	0.09089	rs8106120	2.482	rs397600	0.033508	15.69	-0.0943	58	58	enet	0.056	1.70E-92	0.21955	0.826223
SeqId_5618_50	FAM3B	21	42676139	42729654	0.08533	rs66817580	2.29	rs57529409	0.044	-17.87	0.066	22	22	enet	0.074	1.30E-122	0.2182	0.827
SeqId_5584_21	TCN2	22	31002990	31023258	0.34096	rs1807515	4.03	rs12169610	0.13815	-32.35	-0.394	104	104	enet	0.34	0	0.2178	0.82759
SeqId_6366_38	TXNDC15	5	134210061	134237325	0.05044	rs319597	1.81	rs3733897	5.20E-02	19.41	0.057	20	20	enet	0.056	1.60E-92	0.21718	0.82807
SeqId_9211_19	SERPINF1	17	1665323	1680859	0.20169	rs55815331	3.368	rs62088172	0.144288	-32.4	-1.107	170	170	enet	0.18	1e-312	0.2159	0.82908
SeqId_11441_11	PYGL	14	51324609	51411201	0.1414	rs7158729	-4.162	rs12717412	0.041825	-17.56	0.212	58	58	enet	0.063	3.10E-104	-0.2152	8.30E-01
SeqId_4324_33	CST2	20	23804406	23807366	0.13742	rs150615439	2.25	rs4260306	0.100253	-27.59	-0.75	26	26	enet	0.14	2.60E-240	0.2147	0.83002
SeqId_2705_5	CCL25	19	8117202	8127544	0.20655	rs36248	-3.185	rs74959615	0.195089	-37.54	0.064	40	40	enet	0.25	0	-0.21465	0.830043
SeqId_9384_17	CAMP	3	48264837	48266981	0.00526	rs149065620	1.84	rs11716848	2.26E-03	4.32	0.292	35	35	enet	0.0065	5.00E-12	0.21355	8.31E-01
SeqId_10916_44	PLA2R1	2	160788517	160919126	0.38893	rs183026621	3.26	rs3749117	0.27831	-44.82	0.725	189	189	enet	0.37	0	-0.2105	8.33E-01
SeqId_18871_24	AIF1L	9	133971863	133998539	0.11723	rs72770746	1.906	rs11244284	0.11974	-29.44	0.316	44	44	enet	0.17	2.80E-286	-0.2113	8.33E-01
SeqId_8039_41	FAM177A1	14	35514113	35582336	0.21296	rs55791227	-2.898	rs799473	0.209536	-38.91	-0.634	77	77	enet	0.23	0	0.2097	8.34E-01
SeqId_8697_38	GPC1	2	241375080	241407493	0.11564	rs10933615	-1.69	rs1126920	0.12516	30.09	0.39	54	54	enet	0.13	2.10E-222	0.2093	8.34E-01
SeqId_16916_19	SLITRK6	13	86366925	86380818	0.07463	rs146207806	-2.67	rs9547187	0.04169	-17.51	-0.212	88	88	enet	0.056	2.00E-92	0.208	0.8355
SeqId_3232_28	ACP5	19	11685475	11689823	0.10227	rs17242899	1.671	rs2305799	0.042065	-17.6	0.637	43	43	enet	0.082	2.60E-136	-0.20697	0.836034
SeqId_2774_10	IL16	15	81451916	81606399	0.27742	rs59216753	3.078	rs4778639	0.28643	-45.64	-0.557	56	56	enet	0.3	0	0.2069	0.8361
SeqId_4568_17	SLITRK5	13	88323626	88348527	0.07672	rs185684080	3.82	rs12856670	0.0209	-12.32	0.248	109	109	enet	0.046	4.90E-76	0.207	0.8362
SeqId_7849_3	QPCT	2	37569970	37600465	0.07833	rs17462364	-2.141	rs75573663	0.02689	-15.1	-0.576	57	57	enet	0.063	2.40E-104	-0.2054	8.37E-01
SeqId_4968_50	CAPG	2	85621876	85645555	0.18254	rs12463933	-1.509	rs62623452	0.07879	-24.16	-0.404	55	55	enet	0.16	4.90E-268	0.2047	8.38E-01
SeqId_4496_60	MMP12	11	102733467	102745712	0.13413	rs72975680	-1.225	rs2276109	0.124634	-30.27	-0.039	66	66	enet	0.15	1.80E-253	-0.20292	8.39E-01
SeqId_9877_28	CRKL	22	21271695	21308035	0.05601	rs491264	2.04	rs117858197	0.01144	-9.34	-0.645	31	31	enet	0.019	1.30E-31	-0.2017	0.84017
SeqId_10977_55	UCMA	10	13263766	13276374	0.16173	rs148371284	-2.24	rs1537771	0.047975	-18.67	-0.4261	96	96	enet	0.1	8.60E-173	0.200069	0.8414
SeqId_4342_10	ICAM1	19	10381769	10397291	0.39829	rs11670097	-3.641	rs5498	0.515609	-60.99	-0.623	85	85	enet	0.53	0	0.19926	0.842061
SeqId_9884_8	PPIL1	6	36822603	36842579	0.23835	rs76207768	-2.45	rs10440825	0.10258	-27.21	0.045	17	17	enet	0.1	2.00E-172	0.198	8.43E-01
SeqId_15299_102	MESDC2	15	81238630	81282169	0.02212	rs11633987	2.28	rs73489798	0.01552	-11.25	-0.41	43	43	enet	0.019	9.30E-33	-0.197	0.8438
SeqId_3415_61	IBSP	4	88720706	88733587	0.01374	rs112229979	2.005	rs2616262	0.006083	-7.02	-0.414	31	31	enet	0.0075	1.20E-13	0.1968	8.44E-01
SeqId_15573_110	NCAN	19	19322767	19363042	0.03004	rs75072017	-2.358	rs2228603	0.030074	-14.79	-0.196	24	1	top1	0.03	5.00E-50	0.196	0.84461
SeqId_7761_125	CHKB	22	51017388	51039884	0.04118	rs73187217	-1.84	rs5770922	0.02275	-12.97	-0.242	34	34	enet	0.03	1.90E-49	0.1956	0.84494
SeqId_7227_75	COCH	14	31343720	31364271	0.29442	rs10483371	-3.09	rs28400019	0.107776	27.91	-2.1625	115	115	enet	0.19	0	0.1954	8.45E-01
SeqId_3343_1	ACY1	3	52017356	52023213	0.03411	rs184102325	1.35	rs148804382	1.06E-02	-9.14	0.196	18	1	top1	0.011	1.10E-18	-0.196	8.45E-01
SeqId_6986_17	HS3ST3B1	17	14204398	14252721	0.02113	rs72815717	-2.705	rs62056073	0.00678	-8.17	0.267	27	27	enet	0.015	1.10E-25	-0.1952	0.84525

SeqId_14747_9	CRLF1	19	18683030	18718551	0.03827	rs2161106	-1.752	rs141412226	0.036998	16.4	-0.0153	29	29	enet	0.037	8.30E-62	0.1949	0.845472
SeqId_6342_10	NPNT	4	106815932	106925184	0.13865	rs72669966	2.513	rs34712979	0.055173	-20.06	-0.412	57	57	enet	0.059	8.70E-97	0.1938	8.46E-01
SeqId_16606_85	AKR1B1	7	134127108	134144036	0.08976	rs782534	2.197	rs782546	0.023028	13.31	1.838	37	37	enet	0.044	4.70E-73	-0.1931	0.846914
SeqId_5740_17	ROBO1	3	78646389	79817148	0.09102	rs10049012	1.82	rs3773232	3.55E-02	16.2	-0.435	53	53	enet	0.054	1.50E-88	-0.19133	8.48E-01
SeqId_3175_51	ADAMTS13	9	136279478	136324524	0.12662	rs7864157	13.232	rs35516997	0.13264	-31.06	-2.395	37	37	enet	0.19	0	-0.1897	8.50E-01
SeqId_11382_5	BLVRA	7	43798279	43846941	0.1181	rs145849190	-1.84	rs1050916	0.020426	-14.32	0.282	51	51	enet	0.067	3.00E-111	-0.1891	0.850007
SeqId_2913_1	CCL23	17	34340096	34345009	0.43285	rs113688187	2.983	rs712048	0.071247	-22.79	1.026	126	126	enet	0.26	0	0.1889	0.85014
SeqId_3858_5	ACP1	2	264140	278283	0.29912	rs143938482	3.039	rs79716074	0.48529	59.17	-0.179	102	102	enet	0.49	0	-0.1878	8.51E-01
SeqId_6081_52	PCOLCE2	3	142534764	142608048	0.32173	rs9860003	2.76	rs11716897	1.09E-01	28.12	-0.302	96	96	enet	0.23	0	0.18672	8.52E-01
SeqId_3322_52	LRIG3	12	59265931	59314285	0.13873	rs147407935	-2.49	rs11172791	0.055191	-21.04	0.197	88	88	enet	0.18	5.8e-319	-0.18493	0.85328
SeqId_5621_64	THSD1	13	52951302	52990508	0.28705	rs3154	2.53	rs41292808	0.11142	28.4	0.349	60	60	enet	0.12	4.50E-208	0.184	0.8537
SeqId_6930_95	ST8SIA6	10	17357420	17496594	0.02534	rs7096979	3.13	rs11595070	0.006679	-8.29	0.7052	44	44	enet	0.024	1.10E-40	-0.182289	0.8554
SeqId_5810_25	TDGF1	3	46616024	46623947	0.42487	rs11709020	1.51	rs3806702	4.81E-01	58.94	0.049	27	27	enet	0.49	0	-0.18095	8.56E-01
SeqId_17453_34	CP	3	148880197	148939616	0.02258	rs13089226	-2	rs13089226	5.98E-03	6.68	-1.999	45	45	enet	0.0092	1.80E-16	-0.17779	8.59E-01
SeqId_19113_66	SH3BGRL2	6	80341046	80413372	0.10395	rs116843610	1.61	rs2245410	0.08898	25.43	-0.214	44	44	enet	0.1	4.20E-169	-0.177	8.60E-01
SeqId_15487_164	CES1	16	55836763	55867249	0.05679	rs143216460	-2.42	rs76336259	0.01814	-11.64	0.931	66	66	enet	0.027	1.30E-45	0.1747	8.61E-01
SeqId_3461_58	BCAN	1	156611182	156629324	0.14819	rs115620618	1.957	rs2365715	4.59E-02	-18.31	0.775	28	28	enet	0.063	4.70E-105	-0.175	0.861042
SeqId_11646_4	CHST9	18	24486445	24765272	0.04417	rs675150	2.21	rs11660451	0.03907	17	-1.102	41	41	enet	0.062	1.10E-102	0.175	0.8611
SeqId_9191_8	TFF2	21	43766466	43771106	0.00774	rs58569031	-1.5	rs58206891	0.00226	-5.83	0.619	32	32	enet	0.0067	2.00E-12	-0.1727	0.863
SeqId_3457_57	POSTN	13	38136720	38172981	0.08906	rs9576354	3.23	rs7329947	0.02727	14.08	-1.169	100	100	enet	0.058	2.20E-95	-0.168	0.8667
SeqId_16060_99	NID2	14	52471521	52535946	0.10243	rs144186852	2.221	rs941622	0.125698	30.22	-0.088	74	74	enet	0.16	7.10E-277	-0.1671	8.67E-01
SeqId_3043_49	SPARC	5	151040657	151066536	0.03817	rs6894313	-2.156	rs59311424	8.56E-03	-8.05	-0.296	32	32	enet	0.02	4.70E-34	-0.16587	0.86826
SeqId_2602_2	ANGPT2	8	6357153	6420930	0.04789	rs1014199	1.892	rs1968586	0.021563	-12.56	0.3013	70	70	enet	0.027	4.40E-45	-0.165	0.868965
SeqId_6507_16	NCAM2	21	22370727	22915650	0.31831	rs75296494	2.34	rs2826851	0.12302	29.83	-1.122	102	102	enet	0.23	0	0.1632	0.87
SeqId_5660_51	SOD3	4	24791534	24802464	0.18885	rs113089419	2.647	rs2536512	0.047282	18.6	-0.11	213	213	enet	0.12	3.40E-199	-0.1633	8.70E-01
SeqId_10037_98	SIGLEC12	19	51994481	52005054	0.21827	rs2168643	-1.614	rs3826667	0.311978	-47.46	-0.1648	16	16	enet	0.32	0	0.16304	0.87049
SeqId_19207_119	ADK	10	75910960	76469721	0.01075	rs2227579	2.06	rs7099085	0.001771	5.95	0.82	41	41	enet	0.0054	3.00E-10	0.162408	0.871
SeqId_6470_19	FBLN1	22	45898118	45997015	0.07769	rs13053621	2.16	rs11090631	0.03579	-16.25	0.57	87	87	enet	0.058	4.90E-96	0.1579	0.87454
SeqId_16558_2	MYOC	1	171604557	171621828	0.2632	rs78578111	-2.589	rs235879	9.18E-02	25.76	-0.636	131	131	enet	0.2	0	0.1555	0.876408
SeqId_9076_25	PENK	8	57349233	57359230	0.10137	rs16920917	2.753	rs2576581	0.14522	-32.4	-0.13	82	82	enet	0.2	0	0.1537	0.877851
SeqId_17686_27	TBCB	19	36605191	36616849	0.096	rs113300978	1.975	rs2231569	0.053681	19.73	-0.521	39	39	enet	0.065	6.00E-107	-0.15312	0.878304
SeqId_4498_62	NCAM1	11	112831969	113149158	0.19808	rs74800050	2.249	rs2288158	0.048359	18.72	-0.52	115	115	enet	0.13	5.50E-225	0.15143	8.80E-01
SeqId_8297_8	DNAJC10	2	183580982	183659191	0.04477	rs74375064	2.162	rs288334	0.01949	12.23	0.303	43	43	enet	0.033	2.30E-55	-0.1471	8.83E-01
SeqId_3196_6	HAPLN1	5	82933624	83016674	0.03075	rs17206048	-2.666	rs6886442	6.33E-03	8.96	0.207	47	47	enet	0.016	2.20E-27	-0.145	0.88471
SeqId_3855_56	PRDX1	1	45976586	46008404	0.08447	rs11211120	2.074	rs11211133	4.43E-02	17.95	-0.982	40	40	enet	0.075	5.50E-124	0.1429	0.886378
SeqId_8014_359	MANEA	6	96025361	96057346	0.33329	rs140487152	2.63	rs13205436	0.32064	-48.12	0.286	92	92	enet	0.42	0	-0.141	8.88E-01
SeqId_11308_8	CREB3L4	1	153940010	153946837	0.02709	rs111689685	-1.116	rs4845586	4.09E-02	17.3	0.119	16	16	enet	0.042	3.70E-70	-0.1386	0.889801
SeqId_14203_3	ANXA7	10	75134859	75173834	0.15699	rs7898735	2.69	rs76150063	0.00791	-7.73	0.881	28	28	enet	0.017	2.10E-29	-0.137815	0.8904
SeqId_4834_61	EPHA2	1	16450832	16482564	0.14755	rs115041526	2.082	rs6690317	6.44E-02	-22.11	0.545	49	49	enet	0.094	1.80E-157	0.1378	0.890402
SeqId_10088_37	APRT	16	88875747	88878345	0.01993	rs8060375	-2.39	rs535070	0.00051	-6.28	0.47	45	45	enet	0.012	3.40E-21	0.137	8.91E-01
SeqId_17680_12	EPHB1	3	134514102	134979309	0.0805	rs142888914	-2.31	rs10935143	7.39E-02	23.74	-0.404	67	67	enet	0.12	2.70E-201	-0.13682	8.91E-01
SeqId_3298_52	CNTN4	3	2140497	3099643	0.16705	rs72997999	-2.24	rs13071423	3.54E-02	17.3	0.469	160	160	enet	0.11	3.00E-183	-0.13738	8.91E-01
SeqId_19262_219	ACADVL	17	7120444	7128585	0.0055	rs111901268	-0.383	rs446994	0.002298	-5.73	0.137	11	1	top1	0.0023	2.70E-05	-0.137	0.89103
SeqId_13733_5	IL12B	5	158741788	158757495	0.16095	rs80205166	1.7	rs4244437	1.47E-01	32.65	0.347	30	30	enet	0.19	0	0.13584	0.89195
SeqId_4292_5	NAPA	19	47990894	48018348	0.04094	rs296371	1.369	rs71363738	0.022055	12.71	-0.076	26	26	enet	0.03	3.50E-49	-0.13442	0.893072
SeqId_18216_22	IL11RA	9	34650699	34661899	0.08134	rs142369632	-2.424	rs11575578	0.10712	27.91	0.3	39	39	enet	0.11	6.10E-187	0.132	8.95E-01
SeqId_17383_4	STARD5	15	81601394	81616524	0.10016	rs111430118	2.358	rs28555408	0.03409	-15.77	0.933	71	71	enet	0.083	1.80E-137	0.129	0.8974
SeqId_13488_3	ARFIP1	4	153701089	153839615	0.04967	rs6535908	1.866	rs4619875	0.08699	25.07	-0.126	28	1	top1	0.087	6.00E-145	-0.126	9.00E-01
SeqId_4129_72	CFB	6	31913427	31919861	0.23237	rs9267480	-3.71	rs541862	0.11286	28.59	-0.577	80	80	enet	0.21	0	0.126	9.00E-01
SeqId_18403_25	AMPD2	1	110158726	110174673	0.01093	rs17024258	3.457	rs523786	2.54E-03	-5.42	-0.124	48	1	top1	0.0025	1.10E-05	0.124	0.901315
SeqId_2652_15	PLAUR	19	44150247	44174699	0.15225	rs373560341	2.954	rs2302524	0.04725	-18.67	0.752	85	85	enet	0.081	3.60E-134	0.12277	0.902289
SeqId_4413_3	SLPI	20	43880880	43883205	0.05447	rs34643859	2.58	rs2143943	0.02467	13.43	0.758	59	59	enet	0.04	3.90E-66	0.1225	0.90253
SeqId_5066_134	CD300C	17	72537212	72542254	0.08432	rs62063445	2.19	rs62087200	0.054569	-19.91	0.189	37	37	enet	0.068	5.30E-112	-0.1219	0.90298

SeqId_2567_5	CFI	4	110661850	110723155	0.12491	rs17041514	-3.033	rs7439493	0.066968	22.03	-0.11	124	124	enet	0.091	1.30E-151	0.1217	9.03E-01
SeqId_5463_22	GAS1	9	89559277	89562421	0.05558	rs76595492	-2.817	rs4878043	0.00856	9.32	0.196	58	58	enet	0.041	3.20E-67	-0.1214	9.03E-01
SeqId_14636_25	HRSP12	8	99114572	99129399	0.07899	rs113019046	-1.042	rs10955148	0.071971	-22.86	0.326	11	11	enet	0.078	2.70E-130	-0.1209	0.903735
SeqId_18236_3	HSPC159	2	64681103	64688515	0.04789	rs12713506	-2.786	rs76392037	0.03336	15.56	-0.282	42	42	enet	0.038	2.80E-63	0.1208	9.04E-01
SeqId_16913_8	RNASET2	6	167335601	167370679	0.1956	rs9295358	-3.13	rs9457245	0.09817	-26.7	-0.646	66	66	enet	0.16	2.00E-270	0.121	9.04E-01
SeqId_7628_40	CRELD1	3	9975477	9987097	0.22371	rs17032711	-2.7	rs6762702	3.42E-01	-49.7	-0.243	72	72	enet	0.4	0	0.11974	9.05E-01
SeqId_19365_11	BCAT2	19	49298319	49314286	0.1074	rs2638282	3.722	rs62125920	0.108027	-27.97	0.27	69	69	enet	0.12	4.50E-194	-0.11917	0.905144
SeqId_16621_77	APOA1BP	1	156561548	156579299	0.09228	rs2365715	-2.337	rs41267389	1.19E-01	-29.5	0.522	87	87	enet	0.14	3.80E-241	-0.1192	0.905147
SeqId_3508_78	CCL22	16	57392695	57400101	0.06803	rs192297333	2.46	rs170364	0.01632	11.77	0.893	114	114	enet	0.046	2.50E-76	-0.118	9.06E-01
SeqId_13552_7	SWAP70	11	9685624	9774540	0.20568	rs405449	2.326	rs10770059	0.171952	-35.27	-0.555	58	58	enet	0.2	0	0.11573	9.08E-01
SeqId_18340_2	PSMB4	1	151372036	151374413	0.07949	rs114873697	-3.035	rs4603	1.52E-01	-33.23	0.113	52	1	top1	0.15	1.40E-260	-0.113	0.910031
SeqId_15515_2	SAA1	11	18287807	18291524	0.21937	rs7946133	1.652	rs11024589	0.12056	-29.54	-0.099	45	45	enet	0.15	2.00E-264	-0.11097	9.12E-01
SeqId_2998_53	JAM3	11	133938966	134021896	0.02191	rs1259022	1.652	rs655627	0.022363	12.82	0.635	12	12	enet	0.024	1.70E-39	0.10837	9.14E-01
SeqId_5275_28	VAV1	19	6772719	6857377	0.04554	rs112152495	-1.606	rs36097961	0.048077	-18.69	0.22	34	34	enet	0.05	5.20E-82	-0.10761	0.914306
SeqId_8221_19	MIF	22	24236570	24237414	0.03659	rs5759855	1.56	rs5751764	0.03418	-15.92	0.09	39	39	enet	0.039	3.70E-64	0.1068	0.91493
SeqId_17675_17	ACOT13	6	24667263	24705293	0.0863	rs114272893	1.94	rs4544900	0.0259	14.4	-0.047	33	33	enet	0.054	1.10E-88	-0.107	0.915
SeqId_15414_316	LDHA	11	18416107	18429972	0.01466	rs35946818	1.696	rs7112492	0.009599	-8.73	0.261	25	25	enet	0.01	9.80E-18	0.10586	9.16E-01
SeqId_18878_15	GREM1	15	33010205	33037307	0.22825	rs10519739	1.63	rs2293582	0.24448	42.06	0.01	35	35	enet	0.26	0	-0.1004	0.92
SeqId_15375_49	CPB1	3	148508889	148577974	0.13106	rs12489871	-2.02	rs13318853	5.91E-02	20.72	0.15	44	44	enet	0.089	4.70E-148	-0.09933	9.21E-01
SeqId_15626_223	HSPG2	1	22148737	22263803	0.0756	rs7529220	2.143	rs12742444	5.73E-02	20.58	0.067	64	64	enet	0.063	3.20E-104	-0.099	0.921143
SeqId_11476_43	VPS4A	16	69345253	69360842	0.00536	rs72801310	-1.33	rs9924894	0.00509	6.98	0.098	15	1	top1	0.0051	7.90E-10	0.098	9.22E-01
SeqId_19614_8	TCN1	11	59620273	59633962	0.27295	rs2165832	3.057	rs34324219	0.054065	-21.11	-1.007	42	42	enet	0.13	1.40E-213	0.09675	9.23E-01
SeqId_16858_384	RCN3	19	50031260	50050219	0.00707	rs11554772	2.79	rs73582441	0.001103	4.32	0.096	52	1	top1	0.0011	0.0028	0.096	0.923521
SeqId_12831_21	TESC	12	117476728	117537284	0.05071	rs7297446	1.324	rs10850746	0.055747	-20.23	0.028	33	33	enet	0.063	6.70E-105	0.09484	0.92444
SeqId_6998_106	ASPH	8	62413115	62627199	0.05766	rs17208884	2.895	rs11775077	0.025757	-15.08	0.764	79	79	enet	0.047	1.40E-77	0.0939	0.925217
SeqId_8992_1	TMEM2	9	74298282	74431606	0.03624	rs17057093	-1.782	rs1410988	0.02284	13.09	0.093	9	1	top1	0.023	2.50E-38	0.093	9.26E-01
SeqId_3378_49	KLK7	19	51479724	51487355	0.09106	rs1654531	-2.581	rs1654523	0.05244	19.66	1.212	83	83	enet	0.07	3.50E-116	0.0926	0.92622
SeqId_6491_59	KLK15	19	51328545	51340469	0.02132	rs59300606	-2.344	rs73048483	0.01775	11.5	1.216	27	27	enet	0.018	5.20E-31	0.09231	0.926451
SeqId_12643_4	ARRB1	11	74971166	75062749	0.13292	rs139001251	2.935	rs504683	0.1173	29.15	-0.214	56	56	enet	0.13	9.10E-229	-0.09178	9.27E-01
SeqId_15589_1	GC	4	72607413	72669758	0.03051	rs4588	-1.937	rs7697091	0.008557	-9.31	-1.659	49	49	enet	0.018	2.90E-31	0.0898	9.28E-01
SeqId_19173_5	ZFAND1	8	82613569	82645138	0.15425	rs144200366	3.023	rs2912805	0.133522	31.12	0.866	78	78	enet	0.24	0	0.0898	0.92843
SeqId_3212_30	ASAH2	10	51942538	52039480	0.3705	rs3011759	2.59	rs2842126	0.134254	31.21	0.05	108	108	enet	0.26	0	-0.0894	0.9288
SeqId_8229_1	GXYLT1	12	42475647	42538676	0.22484	rs151161288	-2.045	rs139695917	0.076779	23.59	0.638	52	52	enet	0.14	1.30E-230	0.08933	0.92882
SeqId_14079_14	IL18R1	2	102927989	103015237	0.16442	rs12618571	2.622	rs2001461	0.27917	-44.98	-0.595	90	90	enet	0.32	0	0.0894	9.29E-01
SeqId_13986_6	LANCL1	2	211295973	211342376	0.01485	rs116012745	1.261	rs17552879	0.00888	8.13	-0.119	21	21	enet	0.012	1.30E-20	0.0874	9.30E-01
SeqId_5737_61	SEMA4D	9	91975702	92113045	0.24102	rs75249546	1.944	rs45464494	0.28016	-45	0.072	53	53	enet	0.31	0	-0.0881	9.30E-01
SeqId_6527_1	TRIL	7	28992974	28997946	0.03246	rs10264676	-1.567	rs740250	0.018422	-12	-0.044	30	30	enet	0.02	2.60E-34	0.0875	0.930298
SeqId_8225_86	EPHB2	1	23037332	23247993	0.15603	rs10799764	-2.406	rs2043970	1.00E-01	-27.07	0.14	54	54	enet	0.11	1.30E-193	-0.087	0.930705
SeqId_13621_31	AP2A2	11	924894	1012245	0.02303	rs72850167	2.435	rs4963151	0.012521	-10.08	-0.411	41	41	enet	0.019	1.10E-32	-0.08686	9.31E-01
SeqId_18813_15	DDX19A	16	70380764	70407286	0.0591	rs71401807	-2.8	rs138325325	0.02909	-14.75	-0.786	24	24	enet	0.031	4.00E-52	-0.0869	9.31E-01
SeqId_18338_26	IDH1	2	209100953	209130798	0.23911	rs115733739	3.068	rs3928183	0.06312	23.35	-0.716	47	47	enet	0.15	3.20E-257	-0.086	9.31E-01
SeqId_15524_30	PGAM2	7	44102326	44105166	0.00649	rs62459096	-1.413	rs6956492	0.003559	-6.6	0.331	17	17	enet	0.0051	6.40E-10	0.086	0.931505
SeqId_19615_213	NT5C3A	7	33053742	33102408	0.03032	rs4316067	-1.687	rs12155117	0.013747	-10.32	0.07	37	37	enet	0.02	2.10E-33	-0.0856	0.931779
SeqId_15476_6	REG3G	2	79252812	79255631	0.12057	rs7595032	2.426	rs430298	0.11659	29.07	0.566	89	89	enet	0.19	0	-0.0853	9.32E-01
SeqId_4209_60	VTA1	6	142468299	142545822	0.04631	rs117148924	-2.32	rs225628	0.03434	-15.87	-0.525	43	43	enet	0.061	2.10E-101	-0.084	9.33E-01
SeqId_3459_49	PDGFRB	5	149493402	149535435	0.51855	rs111964094	-1.971	rs3816018	5.35E-01	62.13	-0.034	83	83	enet	0.55	0	0.08228	0.93442
SeqId_3607_71	DKK3	11	11977754	12031316	0.24785	rs72860953	-2.404	rs11022114	0.097969	26.61	0.471	89	89	enet	0.14	5.70E-232	0.07964	9.37E-01
SeqId_2609_59	CST3	20	23607343	23619110	0.11393	rs71338673	1.59	rs2405367	0.117335	-29.54	-0.00493	61	61	enet	0.15	8.50E-262	0.0778	0.93798
SeqId_2992_59	IL17RA	22	17565844	17596584	0.70416	rs147797180	-3.6	rs4819959	0.31633	47.81	-0.586	342	342	enet	0.49	0	-0.0766	0.93893
SeqId_18215_5	THG1L	5	157158428	157168457	0.42884	rs11134934	-1.235	rs2270812	3.22E-01	48.21	-0.178	42	42	enet	0.35	0	-0.07441	0.94069
SeqId_3181_50	CTSS	1	150702555	150738433	0.2077	rs6694485	-1.965	rs41271951	1.68E-01	-34.81	0.679	45	45	enet	0.21	0	-0.0701	0.944101
SeqId_9599_6	PIANP	12	6802957	6809981	0.06538	rs870849	1.818	rs11064306	0.028713	14.59	-1.242	34	34	enet	0.044	6.30E-73	0.06944	0.94464
SeqId_8289_8	GPNMB	7	23275586	23314727	0.15426	rs191221122	2.527	rs28458177	0.042565	-17.6	-0.14	92	92	enet	0.09	3.30E-150	0.0674	0.946249

SeqId_15475_4	PLTP	20	44527260	44540794	0.18125	rs6124739	2.64	rs111602331	0.161079	-34.32	-0.296	56	56	enet	0.2	0	-0.0674	0.94629
SeqId_6252_62	SCGB3A1	5	180017105	180018499	0.01746	rs139067380	2.538	rs307802	5.49E-03	6.87	-0.461	24	24	enet	0.0088	8.00E-16	-0.06519	0.94802
SeqId_6245_4	PVRL2	19	45349432	45392480	0.02087	rs123187	2.158	rs440277	0.00768	-7.93	0.794	61	61	enet	0.0087	1.10E-15	-0.06372	0.949193
SeqId_3453_87	LYN	8	56792394	56926728	0.0124	rs61753686	2.193	rs1050855	0.005572	-8.21	-0.02	16	16	enet	0.0095	5.70E-17	-0.063	0.949801
SeqId_18244_1	ANXA7	10	75134859	75173834	0.15979	rs3829126	2.62	rs76150063	0.005603	-7.29	0.881	38	38	enet	0.016	5.00E-27	0.061669	0.9508
SeqId_9360_33	EDIL3	5	83236373	83680698	0.01274	rs17206390	1.764	rs10073371	3.52E-03	-5.87	0.063	32	32	enet	0.0094	1.10E-16	0.05718	0.9544
SeqId_15323_112	AGO3	1	36396319	36538101	0.00425	rs6682536	2.376	rs139140696	-2.03E-05	-3.39	1.107	8	8	enet	1.90E-05	0.29	-0.0561	0.955234
SeqId_8963_8	VTI1B	14	68113802	68141537	0.01705	rs17184650	2.391	rs10483801	0.008382	7.9	0.743	33	33	enet	0.013	3.20E-22	0.0535	9.57E-01
SeqId_5061_27	ICOSLG	21	45636897	45660849	0.0605	rs148056932	2.36	rs2838537	0.00958	-9.14	-0.049	58	58	enet	0.025	4.80E-42	0.0533	0.957
SeqId_3363_31	CSK	15	75074421	75095538	0.0857	rs57437866	1.295	rs34933034	0.06048	21.89	-0.062	24	24	enet	0.076	3.50E-126	0.0537	0.9572
SeqId_16324_38	TLR1	4	38792298	38858438	0.03415	rs114456163	1.38	rs5743618	0.033485	15.64	0.376	22	22	enet	0.044	6.20E-72	-0.053	9.58E-01
SeqId_15576_158	RNASE3	14	21359544	21360507	0.19237	rs4981353	-2.071	rs147307766	0.077446	24.79	-0.007	181	181	enet	0.17	1.30E-298	0.0499	9.60E-01
SeqId_7787_25	LILRA5	19	54818353	54824436	0.06746	rs62133394	-1.604	rs397600	0.025155	13.63	0.2265	37	37	enet	0.042	8.60E-70	0.0477	0.961959
SeqId_10974_20	SPINK7	5	147691982	147695485	0.13945	rs79778755	-1.909	rs9784645	3.31E-02	15.62	-0.014	65	65	enet	0.054	7.50E-89	0.04676	0.9627
SeqId_5088_175	IL23R	1	67604590	67725662	0.01538	rs112937404	1.115	rs12569203	2.40E-03	-6.15	0.481	29	29	enet	0.0071	4.20E-13	-0.0465	0.962927
SeqId_3325_2	MATN2	8	98881068	99048952	0.14341	rs112052141	-2.567	rs17831160	0.018852	12.54	1.355	100	100	enet	0.077	4.10E-128	0.0455	0.963738
SeqId_15452_5	NT5E	6	86159302	86205509	0.13098	rs78724322	1.99	rs6903114	0.09176	-25.82	-0.318	51	51	enet	0.15	2.40E-259	0.044	9.65E-01
SeqId_8397_147	QSOX2	9	139098179	139137658	0.16031	rs55846135	-3.215	rs10858248	0.10195	-27.18	0.406	31	31	enet	0.14	1.40E-239	-0.0434	9.65E-01
SeqId_3805_16	ESM1	5	54273695	54318499	0.05286	rs114063279	2.571	rs4242051	1.20E-02	9.41	-0.126	74	74	enet	0.026	1.30E-42	-0.04286	0.96582
SeqId_9172_69	MMP8	11	102582527	102597781	0.05173	rs35231465	-2.143	rs11225395	0.024796	-13.92	0.73	53	53	enet	0.034	2.30E-56	-0.03975	9.68E-01
SeqId_17796_15	IMPA1	8	82569149	82598566	0.01428	rs10958048	-1.556	rs2142316	0.007294	-8.23	0.352	23	23	enet	0.014	7.50E-25	0.04	0.968093
SeqId_8923_94	GALNT16	14	69725994	69823750	0.1643	rs77546080	4.327	rs12100668	0.046997	18.51	0.251	85	85	enet	0.14	8.70E-232	0.0391	9.69E-01
SeqId_12016_60	CBL	11	119077008	119184636	0.08607	rs58412813	2.76	rs76683040	0.062861	-21.75	-0.263	89	89	enet	0.096	3.70E-161	0.03771	9.70E-01
SeqId_17331_138	KREMEN1	22	29469023	29564321	0.23183	rs141192812	3.17	rs2205771	0.04595	18.26	-0.144	149	149	enet	0.086	1.30E-142	0.0371	0.97041
SeqId_16825_20	ATXN3	14	92510840	92572965	0.12546	rs1241688	-2.487	rs1051340	0.155344	-33.53	0.371	82	82	enet	0.25	0	-0.0366	9.71E-01
SeqId_16620_26	LY75	2	160659866	160761267	0.49106	rs77592069	-2.627	rs56186131	0.36712	51.38	0.772	230	230	enet	0.49	0	0.0368	9.71E-01
SeqId_15470_11	HEXB	5	73935848	74018472	0.17768	rs12189470	3.356	rs13164140	7.92E-02	23.94	0.362	52	52	enet	0.15	1.50E-259	0.03566	0.97156
SeqId_15594_47	HTRA1	10	124218067	124274423	0.05346	rs78014377	-3.14	rs7074542	0.014598	-10.57	0.191	58	58	enet	0.038	2.90E-63	0.034549	0.9724
SeqId_19392_6	DDAH1	1	85784164	86044046	0.01065	rs34028770	-2.069	rs233071	6.87E-03	-7.16	-0.033	45	1	top1	0.0069	1.10E-12	0.033	0.973675
SeqId_4564_2	PLXNC1	12	94542353	94701451	0.50853	rs7136023	2.778	rs7971713	0.143891	-32.23	-0.633	85	85	enet	0.34	0	-0.03183	0.97461
SeqId_9960_2	TBC1D5	3	17198654	18486309	0.00519	rs17043133	2	rs11128865	-1.97E-05	-3.79	-1.723	14	14	enet	0.0012	0.0016	0.0312	9.75E-01
SeqId_11266_8	SELPLG	12	109015671	109027670	0.06039	rs17040551	-1.993	rs73191242	0.038126	-16.62	0.696	41	41	enet	0.054	9.30E-89	-0.03013	0.97596
SeqId_9223_11	MANF	3	51422722	51426828	0.04422	rs74776103	-1.68	rs4611808	2.95E-02	-14.73	0.036	24	24	enet	0.037	5.00E-61	0.03015	9.76E-01
SeqId_17224_12	OGN	9	95145550	95166978	0.06675	rs144889947	1.157	rs10992291	0.15145	33.09	0.119	45	45	enet	0.16	6.00E-271	0.0307	9.76E-01
SeqId_9595_11	B4GALT2	1	44444615	44456842	0.11764	rs530373	-1.65	rs2231280	9.07E-02	-25.77	-0.173	57	57	enet	0.11	1.30E-185	0.0291	0.976768
SeqId_11347_9	TALDO1	11	747415	765012	0.00828	rs28679631	-2.122	rs10902210	0.003125	5.64	-0.029	20	1	top1	0.0031	1.20E-06	-0.029	9.77E-01
SeqId_10565_19	SLITRK3	3	164904508	164914897	0.15358	rs62293358	-2.4	rs62282371	6.96E-02	-22.66	0.367	115	115	enet	0.092	1.30E-153	-0.02925	9.77E-01
SeqId_8814_33	PSAPL1	4	7432012	7436657	0.16502	rs139793474	-2.697	rs35791045	0.095429	26.28	-0.572	48	48	enet	0.15	6.20E-248	0.0284	9.77E-01
SeqId_16613_3	CDH17	8	95139390	95229531	0.12122	rs74567034	2.531	rs12056840	0.057239	-20.42	0.5741	54	54	enet	0.13	4.20E-212	0.028	0.977647
SeqId_13519_112	ARHGEF25	12	58003963	58011028	0.04585	rs117405300	3.022	rs10437954	0.016808	-11.19	1.383	34	34	enet	0.021	2.70E-35	0.02783	0.97779
SeqId_5000_52	LGALS3BP	17	76967320	76976029	0.03306	rs181483393	-1.853	rs3826311	0.010426	10.05	0.0031	71	71	enet	0.03	3.40E-49	-0.0268	0.97864
SeqId_3038_9	CXCL11	4	76954835	76962568	0.02849	rs13111888	-1.655	rs6827617	0.015654	-11.29	-0.038	33	33	enet	0.026	7.50E-43	0.0228	9.82E-01
SeqId_6478_2	IGLON5	19	51814898	51834145	0.05843	rs79814518	1.995	rs7246422	0.012259	10.35	0.495	59	59	enet	0.038	1.50E-62	-0.02244	0.982099
SeqId_9177_6	FAM3B	21	42676139	42729654	0.28621	rs4818230	2.68	rs57529409	0.1191	-29.39	0.066	85	85	enet	0.27	0	0.0202	0.984
SeqId_7128_9	VWA2	10	115999013	116054248	0.15702	rs61869891	2.43	rs35060624	0.029364	15.29	1.55	84	84	enet	0.11	3.50E-179	0.019292	0.9846
SeqId_19188_21	NAP1L4	11	2965661	3013607	0.13572	rs118092312	-1.732	rs4758622	0.238863	41.53	-0.042	38	38	enet	0.24	0	0.01833	9.85E-01
SeqId_7921_65	FJX1	11	35640008	35642413	0.07853	rs4265577	1.821	rs10768174	0.036659	-16.41	0.118	31	31	enet	0.053	8.30E-87	-0.01865	9.85E-01
SeqId_4160_49	MMP2	16	55423612	55540603	0.01655	rs12598247	-2.59	rs171497	0.00595	-6.83	0.017	75	1	top1	0.006	3.20E-11	-0.017	9.86E-01
SeqId_5078_82	EPHB6	7	142552823	142568847	0.20075	rs117831221	2.129	rs7789303	0.140539	31.88	0.3455	47	47	enet	0.16	7.50E-285	0.0161	0.987176
SeqId_14675_20	EIF4B	12	53400066	53435999	0.00841	rs117769421	2.912	rs115019703	0.002832	-4.7	-0.016	23	1	top1	0.0028	3.60E-06	0.016	0.98723
SeqId_8007_19	CTSB	8	11700033	11727042	0.26132	rs62489352	4.186	rs1736081	0.205617	38.57	-0.798	150	150	enet	0.25	0	0.0159	0.987328
SeqId_13748_4	CCL8	17	32646454	32648421	0.29104	rs78651143	-2.855	rs3138037	0.344393	-49.88	0.109	50	50	enet	0.38	0	0.0153	0.98783
SeqId_13700_10	ANXA2	15	60639333	60695082	0.10752	rs9920812	-2.074	rs12907068	0.06653	22.92	0.038	67	67	enet	0.11	1.70E-193	-0.0133	0.9894

SeqId_18875_125	COL2A1	12	48366750	48398337	0.07915	rs35299693	1.52	rs719008	0.093752	-26.12	-0.277	30	30	enet	0.11	1.20E-182	0.01172	0.99065
SeqId_17151_84	IRF3	19	50162826	50169132	0.17296	rs148314679	3.104	rs10415576	0.096355	-26.39	0.057	62	62	enet	0.13	6.80E-228	-0.01126	0.991016
SeqId_19617_5	PTGR1	9	114312002	114362173	0.28758	rs140363385	2.799	rs7036102	0.09922	28.27	-0.101	119	119	enet	0.27	0	-0.0094	9.93E-01
SeqId_8773_172	EMILIN3	20	39988602	39995458	0.20007	rs76710286	3.23	rs4810316	0.062248	21.3	-0.059	88	88	enet	0.13	3.90E-224	0.006	0.99522
SeqId_7806_33	B4GALT7	5	177027133	177037331	0.02674	rs115220376	1.856	rs59621415	1.65E-02	11.35	-0.259	35	35	enet	0.026	8.90E-43	0.00594	0.99526
SeqId_8360_169	NCR1	19	55417506	55427508	0.06361	rs73053177	2.895	rs2915993	0.011829	-10.48	0.058	60	60	enet	0.037	2.80E-61	-0.00557	0.995557
SeqId_14134_49	AMIGO2	12	47469490	47473742	0.03169	rs73098714	3.037	rs1101751	0.008281	-8.21	0.4	54	54	enet	0.017	1.90E-28	0.00446	0.99644
SeqId_9313_27	CBLN1	16	49311828	49315749	0.34996	rs117761915	-2.79	rs12596832	0.26051	-43.5	-0.274	226	226	enet	0.31	0	0.0037	9.97E-01
SeqId_2635_61	LAYN	11	111411005	111432470	0.03435	rs28690953	-0.566	rs4938792	0.028963	-14.78	-0.219	13	13	enet	0.03	1.60E-50	0.00249	9.98E-01
SeqId_17350_13	CHMP2B	3	87276421	87304706	0.07533	rs139351833	2.21	rs300978	1.45E-01	32.35	-0.149	50	50	enet	0.16	4.60E-269	0.0024	9.98E-01
SeqId_8398_277	RARRES1	3	158414681	158450278	0.39465	rs77413822	-1.96	rs4680458	1.77E-01	35.82	-0.796	62	62	enet	0.31	0	-0.00266	9.98E-01
SeqId_14054_17	IL15RA	10	5985602	6020150	0.38044	rs72781949	-2.38	rs8177643	0.187488	36.81	-0.233	96	96	enet	0.35	0	-0.000281	0.9998
SeqId_11302_237	TNR	1	175284330	175712752	0.03254	NA	NA	NA	NA	NA	NA	17	0	top1	0.023	9.30E-39	NA	NA
SeqId_13942_140	SPSB1	1	9352953	9429591	0.00872	NA	NA	NA	NA	NA	NA	42	0	enet	0.00041	0.048	NA	NA
SeqId_15521_4	CLSTN1	1	9788984	9884042	0.21041	NA	NA	NA	NA	NA	NA	29	0	enet	0.12	4.20E-199	NA	NA
SeqId_3152_57	TNFRSF1B	1	12227048	12269285	0.06344	NA	NA	NA	NA	NA	NA	50	0	enet	0.049	9.50E-82	NA	NA
SeqId_7192_37	IFNLR1	1	24480658	24514449	0.06664	NA	NA	NA	NA	NA	NA	58	0	enet	0.028	5.40E-47	NA	NA
SeqId_17210_2	TCL1A	14	96176284	96180533	0.0318	NA	NA	NA	NA	NA	NA	21	0	enet	0.025	8.90E-42	NA	NA
SeqId_7211_2	RNASE1	14	21269387	21271014	0.20067	NA	NA	NA	NA	NA	NA	45	0	enet	0.14	3.00E-247	NA	NA
SeqId_8394_56	RNASE2	14	21423646	21424595	0.04224	NA	NA	NA	NA	NA	NA	34	0	top1	0.046	1.50E-76	NA	NA
SeqId_15535_3	PRSS27	16	2762420	2770552	0.00679	NA	NA	NA	NA	NA	NA	7	0	top1	0.005	9.80E-10	NA	NA
SeqId_18893_26	GPR56	16	57644564	57699492	0.01706	NA	NA	NA	NA	NA	NA	46	0	enet	0.014	7.40E-24	NA	NA
SeqId_19129_15	MTHFSD	16	86563784	86588841	0.06384	NA	NA	NA	NA	NA	NA	19	0	enet	0.099	1.90E-165	NA	NA
SeqId_2827_23	CX3CL1	16	57406389	57418956	0.02161	NA	NA	NA	NA	NA	NA	18	0	enet	0.03	4.00E-49	NA	NA
SeqId_5691_2	CRISPLD2	16	84853591	84954374	0.02526	NA	NA	NA	NA	NA	NA	31	0	enet	0.015	5.50E-26	NA	NA
SeqId_11152_46	KLK13	19	51558883	51568371	0.12041	NA	NA	NA	NA	NA	NA	71	0	enet	0.095	9.70E-159	NA	NA
SeqId_15631_18	PSG1	19	43370616	43383974	0.01395	NA	NA	NA	NA	NA	NA	29	0	enet	0.0076	7.10E-14	NA	NA
SeqId_2831_29	KLK11	19	51525472	51531295	0.26011	NA	NA	NA	NA	NA	NA	35	0	enet	0.18	6.90E-308	NA	NA
SeqId_2966_65	CLEC11A	19	51226621	51228975	0.10631	NA	NA	NA	NA	NA	NA	43	0	enet	0.086	5.00E-143	NA	NA
SeqId_4500_50	CLEC11A	19	51226621	51228975	0.11178	NA	NA	NA	NA	NA	NA	36	0	enet	0.09	8.00E-151	NA	NA
SeqId_5649_83	PSG4	19	43696854	43711451	0.24123	NA	NA	NA	NA	NA	NA	127	0	enet	0.27	0	NA	NA
SeqId_5651_50	PSG7	19	43428284	43441359	0.01621	NA	NA	NA	NA	NA	NA	9	0	enet	0.014	7.60E-24	NA	NA
SeqId_5963_9	DMKN	19	35988122	36004560	0.01341	NA	NA	NA	NA	NA	NA	12	0	enet	0.011	6.30E-19	NA	NA
SeqId_6049_64	PTPRS	19	5158506	5340814	0.01132	NA	NA	NA	NA	NA	NA	44	0	enet	0.003	2.10E-06	NA	NA
SeqId_6398_12	LONP1	19	5691845	5720583	0.00608	NA	NA	NA	NA	NA	NA	17	0	top1	0.00011	0.18	NA	NA
SeqId_6444_15	PSG3	19	43225790	43244633	0.26457	NA	NA	NA	NA	NA	NA	108	0	enet	0.33	0	NA	NA
SeqId_9314_9	PSG5	19	43670408	43690688	0.17114	NA	NA	NA	NA	NA	NA	115	0	enet	0.28	0	NA	NA
SeqId_9335_28	PSG9	19	43715943	43773682	0.11247	NA	NA	NA	NA	NA	NA	87	0	enet	0.086	3.20E-143	NA	NA
SeqId_9794_17	ADCK4	19	41197419	41231689	0.0043	NA	NA	NA	NA	NA	NA	7	0	enet	0.00083	0.0081	NA	NA
SeqId_13116_25	CD177	19	43857838	43867324	0.66904	NA	NA	NA	NA	NA	NA	267	0	enet	0.38	0	NA	NA
SeqId_5095_21	KIR2DL4	19	55314990	55325972	0.27266	NA	NA	NA	NA	NA	NA	7	0	enet	0.29	0	NA	NA
SeqId_10908_2	GALNT13	2	154728435	155310491	0.01176	NA	NA	NA	NA	NA	NA	7	0	top1	0.0042	2.00E-08	NA	NA
SeqId_13686_2	IL5RA	3	3108008	3168297	0.33274	NA	NA	NA	NA	NA	NA	85	0	enet	0.2	0	NA	NA
SeqId_10833_64	HHIP	4	145567308	145666423	0.0287	NA	NA	NA	NA	NA	NA	21	0	enet	0.016	1.00E-26	NA	NA
SeqId_13959_7	LAP3	4	17578821	17609595	0.01379	NA	NA	NA	NA	NA	NA	18	0	enet	0.013	4.30E-23	NA	NA
SeqId_4867_15	VEGFA	6	43737921	43754224	0.01823	NA	NA	NA	NA	NA	NA	13	0	top1	0.014	6.20E-24	NA	NA
SeqId_3049_61	PRSS1	7	142457319	142460927	0.01511	NA	NA	NA	NA	NA	NA	13	0	top1	0.0082	7.00E-15	NA	NA
SeqId_10339_48	ENO2	12	7022909	7032861	0.02024	NA	NA	NA	NA	NA	NA	11	0	enet	0.015	9.40E-26	NA	NA
SeqId_15482_12	A2ML1	12	8975217	9039597	0.04291	NA	NA	NA	NA	NA	NA	NA	0	enet	0.028	2.50E-46	NA	NA
SeqId_19241_31	RBP5	12	7268332	7281485	0.10834	NA	NA	NA	NA	NA	NA	3	0	enet	0.09	8.10E-150	NA	NA
SeqId_3204_2	LTA4H	12	96394531	96437298	0.01992	NA	NA	NA	NA	NA	NA	45	0	enet	0.013	2.50E-22	NA	NA
SeqId_6580_29	PZP	12	9301436	9360991	0.20676	NA	NA	NA	NA	NA	NA	NA	0	enet	0.19	0	NA	NA
SeqId_7173_141	TMEM132C	12	128751715	129192456	0.06958	NA	NA	NA	NA	NA	NA	77	0	enet	0.071	5.10E-118	NA	NA

SeqId_9094_5	CLEC4C	12	7881979	7904201	0.10691	NA	NA	NA	NA	NA	NA	NA	0	enet	0.18	1.1e-317	NA	NA
SeqId_12990_39	MAP3K3	17	61699775	61773665	0.00302	NA	NA	NA	NA	NA	NA	4	0	top1	0.00062	0.019	NA	NA
SeqId_4278_14	P4HB	17	79801035	79818732	0.02845	NA	NA	NA	NA	NA	NA	6	0	top1	0.019	2.10E-32	NA	NA
SeqId_4990_87	GP1BA	17	4835572	4838318	0.02626	NA	NA	NA	NA	NA	NA	22	0	enet	0.022	3.40E-37	NA	NA
SeqId_7947_19	ENTHD2	17	79202077	79212891	0.0054	NA	NA	NA	NA	NA	NA	23	0	enet	0.0014	0.00079	NA	NA
SeqId_8252_2	NOTUM	17	79910383	79919716	0.01281	NA	NA	NA	NA	NA	NA	18	0	enet	0.0089	6.10E-16	NA	NA
SeqId_8268_98	HS3ST3A1	17	13397349	13505246	0.01016	NA	NA	NA	NA	NA	NA	41	0	enet	0.0067	1.80E-12	NA	NA
SeqId_9876_20	ALDOC	17	26900133	26903966	0.02635	NA	NA	NA	NA	NA	NA	23	0	top1	0.014	2.00E-23	NA	NA
SeqId_8356_88	OXT	20	3052266	3053163	0.33125	NA	NA	NA	NA	NA	NA	65	0	enet	0.15	1.50E-250	NA	NA
SeqId_7935_26	LARGE	22	33558212	34318829	0.00527	NA	NA	NA	NA	NA	NA	10	0	top1	2.00E-04	0.12	NA	NA
SeqId_9343_16	IL2RB	22	37521883	37571094	0.01255	NA	NA	NA	NA	NA	NA	20	0	top1	0.011	4.40E-20	NA	NA
SeqId_13947_371	NUDT12	5	102884556	102898491	0.03735	NA	NA	NA	NA	NA	NA	26	0	top1	0.026	2.30E-43	NA	NA
SeqId_19518_12	HK3	5	176307870	176326347	0.01356	NA	NA	NA	NA	NA	NA	19	0	enet	0.013	5.20E-23	NA	NA
SeqId_7251_64	C1QTNF3	5	34017963	34043318	0.03208	NA	NA	NA	NA	NA	NA	63	0	enet	0.017	3.50E-28	NA	NA
SeqId_10620_21	MSMB	10	51562515	51547642	0.61829	NA	NA	NA	NA	NA	NA	196	0	enet	0.31	0	NA	NA
SeqId_2637_77	MRC1	10	17851347	18200093	0.20436	NA	NA	NA	NA	NA	NA	44	0	enet	0.23	0	NA	NA
SeqId_2828_82	SPINT1	15	41136216	41150405	0.05838	NA	NA	NA	NA	NA	NA	20	0	enet	0.028	8.80E-47	NA	NA
SeqId_4232_19	IGF1R	15	99191768	99507759	0.06153	NA	NA	NA	NA	NA	NA	58	0	enet	0.028	8.40E-47	NA	NA
SeqId_15298_199	NETO1	18	70409549	70535381	0.00596	NA	NA	NA	NA	NA	NA	4	0	enet	0.0014	8.00E-04	NA	NA
SeqId_5457_5	COLEC12	18	316737	500722	0.08884	NA	NA	NA	NA	NA	NA	115	0	enet	0.046	1.10E-76	NA	NA
SeqId_4157_2	F2	11	46740746	46761056	0.01965	NA	NA	NA	NA	NA	NA	NA	0	top1	0.0086	1.60E-15	NA	NA
SeqId_19631_13	KNG1	3	186435137	186462199	0.02637	NA	NA	NA	NA	NA	NA	NA	0	enet	0.025	4.60E-41	NA	NA

Supplementary Table 2 MR results of plasma pQTL and VTE GWAS.

Protein	IVs			MR-IVW								MR-Egger								MR-Egger-intercept							
**protein**	**num_SNP**	**F**	**PVE**	**beta**	**se**	**CILower**	**CIUpper**	**OR**	**ORLower**	**ORUpper**	**pval**	**beta**	**se**	**CILower**	**CIUpper**	**OR**	**ORLower**	**ORUpper**	**pval**	**beta**	**se**	**CILower**	**CIUpper**	**OR**	**ORLower**	**ORUpper**	**pval**
F2	6	47.4738	0.0380	0.5108	0.0439	0.4248	0.5968	1.6666	1.5292	1.8162	2.54E-31	0.5713	0.0934	0.3882	0.7543	1.7705	1.4743	2.1262	9.59E-10	-0.0139	0.0187	-0.0505	0.0227	0.9862	0.9507	1.0230	4.57E-01
F11	21	108.8984	0.2413	0.3566	0.0303	0.2972	0.4159	1.4284	1.3461	1.5158	5.42E-32	0.3856	0.0561	0.2756	0.4955	1.4704	1.3173	1.6414	6.31E-12	-0.0081	0.0131	-0.0337	0.0175	0.9920	0.9669	1.0177	5.37E-01
ABO	32	302.8645	0.5744	0.2482	0.0362	0.1773	0.3190	1.2817	1.1940	1.3758	6.83E-12	0.2519	0.0647	0.1250	0.3788	1.2865	1.1332	1.4606	9.97E-05	-0.0013	0.0185	-0.0376	0.0350	0.9987	0.9631	1.0356	9.44E-01
PLCG2	13	50.3990	0.0834	0.1992	0.0341	0.1323	0.2661	1.2205	1.1415	1.3049	5.31E-09	0.1623	0.0828	0.0000	0.3247	1.1763	1.0000	1.3836	5.00E-02	0.0064	0.0131	-0.0192	0.0320	1.0065	0.9810	1.0326	6.22E-01
LRP4	15	121.8032	0.2025	0.1520	0.0278	0.0975	0.2065	1.1642	1.1024	1.2294	4.56E-08	0.2292	0.0931	0.0466	0.4117	1.2576	1.0477	1.5094	1.39E-02	-0.0273	0.0314	-0.0887	0.0342	0.9731	0.9151	1.0348	3.85E-01
PLEK	23	92.9259	0.2292	-0.1258	0.0180	-0.1610	-0.0906	0.8818	0.8513	0.9134	2.61E-12	-0.1420	0.0361	-0.2127	-0.0712	0.8677	0.8084	0.9313	8.36E-05	0.0043	0.0083	-0.0120	0.0205	1.0043	0.9881	1.0207	6.06E-01
KLKB1	19	76.8540	0.1687	0.1432	0.0731	-0.0002	0.2865	1.1539	0.9998	1.3318	5.03E-02	0.2061	0.1332	-0.0551	0.4672	1.2289	0.9464	1.5956	1.22E-01	-0.0157	0.0276	-0.0697	0.0383	0.9844	0.9327	1.0391	5.69E-01
PROC	9	42.1744	0.0501	-0.2112	0.0568	-0.3225	-0.0999	0.8096	0.7244	0.9049	2.00E-04	-0.3330	0.1293	-0.5865	-0.0795	0.7167	0.5562	0.9235	1.00E-02	0.0196	0.0187	-0.0171	0.0564	1.0198	0.9830	1.0580	2.95E-01
KNG1	13	91.8159	0.1422	0.1139	0.0223	0.0701	0.1577	1.1207	1.0726	1.1709	3.44E-07	0.0886	0.0456	-0.0007	0.1780	1.0927	0.9993	1.1948	5.19E-02	0.0074	0.0116	-0.0153	0.0301	1.0074	0.9848	1.0306	5.24E-01
THBS2	30	172.6856	0.4191	-0.0981	0.0137	-0.1249	-0.0714	0.9065	0.8826	0.9311	6.56E-13	-0.0653	0.0220	-0.1085	-0.0221	0.9368	0.8972	0.9782	3.06E-03	-0.0098	0.0052	-0.0201	0.0004	0.9902	0.9801	1.0004	6.04E-02
SERPINA1	38	272.5745	0.5908	-0.0666	0.0123	-0.0907	-0.0424	0.9356	0.9133	0.9585	6.60E-08	-0.0806	0.0197	-0.1192	-0.0420	0.9226	0.8876	0.9589	4.32E-05	0.0049	0.0054	-0.0056	0.0155	1.0050	0.9944	1.0157	3.60E-01
RARRES2	9	137.1850	0.1463	-0.0695	0.0290	-0.1264	-0.0127	0.9328	0.8813	0.9874	1.65E-02	-0.0970	0.0495	-0.1940	0.0001	0.9076	0.8236	1.0001	5.02E-02	0.0089	0.0127	-0.0161	0.0338	1.0089	0.9841	1.0344	4.86E-01
CEL	11	35.0914	0.0509	-0.2532	0.1471	-0.5414	0.0350	0.7763	0.5819	1.0357	8.51E-02	0.2330	0.2086	-0.1759	0.6418	1.2623	0.8387	1.9000	2.64E-01	-0.0830	0.0299	-0.1415	-0.0245	0.9204	0.8680	0.9758	5.44E-03
GP6	13	85.6452	0.1339	0.0528	0.0255	0.0028	0.1029	1.0543	1.0028	1.1084	3.86E-02	0.0412	0.0512	-0.0592	0.1415	1.0420	0.9425	1.1520	4.21E-01	0.0036	0.0136	-0.0230	0.0303	1.0036	0.9773	1.0307	7.89E-01
SERPINE2	44	420.9165	0.7210	-0.0461	0.0101	-0.0659	-0.0262	0.9550	0.9362	0.9741	5.24E-06	-0.0607	0.0162	-0.0925	-0.0288	0.9412	0.9117	0.9716	1.87E-04	0.0065	0.0057	-0.0046	0.0176	1.0065	0.9954	1.0178	2.51E-01
SERPINA10	31	163.4682	0.4137	-0.0579	0.0176	-0.0924	-0.0234	0.9437	0.9118	0.9768	9.92E-04	-0.0546	0.0257	-0.1050	-0.0041	0.9469	0.9003	0.9959	3.40E-02	-0.0013	0.0070	-0.0149	0.0124	0.9987	0.9852	1.0125	8.57E-01
OBP2B	7	93.0718	0.0829	-0.1566	0.1019	-0.3564	0.0432	0.8550	0.7002	1.0441	1.24E-01	0.2047	0.1079	-0.0068	0.4161	1.2271	0.9932	1.5161	5.78E-02	-0.1006	0.0258	-0.1511	-0.0501	0.9043	0.8598	0.9511	9.44E-05
EFEMP1	22	75.1299	0.1869	-0.1089	0.0199	-0.1479	-0.0700	0.8968	0.8625	0.9324	4.27E-08	-0.0893	0.0430	-0.1735	-0.0050	0.9146	0.8407	0.9950	3.78E-02	-0.0051	0.0098	-0.0244	0.0142	0.9949	0.9759	1.0143	6.06E-01
F5	11	47.5579	0.0677	0.1440	0.2328	-0.3123	0.6003	1.1549	0.7318	1.8227	5.36E-01	-0.1992	0.4175	-1.0176	0.6191	0.8194	0.3615	1.8573	6.33E-01	0.0733	0.0740	-0.0717	0.2183	1.0760	0.9308	1.2439	3.22E-01
MSR1	10	114.3170	0.1370	-0.0689	0.0241	-0.1162	-0.0216	0.9334	0.8903	0.9786	4.29E-03	-0.1094	0.0331	-0.1742	-0.0446	0.8964	0.8401	0.9564	9.39E-04	0.0144	0.0086	-0.0025	0.0313	1.0145	0.9975	1.0317	9.45E-02

Supplementary Table 3 Instrumental variables for 20 VTE significant proteins and annotations.

exposure	Outcome	SNP	CHR	BP	N	MAF	A1.exp	A2.out	BETA.exp	SE.exp	P.exp	BETA.out	SE.out	P.out
F2	VTE	rs113954134	11	47181131	7213	0.035214	A	G	0.2758	0.0448	7.56E-10	0.1035	0.0336	0.002031
F2	VTE	rs12796154	11	46810006	7213	0.063704	G	C	-0.1691	0.0342	7.73E-07	-0.0428	0.0266	0.10715
F2	VTE	rs183452254	11	46596504	7213	0.037571	T	C	0.1935	0.0432	7.73E-06	0.0860	0.0307	0.005109
F2	VTE	rs3136516	11	46739206	7213	0.467628	G	A	0.1525	0.0168	1.19E-19	0.0810	0.0122	2.78E-11
F2	VTE	rs78744550	11	46928050	7213	0.139609	C	T	0.0946	0.0239	7.43E-05	0.0486	0.0169	0.004043
F2	VTE	rs78807356	11	46575120	7213	0.026133	T	G	0.5055	0.0520	3.16E-22	0.2968	0.0321	2.24E-20
F11	VTE	rs114871367	4	186160567	7213	0.043047	C	A	0.1682	0.0411	4.26E-05	-0.0234	0.0338	0.489866
F11	VTE	rs115607076	4	186261041	7213	0.009982	A	G	0.3301	0.0835	7.74E-05	0.0382	0.0598	0.523137
F11	VTE	rs12331264	4	186312684	7213	0.322265	C	T	-0.0967	0.0178	5.22E-08	-0.0260	0.0129	0.044146
F11	VTE	rs181701858	4	186302485	7213	0.011091	C	T	-0.3202	0.0788	4.92E-05	0.0002	0.0625	0.997701
F11	VTE	rs2289252	4	186286227	7213	0.410786	T	C	0.4186	0.0163	1.66E-139	0.1551	0.0122	4.94E-37
F11	VTE	rs28697915	4	186259279	7213	0.339457	G	T	0.1092	0.0177	6.83E-10	0.0231	0.0471	0.624283
F11	VTE	rs35834666	4	186217021	7213	0.116526	C	T	-0.1417	0.0256	3.08E-08	-0.0475	0.0190	0.012217
F11	VTE	rs4253276	4	186242705	7213	0.04686	C	G	-0.3243	0.0394	2.10E-16	-0.0370	0.0277	0.181292
F11	VTE	rs4253406	4	186270238	7213	0.081104	T	G	0.2549	0.0303	4.53E-17	0.0615	0.0219	0.005053
F11	VTE	rs4253414	4	186275699	7213	0.027589	C	T	0.3067	0.0506	1.44E-09	0.1115	0.0334	0.000827
F11	VTE	rs4253421	4	186283783	7213	0.118467	A	G	-0.5102	0.0249	6.96E-91	-0.2103	0.0202	2.28E-25
F11	VTE	rs4456995	4	186349812	7213	0.170317	G	T	-0.1247	0.0220	1.56E-08	-0.0535	0.0164	0.001117
F11	VTE	rs6817039	4	186392223	7213	0.488146	A	G	0.0699	0.0167	3.01E-05	-0.0024	0.0121	0.842782
F11	VTE	rs6832067	4	186402766	7213	0.403716	C	T	-0.1402	0.0170	1.76E-16	-0.0329	0.0124	0.007877
F11	VTE	rs72646294	4	186209466	7213	0.018162	A	G	0.2722	0.0623	1.29E-05	0.1847	0.0434	2.08E-05
F11	VTE	rs72710593	4	186281549	7213	0.028213	C	T	0.2082	0.0503	3.53E-05	0.0705	0.0361	0.050856
F11	VTE	rs7667777	4	186204923	7213	0.417163	G	C	0.1457	0.0167	2.86E-18	0.0964	0.0122	3.09E-15
F11	VTE	rs7688348	4	186326085	7213	0.041592	G	A	-0.4098	0.0417	1.32E-22	-0.1775	0.0346	2.85E-07
F11	VTE	rs78134251	4	186342765	7213	0.03771	T	G	-0.2412	0.0434	2.78E-08	-0.0562	0.0321	0.080176
F11	VTE	rs80183441	4	186307395	7213	0.164356	C	A	-0.1040	0.0224	3.34E-06	-0.0694	0.0163	2.10E-05
F11	VTE	rs80229218	4	186104402	7213	0.111049	C	G	-0.1663	0.0265	3.87E-10	-0.0249	0.0194	0.200029
ABO	VTE	rs11244024	9	133183544	7213	0.015736	C	T	0.4371	0.0669	6.97E-11	0.0821	0.0483	0.089024
ABO	VTE	rs11244030	9	133200162	7213	0.254194	T	C	-0.1440	0.0189	2.654E-14	-0.0635	0.0141	6.77E-06
ABO	VTE	rs11523306	9	133511369	7213	0.168238	A	G	0.1133	0.0222	3.352E-07	0.0679	0.0159	1.86E-05

ABO	VTE	rs116842520	9	133078678	7213	0.033481	A	G	-0.2274	0.0461	8.295E-07	-0.0657	0.0349	0.059694
ABO	VTE	rs117119759	9	133345313	7213	0.020311	A	G	1.0161	0.0578	7.994E-68	0.2137	0.0348	8.12E-10
ABO	VTE	rs117405640	9	133015880	7213	0.026688	G	A	0.7591	0.0507	7.538E-50	0.0746	0.0512	0.145081
ABO	VTE	rs118070731	9	133109293	7213	0.020449	A	G	0.3947	0.0584	1.545E-11	-0.0334	0.0455	0.463013
ABO	VTE	rs140026638	9	133695394	7213	0.013517	A	G	0.3004	0.0717	2.85E-05	0.0218	0.0601	0.716379
ABO	VTE	rs147375567	9	133279293	7213	0.021974	G	A	-0.2982	0.0568	1.539E-07	-0.0828	0.0383	0.030528
ABO	VTE	rs2073926	9	133156163	7213	0.056357	T	A	0.1711	0.0358	1.847E-06	0.0903	0.0254	0.000383
ABO	VTE	rs2519150	9	133626050	7213	0.49494	C	T	0.0710	0.0166	1.828E-05	0.0387	0.0121	0.001394
ABO	VTE	rs3118667	9	133425943	7213	0.478788	C	T	0.1636	0.0166	9.378E-23	-0.0219	0.0121	0.07022
ABO	VTE	rs35410697	9	133424303	7213	0.075489	T	C	-0.1556	0.0316	8.785E-07	-0.0612	0.0245	0.012482
ABO	VTE	rs36221464	9	133443612	7213	0.021142	A	G	-0.2456	0.0576	2.054E-05	-0.1152	0.0434	0.007924
ABO	VTE	rs41297217	9	133473458	7213	0.106405	A	G	-0.1940	0.0269	5.59E-13	-0.0606	0.0206	0.003237
ABO	VTE	rs4962215	9	132780859	7213	0.355192	T	C	-0.0770	0.0173	9.072E-06	0.0104	0.0127	0.413224
ABO	VTE	rs549542	9	133126222	7213	0.206502	G	A	0.1933	0.0207	1.08E-20	0.0224	0.0151	0.136774
ABO	VTE	rs565126	9	133285059	7213	0.269375	A	G	-0.1923	0.0186	8.957E-25	-0.0074	0.0138	0.591567
ABO	VTE	rs586882	9	132981096	7213	0.089491	G	A	-0.1119	0.0287	9.985E-05	0.0085	0.0205	0.678729
ABO	VTE	rs598123	9	133137405	7213	0.060862	T	C	-0.1550	0.0346	7.547E-06	-0.0150	0.0256	0.558504
ABO	VTE	rs62575398	9	133603744	7213	0.045959	A	C	0.2509	0.0399	3.455E-10	0.0111	0.0309	0.7202
ABO	VTE	rs62576045	9	133522244	7213	0.232636	A	G	0.1122	0.0195	9.73E-09	-0.0573	0.0145	7.47E-05
ABO	VTE	rs630014	9	133274306	7213	0.46846	A	G	-0.4267	0.0160	2.54E-150	-0.1770	0.0122	1.17E-47
ABO	VTE	rs633862	9	133279871	7213	0.450853	C	T	-0.4178	0.0160	5.83E-143	-0.1718	0.0123	2.89E-44
ABO	VTE	rs671537	9	133337474	7213	0.046652	A	G	0.8434	0.0388	1.05E-101	-0.0597	0.0304	0.049571
ABO	VTE	rs68032997	9	133286683	7213	0.044295	C	T	-0.2992	0.0405	1.697E-13	-0.0698	0.0311	0.024863
ABO	VTE	rs685523	9	133445787	7213	0.109178	T	C	-0.2195	0.0268	2.758E-16	-0.0710	0.0200	0.000393
ABO	VTE	rs7022543	9	133167984	7213	0.345071	T	C	-0.1769	0.0175	6.02E-24	-0.0462	0.0127	0.000282
ABO	VTE	rs7023066	9	133215442	7213	0.188548	G	A	0.2630	0.0210	9.515E-36	0.0725	0.0157	4.05E-06
ABO	VTE	rs78711238	9	133341592	7213	0.015458	T	C	0.8278	0.0661	1.212E-35	-0.0570	0.0517	0.270145
ABO	VTE	rs8176690	9	133262914	7213	0.416401	G	A	-0.4005	0.0163	3.98E-128	-0.1554	0.0123	1.93E-36
ABO	VTE	rs8176739	9	133256136	7213	0.023083	A	G	-0.2306	0.0557	3.497E-05	-0.1156	0.0428	0.006897
PLCG2	VTE	rs1071644	16	81937798	7213	0.472411	T	C	-0.1654	0.0166	3.807E-23	-0.0468	0.0121	0.000113
PLCG2	VTE	rs117206377	16	81936864	7213	0.014211	C	G	-0.3707	0.0697	1.058E-07	-0.0802	0.0565	0.156138
PLCG2	VTE	rs11860765	16	81868266	7213	0.25357	G	A	-0.0947	0.0190	5.901E-07	-0.0397	0.0143	0.005539

PLCG2	VTE	rs11863412	16	81934048	7213	0.108069	A	C	-0.1088	0.0268	4.952E-05	0.0113	0.0198	0.569529
PLCG2	VTE	rs12928126	16	81949692	7213	0.043533	G	C	-0.1958	0.0406	1.425E-06	-0.0374	0.0297	0.207795
PLCG2	VTE	rs4133124	16	81885924	7213	0.457438	G	T	0.0914	0.0166	3.842E-08	0.0129	0.0122	0.289424
PLCG2	VTE	rs4254322	16	81779948	7213	0.237557	G	A	-0.1660	0.0194	1.532E-17	-0.0318	0.0144	0.027619
PLCG2	VTE	rs61749044	16	81786071	7213	0.014072	T	A	-0.2897	0.0700	3.576E-05	0.0279	0.0441	0.527273
PLCG2	VTE	rs62045706	16	81851810	7213	0.204353	A	G	0.2800	0.0202	5.913E-43	0.0582	0.0147	7.21E-05
PLCG2	VTE	rs7199605	16	81777873	7213	0.473659	C	T	-0.0693	0.0167	3.166E-05	-0.0085	0.0122	0.484905
PLCG2	VTE	rs79465773	16	81839989	7213	0.020103	T	C	-0.2566	0.0594	1.598E-05	0.0013	0.0555	0.981473
PLCG2	VTE	rs9926920	16	81825447	7213	0.300707	G	C	-0.1349	0.0181	1.103E-13	-0.0449	0.0134	0.000776
PLCG2	VTE	rs9933207	16	81997672	7213	0.015181	A	G	-0.2865	0.0676	2.269E-05	0.0262	0.0505	0.604314
LRP4	VTE	rs111426027	11	46868056	7213	0.01428	T	C	0.2712	0.0692	9.037E-05	0.0077	0.0534	0.884527
LRP4	VTE	rs113954134	11	47181131	7213	0.035214	A	G	0.3424	0.0447	2.088E-14	0.1035	0.0336	0.002031
LRP4	VTE	rs117426515	11	46685487	7213	0.03362	G	A	-0.2741	0.0461	2.835E-09	-0.1049	0.0391	0.007282
LRP4	VTE	rs12796154	11	46810006	7213	0.063704	G	C	-0.2088	0.0342	1.042E-09	-0.0428	0.0266	0.10715
LRP4	VTE	rs139226547	11	47028108	7213	0.063566	G	A	0.3766	0.0339	1.765E-28	0.0630	0.0244	0.009907
LRP4	VTE	rs141348433	11	46755515	7213	0.010814	C	A	0.3730	0.0808	3.971E-06	-0.0915	0.0596	0.124857
LRP4	VTE	rs146218162	11	46764143	7213	0.020727	G	A	-0.3867	0.0577	2.175E-11	-0.0713	0.0485	0.141215
LRP4	VTE	rs17790204	11	46947730	7213	0.13954	G	C	0.2443	0.0237	1.04E-24	0.0485	0.0169	0.004067
LRP4	VTE	rs182261904	11	46493016	7213	0.024331	C	T	0.2738	0.0535	3.176E-07	0.0566	0.0382	0.138438
LRP4	VTE	rs2306029	11	46871557	7213	0.450229	T	C	0.4596	0.0161	9.97E-171	0.0721	0.0122	3.1E-09
LRP4	VTE	rs55971594	11	46453413	7213	0.053099	A	G	-0.1961	0.0371	1.31E-07	-0.0283	0.0279	0.309841
LRP4	VTE	rs61897843	11	47165998	7213	0.088382	A	G	0.2902	0.0289	1.587E-23	0.0389	0.0217	0.072539
LRP4	VTE	rs72899681	11	47049494	7213	0.036601	G	T	0.2977	0.0441	1.552E-11	0.0870	0.0324	0.007191
LRP4	VTE	rs78612907	11	46410153	7213	0.090947	C	G	-0.1854	0.0285	8.308E-11	0.0453	0.0203	0.025921
LRP4	VTE	rs79187134	11	47324914	7213	0.023846	T	C	-0.3136	0.0543	7.975E-09	0.0125	0.0398	0.753976
PLEK	VTE	rs10172004	2	68724345	7213	0.181755	T	C	0.0839	0.0214	9.07E-05	-0.0202	0.0157	0.198703
PLEK	VTE	rs10496166	2	68836777	7213	0.152433	A	G	-0.1103	0.0231	1.76E-06	0.0033	0.0167	0.843208
PLEK	VTE	rs112848132	2	68389536	7213	0.011438	T	G	-0.3502	0.0786	8.55E-06	-0.0321	0.0695	0.644145
PLEK	VTE	rs113437182	2	68290417	7213	0.033759	C	T	0.1796	0.0458	8.81E-05	0.0033	0.0362	0.927172
PLEK	VTE	rs113919063	2	68646975	7213	0.026272	A	G	-0.3285	0.0522	3.24E-10	0.0343	0.0412	0.404334
PLEK	VTE	rs114095419	2	68223835	7213	0.016151	C	G	-0.2591	0.0662	9.19E-05	0.0524	0.0577	0.363959
PLEK	VTE	rs114352691	2	68541745	7213	0.021489	A	T	0.2821	0.0574	8.95E-07	-0.0039	0.0418	0.925874

PLEK	VTE	rs11675599	2	68480147	7213	0.12221	G	A	0.2119	0.0255	1.24E-16	-0.0150	0.0182	0.408445
PLEK	VTE	rs13013374	2	68555250	7213	0.34729	G	A	-0.1456	0.0174	7.87E-17	-0.0016	0.0127	0.899303
PLEK	VTE	rs138474395	2	68347900	7213	0.022806	A	G	0.3191	0.0556	9.85E-09	-0.0621	0.0426	0.14506
PLEK	VTE	rs139725172	2	68056046	7213	0.013171	C	T	-0.3335	0.0734	5.63E-06	0.0294	0.0607	0.628229
PLEK	VTE	rs1443653	2	68364308	7213	0.030431	C	G	-0.3768	0.0483	7.31E-15	0.0096	0.0348	0.782348
PLEK	VTE	rs149240121	2	68343627	7213	0.017261	G	A	0.2860	0.0628	5.40E-06	-0.0113	0.0602	0.851161
PLEK	VTE	rs17035364	2	68365364	7213	0.026965	T	C	-0.4313	0.0510	3.45E-17	0.0751	0.0360	0.036834
PLEK	VTE	rs1867312	2	68392849	7213	0.432414	C	A	-0.4435	0.0161	3.92E-159	0.0684	0.0122	1.89E-08
PLEK	VTE	rs34370937	2	68392731	7213	0.032372	G	A	-0.2615	0.0471	2.90E-08	0.0793	0.0339	0.019247
PLEK	VTE	rs35052538	2	68383980	7213	0.061209	G	A	0.1446	0.0345	2.79E-05	-0.0531	0.0255	0.037557
PLEK	VTE	rs60878442	2	68354983	7213	0.467836	G	A	0.1624	0.0164	5.88E-23	-0.0334	0.0122	0.006323
PLEK	VTE	rs62143885	2	68332470	7213	0.028005	C	G	0.2569	0.0498	2.58E-07	-0.0470	0.0371	0.205515
PLEK	VTE	rs6727322	2	68802638	7213	0.356093	T	C	0.0721	0.0174	3.33E-05	-0.0068	0.0127	0.590247
PLEK	VTE	rs72835038	2	68523546	7213	0.029599	A	G	0.2644	0.0487	5.85E-08	0.0097	0.0329	0.767367
PLEK	VTE	rs744636	2	68115898	7213	0.383821	C	A	0.1301	0.0170	2.44E-14	-0.0201	0.0125	0.108562
PLEK	VTE	rs7579928	2	68375843	7213	0.394843	C	G	0.2336	0.0170	2.90E-42	-0.0162	0.0124	0.191926
KLKB1	VTE	rs10866290	4	186193325	7213	0.328573	C	T	0.0931	0.0178	1.63E-07	0.0370	0.0127	0.003465
KLKB1	VTE	rs141373173	4	186106897	7213	0.037432	T	C	-0.2354	0.0437	7.33E-08	-0.0001	0.0337	0.997042
KLKB1	VTE	rs148208197	4	186418597	7213	0.019479	A	G	0.2423	0.0603	5.95E-05	0.0298	0.0425	0.482718
KLKB1	VTE	rs180978231	4	186319217	7213	0.013101	C	T	-0.3105	0.0736	2.48E-05	0.1382	0.0593	0.019848
KLKB1	VTE	rs2241818	4	186164791	7213	0.179953	G	C	0.1382	0.0218	2.23E-10	0.0348	0.0154	0.023903
KLKB1	VTE	rs34745240	4	186201178	7213	0.031402	A	G	0.3001	0.0477	3.40E-10	0.0422	0.0350	0.226915
KLKB1	VTE	rs34852777	4	186217908	7213	0.117011	C	T	-0.1864	0.0255	3.05E-13	-0.0473	0.0190	0.012781
KLKB1	VTE	rs4253304	4	186252417	7213	0.420006	C	G	0.3605	0.0163	3.53E-105	0.1200	0.0122	6.46E-23
KLKB1	VTE	rs4253331	4	186257981	7213	0.08249	C	T	-0.4820	0.0296	1.57E-58	0.0506	0.0219	0.020566
KLKB1	VTE	rs4253419	4	186278734	7213	0.17011	G	A	-0.1884	0.0222	2.58E-17	-0.0851	0.0159	8.67E-08
KLKB1	VTE	rs4862665	4	186214988	7213	0.115417	A	G	0.1178	0.0262	6.81E-06	-0.1823	0.0202	1.57E-19
KLKB1	VTE	rs55857110	4	186152916	7213	0.284764	C	G	-0.0769	0.0184	3.00E-05	-0.0033	0.0133	0.804822
KLKB1	VTE	rs5743305	4	186068179	7213	0.371829	A	T	-0.0883	0.0172	2.80E-07	0.0052	0.0125	0.676752
KLKB1	VTE	rs6849187	4	186125608	7213	0.489117	T	C	-0.0700	0.0166	2.68E-05	0.0089	0.0121	0.460131
KLKB1	VTE	rs72646269	4	186199265	7213	0.022945	A	G	0.3737	0.0556	1.87E-11	0.1360	0.0349	9.98E-05
KLKB1	VTE	rs72710569	4	186254192	7213	0.019964	G	T	-0.2969	0.0596	6.46E-07	0.1680	0.0443	0.000149

KLKB1	VTE	rs74541206	4	186072411	7213	0.025718	T	C	-0.2381	0.0522	5.14E-06	-0.0473	0.0393	0.228823
KLKB1	VTE	rs75470212	4	186130668	7213	0.071052	A	T	0.1620	0.0325	6.30E-07	-0.0105	0.0259	0.684035
KLKB1	VTE	rs8192550	4	186533818	7213	0.042839	C	T	-0.1580	0.0405	9.80E-05	0.0068	0.0293	0.816668
PROC	VTE	rs13385611	2	127247114	7213	0.075627	C	T	-0.1289	0.0315	4.47E-05	0.0113	0.0224	0.614465
PROC	VTE	rs1799809	2	127418299	7213	0.430473	G	A	-0.2312	0.0167	5.25E-43	0.0536	0.0122	1.13E-05
PROC	VTE	rs1820970	2	127615448	7213	0.161791	A	G	-0.1145	0.0224	3.24E-07	0.0498	0.0164	0.002362
PROC	VTE	rs2069921	2	127422262	7213	0.259115	G	A	0.1062	0.0190	2.20E-08	-0.0301	0.0139	0.029885
PROC	VTE	rs6722345	2	127150899	7213	0.385415	A	G	-0.0675	0.0172	8.56E-05	0.0010	0.0124	0.933164
PROC	VTE	rs72845944	2	127265947	7213	0.062249	T	C	-0.1821	0.0345	1.34E-07	0.0815	0.0240	0.000682
PROC	VTE	rs72848646	2	127407859	7213	0.049494	C	T	-0.1616	0.0384	2.56E-05	-0.0073	0.0288	0.80056
PROC	VTE	rs74392719	2	127418647	7213	0.010329	C	G	-0.3851	0.0826	3.22E-06	0.0267	0.0752	0.722535
PROC	VTE	rs7584040	2	127105648	7213	0.222515	T	C	0.0784	0.0201	9.41E-05	0.0215	0.0144	0.133437
KNG1	VTE	rs111711525	3	186706201	7213	0.05809	T	C	0.1803	0.0357	4.573E-07	0.0007	0.0268	0.977701
KNG1	VTE	rs115357776	3	186710844	7213	0.024054	C	T	-0.3526	0.0545	1.066E-10	-0.0874	0.0427	0.040602
KNG1	VTE	rs148892840	3	186736520	7213	0.025579	G	A	-0.2653	0.0523	4.012E-07	-0.0683	0.0417	0.101349
KNG1	VTE	rs17299991	3	186788141	7213	0.039997	T	C	-0.2444	0.0424	8.691E-09	-0.0141	0.0284	0.619173
KNG1	VTE	rs266735	3	186814716	7213	0.500901	A	C	-0.1277	0.0165	1.323E-14	-0.0236	0.0121	0.050718
KNG1	VTE	rs5029980	3	186720155	7213	0.114446	C	T	0.1452	0.0262	2.959E-08	0.0266	0.0188	0.156749
KNG1	VTE	rs5030062	3	186736391	7213	0.37682	C	A	0.3134	0.0166	2.418E-77	0.0431	0.0124	0.00053
KNG1	VTE	rs5030083	3	186741394	7213	0.032095	T	C	-0.2258	0.0473	1.852E-06	0.0077	0.0347	0.824482
KNG1	VTE	rs58005713	3	186748163	7213	0.094898	C	G	-0.3400	0.0279	9.421E-34	-0.0264	0.0213	0.216458
KNG1	VTE	rs71322413	3	186784065	7213	0.074865	T	C	0.1500	0.0319	2.528E-06	0.0115	0.0219	0.601038
KNG1	VTE	rs76438938	3	186743735	7213	0.027312	T	C	0.7631	0.0501	1.142E-51	0.0698	0.0339	0.039195
KNG1	VTE	rs76664993	3	186756895	7213	0.022321	G	C	0.2715	0.0558	1.18E-06	-0.0155	0.0428	0.716736
KNG1	VTE	rs76675228	3	186705548	7213	0.220851	A	G	-0.1306	0.0201	7.826E-11	-0.0193	0.0148	0.194156
THBS2	VTE	rs10945328	6	168903499	7213	0.325731	C	G	-0.0902	0.0177	3.686E-07	0.0173	0.0128	0.177939
THBS2	VTE	rs10945406	6	169238696	7213	0.053029	C	A	-0.2002	0.0367	4.978E-08	0.0455	0.0264	0.084769
THBS2	VTE	rs11759438	6	169233240	7213	0.491058	C	T	-0.2382	0.0166	4.546E-46	0.0546	0.0122	7.1E-06
THBS2	VTE	rs117698690	6	169044901	7213	0.014488	T	C	0.3916	0.0697	1.985E-08	-0.0343	0.0561	0.540531
THBS2	VTE	rs12174547	6	169224627	7213	0.197768	T	C	-0.3676	0.0202	2.146E-72	0.0165	0.0150	0.272434
THBS2	VTE	rs12180739	6	169214618	7213	0.434216	C	G	-0.1780	0.0166	1.676E-26	0.0198	0.0122	0.105469
THBS2	VTE	rs141319253	6	169659562	7213	0.020588	A	G	-0.2273	0.0578	8.438E-05	0.1019	0.0438	0.019907

THBS2	VTE	rs144629105	6	169163606	7213	0.017815	A	G	-0.2591	0.0627	3.619E-05	0.0029	0.0446	0.948987
THBS2	VTE	rs183293345	6	169184552	7213	0.011784	T	A	-0.4005	0.0761	1.451E-07	-0.0338	0.0572	0.55454
THBS2	VTE	rs2181284	6	169158857	7213	0.035699	T	C	-0.3977	0.0450	1.21E-18	0.0617	0.0335	0.065757
THBS2	VTE	rs28735654	6	169265609	7213	0.166089	C	T	-0.1253	0.0221	1.496E-08	0.0127	0.0162	0.431632
THBS2	VTE	rs35239983	6	168901700	7213	0.15479	A	G	0.1412	0.0231	1.022E-09	0.0003	0.0167	0.987606
THBS2	VTE	rs4346834	6	169130820	7213	0.15278	G	C	0.2770	0.0230	3.991E-33	-0.0251	0.0165	0.12786
THBS2	VTE	rs55995725	6	169256176	7213	0.082143	A	G	-0.1412	0.0302	2.933E-06	0.0113	0.0213	0.597191
THBS2	VTE	rs56003273	6	169250135	7213	0.425551	T	C	0.1984	0.0167	4.153E-32	-0.0193	0.0123	0.115325
THBS2	VTE	rs6900380	6	168837844	7213	0.338625	A	C	0.0780	0.0176	9.475E-06	-0.0105	0.0129	0.415498
THBS2	VTE	rs6914478	6	169227088	7213	0.408221	C	G	-0.1871	0.0167	6.13E-29	0.0048	0.0123	0.695282
THBS2	VTE	rs6921774	6	169255984	7213	0.422778	C	G	-0.1389	0.0166	6.606E-17	0.0125	0.0122	0.304598
THBS2	VTE	rs73038016	6	169144721	7213	0.018855	A	G	-0.3427	0.0612	2.216E-08	0.0750	0.0483	0.12086
THBS2	VTE	rs73043894	6	169240832	7213	0.062595	T	C	-0.1742	0.0342	3.604E-07	0.0432	0.0239	0.070911
THBS2	VTE	rs73238295	6	169226100	7213	0.051088	T	C	-0.2061	0.0378	5.043E-08	0.0192	0.0299	0.521782
THBS2	VTE	rs74507247	6	169224675	7213	0.098295	A	G	0.9451	0.0256	4.93E-274	-0.0590	0.0210	0.005003
THBS2	VTE	rs74920237	6	169334738	7213	0.068418	G	A	0.1416	0.0330	1.833E-05	-0.0184	0.0245	0.452314
THBS2	VTE	rs7753360	6	168998277	7213	0.411549	G	T	0.0777	0.0169	4.337E-06	0.0001	0.0121	0.990766
THBS2	VTE	rs78681488	6	169156028	7213	0.021489	A	G	0.5913	0.0574	1.091E-24	-0.0857	0.0408	0.035867
THBS2	VTE	rs9346611	6	169070944	7213	0.175794	T	C	-0.1666	0.0218	2.543E-14	0.0298	0.0159	0.060194
THBS2	VTE	rs9478030	6	169489802	7213	0.275613	C	T	-0.1157	0.0185	4.349E-10	0.0169	0.0135	0.212301
THBS2	VTE	rs9800437	6	169319631	7213	0.032164	T	C	0.3870	0.0469	1.856E-16	-0.0816	0.0359	0.023
THBS2	VTE	rs9800620	6	169314235	7213	0.407528	G	A	-0.0895	0.0168	1.085E-07	0.0362	0.0124	0.003515
THBS2	VTE	rs9800782	6	169241347	7213	0.019617	T	C	-0.2936	0.0590	6.686E-07	0.0178	0.0460	0.699059
SERPINA1	VTE	rs1016686	14	94372903	7213	0.225149	T	C	0.5164	0.0191	4.92E-154	-0.0208	0.0147	0.156638
SERPINA1	VTE	rs111397271	14	94611890	7213	0.011923	A	G	0.3401	0.0761	8.08E-06	0.0161	0.0547	0.768964
SERPINA1	VTE	rs11160184	14	94471654	7213	0.160682	T	C	-0.2078	0.0227	6.58E-20	0.0203	0.0169	0.229316
SERPINA1	VTE	rs112209366	14	94408296	7213	0.055733	G	A	0.5386	0.0357	1.17E-50	0.0150	0.0270	0.57895
SERPINA1	VTE	rs112452424	14	94074461	7213	0.08346	A	G	0.2355	0.0299	4.08E-15	-0.0120	0.0224	0.591431
SERPINA1	VTE	rs11621961	14	94303139	7213	0.369957	T	C	-0.3050	0.0168	2.29E-72	0.0423	0.0124	0.000653
SERPINA1	VTE	rs116862882	14	94500380	7213	0.035492	C	T	0.1880	0.0448	2.70E-05	-0.0322	0.0324	0.319531
SERPINA1	VTE	rs12880244	14	94295255	7213	0.146402	A	T	0.1602	0.0233	6.50E-12	-0.0306	0.0182	0.093019
SERPINA1	VTE	rs139421381	14	94459937	7213	0.023499	T	G	-0.7402	0.0543	7.16E-42	0.0953	0.0394	0.015551

SERPINA1	VTE	rs143038446	14	94363153	7213	0.015528	T	C	0.4131	0.0671	7.70E-10	-0.0938	0.0562	0.095215
SERPINA1	VTE	rs146066805	14	94694328	7213	0.025094	T	C	0.2421	0.0526	4.30E-06	0.0182	0.0394	0.643503
SERPINA1	VTE	rs17825650	14	94482062	7213	0.160128	T	C	-0.1598	0.0225	1.44E-12	-0.0080	0.0162	0.619417
SERPINA1	VTE	rs1956452	14	94154058	7213	0.338417	C	T	0.1662	0.0175	3.17E-21	-0.0318	0.0129	0.014096
SERPINA1	VTE	rs28373907	14	94108068	7213	0.149244	C	T	0.2110	0.0230	5.33E-20	-0.0065	0.0169	0.700292
SERPINA1	VTE	rs2854946	14	94582042	7213	0.217732	G	C	0.0909	0.0202	6.82E-06	0.0108	0.0145	0.458172
SERPINA1	VTE	rs28929474	14	94378610	7213	0.018786	T	C	-1.8065	0.0576	2.77E-202	0.1543	0.0397	0.000103
SERPINA1	VTE	rs2896291	14	94666318	7213	0.439484	G	A	0.0985	0.0168	4.31E-09	0.0014	0.0122	0.910732
SERPINA1	VTE	rs34025389	14	94343473	7213	0.035422	T	C	0.3704	0.0443	7.18E-17	-0.0404	0.0345	0.241626
SERPINA1	VTE	rs34097828	14	94321337	7213	0.240053	G	C	-0.2774	0.0191	5.72E-47	0.0212	0.0141	0.132952
SERPINA1	VTE	rs55704412	14	94282356	7213	0.032234	A	G	0.3069	0.0470	7.37E-11	-0.0042	0.0342	0.901589
SERPINA1	VTE	rs61049496	14	94516476	7213	0.242756	G	A	0.1266	0.0192	4.38E-11	0.0251	0.0141	0.075704
SERPINA1	VTE	rs61982442	14	93915936	7213	0.054138	G	T	0.2039	0.0369	3.42E-08	0.0104	0.0266	0.696413
SERPINA1	VTE	rs6575426	14	94392997	7213	0.039304	T	C	0.3135	0.0426	2.09E-13	0.0014	0.0329	0.964852
SERPINA1	VTE	rs709932	14	94382864	7213	0.154929	T	C	0.1540	0.0228	1.48E-11	0.0061	0.0167	0.717601
SERPINA1	VTE	rs7145310	14	94423969	7213	0.45619	C	A	-0.0806	0.0168	1.73E-06	-0.0073	0.0123	0.549234
SERPINA1	VTE	rs7145904	14	94086553	7213	0.089561	G	T	-0.2739	0.0289	3.36E-21	0.0016	0.0212	0.941481
SERPINA1	VTE	rs7156903	14	94000564	7213	0.374116	T	G	-0.1217	0.0171	1.16E-12	0.0048	0.0126	0.699812
SERPINA1	VTE	rs7158855	14	94560247	7213	0.255303	G	A	-0.1803	0.0191	3.85E-21	0.0136	0.0138	0.326206
SERPINA1	VTE	rs72702320	14	94101386	7213	0.101414	T	C	-0.1729	0.0275	3.37E-10	-0.0113	0.0204	0.579042
SERPINA1	VTE	rs76683768	14	94622606	7213	0.024747	C	A	-0.2775	0.0532	1.92E-07	0.0736	0.0344	0.032123
SERPINA1	VTE	rs79074413	14	93907762	7213	0.014488	C	T	-0.4533	0.0700	9.92E-11	0.0734	0.0460	0.110309
SERPINA1	VTE	rs79247904	14	94309697	7213	0.027797	A	G	0.3558	0.0508	2.70E-12	-0.0870	0.0362	0.016317
SERPINA1	VTE	rs80004767	14	94064607	7213	0.014211	A	G	-0.2714	0.0691	8.56E-05	-0.0136	0.0478	0.776138
SERPINA1	VTE	rs8004587	14	94521659	7213	0.207681	G	A	0.1111	0.0204	5.38E-08	0.0050	0.0147	0.731505
SERPINA1	VTE	rs8006225	14	94753320	7213	0.172744	T	G	-0.0998	0.0218	4.82E-06	0.0444	0.0156	0.004477
SERPINA1	VTE	rs8006968	14	94515198	7213	0.168169	A	T	0.1579	0.0221	9.87E-13	-0.0308	0.0165	0.062052
SERPINA1	VTE	rs8015166	14	94500833	7213	0.136906	C	T	0.1050	0.0239	1.13E-05	-0.0081	0.0177	0.646155
SERPINA1	VTE	rs965344	14	94351741	7213	0.207265	A	G	-0.4209	0.0198	2.19E-97	0.0119	0.0148	0.423803
RARRES2	VTE	rs10250758	7	150389942	7213	0.462082	A	C	-0.1489	0.0167	5.15E-19	0.0174	0.0122	0.153584
RARRES2	VTE	rs10263027	7	150422969	7213	0.481422	C	T	-0.1065	0.0167	1.89E-10	0.0018	0.0121	0.880597
RARRES2	VTE	rs111831496	7	150366610	7213	0.031887	C	T	-0.2085	0.0475	1.13E-05	0.0424	0.0329	0.197473

RARRES2	VTE	rs117067760	7	150339334	7213	0.026411	A	G	0.4366	0.0519	5.09E-17	0.0062	0.0353	0.859817
RARRES2	VTE	rs146643012	7	150344776	7213	0.01525	A	G	0.4776	0.0679	2.20E-12	-0.0177	0.0530	0.738888
RARRES2	VTE	rs189312654	7	150308510	7213	0.018023	T	C	0.4306	0.0617	3.29E-12	0.0944	0.0539	0.07991
RARRES2	VTE	rs3735167	7	150342466	7213	0.262928	T	C	0.5005	0.0182	1.02E-158	-0.0507	0.0140	0.000289
RARRES2	VTE	rs4725944	7	150779585	7213	0.411757	C	G	-0.0963	0.0168	1.13E-08	-0.0019	0.0124	0.875886
RARRES2	VTE	rs7809489	7	150402117	7213	0.074588	A	G	-0.1254	0.0317	7.67E-05	-0.0361	0.0240	0.132321
CEL	VTE	rs11244014	9	133165097	7213	0.023707	A	G	0.2847	0.0546	1.92E-07	0.0897	0.0388	0.02083
CEL	VTE	rs117731944	9	133168659	7213	0.016567	C	A	0.3148	0.0651	1.36E-06	-0.0714	0.0485	0.140771
CEL	VTE	rs1179011	9	133319548	7213	0.369402	G	A	-0.0787	0.0171	4.17E-06	0.0901	0.0126	8.71E-13
CEL	VTE	rs2075733	9	133072397	7213	0.040968	G	A	0.4428	0.0416	2.90E-26	-0.0177	0.0319	0.580093
CEL	VTE	rs2097646	9	132997916	7213	0.341051	G	A	0.0911	0.0176	2.19E-07	-0.0001	0.0129	0.99343
CEL	VTE	rs4246170	9	133237026	7213	0.087273	G	A	-0.1544	0.0295	1.76E-07	0.1443	0.0209	5.43E-12
CEL	VTE	rs45487303	9	133107106	7213	0.024331	T	C	0.2982	0.0541	3.71E-08	0.0065	0.0400	0.871382
CEL	VTE	rs656105	9	133301911	7213	0.474213	T	C	-0.0715	0.0166	1.75E-05	0.0910	0.0122	9.97E-14
CEL	VTE	rs667990	9	133109267	7213	0.471232	A	G	0.1130	0.0169	2.16E-11	-0.0387	0.0122	0.001448
CEL	VTE	rs72779222	9	133482710	7213	0.057188	C	T	0.1492	0.0353	2.40E-05	-0.0780	0.0259	0.002601
CEL	VTE	rs8176739	9	133256136	7213	0.023083	A	G	-0.2374	0.0557	2.03E-05	-0.1156	0.0428	0.006897
GP6	VTE	rs116850181	19	55031655	7213	0.021836	A	G	0.2419	0.0572	2.344E-05	-0.0432	0.0487	0.375137
GP6	VTE	rs11881907	19	55007769	7213	0.113961	T	A	0.4303	0.0258	3.292E-61	0.0454	0.0185	0.014336
GP6	VTE	rs12104158	19	55161599	7213	0.104256	T	G	-0.1280	0.0273	2.875E-06	0.0055	0.0197	0.78037
GP6	VTE	rs143108326	19	55088077	7213	0.01428	C	G	0.3137	0.0706	8.92E-06	0.0758	0.0549	0.166983
GP6	VTE	rs145342516	19	55090712	7213	0.02953	A	G	-0.3466	0.0495	2.799E-12	0.0168	0.0336	0.616244
GP6	VTE	rs1671168	19	55076638	7213	0.471579	C	G	-0.1354	0.0167	5.895E-16	-0.0086	0.0122	0.480759
GP6	VTE	rs189534942	19	55032557	7213	0.010675	A	G	0.6902	0.0805	1.202E-17	0.0937	0.0618	0.129552
GP6	VTE	rs4029	19	55044477	7213	0.13344	G	A	0.2010	0.0243	1.38E-16	0.0206	0.0177	0.243093
GP6	VTE	rs703466	19	54973075	7213	0.2831	T	C	0.1766	0.0182	4.865E-22	0.0220	0.0134	0.101466
GP6	VTE	rs703473	19	54947448	7213	0.046999	A	G	0.2638	0.0393	2.131E-11	0.0388	0.0281	0.16632
GP6	VTE	rs73064799	19	55150064	7213	0.070359	T	G	0.1579	0.0325	1.229E-06	-0.0297	0.0236	0.208841
GP6	VTE	rs79287667	19	55025316	7213	0.013656	T	G	-0.7798	0.0716	2.212E-27	0.0141	0.0478	0.767883
GP6	VTE	rs8104288	19	55023305	7213	0.020519	A	G	0.5957	0.0583	2.441E-24	-0.0356	0.0415	0.390889
SERPINE2	VTE	rs112044375	2	223712660	7213	0.010814	T	C	-0.3283	0.0803	4.4E-05	0.0453	0.0585	0.438736
SERPINE2	VTE	rs112197117	2	223789049	7213	0.032441	T	C	0.2385	0.0470	3.886E-07	-0.0195	0.0335	0.561167

SERPINE2	VTE	rs113685646	2	223824976	7213	0.023846	A	G	-0.3921	0.0547	8.434E-13	0.0139	0.0427	0.74492
SERPINE2	VTE	rs114211301	2	223978047	7213	0.044988	G	T	-0.1765	0.0400	1.043E-05	0.0115	0.0323	0.722776
SERPINE2	VTE	rs116256765	2	224013743	7213	0.017954	A	G	0.3814	0.0629	1.369E-09	0.0308	0.0461	0.504071
SERPINE2	VTE	rs116502077	2	223930912	7213	0.030362	G	A	-0.4952	0.0479	7.028E-25	-0.0116	0.0359	0.747454
SERPINE2	VTE	rs116598593	2	224031445	7213	0.014696	C	T	-0.6053	0.0687	1.464E-18	-0.0406	0.0471	0.388647
SERPINE2	VTE	rs11689646	2	223978048	7213	0.38188	T	G	0.1571	0.0171	5.723E-20	0.0043	0.0125	0.729234
SERPINE2	VTE	rs12478391	2	224020573	7213	0.023083	A	C	-0.7640	0.0550	2.736E-43	0.0734	0.0372	0.048342
SERPINE2	VTE	rs12998332	2	224434849	7213	0.173645	A	G	-0.0984	0.0221	8.239E-06	0.0048	0.0162	0.768582
SERPINE2	VTE	rs13412535	2	224010157	7213	0.234784	A	G	-0.9268	0.0162	3.662e-587	0.0545	0.0142	0.000118
SERPINE2	VTE	rs139935662	2	223731399	7213	0.028352	G	T	-0.4047	0.0496	4.23E-16	0.0204	0.0378	0.588937
SERPINE2	VTE	rs140405039	2	224018827	7213	0.025995	T	C	-0.7067	0.0521	1.874E-41	0.0879	0.0450	0.050921
SERPINE2	VTE	rs142948546	2	223943779	7213	0.010398	C	A	0.4624	0.0818	1.608E-08	-0.0016	0.0737	0.983161
SERPINE2	VTE	rs143958932	2	224183342	7213	0.010814	G	A	0.4226	0.0797	1.191E-07	0.0266	0.0674	0.693219
SERPINE2	VTE	rs1439932	2	223501176	7213	0.252322	C	T	-0.0969	0.0191	4.243E-07	-0.0063	0.0142	0.654663
SERPINE2	VTE	rs1453661	2	224080761	7213	0.050395	G	C	0.1837	0.0380	1.329E-06	0.0015	0.0278	0.958138
SERPINE2	VTE	rs145554607	2	223966154	7213	0.036323	T	C	0.1836	0.0446	3.856E-05	0.0021	0.0331	0.95045
SERPINE2	VTE	rs147808525	2	224101292	7213	0.01227	T	C	0.4264	0.0751	1.39E-08	0.1252	0.0547	0.022173
SERPINE2	VTE	rs147859711	2	223563032	7213	0.011368	T	A	-0.3104	0.0789	8.398E-05	-0.0389	0.0624	0.533108
SERPINE2	VTE	rs148629664	2	223721246	7213	0.017053	A	G	-0.2581	0.0645	6.376E-05	0.0559	0.0502	0.264816
SERPINE2	VTE	rs151126513	2	223496032	7213	0.03154	G	A	-0.1996	0.0473	2.437E-05	0.0652	0.0380	0.086268
SERPINE2	VTE	rs1530018	2	224060404	7213	0.226189	G	A	0.4158	0.0192	7.11E-101	-0.0222	0.0144	0.122598
SERPINE2	VTE	rs16865181	2	223769568	7213	0.01733	C	G	0.2532	0.0632	6.303E-05	0.0343	0.0478	0.472995
SERPINE2	VTE	rs16865990	2	224459365	7213	0.011507	G	A	-0.3444	0.0774	8.864E-06	0.0749	0.0650	0.249199
SERPINE2	VTE	rs17196910	2	224025763	7213	0.031956	T	C	0.3116	0.0471	4.017E-11	0.0192	0.0310	0.537066
SERPINE2	VTE	rs187436386	2	223779796	7213	0.010814	T	C	-0.3894	0.0808	1.47E-06	0.1122	0.0614	0.067927
SERPINE2	VTE	rs192260819	2	223911711	7213	0.016082	G	C	0.2796	0.0652	1.821E-05	-0.0135	0.0510	0.790546
SERPINE2	VTE	rs2543659	2	224390297	7213	0.204561	C	T	0.0910	0.0207	1.132E-05	-0.0148	0.0150	0.322569
SERPINE2	VTE	rs282252	2	224067522	7213	0.018716	T	C	-0.3832	0.0611	3.886E-10	-0.0186	0.0505	0.712801
SERPINE2	VTE	rs2894512	2	223701170	7213	0.012547	A	T	-0.2971	0.0739	5.916E-05	0.0461	0.0587	0.432393
SERPINE2	VTE	rs34439826	2	224164071	7213	0.121586	A	C	0.1889	0.0253	8.873E-14	0.0095	0.0181	0.598097
SERPINE2	VTE	rs35990446	2	223947729	7213	0.135519	A	G	-0.2143	0.0241	7.441E-19	-0.0040	0.0174	0.818463
SERPINE2	VTE	rs4674826	2	223836906	7213	0.101137	T	C	-0.1896	0.0273	4.48E-12	0.0416	0.0212	0.049543

SERPINE2	VTE	rs4674836	2	223990794	7213	0.377651	G	A	-0.3705	0.0166	3.25E-107	0.0071	0.0126	0.572909
SERPINE2	VTE	rs62185104	2	223781461	7213	0.052197	C	T	0.1635	0.0369	9.792E-06	-0.0083	0.0267	0.755219
SERPINE2	VTE	rs6714137	2	223863261	7213	0.140995	A	G	0.2670	0.0236	2.37E-29	-0.0190	0.0173	0.272077
SERPINE2	VTE	rs6748928	2	224412289	7213	0.19347	G	C	0.0876	0.0212	3.539E-05	0.0073	0.0150	0.627565
SERPINE2	VTE	rs74807729	2	224031068	7213	0.018023	A	G	0.2827	0.0623	5.828E-06	-0.0521	0.0499	0.296281
SERPINE2	VTE	rs755018	2	223740623	7213	0.481214	C	T	-0.0841	0.0166	4.095E-07	0.0017	0.0121	0.890795
SERPINE2	VTE	rs7584132	2	224007581	7213	0.165742	G	A	0.4194	0.0219	9.545E-80	-0.0218	0.0163	0.180367
SERPINE2	VTE	rs7588220	2	224008887	7213	0.023361	A	G	0.2491	0.0554	6.902E-06	0.0080	0.0373	0.830452
SERPINE2	VTE	rs77033874	2	224170096	7213	0.019479	T	C	-0.2997	0.0594	4.669E-07	-0.0073	0.0495	0.882358
SERPINE2	VTE	rs79074603	2	224220878	7213	0.037987	A	G	-0.1776	0.0436	4.687E-05	0.0102	0.0299	0.733957
SERPINA10	VTE	rs10873448	14	94164034	7213	0.168654	T	C	0.8074	0.0200	8.73673e-322	-0.0463	0.0166	0.005337
SERPINA10	VTE	rs117186178	14	94413966	7213	0.022321	T	C	-0.3615	0.0558	9.612E-11	-0.0273	0.0430	0.525244
SERPINA10	VTE	rs117635679	14	94159715	7213	0.020519	C	T	0.8425	0.0579	2.478E-47	0.0256	0.0447	0.566777
SERPINA10	VTE	rs12587447	14	94300389	7213	0.03459	C	T	-0.1937	0.0454	1.984E-05	-0.0503	0.0353	0.153758
SERPINA10	VTE	rs12880427	14	94107161	7213	0.138847	T	C	-0.1283	0.0237	6.449E-08	-0.0068	0.0175	0.69641
SERPINA10	VTE	rs12886767	14	94173587	7213	0.240191	G	C	-0.1483	0.0193	1.617E-14	-0.0070	0.0145	0.629782
SERPINA10	VTE	rs12893359	14	94446806	7213	0.427769	A	G	-0.0711	0.0168	2.237E-05	-0.0030	0.0123	0.809766
SERPINA10	VTE	rs151207481	14	94460643	7213	0.015874	A	G	0.3375	0.0661	3.428E-07	-0.0439	0.0567	0.438976
SERPINA10	VTE	rs17090693	14	94374994	7213	0.243241	C	T	-0.1261	0.0192	5.82E-11	0.0174	0.0140	0.21313
SERPINA10	VTE	rs17129523	14	94250303	7213	0.020311	A	G	0.7909	0.0587	6.624E-41	-0.0856	0.0438	0.05069
SERPINA10	VTE	rs1951022	14	94509410	7213	0.154443	T	C	-0.1160	0.0228	3.567E-07	0.0460	0.0165	0.005187
SERPINA10	VTE	rs1956178	14	94320207	7213	0.17919	A	C	0.1391	0.0214	9.124E-11	-0.0102	0.0160	0.523007
SERPINA10	VTE	rs2232710	14	94284149	7213	0.011854	C	T	0.5787	0.0757	2.411E-14	0.0825	0.0550	0.13359
SERPINA10	VTE	rs2402403	14	94066833	7213	0.01733	C	A	-0.3188	0.0637	5.773E-07	0.0261	0.0508	0.607696
SERPINA10	VTE	rs34520961	14	94176080	7213	0.075558	G	A	-0.1601	0.0314	3.658E-07	0.0166	0.0219	0.44702
SERPINA10	VTE	rs45505795	14	94290606	7213	0.030293	C	G	-0.2793	0.0488	1.06E-08	0.1237	0.0353	0.000461
SERPINA10	VTE	rs4900218	14	94119515	7213	0.046028	T	C	0.2210	0.0396	2.452E-08	-0.0500	0.0290	0.083959
SERPINA10	VTE	rs56280987	14	94288969	7213	0.017122	T	C	0.7831	0.0638	2.738E-34	-0.0708	0.0491	0.149173
SERPINA10	VTE	rs67412635	14	94360148	7213	0.045127	C	T	-0.2144	0.0395	5.851E-08	0.0232	0.0296	0.434013
SERPINA10	VTE	rs67922411	14	94303570	7213	0.204423	G	A	0.1461	0.0205	1.172E-12	0.0023	0.0149	0.87996
SERPINA10	VTE	rs7141881	14	94117527	7213	0.393526	A	G	0.1319	0.0170	9.149E-15	-0.0027	0.0125	0.828495
SERPINA10	VTE	rs743206	14	94413325	7213	0.349716	A	G	0.0968	0.0176	3.919E-08	0.0068	0.0129	0.600487

SERPINA10	VTE	rs74343336	14	94191609	7213	0.012616	C	T	0.7658	0.0737	4E-25	-0.1723	0.0533	0.001226
SERPINA10	VTE	rs74393562	14	94408286	7213	0.019825	C	T	0.4019	0.0595	1.527E-11	0.0365	0.0470	0.437512
SERPINA10	VTE	rs75199535	14	94341457	7213	0.054138	A	G	0.4377	0.0365	9.083E-33	0.0133	0.0272	0.625789
SERPINA10	VTE	rs75255245	14	94339720	7213	0.069527	A	G	0.1620	0.0330	9.193E-07	-0.0550	0.0247	0.026214
SERPINA10	VTE	rs753642	14	93854240	7213	0.395051	C	T	-0.0729	0.0170	1.822E-05	0.0110	0.0124	0.376816
SERPINA10	VTE	rs76185743	14	93818814	7213	0.030986	T	C	0.2188	0.0480	5.174E-06	-0.0237	0.0360	0.509588
SERPINA10	VTE	rs76249699	14	94299746	7213	0.019825	C	T	0.3431	0.0593	7.654E-09	0.0106	0.0471	0.822273
SERPINA10	VTE	rs80319562	14	94434741	7213	0.06516	G	A	0.1783	0.0338	1.424E-07	0.0116	0.0255	0.648246
SERPINA10	VTE	rs9972236	14	94104994	7213	0.15278	T	C	0.2023	0.0231	2.479E-18	-0.0327	0.0176	0.063426
OBP2B	VTE	rs117325350	9	133203126	7213	0.037779	C	T	-0.3299	0.0435	3.813E-14	-0.0485	0.0324	0.134256
OBP2B	VTE	rs117362891	9	133513472	7213	0.019825	T	C	-0.2697	0.0588	4.522E-06	0.0090	0.0435	0.836463
OBP2B	VTE	rs12340741	9	133232142	7213	0.023985	G	A	-0.5332	0.0541	8.406E-23	-0.0185	0.0402	0.645033
OBP2B	VTE	rs4454354	9	133214142	7213	0.21288	T	C	-0.3520	0.0199	1.978E-68	0.0408	0.0151	0.006974
OBP2B	VTE	rs567493	9	133318693	7213	0.272355	A	G	0.0869	0.0188	3.768E-06	-0.0880	0.0140	3E-10
OBP2B	VTE	rs60571346	9	133195896	7213	0.471025	C	T	0.1390	0.0166	7.033E-17	-0.0662	0.0123	6.75E-08
OBP2B	VTE	rs76643124	9	133491326	7213	0.028005	A	G	-0.2946	0.0503	4.889E-09	0.1727	0.0460	0.000173
EFEMP1	VTE	rs10865292	2	55988917	7213	0.088451	G	A	0.1743	0.0291	2.23E-09	0.0029	0.0219	0.896425
EFEMP1	VTE	rs114254392	2	55951979	7213	0.040829	C	A	-0.2369	0.0415	1.21E-08	0.0654	0.0304	0.031295
EFEMP1	VTE	rs114782293	2	55572110	7213	0.016151	T	A	-0.2644	0.0662	6.58E-05	0.0538	0.0493	0.274804
EFEMP1	VTE	rs114869558	2	55753290	7213	0.011299	A	G	-0.3111	0.0791	8.52E-05	-0.0056	0.0561	0.919853
EFEMP1	VTE	rs116679420	2	55629814	7213	0.03362	T	C	-0.2326	0.0460	4.40E-07	0.0343	0.0313	0.27306
EFEMP1	VTE	rs11695502	2	55867864	7213	0.103355	C	T	-0.1703	0.0273	4.81E-10	0.0228	0.0189	0.227953
EFEMP1	VTE	rs13001332	2	56083064	7213	0.099681	A	T	-0.1714	0.0280	9.24E-10	-0.0046	0.0192	0.81163
EFEMP1	VTE	rs1432559	2	55962483	7213	0.213919	G	T	-0.1490	0.0203	2.25E-13	0.0166	0.0147	0.259529
EFEMP1	VTE	rs143681669	2	55809841	7213	0.011784	A	G	0.3520	0.0770	4.96E-06	-0.1050	0.0647	0.104847
EFEMP1	VTE	rs145713418	2	55738460	7213	0.02239	A	C	-0.3195	0.0561	1.26E-08	0.0539	0.0405	0.182792
EFEMP1	VTE	rs146147002	2	56018468	7213	0.016359	A	G	-0.3353	0.0652	2.77E-07	0.0877	0.0511	0.085914
EFEMP1	VTE	rs17047269	2	55862304	7213	0.013171	A	T	0.3293	0.0734	7.37E-06	-0.0320	0.0526	0.543685
EFEMP1	VTE	rs17268653	2	56218166	7213	0.029877	G	A	0.1964	0.0487	5.48E-05	0.0313	0.0362	0.388423
EFEMP1	VTE	rs1802575	2	55866069	7213	0.124497	C	G	-0.3684	0.0248	2.28E-49	0.0571	0.0175	0.00111
EFEMP1	VTE	rs3791679	2	55869757	7213	0.240191	G	A	0.4274	0.0189	3.78E-109	-0.0353	0.0146	0.015584
EFEMP1	VTE	rs41291177	2	55983263	7213	0.219881	G	A	-0.0865	0.0201	1.63E-05	0.0183	0.0145	0.20523

EFEMP1	VTE	rs58754091	2	55842539	7213	0.174199	G	A	-0.1452	0.0219	3.60E-11	0.0226	0.0158	0.152929
EFEMP1	VTE	rs727877	2	55892375	7213	0.071953	T	A	0.2507	0.0323	8.88E-15	-0.0188	0.0242	0.438177
EFEMP1	VTE	rs736345	2	55619011	7213	0.171843	G	A	-0.1227	0.0222	3.20E-08	0.0282	0.0156	0.071175
EFEMP1	VTE	rs7583519	2	56123171	7213	0.30958	A	G	0.1135	0.0181	3.69E-10	-0.0192	0.0134	0.152443
EFEMP1	VTE	rs76771294	2	56049624	7213	0.019271	A	G	0.3274	0.0606	6.73E-08	-0.0059	0.0447	0.894131
EFEMP1	VTE	rs782628	2	55673803	7213	0.096839	A	C	0.2199	0.0282	7.50E-15	-0.0023	0.0208	0.913757
F5	VTE	rs10753787	1	169580537	7213	0.418134	T	C	0.1028	0.0168	1.043E-09	0.0284	0.0122	0.019904
F5	VTE	rs10800427	1	169303213	7213	0.053376	A	T	0.1621	0.0368	1.049E-05	0.5430	0.0257	2E-99
F5	VTE	rs111704584	1	169494768	7213	0.01837	A	G	-0.2549	0.0609	2.832E-05	-0.0474	0.0495	0.338341
F5	VTE	rs1200140	1	169113135	7213	0.212186	A	G	-0.1363	0.0203	1.847E-11	-0.0212	0.0146	0.146488
F5	VTE	rs148459673	1	169767716	7213	0.015874	A	C	-0.3586	0.0658	5.255E-08	0.1428	0.0480	0.002917
F5	VTE	rs149686001	1	169048123	7213	0.010467	A	G	-0.4696	0.0815	8.643E-09	-0.0142	0.0664	0.83059
F5	VTE	rs16861872	1	169031153	7213	0.07743	C	A	-0.1353	0.0310	1.279E-05	0.0034	0.0224	0.879715
F5	VTE	rs61805767	1	169532757	7213	0.182518	A	G	0.1291	0.0218	3.103E-09	-0.0490	0.0164	0.002715
F5	VTE	rs7415756	1	169365044	7213	0.377513	C	T	0.0903	0.0170	1.205E-07	0.0656	0.0125	1.56E-07
F5	VTE	rs76955769	1	169903560	7213	0.042146	A	G	-0.1732	0.0414	2.942E-05	-0.0353	0.0296	0.231822
F5	VTE	rs9332641	1	169524475	7213	0.058644	T	C	-0.5120	0.0350	1.106E-47	0.0549	0.0260	0.034979
MSR1	VTE	rs10090333	8	16230061	7213	0.26577	T	A	-0.0754	0.0191	7.79E-05	0.0169	0.0138	0.219729
MSR1	VTE	rs1030517	8	16330801	7213	0.170456	A	G	-0.2463	0.0223	4.069E-28	0.0206	0.0166	0.215169
MSR1	VTE	rs117609170	8	15976529	7213	0.013656	T	C	-0.4348	0.0717	1.372E-09	0.0797	0.0483	0.098971
MSR1	VTE	rs1871573	8	16166714	7213	0.474491	G	C	-0.1548	0.0166	1.551E-20	-0.0128	0.0122	0.29692
MSR1	VTE	rs1949919	8	16559760	7213	0.407389	C	A	-0.1296	0.0167	1.021E-14	-0.0083	0.0124	0.501588
MSR1	VTE	rs2410390	8	16171084	7213	0.045266	T	C	0.1586	0.0404	8.634E-05	0.0240	0.0272	0.376207
MSR1	VTE	rs41341748	8	16155085	7213	0.010259	A	G	-1.4944	0.0784	3.866E-79	0.1411	0.0541	0.009063
MSR1	VTE	rs59251421	8	16200030	7213	0.077707	T	C	-0.5130	0.0304	1.394E-62	0.0485	0.0234	0.038248
MSR1	VTE	rs7016138	8	16080855	7213	0.097324	A	C	-0.1582	0.0283	2.403E-08	0.0139	0.0210	0.507855
MSR1	VTE	rs80014125	8	16613417	7213	0.03459	A	G	0.2053	0.0454	6.115E-06	0.0083	0.0352	0.814631

Supplementary Table 4 Colocalization results of plasma pQTL and VTE GWAS.

protein	nsnps	PP.H0.abf	PP.H1.abf	PP.H2.abf	PP.H3.abf	PP.H4.abf
PLCG2	3587	7.43E-40	2.26E-03	3.18E-37	0.9661	0.0317
SERPINA10	3328	0	0.56	0	0.1752	0.2635
KNG1	2517	1.78E-72	0.28	2.61E-73	0.0401	0.6822
MSR1	3830	1.40E-58	0.57	2.03E-59	0.0814	0.3526
SERPINE2	2719	0	0.08	0	0.0073	0.9116
LRP4	1288	5.62E-198	5.67E-27	9.92E-172	1.0000	1.17E-21
F11	2973	1.39E-170	1.92E-33	1.50E-140	0.0011	0.9989
PROC	2770	2.17E-39	8.22E-03	1.00E-37	0.3788	0.6129
RARRES2	2159	9.34E-159	0.17	1.74E-159	0.0303	0.8029
GP6	2279	0	0.24	0	0.0752	0.6881
THBS2	3403	1.12E-284	0.54	3.62E-285	0.1732	0.2888
SERPINA1	3348	2.11E-183	0.03	6.79E-184	0.0102	0.9552
KLKB1	3078	1.59E-131	9.26E-31	1.71E-101	1.0000	5.97E-12
F2	1153	3.73E-37	5.94E-30	6.58E-11	4.80E-05	1.0000
F5	3270	2.21E-239	1.21E-183	1.83E-56	1.0000	1.18E-51
OBP2B	2504	1.47E-155	3.04E-93	4.82E-63	1.0000	6.56E-65
PLEK	2721	1.67E-163	2.39E-05	7.36E-162	0.0001	0.9999
EFEMP1	3081	4.99E-105	8.00E-01	5.69E-106	0.0910	0.1095
ABO	2595	0	3.04E-93	0	1.0000	1.38E-89
CEL	2370	1.15E-111	3.04E-93	3.80E-19	1.0000	5.78E-21

Supplementary Table 5 Colocalization results of snp in plasma pQTL and VTE GWAS.

snp	SNP.PP.H4	gene
rs1007738	2.64E-69	F2
rs10160701	3.31E-75	F2
rs10219383	9.05E-75	F2
rs1044796	1.97E-74	F2
rs10466476	3.50E-75	F2
rs10466477	3.45E-75	F2
rs10466478	1.41E-69	F2
rs10501319	1.21E-71	F2
rs1060573	1.21E-69	F2
rs10734548	5.59E-69	F2
rs10734549	3.76E-69	F2
rs10742784	2.66E-69	F2
rs10742797	2.61E-72	F2
rs10769199	3.26E-74	F2
rs10769204	1.36E-70	F2
rs10769205	6.62E-70	F2
rs10769208	3.80E-69	F2
rs10769211	2.39E-69	F2
rs10769212	2.31E-69	F2
rs10769225	2.21E-71	F2
rs10769226	2.59E-71	F2
rs10769227	1.98E-71	F2
rs10769228	1.87E-71	F2
rs10769230	1.69E-69	F2
rs10769233	3.50E-69	F2
rs10769234	2.42E-69	F2
rs10769239	7.86E-70	F2
rs10769244	8.33E-70	F2
rs10769245	6.79E-70	F2
rs10769248	2.18E-72	F2
rs10838579	1.21E-74	F2
rs10838586	6.00E-73	F2
rs10838587	5.19E-73	F2
rs10838588	5.29E-73	F2
rs10838589	7.69E-73	F2
rs10838590	6.52E-73	F2
rs10838595	2.02E-75	F2
rs10838596	1.58E-68	F2
rs10838601	4.30E-68	F2
rs10838602	3.98E-75	F2
rs10838610	1.44E-70	F2
rs10838611	8.90E-64	F2
rs10838612	5.78E-71	F2
rs10838613	2.73E-75	F2
rs10838620	3.66E-69	F2
rs10838621	9.76E-68	F2
rs10838624	4.03E-68	F2
rs10838627	3.42E-68	F2

rs10838628	3.60E-68	F2
rs10838629	2.26E-69	F2
rs10000459	1.08E-137	F11
rs10000731	3.85E-177	F11
rs10001084	1.56E-174	F11
rs10001377	1.01E-177	F11
rs10001944	1.11E-177	F11
rs10002009	1.14E-175	F11
rs10002028	1.07E-177	F11
rs10002129	5.67E-177	F11
rs10002339	1.39E-176	F11
rs10002348	4.27E-177	F11
rs10002615	1.11E-176	F11
rs10002763	5.38E-176	F11
rs10002784	4.63E-177	F11
rs10003196	1.49E-173	F11
rs10003363	1.37E-177	F11
rs10003588	1.55E-173	F11
rs10004157	5.62E-177	F11
rs10004205	1.19E-177	F11
rs10004538	4.81E-176	F11
rs10004932	1.17E-176	F11
rs10005888	4.63E-177	F11
rs10006277	3.33E-175	F11
rs10007140	2.42E-177	F11
rs10007356	6.14E-177	F11
rs10007676	5.81E-177	F11
rs10008039	1.56E-176	F11
rs10008621	8.09E-177	F11
rs10008724	7.75E-176	F11
rs10009030	1.59E-177	F11
rs10010181	2.42E-177	F11
rs10010232	5.27E-177	F11
rs10010668	2.21E-177	F11
rs10011164	1.05E-176	F11
rs10011311	1.43E-176	F11
rs10012237	2.25E-177	F11
rs10012258	1.51E-176	F11
rs10012858	8.42E-177	F11
rs10013071	4.22E-177	F11
rs10013195	2.11E-176	F11
rs10013218	5.32E-177	F11
rs10013254	1.34E-177	F11
rs10013461	2.79E-176	F11
rs10013653	6.91E-148	F11
rs10013900	2.54E-177	F11
rs10014399	2.79E-162	F11
rs10014510	2.26E-175	F11
rs10014524	2.42E-177	F11
rs10014983	6.46E-177	F11
rs10015147	1.24E-176	F11

rs10015908	2.57E-175	F11
rs1000012	0.00E+00	ABO
rs10115001	0.00E+00	ABO
rs10116061	0.00E+00	ABO
rs10117024	0.00E+00	ABO
rs10117159	0.00E+00	ABO
rs10117255	0.00E+00	ABO
rs10117852	0.00E+00	ABO
rs10117989	0.00E+00	ABO
rs10118950	0.00E+00	ABO
rs10119590	0.00E+00	ABO
rs10119905	0.00E+00	ABO
rs10120207	0.00E+00	ABO
rs10121171	0.00E+00	ABO
rs10121513	0.00E+00	ABO
rs10121728	0.00E+00	ABO
rs10121827	0.00E+00	ABO
rs10122534	0.00E+00	ABO
rs10124364	0.00E+00	ABO
rs10125348	0.00E+00	ABO
rs10125492	0.00E+00	ABO
rs1018857	0.00E+00	ABO
rs1034579	0.00E+00	ABO
rs1043077	0.00E+00	ABO
rs10435928	0.00E+00	ABO
rs10441806	0.00E+00	ABO
rs10491534	0.00E+00	ABO
rs10491535	0.00E+00	ABO
rs10491536	0.00E+00	ABO
rs10491903	0.00E+00	ABO
rs1050700	0.00E+00	ABO
rs1052517	0.00E+00	ABO
rs1053878	0.00E+00	ABO
rs1054379	0.00E+00	ABO
rs1055432	0.00E+00	ABO
rs1062218	0.00E+00	ABO
rs1062356	0.00E+00	ABO
rs106906	0.00E+00	ABO
rs1073123	0.00E+00	ABO
rs10736857	0.00E+00	ABO
rs10736859	0.00E+00	ABO
rs10736860	0.00E+00	ABO
rs1074052	0.00E+00	ABO
rs10751500	0.00E+00	ABO
rs10751502	0.00E+00	ABO
rs10751505	0.00E+00	ABO
rs10761397	0.00E+00	ABO
rs10761412	0.00E+00	ABO
rs10761413	0.00E+00	ABO
rs1076150	0.00E+00	ABO
rs1076151	0.00E+00	ABO

rs1000455	6.12E-45	PLCG2
rs1000456	3.33E-44	PLCG2
rs10153053	1.02E-44	PLCG2
rs10153151	4.38E-45	PLCG2
rs1017237	3.54E-44	PLCG2
rs1017238	1.45E-44	PLCG2
rs1017242	4.53E-45	PLCG2
rs1017243	4.38E-45	PLCG2
rs10220955	8.84E-45	PLCG2
rs1027213	1.76E-44	PLCG2
rs1032339	3.66E-45	PLCG2
rs1035572	4.02E-45	PLCG2
rs1035573	6.01E-45	PLCG2
rs1037971	8.83E-44	PLCG2
rs1037972	8.63E-44	PLCG2
rs10438632	5.44E-45	PLCG2
rs10454708	7.01E-44	PLCG2
rs10493891	2.24E-45	PLCG2
rs10514517	3.71E-43	PLCG2
rs10514518	4.66E-44	PLCG2
rs10514519	4.46E-43	PLCG2
rs10514520	1.11E-43	PLCG2
rs10514521	1.06E-43	PLCG2
rs10514523	2.29E-45	PLCG2
rs10514524	2.12E-45	PLCG2
rs10514525	1.68E-45	PLCG2
rs10514527	1.75E-45	PLCG2
rs10514528	2.11E-45	PLCG2
rs10514529	5.05E-44	PLCG2
rs10514530	6.27E-44	PLCG2
rs10514531	3.61E-45	PLCG2
rs10514532	4.10E-45	PLCG2
rs10514533	4.19E-45	PLCG2
rs10514534	4.10E-45	PLCG2
rs1056629	3.53E-45	PLCG2
rs1056654	3.54E-45	PLCG2
rs1056675	2.95E-45	PLCG2
rs1056690	3.54E-45	PLCG2
rs1064208	3.02E-45	PLCG2
rs1071644	4.23E-21	PLCG2
rs10871410	9.37E-45	PLCG2
rs10871411	1.08E-44	PLCG2
rs10871413	3.58E-45	PLCG2
rs10871414	1.91E-45	PLCG2
rs10871415	2.18E-45	PLCG2
rs10871420	1.76E-43	PLCG2
rs10871424	2.72E-45	PLCG2
rs10871425	6.72E-45	PLCG2
rs111302260	3.88E-45	PLCG2
rs111362226	1.71E-44	PLCG2
rs1007738	1.14E-153	LRP4

rs10160701	4.12E-182	LRP4
rs10466476	5.84E-181	LRP4
rs10466477	8.05E-181	LRP4
rs10466478	4.42E-155	LRP4
rs10501319	5.27E-177	LRP4
rs10501320	9.94E-183	LRP4
rs10501321	1.67E-165	LRP4
rs1050244	2.68E-183	LRP4
rs1051006	2.86E-174	LRP4
rs1052373	9.09E-166	LRP4
rs1055510	1.37E-165	LRP4
rs1057233	1.00E-175	LRP4
rs1060573	4.01E-155	LRP4
rs10734548	1.41E-153	LRP4
rs10734549	1.89E-153	LRP4
rs10734557	2.36E-176	LRP4
rs10742784	9.01E-154	LRP4
rs10742797	7.27E-179	LRP4
rs10742799	1.42E-165	LRP4
rs10742801	1.34E-165	LRP4
rs10747	1.23E-177	LRP4
rs10769204	2.17E-149	LRP4
rs10769205	4.55E-134	LRP4
rs10769208	1.91E-153	LRP4
rs10769211	5.84E-154	LRP4
rs10769212	9.08E-154	LRP4
rs10769225	2.23E-176	LRP4
rs10769226	2.29E-176	LRP4
rs10769227	2.11E-176	LRP4
rs10769228	1.90E-176	LRP4
rs10769230	7.15E-154	LRP4
rs10769233	3.27E-153	LRP4
rs10769234	4.75E-154	LRP4
rs10769239	7.97E-156	LRP4
rs10769244	8.53E-156	LRP4
rs10769245	6.05E-156	LRP4
rs10769248	8.91E-179	LRP4
rs10769252	9.39E-166	LRP4
rs10769253	6.16E-175	LRP4
rs10769254	1.18E-171	LRP4
rs10769255	1.62E-175	LRP4
rs10769256	8.76E-184	LRP4
rs10838601	2.84E-158	LRP4
rs10838602	2.09E-182	LRP4
rs10838610	4.26E-149	LRP4
rs10838611	8.51E-78	LRP4
rs10838612	1.72E-144	LRP4
rs10838613	7.77E-181	LRP4
rs10838620	1.82E-153	LRP4
rs1000504	5.14E-170	PLEK
rs1002537	2.02E-168	PLEK

rs10153623	3.86E-169	PLEK
rs1016349	1.56E-139	PLEK
rs1016350	9.15E-140	PLEK
rs10164780	2.27E-159	PLEK
rs10164991	3.46E-167	PLEK
rs10166882	2.33E-167	PLEK
rs10167016	1.38E-167	PLEK
rs10167373	2.28E-167	PLEK
rs10167610	2.22E-170	PLEK
rs10167650	3.42E-120	PLEK
rs10167680	2.28E-167	PLEK
rs10167684	2.28E-167	PLEK
rs10167733	2.18E-170	PLEK
rs10168414	1.20E-152	PLEK
rs10169751	4.39E-169	PLEK
rs10170087	2.37E-170	PLEK
rs10171075	1.29E-170	PLEK
rs10171076	6.06E-169	PLEK
rs10171662	1.47E-170	PLEK
rs10171786	2.69E-170	PLEK
rs10171972	2.10E-170	PLEK
rs10172004	7.74E-167	PLEK
rs10172125	2.32E-159	PLEK
rs10172469	1.41E-167	PLEK
rs10172974	1.86E-167	PLEK
rs10173197	2.68E-167	PLEK
rs10173449	2.38E-167	PLEK
rs10173501	3.99E-167	PLEK
rs10173586	6.38E-169	PLEK
rs10174380	7.99E-140	PLEK
rs10174579	1.26E-139	PLEK
rs10174998	1.13E-167	PLEK
rs10175092	6.47E-170	PLEK
rs10176108	4.45E-170	PLEK
rs10176505	7.10E-167	PLEK
rs10176792	8.15E-167	PLEK
rs10177248	2.08E-170	PLEK
rs10177314	1.85E-167	PLEK
rs10178143	1.96E-168	PLEK
rs10178792	2.33E-51	PLEK
rs10179154	4.85E-168	PLEK
rs10179623	2.77E-167	PLEK
rs10179993	1.93E-169	PLEK
rs10180188	2.99E-169	PLEK
rs10180387	3.45E-167	PLEK
rs10180585	5.84E-170	PLEK
rs10180676	2.59E-170	PLEK
rs10180779	2.42E-167	PLEK
rs10000459	4.02E-43	KLKB1
rs10000731	1.67E-126	KLKB1
rs10000785	2.28E-127	KLKB1

rs10001084	2.01E-124	KLKB1
rs10001377	1.94E-127	KLKB1
rs10001944	2.67E-127	KLKB1
rs10002009	2.29E-125	KLKB1
rs10002028	2.68E-127	KLKB1
rs10002129	9.69E-127	KLKB1
rs10002339	4.30E-127	KLKB1
rs10002348	3.03E-127	KLKB1
rs10002615	4.61E-127	KLKB1
rs10002763	4.89E-127	KLKB1
rs10002784	6.45E-127	KLKB1
rs10003196	3.19E-124	KLKB1
rs10003363	2.10E-127	KLKB1
rs10003588	3.13E-124	KLKB1
rs10004157	1.41E-126	KLKB1
rs10004205	2.30E-127	KLKB1
rs10004538	3.00E-124	KLKB1
rs10004932	2.55E-55	KLKB1
rs10005888	2.74E-127	KLKB1
rs10006277	6.93E-126	KLKB1
rs10007140	3.95E-127	KLKB1
rs10007356	1.69E-126	KLKB1
rs10007676	4.38E-127	KLKB1
rs10008039	2.68E-126	KLKB1
rs10008621	2.45E-126	KLKB1
rs10008724	3.82E-126	KLKB1
rs10009030	2.59E-127	KLKB1
rs10009306	2.27E-126	KLKB1
rs10010181	4.01E-127	KLKB1
rs10010232	2.93E-127	KLKB1
rs10010668	5.12E-127	KLKB1
rs10011164	3.81E-126	KLKB1
rs10011292	1.07E-126	KLKB1
rs10011311	6.07E-127	KLKB1
rs10012237	2.64E-127	KLKB1
rs10012258	3.36E-126	KLKB1
rs10012858	2.59E-127	KLKB1
rs10013071	3.61E-127	KLKB1
rs10013195	3.85E-126	KLKB1
rs10013218	2.94E-127	KLKB1
rs10013254	2.71E-127	KLKB1
rs10013461	2.51E-126	KLKB1
rs10013653	2.28E-75	KLKB1
rs10013900	5.21E-127	KLKB1
rs10014399	1.41E-104	KLKB1
rs10014510	5.24E-126	KLKB1
rs10014865	5.24E-125	KLKB1
rs10048716	4.26E-46	PROC
rs10048749	9.57E-46	PROC
rs1011019	1.70E-19	PROC
rs10164978	8.76E-46	PROC

rs10165440	7.39E-46	PROC
rs10166461	6.77E-46	PROC
rs10167189	2.57E-46	PROC
rs10167294	4.88E-34	PROC
rs10168813	1.09E-44	PROC
rs10169028	9.11E-44	PROC
rs10169310	2.95E-46	PROC
rs10171345	7.86E-45	PROC
rs10171785	7.35E-46	PROC
rs10171898	6.59E-46	PROC
rs10172249	7.29E-46	PROC
rs10173388	7.35E-46	PROC
rs10174611	1.50E-45	PROC
rs10177052	4.38E-38	PROC
rs10178163	3.69E-45	PROC
rs10178188	5.79E-43	PROC
rs10178199	9.53E-46	PROC
rs10178499	3.69E-45	PROC
rs10180714	9.54E-46	PROC
rs10180750	1.47E-45	PROC
rs10180840	2.45E-46	PROC
rs10180930	3.63E-46	PROC
rs10182292	1.01E-45	PROC
rs10182429	5.76E-46	PROC
rs10183605	2.71E-46	PROC
rs10183797	6.12E-46	PROC
rs10185083	9.52E-46	PROC
rs10185637	3.51E-46	PROC
rs10186397	5.82E-46	PROC
rs10188441	9.11E-44	PROC
rs10189694	8.99E-44	PROC
rs10189721	7.65E-46	PROC
rs10190229	1.76E-45	PROC
rs10190872	4.20E-45	PROC
rs10191259	2.47E-46	PROC
rs10191573	9.26E-44	PROC
rs10193351	7.28E-46	PROC
rs10193937	1.49E-45	PROC
rs10194052	6.39E-46	PROC
rs10194509	4.61E-46	PROC
rs10194711	2.00E-45	PROC
rs10194727	1.44E-39	PROC
rs10194975	3.00E-45	PROC
rs10195067	2.98E-45	PROC
rs10195774	1.05E-43	PROC
rs10195813	1.01E-40	PROC
rs1003410	1.89E-79	KNG1
rs1004588	6.10E-78	KNG1
rs1004589	2.46E-78	KNG1
rs1009640	2.06E-78	KNG1
rs1011551	1.15E-77	KNG1

rs1014695	1.93E-78	KNG1
rs10212190	2.84E-79	KNG1
rs10212404	1.01E-78	KNG1
rs1029353	1.22E-76	KNG1
rs1030109	1.62E-77	KNG1
rs1042445	1.56E-78	KNG1
rs1042464	4.44E-79	KNG1
rs1042642	2.02E-79	KNG1
rs1042757	2.48E-79	KNG1
rs10439970	6.80E-71	KNG1
rs10439971	5.42E-71	KNG1
rs10440056	5.42E-71	KNG1
rs10440057	5.47E-71	KNG1
rs10470529	1.69E-77	KNG1
rs1047115	1.58E-78	KNG1
rs1047148	1.06E-78	KNG1
rs10490807	8.69E-79	KNG1
rs1050274	7.49E-71	KNG1
rs10513802	1.46E-78	KNG1
rs10513803	3.68E-77	KNG1
rs10513804	4.76E-79	KNG1
rs10513807	2.47E-79	KNG1
rs10513808	1.45E-78	KNG1
rs10513809	1.50E-78	KNG1
rs10513810	6.55E-78	KNG1
rs10513812	8.51E-78	KNG1
rs1063537	8.06E-78	KNG1
rs1063538	1.34E-78	KNG1
rs1063539	8.90E-78	KNG1
rs1071592	9.94E-78	KNG1
rs10770	4.00E-76	KNG1
rs10804905	1.15E-78	KNG1
rs10804906	7.75E-79	KNG1
rs10804907	6.43E-79	KNG1
rs10804908	1.64E-78	KNG1
rs10937246	1.75E-78	KNG1
rs10937251	3.61E-78	KNG1
rs10937252	6.74E-79	KNG1
rs10937253	4.98E-79	KNG1
rs10937254	1.30E-77	KNG1
rs10937255	1.39E-77	KNG1
rs10937261	6.02E-77	KNG1
rs10937263	6.37E-77	KNG1
rs10937264	3.31E-77	KNG1
rs10937265	6.84E-77	KNG1
rs1001844	1.82E-290	THBS2
rs1002234	4.70E-291	THBS2
rs1003492	2.10E-290	THBS2
rs1004298	2.85E-289	THBS2
rs10080336	1.99E-290	THBS2
rs1014449	5.93E-290	THBS2

rs1018559	3.04E-287	THBS2
rs10223642	9.53E-291	THBS2
rs10280	1.68E-195	THBS2
rs1028295	2.03E-288	THBS2
rs1028296	3.02E-290	THBS2
rs1033458	6.45E-287	THBS2
rs1040	1.86E-195	THBS2
rs1040932	4.09E-290	THBS2
rs1040933	8.86E-291	THBS2
rs1042	1.83E-243	THBS2
rs10455733	3.40E-291	THBS2
rs10455734	3.36E-291	THBS2
rs10455736	1.98E-288	THBS2
rs10455737	1.98E-288	THBS2
rs10455738	5.59E-290	THBS2
rs10455740	1.81E-290	THBS2
rs10455744	2.97E-290	THBS2
rs1047910	3.60E-284	THBS2
rs10485330	1.09E-289	THBS2
rs10485332	9.10E-263	THBS2
rs10485333	6.31E-288	THBS2
rs1056672	1.59E-284	THBS2
rs1072852	3.62E-290	THBS2
rs10755551	3.24E-291	THBS2
rs10755552	3.24E-291	THBS2
rs10755553	1.24E-289	THBS2
rs10755554	2.46E-290	THBS2
rs10755555	5.15E-290	THBS2
rs10755558	2.18E-288	THBS2
rs1077326	7.88E-290	THBS2
rs10806650	3.24E-291	THBS2
rs10806651	3.15E-291	THBS2
rs10806653	7.14E-291	THBS2
rs10806654	3.20E-290	THBS2
rs10806655	2.21E-290	THBS2
rs10806656	1.44E-289	THBS2
rs10806659	5.44E-291	THBS2
rs10806660	7.97E-290	THBS2
rs10806662	2.65E-290	THBS2
rs10806663	4.55E-290	THBS2
rs10806665	1.49E-275	THBS2
rs10806670	1.68E-284	THBS2
rs10945304	7.01E-288	THBS2
rs10945306	5.26E-288	THBS2
rs1002520	8.79E-188	SERPINA1
rs1003740	2.29E-189	SERPINA1
rs1004958	3.30E-188	SERPINA1
rs1005348	2.02E-188	SERPINA1
rs1007148	3.27E-186	SERPINA1
rs1007149	3.69E-186	SERPINA1
rs10083317	2.71E-190	SERPINA1

rs10083334	1.55E-189	SERPINA1
rs1012016	8.77E-180	SERPINA1
rs1012808	1.29E-183	SERPINA1
rs10129195	1.04E-188	SERPINA1
rs10129374	4.90E-190	SERPINA1
rs10129381	1.13E-188	SERPINA1
rs10129487	6.92E-189	SERPINA1
rs10129500	3.12E-123	SERPINA1
rs10129651	7.03E-189	SERPINA1
rs10129672	1.23E-187	SERPINA1
rs10129705	6.76E-188	SERPINA1
rs10129953	2.25E-189	SERPINA1
rs10130697	1.28E-183	SERPINA1
rs10130707	3.54E-190	SERPINA1
rs10130906	2.24E-187	SERPINA1
rs10130927	7.38E-190	SERPINA1
rs10130954	3.27E-184	SERPINA1
rs10130996	2.24E-187	SERPINA1
rs10131174	3.15E-184	SERPINA1
rs10131610	2.14E-189	SERPINA1
rs10131646	1.29E-189	SERPINA1
rs10131729	8.71E-190	SERPINA1
rs10131754	1.67E-190	SERPINA1
rs10131818	1.29E-189	SERPINA1
rs10132066	1.76E-178	SERPINA1
rs10132071	3.81E-189	SERPINA1
rs10132074	3.09E-190	SERPINA1
rs10132232	5.78E-189	SERPINA1
rs10132253	1.74E-189	SERPINA1
rs10132388	8.94E-189	SERPINA1
rs10132393	8.42E-189	SERPINA1
rs10132395	4.21E-190	SERPINA1
rs10132441	3.97E-189	SERPINA1
rs10132534	1.42E-189	SERPINA1
rs10132928	9.78E-188	SERPINA1
rs10132931	8.65E-189	SERPINA1
rs10133253	2.00E-188	SERPINA1
rs10133452	2.47E-190	SERPINA1
rs10133592	1.96E-184	SERPINA1
rs10133701	2.21E-187	SERPINA1
rs10133716	5.29E-189	SERPINA1
rs10133793	2.19E-187	SERPINA1
rs10133971	2.15E-189	SERPINA1
rs10046529	1.33E-165	RARRES2
rs1006581	2.20E-165	RARRES2
rs10085554	4.86E-164	RARRES2
rs10085672	2.43E-164	RARRES2
rs1010614	1.75E-148	RARRES2
rs10155859	2.81E-165	RARRES2
rs10156097	1.28E-149	RARRES2
rs1016193	1.24E-165	RARRES2

rs1021185	1.25E-165	RARRES2
rs10215009	4.14E-164	RARRES2
rs10215048	1.22E-164	RARRES2
rs10215091	1.61E-165	RARRES2
rs10215092	1.21E-164	RARRES2
rs10215370	8.19E-165	RARRES2
rs10215381	7.90E-165	RARRES2
rs10215382	8.84E-165	RARRES2
rs10215385	8.73E-165	RARRES2
rs10216102	1.59E-165	RARRES2
rs10224387	1.10E-165	RARRES2
rs10225462	2.09E-165	RARRES2
rs10226091	4.04E-164	RARRES2
rs10226213	1.11E-165	RARRES2
rs10226390	3.47E-165	RARRES2
rs10226860	5.70E-164	RARRES2
rs10226892	2.77E-165	RARRES2
rs10227049	1.05E-165	RARRES2
rs10227094	1.36E-165	RARRES2
rs10227257	1.18E-165	RARRES2
rs10227304	2.28E-165	RARRES2
rs10227548	3.18E-165	RARRES2
rs10227762	1.85E-163	RARRES2
rs10227906	1.15E-165	RARRES2
rs10227987	1.87E-163	RARRES2
rs10228706	2.14E-165	RARRES2
rs10229175	1.50E-163	RARRES2
rs10230559	2.90E-165	RARRES2
rs10231041	1.81E-165	RARRES2
rs10231133	4.89E-164	RARRES2
rs10231216	1.73E-165	RARRES2
rs10231408	5.18E-164	RARRES2
rs10231482	5.27E-164	RARRES2
rs10231567	2.31E-159	RARRES2
rs10231579	1.19E-164	RARRES2
rs10231759	3.72E-164	RARRES2
rs10232092	2.50E-165	RARRES2
rs10232947	1.70E-165	RARRES2
rs10233783	1.03E-164	RARRES2
rs10234866	2.82E-163	RARRES2
rs10234910	1.24E-165	RARRES2
rs10235151	1.72E-165	RARRES2
rs1000012	4.80E-97	CEL
rs10115625	2.28E-97	CEL
rs10116061	1.74E-98	CEL
rs10117024	2.59E-97	CEL
rs10117159	1.72E-92	CEL
rs10117255	3.31E-93	CEL
rs10117989	1.85E-98	CEL
rs10118950	1.30E-95	CEL
rs10119590	1.36E-97	CEL

rs10119905	1.07E-95	CEL
rs10120207	7.79E-98	CEL
rs10121171	1.84E-98	CEL
rs10121513	1.28E-97	CEL
rs10121728	3.49E-98	CEL
rs10122534	2.10E-98	CEL
rs10123872	3.98E-98	CEL
rs10124364	4.18E-93	CEL
rs10125348	6.31E-98	CEL
rs10125492	6.17E-98	CEL
rs1018857	1.44E-97	CEL
rs1028892	1.29E-97	CEL
rs1034579	6.70E-98	CEL
rs10435974	2.77E-98	CEL
rs10435975	2.94E-98	CEL
rs10441806	1.97E-91	CEL
rs1045737	6.84E-98	CEL
rs1045740	5.82E-98	CEL
rs10491534	3.82E-97	CEL
rs10491535	6.49E-98	CEL
rs10491536	9.61E-98	CEL
rs10491903	8.56E-98	CEL
rs10491904	2.05E-98	CEL
rs10491905	1.03E-97	CEL
rs1050700	7.91E-98	CEL
rs10512415	7.62E-98	CEL
rs1052517	1.88E-94	CEL
rs1053878	3.12E-96	CEL
rs1054379	3.74E-95	CEL
rs1055432	3.28E-97	CEL
rs1062218	4.27E-98	CEL
rs1062356	3.44E-89	CEL
rs106906	2.78E-97	CEL
rs1073123	6.56E-98	CEL
rs10736856	8.58E-98	CEL
rs10736857	1.64E-97	CEL
rs10736859	2.59E-98	CEL
rs10736860	3.19E-94	CEL
rs10751495	8.56E-98	CEL
rs10751500	1.14E-94	CEL
rs10751502	9.08E-94	CEL
rs1000319	0.00E+00	GP6
rs1000320	0.00E+00	GP6
rs1000321	0.00E+00	GP6
rs1004443	0.00E+00	GP6
rs1036231	0.00E+00	GP6
rs10401201	0.00E+00	GP6
rs10401467	0.00E+00	GP6
rs10401558	0.00E+00	GP6
rs10401614	0.00E+00	GP6
rs10401701	0.00E+00	GP6

rs10401754	0.00E+00	GP6
rs10401802	0.00E+00	GP6
rs10401863	0.00E+00	GP6
rs10402153	0.00E+00	GP6
rs10402311	0.00E+00	GP6
rs10402335	0.00E+00	GP6
rs10402506	0.00E+00	GP6
rs10402577	0.00E+00	GP6
rs10402581	0.00E+00	GP6
rs10402586	0.00E+00	GP6
rs10402708	0.00E+00	GP6
rs10402725	0.00E+00	GP6
rs10402743	0.00E+00	GP6
rs10403022	0.00E+00	GP6
rs10403074	0.00E+00	GP6
rs10403091	0.00E+00	GP6
rs10403164	0.00E+00	GP6
rs10403270	0.00E+00	GP6
rs10403361	0.00E+00	GP6
rs10403406	0.00E+00	GP6
rs10403552	0.00E+00	GP6
rs10403570	0.00E+00	GP6
rs10403571	0.00E+00	GP6
rs10404178	0.00E+00	GP6
rs10405231	0.00E+00	GP6
rs10405277	0.00E+00	GP6
rs10405574	0.00E+00	GP6
rs10406079	0.00E+00	GP6
rs10406989	0.00E+00	GP6
rs10407001	0.00E+00	GP6
rs10407009	0.00E+00	GP6
rs10407012	0.00E+00	GP6
rs10407760	0.00E+00	GP6
rs10408175	0.00E+00	GP6
rs10408421	0.00E+00	GP6
rs10408436	0.00E+00	GP6
rs10409029	0.00E+00	GP6
rs10409108	0.00E+00	GP6
rs10409160	0.00E+00	GP6
rs10409253	0.00E+00	GP6
rs1000520	0.00E+00	SERPINE2
rs1001393	0.00E+00	SERPINE2
rs1002998	0.00E+00	SERPINE2
rs1006212	0.00E+00	SERPINE2
rs1006213	0.00E+00	SERPINE2
rs1006214	0.00E+00	SERPINE2
rs1008415	0.00E+00	SERPINE2
rs1009270	0.00E+00	SERPINE2
rs1010837	0.00E+00	SERPINE2
rs1014491	0.00E+00	SERPINE2
rs1014492	0.00E+00	SERPINE2

rs10153617	0.00E+00	SERPINE2
rs10164837	0.00E+00	SERPINE2
rs10165501	0.00E+00	SERPINE2
rs10166590	0.00E+00	SERPINE2
rs10166620	0.00E+00	SERPINE2
rs10166881	0.00E+00	SERPINE2
rs10166898	0.00E+00	SERPINE2
rs10166964	0.00E+00	SERPINE2
rs10167081	0.00E+00	SERPINE2
rs10167473	0.00E+00	SERPINE2
rs10168322	0.00E+00	SERPINE2
rs10168404	0.00E+00	SERPINE2
rs10168863	0.00E+00	SERPINE2
rs10169504	0.00E+00	SERPINE2
rs10169530	0.00E+00	SERPINE2
rs10169710	0.00E+00	SERPINE2
rs10170379	0.00E+00	SERPINE2
rs10170775	0.00E+00	SERPINE2
rs10171423	0.00E+00	SERPINE2
rs10171985	0.00E+00	SERPINE2
rs10172605	0.00E+00	SERPINE2
rs10172774	0.00E+00	SERPINE2
rs10172799	0.00E+00	SERPINE2
rs10173406	0.00E+00	SERPINE2
rs10173508	0.00E+00	SERPINE2
rs10173653	0.00E+00	SERPINE2
rs10174003	0.00E+00	SERPINE2
rs10174215	0.00E+00	SERPINE2
rs10174440	0.00E+00	SERPINE2
rs1017448	0.00E+00	SERPINE2
rs10174705	0.00E+00	SERPINE2
rs10175515	0.00E+00	SERPINE2
rs10176412	0.00E+00	SERPINE2
rs10176735	0.00E+00	SERPINE2
rs10176854	0.00E+00	SERPINE2
rs10177151	0.00E+00	SERPINE2
rs10177259	0.00E+00	SERPINE2
rs10177420	0.00E+00	SERPINE2
rs10177693	0.00E+00	SERPINE2
rs1001381	0.00E+00	SERPINA10
rs1002520	0.00E+00	SERPINA10
rs1003740	0.00E+00	SERPINA10
rs1004027	0.00E+00	SERPINA10
rs1004958	0.00E+00	SERPINA10
rs1005348	0.00E+00	SERPINA10
rs1007148	0.00E+00	SERPINA10
rs1007149	0.00E+00	SERPINA10
rs10083317	0.00E+00	SERPINA10
rs10083334	0.00E+00	SERPINA10
rs1012016	0.00E+00	SERPINA10
rs1012808	0.00E+00	SERPINA10

rs10129195	0.00E+00	SERPINA10
rs10129374	0.00E+00	SERPINA10
rs10129381	0.00E+00	SERPINA10
rs10129487	0.00E+00	SERPINA10
rs10129500	0.00E+00	SERPINA10
rs10129651	0.00E+00	SERPINA10
rs10129672	0.00E+00	SERPINA10
rs10129705	0.00E+00	SERPINA10
rs10129953	0.00E+00	SERPINA10
rs10130697	1.12E-304	SERPINA10
rs10130707	0.00E+00	SERPINA10
rs10130906	0.00E+00	SERPINA10
rs10130954	89668569378	SERPINA10
rs10130996	0.00E+00	SERPINA10
rs10131174	89363964246	SERPINA10
rs10131610	0.00E+00	SERPINA10
rs10131646	0.00E+00	SERPINA10
rs10131729	0.00E+00	SERPINA10
rs10131754	0.00E+00	SERPINA10
rs10131818	0.00E+00	SERPINA10
rs10131882	0.00E+00	SERPINA10
rs10132066	2.28E-283	SERPINA10
rs10132071	0.00E+00	SERPINA10
rs10132074	0.00E+00	SERPINA10
rs10132232	0.00E+00	SERPINA10
rs10132253	0.00E+00	SERPINA10
rs10132388	0.00E+00	SERPINA10
rs10132393	0.00E+00	SERPINA10
rs10132395	0.00E+00	SERPINA10
rs10132534	0.00E+00	SERPINA10
rs10132668	0.00E+00	SERPINA10
rs10132928	0.00E+00	SERPINA10
rs10132931	0.00E+00	SERPINA10
rs10133253	0.00E+00	SERPINA10
rs10133592	0.00E+00	SERPINA10
rs10133607	0.00E+00	SERPINA10
rs10133685	0.00E+00	SERPINA10
rs10133701	0.00E+00	SERPINA10
rs1000012	1.20E-97	OBP2B
rs10115001	1.42E-97	OBP2B
rs10115625	4.59E-98	OBP2B
rs10116061	1.06E-97	OBP2B
rs10117024	2.89E-98	OBP2B
rs10117159	1.49E-92	OBP2B
rs10117255	2.49E-92	OBP2B
rs10117989	5.66E-98	OBP2B
rs10118950	1.78E-98	OBP2B
rs10119590	1.41E-77	OBP2B
rs10119905	1.77E-98	OBP2B
rs10120207	5.01E-98	OBP2B
rs10121171	5.65E-98	OBP2B

rs10121513	5.70E-98	OBP2B
rs10121728	4.00E-98	OBP2B
rs10121827	2.40E-97	OBP2B
rs10122534	7.08E-98	OBP2B
rs10123872	5.32E-98	OBP2B
rs10124364	4.12E-92	OBP2B
rs10125348	3.00E-98	OBP2B
rs10125492	3.16E-98	OBP2B
rs1018857	1.60E-97	OBP2B
rs1034579	5.20E-98	OBP2B
rs10435928	3.81E-98	OBP2B
rs10441806	4.46E-91	OBP2B
rs10491534	3.81E-97	OBP2B
rs10491535	7.77E-98	OBP2B
rs10491536	8.54E-98	OBP2B
rs10491903	8.97E-98	OBP2B
rs1050700	3.77E-98	OBP2B
rs1052517	2.33E-57	OBP2B
rs1053878	4.05E-96	OBP2B
rs1054379	5.49E-96	OBP2B
rs1055432	1.09E-97	OBP2B
rs1062218	1.51E-97	OBP2B
rs1062356	3.46E-98	OBP2B
rs106906	1.91E-97	OBP2B
rs1073123	8.16E-98	OBP2B
rs10736857	4.01E-98	OBP2B
rs10736859	1.05E-94	OBP2B
rs10736860	3.06E-48	OBP2B
rs1074052	1.73E-98	OBP2B
rs10751495	3.78E-98	OBP2B
rs10751500	1.00E-91	OBP2B
rs10751502	1.38E-96	OBP2B
rs10751505	6.20E-96	OBP2B
rs10761397	1.90E-98	OBP2B
rs10761412	6.00E-98	OBP2B
rs10761413	2.04E-98	OBP2B
rs1076150	1.78E-93	OBP2B
rs1001471	5.95E-111	EFEMP1
rs1013138	1.38E-110	EFEMP1
rs1016339	1.86E-110	EFEMP1
rs1016340	6.35E-111	EFEMP1
rs10165864	5.15E-111	EFEMP1
rs10166638	4.30E-111	EFEMP1
rs10166672	1.11E-110	EFEMP1
rs10167115	2.48E-109	EFEMP1
rs10167227	2.17E-79	EFEMP1
rs10168390	1.18E-110	EFEMP1
rs10169061	3.92E-110	EFEMP1
rs10169185	9.04E-111	EFEMP1
rs10170404	1.47E-106	EFEMP1
rs10170461	4.04E-110	EFEMP1

rs10171335	4.08E-107	EFEMP1
rs10171458	2.71E-110	EFEMP1
rs1017178	2.48E-110	EFEMP1
rs1017179	2.88E-110	EFEMP1
rs10171800	1.57E-107	EFEMP1
rs10172020	1.78E-110	EFEMP1
rs10172181	3.41E-105	EFEMP1
rs10172460	1.31E-110	EFEMP1
rs10172506	3.77E-105	EFEMP1
rs10172729	1.05E-109	EFEMP1
rs10172790	1.16E-109	EFEMP1
rs10173150	2.29E-105	EFEMP1
rs10173165	9.77E-106	EFEMP1
rs10173401	1.23E-108	EFEMP1
rs10173540	2.32E-110	EFEMP1
rs10173561	6.70E-111	EFEMP1
rs1017371	8.46E-104	EFEMP1
rs10174335	2.30E-67	EFEMP1
rs10175691	7.44E-111	EFEMP1
rs10176080	7.02E-110	EFEMP1
rs10176385	5.89E-110	EFEMP1
rs10176451	1.04E-110	EFEMP1
rs10176730	1.06E-105	EFEMP1
rs10177559	1.78E-109	EFEMP1
rs10177584	2.63E-109	EFEMP1
rs10178481	1.21E-105	EFEMP1
rs10178508	1.39E-108	EFEMP1
rs10178971	5.33E-111	EFEMP1
rs10179694	1.14E-107	EFEMP1
rs10179998	5.40E-111	EFEMP1
rs10180043	1.37E-104	EFEMP1
rs10180182	1.15E-109	EFEMP1
rs10180643	2.00E-110	EFEMP1
rs10181430	2.20E-110	EFEMP1
rs10181648	6.28E-111	EFEMP1
rs10182056	5.99E-109	EFEMP1
rs10047168	1.95E-193	F5
rs10081959	3.76E-186	F5
rs10081960	1.84E-182	F5
rs10082323	3.68E-195	F5
rs1011266	1.06E-190	F5
rs1011638	7.53E-191	F5
rs1014965	4.28E-195	F5
rs10157246	1.95E-195	F5
rs10157266	2.18E-195	F5
rs10157398	2.12E-195	F5
rs10157960	1.08E-193	F5
rs10158131	1.84E-191	F5
rs10158265	5.27E-193	F5
rs10158595	7.90E-183	F5
rs10158861	6.39E-193	F5

rs10159425	1.14E-194	F5
rs1018827	1.41E-111	F5
rs1018828	3.40E-194	F5
rs1018831	5.22E-185	F5
rs1018832	2.07E-180	F5
rs1024340	5.35E-194	F5
rs1024341	5.54E-194	F5
rs1028180	5.32E-194	F5
rs1040500	5.90E-194	F5
rs1040501	5.92E-194	F5
rs1040502	7.01E-190	F5
rs1040503	1.03E-191	F5
rs1040504	5.04E-195	F5
rs1040505	5.12E-195	F5
rs10429893	9.13E-184	F5
rs10442644	2.60E-195	F5
rs10442649	2.33E-195	F5
rs10458388	2.21E-180	F5
rs10458394	2.53E-180	F5
rs10458395	3.80E-188	F5
rs10458396	3.81E-188	F5
rs10458397	3.82E-188	F5
rs1046058	3.32E-193	F5
rs10465575	3.52E-184	F5
rs10465576	7.92E-184	F5
rs10465577	1.96E-193	F5
rs10489170	4.48E-194	F5
rs10489172	5.82E-195	F5
rs10489173	3.13E-193	F5
rs10489174	3.34E-193	F5
rs10489175	4.29E-195	F5
rs10489176	3.54E-193	F5
rs10489177	5.28E-194	F5
rs10489179	8.58E-195	F5
rs10489180	8.66E-194	F5
rs10046696	1.43E-63	MSR1
rs10086239	3.36E-64	MSR1
rs10086281	3.94E-60	MSR1
rs10086744	9.33E-65	MSR1
rs10086945	4.91E-65	MSR1
rs10087035	5.31E-58	MSR1
rs10087431	1.99E-63	MSR1
rs10087975	2.05E-63	MSR1
rs10088332	3.19E-65	MSR1
rs10088456	3.16E-65	MSR1
rs10088599	7.94E-64	MSR1
rs10088735	8.09E-63	MSR1
rs10088792	3.65E-52	MSR1
rs10089369	6.21E-65	MSR1
rs10089419	3.83E-64	MSR1
rs10089515	1.35E-64	MSR1

rs10089993	5.05E-64	MSR1
rs10090330	7.23E-65	MSR1
rs10090333	1.60E-61	MSR1
rs10091296	2.81E-64	MSR1
rs10091356	9.78E-65	MSR1
rs10091618	4.33E-64	MSR1
rs10092182	6.79E-65	MSR1
rs1009307	8.16E-63	MSR1
rs10093075	3.01E-64	MSR1
rs1009308	1.19E-57	MSR1
rs10093626	3.82E-59	MSR1
rs10093681	4.34E-64	MSR1
rs10093893	1.60E-63	MSR1
rs10094036	1.99E-63	MSR1
rs10094335	3.13E-62	MSR1
rs10094611	1.01E-63	MSR1
rs10094727	3.79E-62	MSR1
rs10095023	4.07E-57	MSR1
rs10095510	5.54E-52	MSR1
rs10096072	1.66E-63	MSR1
rs10096600	2.94E-65	MSR1
rs10097138	8.43E-65	MSR1
rs10097239	1.80E-63	MSR1
rs10098072	8.15E-64	MSR1
rs10098364	6.43E-58	MSR1
rs10098429	2.20E-61	MSR1
rs10098551	1.59E-63	MSR1
rs10099016	1.13E-63	MSR1
rs10099038	1.63E-64	MSR1
rs10099042	6.23E-64	MSR1
rs10099315	2.91E-63	MSR1
rs10099575	2.71E-64	MSR1
rs10099815	2.81E-65	MSR1
rs10099900	2.83E-65	MSR1

Supplementary Table 6 Protein-protein interactions identified by the STRING Database.

node1	node2	node1_string_id	node2_string_id	phylogenetic_cooccurrence	homology	experimentally_determined_interaction	database_annotated	combined_score
F11	PROC	9606.ENSP00000384957	9606.ENSP00000234071	0.351	0.695	0.078	0.9	0.913
F11	KLKB1	9606.ENSP00000384957	9606.ENSP00000264690	0.436	0.963	0	0.65	0.655
F11	KNG1	9606.ENSP00000384957	9606.ENSP00000265023	0	0	0.473	0.65	0.807
F11	F2	9606.ENSP00000384957	9606.ENSP00000308541	0.282	0.611	0.213	0.9	0.925
F11	SERPINE2	9606.ENSP00000384957	9606.ENSP00000415786	0	0	0.12	0.9	0.908
F2	PROC	9606.ENSP00000308541	9606.ENSP00000234071	0.4	0.741	0.835	0.9	0.984
F2	KNG1	9606.ENSP00000308541	9606.ENSP00000265023	0	0	0.225	0.5	0.596
F2	SERPINE2	9606.ENSP00000308541	9606.ENSP00000415786	0	0	0.696	0.6	0.873
F2	F5	9606.ENSP00000308541	9606.ENSP00000356771	0	0	0.478	0.9	0.945
F5	PROC	9606.ENSP00000356771	9606.ENSP00000234071	0	0	0.237	0.9	0.92
F5	SERPINE2	9606.ENSP00000356771	9606.ENSP00000415786	0	0	0.113	0.9	0.907
KLKB1	KNG1	9606.ENSP00000264690	9606.ENSP00000265023	0	0	0.877	0.9	0.987
PROC	SERPINA1	9606.ENSP00000234071	9606.ENSP00000416066	0	0	0.12	0.8	0.816

Supplementary Table 7 Enrichment pathways among the 20 potential proteins from the PWAS of VTE.

Enrichment pathways	ID	Description	GeneRatio	BgRatio	pvalue	p.adjust	qvalue	geneID	Count
KEGG_COMPLEMENT_AND_COAGULATION_CASCADES	KEGG_COMPLEMENT_AND_COAGULATION_CASCADES	KEGG_COMPLEMENT_AND_COAGULATION_CASCADES	44754	69/5245	3.7588E-11	9.397E-10	8.7046E-10	F2/F11/KLKB1/PROC/KNG1/SERPINA1/F5	7
GOBP_NEGATIVE_REGULATION_OF_COAGULATION	GOBP_NEGATIVE_REGULATION_OF_COAGULATION	GOBP_NEGATIVE_REGULATION_OF_COAGULATION	44732	52/17872	1.6918E-11	2.4658E-09	1.5805E-09	F2/F11/KLKB1/PROC/KNG1/SERPINE2	6
GOBP_REGULATION_OF_COAGULATION	GOBP_REGULATION_OF_COAGULATION	GOBP_REGULATION_OF_COAGULATION	44732	71/17872	1.175E-10	1.3701E-08	8.7819E-09	F2/F11/KLKB1/PROC/KNG1/SERPINE2	6
GOBP_PLATELET_ACTIVATION	GOBP_PLATELET_ACTIVATION	GOBP_PLATELET_ACTIVATION	44701	131/17872	2.7815E-07	1.4742E-05	9.449E-06	F2/PLCG2/PLEK/GP6/SERPINE2	5
GOBP_HUMORAL_IMMUNE_RESPONSE	GOBP_HUMORAL_IMMUNE_RESPONSE	GOBP_HUMORAL_IMMUNE_RESPONSE	44612	312/17872	0.04693881	0.12124703	0.07771544	F2/RARRES2	2

Supplementary Table 8: Lowest p-values for association between SNPs within 1 Mb of each of the 20 causal proteins using summary statistics from the GWAS.

Gene	Lowest GWAS p
F2	1.83E-34
F11	4.94E-37
ABO	2.53E-100
PLCG2	3.29E-09
LRP4	1.83E-34
PLEK	1.89E-08
KLKB1	4.94E-37
PROC	5.83E-07
KNG1	1.63E-04
THBS2	7.10E-06
SERPINA1	1.03E-04
RARRES2	2.89E-04
CEL	2.53E-100
GP6	5.20E-05
SERPINE2	1.18E-04
SERPINA10	1.03E-04
OBP2B	2.53E-100
EFEMP1	1.98E-04
F5	6.04E-194
MSR1	4.05E-04
